# Ribifolones A–H, New Macrocyclic Diterpenes from *Jatropha ribifolia*, Their Cytotoxic Activity and Insights Supported by Network Pharmacology and Molecular Modeling

**DOI:** 10.3390/molecules31101663

**Published:** 2026-05-14

**Authors:** Thalisson Amorim de Souza, Alan Ferreira Alves, Ramon Ramos Marques de Souza, Ana Carolina Ferreira de Albuquerque, Thiago Araújo de Medeiros Brito, Marianna Vieira Sobral, Fernando Martins dos Santos Júnior, Maria de Fátima Agra, Luciana Scotti, Lucas Silva Abreu, Marcus Tullius Scotti, Josean Fechine Tavares, Marcelo Sobral da Silva

**Affiliations:** 1 Health Sciences Center, Graduate Program on Natural and Synthetic Bioactive Products (PgPNSB), Federal University of Paraiba, João Pessoa 58051-900, Paraíba, Brazil; thalisson.amorim@ltf.ufpb.br (T.A.d.S.); alanalves@ltf.ufpb.br (A.F.A.); ramonramos.rm@gmail.com (R.R.M.d.S.); thiagobrito@ltf.ufpb.br (T.A.d.M.B.); mariannavbs@gmail.com (M.V.S.); luciana.scotti@gmail.com (L.S.); marcelosobral@ltf.ufpb.br (M.S.d.S.); 2 Department of Organic Chemistry, Fluminense Federal University, Niterói 24220-900, Rio de Janeiro, Brazil; dealbuquerque.acf@gmail.com (A.C.F.d.A.); fernando_martins@id.uff.br (F.M.d.S.J.); 3 Center of Biotechnology, Federal University of Paraiba, João Pessoa 58050-585, Paraíba, Brazil; agramf@ltf.ufpb.br

**Keywords:** terpenoids, target deconvolution, antiproliferative compounds

## Abstract

Belonging to the Euphorbiaceae family, the *Jatropha* genus is a promising source for the discovery of antitumor compounds. *Jatropha ribifolia* is a traditionally used species in folk medicine in the semi-arid region of Brazil, with a few chemical and pharmacological reports. Based on that, the aim of the current work is to isolate, structurally characterize, and assess the cytotoxic activity of isolated compounds through in vitro and in silico analyses. To achieve these main goals, the underground parts were dried, extracted and purified using classical and instrumental chromatographic techniques, leading to the isolation of 16 compounds. Altogether with HR-ESI-MS, IR, one- and two-dimensional NMR experiments, eight previously unreported diterpenes, named ribifolones A-H, along with eight known compounds, were obtained and are herein described. Regarding their activity against melanoma (SK-MEL-28) and colorectal cancer (HCT-116) cell lines, jatrophone was the most potent with IC_50_ values of 6.19 µM and 10.09 µM, followed by ribifolone C that exhibited a moderate cytotoxicity with IC_50_ values of 50.71 µM and 33.39 µM, respectively. Network pharmacology analysis suggests the involvement of the PI3K-AKT-mTOR pathway in the activity of both compounds; meanwhile, molecular docking and dynamics simulations demonstrate the main interactions with key proteins in the pathway, indicating putative targets. This work opens new perspectives for the discovery of bioactive compounds found in Euphorbiaceae species, especially from those occurring in Caatinga.

## 1. Introduction

Euphorbiaceae is one of the largest genera of flowering plants; among its 340 genera, *Jatropha* includes approximately 175 species [1]. In Brazil, the semi-arid region is home to a diverse range of medicinal species, many of which remain poorly investigated regarding their chemical and pharmacological potential. Among these plants, *J. ribifolia* (Pohl) Bail. stands out as a promising source for the discovery of new bioactive compounds.

**Figure 1:**
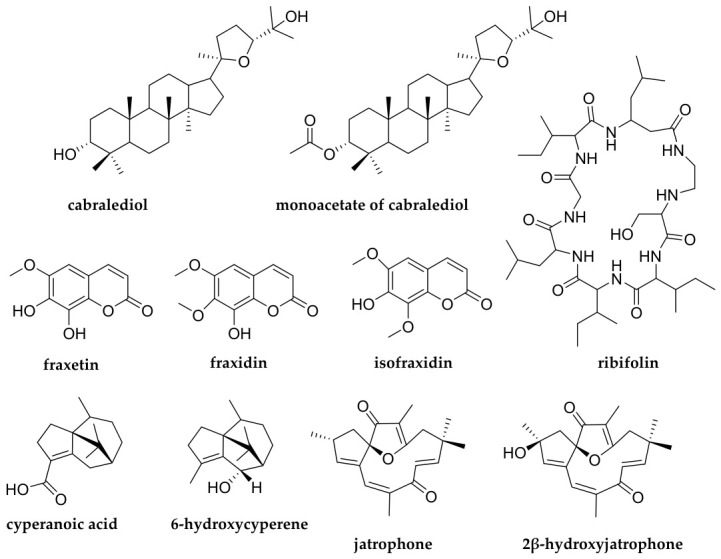
Compounds previously isolated from *J. ribifolia.*

Conventionally known as “pinhão-de-purga”, *J. ribifolia* latex is used throughout northeastern Brazil due to its antivenom activity. Moreover, its seeds are a source for oil production and are also applied in the veterinary field as a purgative [2]. Previous studies report the composition of essential oil and the isolation of ten compounds from this plant, such as coumarins, an orbitide-type peptide and terpenoid derivatives [3,4], as shown in Figure 1. The genus *Jatropha* is an inestimable reservoir of diterpenes with remarkable cytotoxic activity [5]. However, to our knowledge, only two diterpenes were isolated from *J. ribifolia*: jatrophone and 2β-hydroxyjatrophone.

In recent years, the integration of omics sciences with bioinformatics and molecular modeling tools has enabled a more efficient exploration of the chemical space and biological activity associated with natural substances [6]. Based on the principles of polypharmacology, approaches such as network pharmacology have consolidated modern paradigms in the field, allowing not only the rational selection of biological assays to confirm the activity of new chemical entities but also supporting the elucidation of their respective mechanisms of action. These technologies have provided new useful insights for drug repositioning, reinvestigation of classically described natural compounds, and the study of preparations from traditional Chinese medicine [7,8]. Such findings have contributed to highlighting the potential of natural compounds as strategic elements in the development of innovative drugs.

In this context, our research group has been continuously investigating the chemodiversity of the Brazilian Caatinga, unveiling its bioactive potential. Therefore, the main goals of the present work are to isolate, characterize, and evaluate the cytotoxic activity of new macrocyclic diterpenes from *J. ribifolia* through in vitro assays and in silico approaches, including network pharmacology, molecular docking, and molecular dynamics.

## 2. Results

### 2.1. Structural Characterization

Compound **1** was obtained as a yellowish oil. Its molecular formula was determined as C_20_H_25_O_6_ by HR-ESI-MS at *m*/*z* 345.1690 [M − H_2_O + H]^+^ (calcd. for C_20_H_25_O_5_, 345.1697, Δ = 2.0 ppm). The ^13^C NMR spectrum of compound **1** revealed twenty carbon signals. Among them, signals at δ_C_ 212.4 and 202.7 were assigned to ketone and conjugated ketone carbonyl groups, respectively. Additionally, two olefinic carbon signals, two carbinolic carbons, two methylene carbons, and five methyl carbons were observed. In the ^1^H NMR spectrum, methyl hydrogen signals appeared at δ_H_ 1.83 (d, J = 1.3 Hz), 1.16 (s), 1.13 (s), 1.12 (d, J = 6.6 Hz), and 1.11 (s). Olefinic hydrogen signals were detected at δ_H_ 5.63 (bq, J = 1.3 Hz), alongside an oximethinic hydrogen at δ_H_ 4.00 (d, J = 12.0 Hz) and 3.34 (s). Analyses of the NMR combined data (Table 1 and Table 2) were then compared with the literature and allowed the identification of compound **1** as a macrocyclic diterpene analogous to 9β,13α-Dihydroxyisabellione (**9**). Differences between the compounds included the absence of olefinic signals at C-3 and C-4 and the presence of carbinolic signals at δ_C_ 75.6 (C-3) and 64.0 (C-4), indicating oxidation at these positions and the formation of an epoxide group.

**Figure 2:**
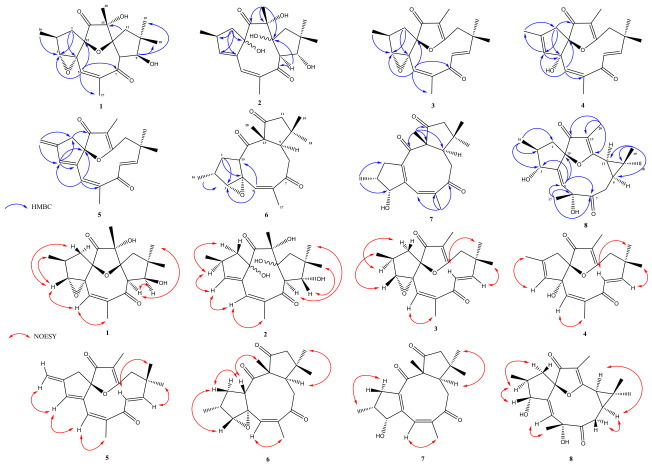
Key correlations of HMBC and NOESY of ribifolones 1–8.

In the HMBC spectrum, correlations were observed between H-5 and the carbons C-3, C-6, C-7, C-15, and C-17; between H-3 and C-1, C-2, C-4, C-15, and C-16; between H3-16 and C-3 and C-1; and between H2-1 and C-3, C-4, and C-14. All chemical shifts in protonated carbons were confirmed by the HSQC spectrum. In the COSY spectrum, correlations were seen between H-3 and H-2, and between H-2 and H3-16, confirming the presence of the epoxide group in this ring, as shown in Figure 2. The NOESY spectra showed cross-peaks between H-3 → H3-16 → H-1β; H-9 → H3-18 → H-11β → H3-20; and H-8 → H3-19, evidencing the relative stereochemistry of compound **1**. The compound was named ribifolone A, a novel natural product. 

Compound **2** was obtained as a yellowish oil. Its molecular formula was determined as C_20_H_28_O_6_ by HR-ESI-MS at *m*/*z* 347.1857 [M − H_2_O + H]^+^ (calcd. for C_20_H_26_O_5_, 347.1853, Δ = −1.3 ppm). The ^1^H and ^13^C NMR analyses of compound **2** revealed twenty carbon signals in the ^13^C NMR spectrum. Similarly, as observed in Ribifolone A, the compound **2** exhibited in the ^13^C NMR spectrum, the presence of two carbonyl groups at δ_C_ 206.8 and 199.8, attributed to ketone and conjugated ketone carbonyl groups, respectively. It also displayed four olefinic carbon signals (as seen in compound **9**) and four carbinolic carbon signals, differentiating it from ribifolone A. Additionally, in DEPT 135 spectrum, two methylenic carbons and five methyl carbons were identified. In the ^1^H NMR spectrum, methyl hydrogen signals were observed at δ_H_ 1.06 (d, J = 5.6), 1.98 (s), 1.22 (s), 0.94 (s), and 1.52 (s), with olefinic hydrogens at δ_H_ 6.15 (d, J = 2.0) and 6.51 (s), and an oxymethine hydrogen at δ_H_ 4.81 (t). The shared similarities between the NMR spectra of ribifolone A (**1**), 9β,13α-Dihydroxyisabellione (**9**), and compound **2** enabled its identification as a macrocyclic diterpene. Distinct differences included signals at δ_C_ 91.0 (C-15) and δ_C_ 91.1 (C-12), indicating lactone cleavage and the presence of two carbinolic carbons.

In the HMBC spectrum, correlations were observed between H2-1 and C-2, C-3, C-4, and C-15, between H3-20 and C-12, C-13 and C-14; and between H-8 and C-7 and C-12. In the COSY spectrum, correlations were seen between H-3 and H-2, H-2 and H3-16, and H2-1, confirming the presence of the double bond in C-3/C-4 (Figure 2). Key NOE correlations between H-2 → H-1ɑ, H-8 → H3-19, and H-9 → H3-18, demonstrate that the relative stereochemistry of compound **2** is similar to compound **1** and **9**. The ^1^H and ^13^C NMR data are shown in Table 1 and Table 2. From this evidence, the structure of compound **2** was proposed as a new diterpene, named ribifolone B.

Compound **3** was obtained as a yellowish oil. Its molecular formula was determined as C_20_H_24_O_4_ by HR-ESI-MS at *m*/*z* 351.1572 [M + Na]^+^ (calcd. for C_20_H_24_NaO_4_, 351.1567, Δ = −1.6 ppm). The ^13^C NMR spectrum of compound **3** revealed twenty carbon signals, including δ_C_ 204.3 and 201.3, corresponding to conjugated ketone carbonyl groups. Additionally, six olefinic carbon signals were observed between δ_C_ 112.2 and 185.3, one of which was an oxygenated double bond, and three carbinolic carbons between δ_C_ 68.4 and 94.8. Analysis of the NMR data (Table 1 and Table 2), along with comparisons to the literature, showed that compound **3** exhibited ^1^H and ^13^C NMR spectra similar to jatrophone (**10**) and the main difference between them was the absence of olefinic signals at C-3 and C-4 (reported for **10**) and the presence of carbinolic signals at δ_C_ 69.2 (C-3) and 68.4 (C-4) in **3**, similar to that observed in compound **1**. This indicated oxidation at these positions and the formation of an epoxide group.

In the HMBC spectrum, correlations were observed between H-5 and carbons C-3, C-7, C-15, and C-17; between H-3 and C-1, C-2, C-4, C-15, and C-16; between H3-16 and C-3 and C-1; between H2-1 and C-3, C-4, and C-14; between H-9 and C-7, C-8, C-18, and C-19; and between H3-20 and C-12, C-13, and C-14. Similarly to compound **1**, in the COSY spectrum, correlations were seen between H-3 and H-2, and between H-2 and H3-16, confirming the presence of the epoxide group in this ring (Figure 2). The NOESY spectra showed cross-peaks between H-3 → H3-16 → H-1β, evidencing the relative stereochemistry of cyclopentane in **3**. From this evidence, the structure of compound **4** was proposed as a new diterpene, named ribifolone C.

Compound **4** was obtained as a yellowish oil. Its molecular formula was determined as C_20_H_24_O_4_ by HR-ESI-MS at *m*/*z* 311.1632 [M − H_2_O + H]^+^ (calcd. for C_20_H_23_O_3_, 311.1642, Δ = 3.3 ppm). Compound **4** featured twenty carbon signals in its ^13^C NMR spectrum. The presence of two carbonyl signals in δ_C_ 204.4 and 202.3 was observed, attributed to conjugated ketone carbonyl groups, eight olefinic carbons, two carbinolic carbons (δ_C_ 93.9 and 97.7), two methylene carbons, and five methyl carbons. Likewise, in compounds **3** and **10**, the presence of one oxygenated double bond in δ_C_ 185.6 indicated structural similarities to jatrophone. In the ^1^H NMR spectrum, five methyl hydrogen signals were observed at δ_H_ 1.95 (d, J = 1.3), 1.73 (s), 1.65 (s), 1.33 (s), and 1.21 (s). Olefinic hydrogens were identified at δ_H_ 6.11 (bs), 6.10 (s), 6.00 (d, J = 13.2 Hz), and 6.5 (d, J = 13.2 Hz). The presence of three methyl groups linked to double bonds and the absence of the characteristic signs of the epoxide group present in compound **3** indicate the presence of a double bond between carbons C-2 and C-3. Furthermore, the signal at δ_C_ 93.9, which is not present in the DEPT 135 spectrum, indicates the presence of a hydroxyl group at C-4.

In the HMBC spectrum, correlations were observed between H-5 and carbons C-3, C-6 and C-15; between H-3 and C-1, C-2, C-4, C-15, and C-16; between H3-16 and C-1 and C-3; and between H2-1 and C-2, C-3, C-4, C-14, and C-16. All chemical shifts in protonated carbons were confirmed by the HSQC spectrum. In the COSY spectrum, correlations were observed between olefinic hydrogens H-8 to H-9 and H-5 to H-17, confirming the presence of two other double bonds, one tri-substituted and the other tetra-substituted (Figure 2). From this evidence, the structure of compound **4** was proposed as a new diterpene, named ribifolone D.

Compound **5** was obtained as a yellowish oil. Its molecular formula was determined as C_20_H_22_O_3_ by HR-ESI-MS at *m*/*z* 311.1637 [M + H]^+^ (calcd. for C_20_H_23_O_3_, 311.1642, Δ = 1.5 ppm). Compound **5** exhibited twenty carbon signals in its ^13^C NMR spectrum, including δ_C_ 203.2 and 201.9, corresponding to two conjugated ketone carbonyl groups. Additionally, ten olefinic carbon signals and four methyl carbon signals were observed. The ^1^H NMR spectrum displayed four methyl hydrogen signals at δ_H_ 1.98 (d, J = 0.8), 1.77 (s), 1.36 (s) and 1.23 (s). As observed previously, the presence of an oxygenated double bond at δ_C_ 185.3 indicates the presence of a jatrophane-type structure, similar to compounds **3**, **4**, and **10**. Differences between compound **5** and jatrophone (**10**) were indicated by signals at δ_C_ 148.9 (C-2) and δ_C_ 109.6 (C-16), suggesting the presence of an exocyclic double bond. This was supported by hydrogen signals at δ_H_ 5.10 (bs) and 5.25 (bs).

In the HMBC spectrum, correlations were observed between H-3 and carbons C-2, C-4, and C-15; between H3-16 and C-1 and C-3; between H-9 and C-7, C-11, and C-19 (Figure 2). In the COSY spectrum, there were observed correlations between H-8 and H-9, H-5 and H3-17, and H3-16 to H2-1 (Figure 2). From this evidence, the structure of compound **5** was proposed as a new diterpene, named ribifolone E.

Compound **6** was obtained as a yellowish oil. Its molecular formula was determined as C_20_H_26_O_4_ by HR-ESI-MS at *m*/*z* 331.1903 [M + H]^+^ (calcd. for C_20_H_27_O_4_, 331.1904, Δ = 0.1 ppm). Compound **6** displayed twenty carbon signals in its ^13^C NMR spectrum, including three carbonyl signals at δ_C_ 214.7, 211.5, and 211.2, corresponding to two ketones and one conjugated ketone. Additionally, olefinic carbon signals were detected at δ_C_ 139.1 and 129.1. These signals suggest a different skeletal structure compared to previous compounds. Analysis of the spectroscopic data, supplemented by a comparison with the literature, revealed that compound **4** closely resembled citlalitrione (**13**), with differences in the signals for carbons C-3, C-4, and C-16 (Table 1 and Table 2). A previous study targeting the total synthesis of citlalitrione resulted in two derivatives showing ɑ and β epoxy moieties, between C-3 and C-4. One of them was citlalitrione, which bears a β-epoxy group displaying chemical shifts at δ_C_ 72.5 and 67.8, similarly to those observed for compound **13**. Meanwhile, the other compound, bearing the ɑ-epoxy group, showed chemical shifts at δ_C_ 65.8 and 66.8, likewise those observed for compound **6**. Based on that feature, the stereochemical inversions of the epoxide between C-3 and C-4 were proposed [9]. The NOESY spectrum showing crossed peaks between H-3 → H3-16, H-15 → H3-20 confirmed that the hydrogens are in the same plane, and crossed peaks between H-9 and methyls 17, 18, and 19. The compound **6** was named ribifolone F, a novel macrocyclic diterpene.

Compound **7** was obtained as a yellowish oil. Its molecular formula was determined as C_20_H_26_O_4_ by HR-ESI-MS at *m*/*z* 313.1798 [M − H_2_O + H]^+^ (calcd. for C_20_H_25_O_3_, 313.1798, Δ = 1.4 ppm). Compound **7** displayed twenty carbon signals in its ^13^C NMR spectrum, including three carbonyl signals at δ_C_ 214.7, 211.3, and 209.1, corresponding to two ketones and one conjugated ketone. These findings suggested a resemblance to compounds **6** and **13**, a cyclojatrophane. The distinction between compound **7** and the previously identified substance was observed in four olefinic carbon signals at δ_C_ 125.2 (C-5), 143.6 (C-6), 143.0 (C-4), and 139.7 (C-15), the absence of a carbinolic hydrogen signal at δ_H_ 3.4 (C-15), and differences in the chemical shift in the oxymethine proton from δ_H_ 3.16 (s) to δ_H_ 4.27 (d, J = 4.0). The shift in the C-3 carbon signal from δ_C_ 65.8 to δ_C_ 86.9 further supported this distinction.

In the HMBC spectrum, correlations were observed between H3-16 and C-1 and C-3; between H3-17 and C-5, C-6, and C-7; and between H3-20 and C-9, C-12, C-13, and C-14. (Figure 2). The NOESY spectrum showed crossed peaks between H-9 → H3-19, H3-20 → H-11 → H3-18, as also observed in compounds **6** and **13**. From this evidence, the structure of compound **7** was proposed as a new diterpene, named ribifolone G.

Compound **8** was obtained as a yellowish oil. Its molecular formula was determined as C_20_H_26_O_5_ by HR-ESI-MS at *m*/*z* 369.1672 [M + Na]^+^ (calcd. for C_20_H_26_NaO_5_, 369.1672, Δ = 0.1 ppm). The ^13^C NMR spectrum of compound **8** revealed twenty carbon signals. Among them, two signals at δ_C_ 208.5 and 203.4 were assigned to ketone and conjugated ketone carbonyl groups, respectively. Additionally, one olefinic carbon signal, one carbinolic carbon, two methylene carbons, and five methyl carbons were observed. In the ^1^H NMR spectrum, methyl hydrogen signals appeared at δH 1.83 (d, J = 1.3 Hz), 1.16 (s), 1.13 (s), 1.13 (d, J = 6.7 Hz), and 1.11 (s). Olefinic hydrogen signals were detected at δ_H_ 5.70 (ql, J = 1.3 Hz), alongside oximethinic hydrogens at δ_H_ 4.06 (d, J = 12.0 Hz), as shown in Table 1 and Table 2.

In the HMBC spectrum, key correlations were observed between the olefinic proton H-5 and carbons C-3, C-7, C-15, and CH3-17. Additionally, the correlations observed from the methyl groups were also important to structural elucidation; among them, CH3-16 and C-1, C-2, and C-3, CH3-17 and C-5, C6, and C-7, between CH3-18 and C-9 and C10 and C-11, and similar correlations of CH3-20 and C-12, C-13, and C14. The chemical shifts attributed to protonated carbons were confirmed by the HSQC spectrum. In the COSY spectrum, correlations were seen between CH3-16 and H-2, and H-2 to H-3, confirming the presence of a hydroxyl group in C-3. Additionally, the correlations between H-8 and H-9 and H-9 and H-11 support the presence of a cyclopropane ring in the structure. The NOESY spectra showed cross-peaks between H3 → H3-16 → H-1β; H-9 → H3-18 → H-11β → H3-20; and H-8 → H3-19, evidencing the relative stereochemistry of compound **1**.

Based on the chemical shifts, comparison with the published literature and the observed correlations, the obtained data indicated structural similarities between compound **8** and multifidanol, a lathyrane diterpene previously isolated from *J. multifida* [10]. Differing from this compound, **8** exhibits the additional signal at 208.5 attributed to C-7, the chemical shifts attributed to CH_3_-20, C-12, and C-15 confirmed the presence of a substituted 2-oxolane ring, as previously observed in ribifolones C, D, and E and in other jatrophane-type diterpenes isolated from *J. ribifolia*. These structural features result in a rare combination of motifs within the molecule. Considering this set of evidence, the structure of compound **8** was proposed as a new diterpene, named ribifolone H.

**Figure 3:**
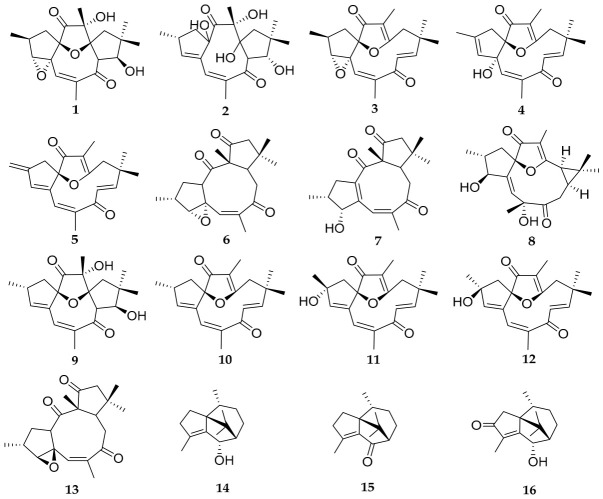
Isolated compounds from *J. ribifolia* roots.

Among the eight new diterpenes described, five of them are jatrophanes, one belongs to the lathyrane class, and the last two are rare cyclojatrophane derivatives. Only two other 9,13-cyclojatrophane compounds have been previously reported in the *Jatropha* genus, namely citlalitrione and jatrophatrione [11,12]. These findings highlight the chemodiversity contained in plant species from the Brazilian semi-arid region. In this context, the chromatographic fractionation of the hexane extract of *J. ribifolia*’s roots allowed the isolation of ribifolones A–H and eight previously described compounds: 9β,13α-dihydroxyisabellione (**9**), jatrophone (**10**), 2α and 2β-jatrophone (**11**–**12**), citlalitrione (**13**), (6S)-patchoulan-4-en-6-ol (**14**), patchoulenone (**15**), and sugeonol (**16**). Their structures are depicted in Figure 3 [4,9,13,14].

### 2.2. Biological Assay

After structural elucidation, ribifolones A–H were evaluated against two human tumor cell lines. The selected SK-MEL-28 (melanoma) and HCT-116 (colorectal cancer) were subjected to the MTT reduction assay after 72 h of treatment with the compounds. Table 3 presents the corresponding results.

In the set of the new compounds tested, only three of them exhibited cytotoxicity. Demonstrating a moderate activity, ribifolone C achieved IC_50_ values of 50.71 and 33.39 µM against melanoma and colorectal cancer cells, respectively. Ribifolone A inhibited cell viability only against melanoma (IC_50_ = 32.25 µM). Meanwhile, ribifolone D acted on both tumor lines, although with lower potency (IC_50_ = 95.64 and 75.35 µM) than ribifolone C. Ribifolones B and E–H, were considered inactive, as shown in Table 3.

Known by its high cytotoxicity against different cancer cell types, jatrophone is the major compound found in the roots of *J. ribifolia* [4]. To compare how structure modifications could reflect on the biological activity, jatrophone was also tested and displayed the strongest activity among the series (IC_50_ = 6.19 µM in SK-MEL-28 and IC_50_ = 10.09 µM for HCT-116).

The evaluation of the selectivity index (SI) is a crucial step in the early stages of drug discovery. However, many previously published studies report the bioactivity of medicinal plants and their isolated compounds without providing SI data, which represents a limitation for further pharmacological development [15]. In this context, compounds that exhibited cytotoxic activity were further evaluated in HEK-293 cells, a non-tumoral human embryonic kidney cell line. Regarding selectivity, ribifolones A and C–D showed IC_50_ values > 100 µM, indicating low cytotoxicity toward normal cells, as shown in Table 3. Consequently, SI values were calculated for jatrophone, which was 3.28-fold more selective for SK-MEL-28 cells and 2.01-fold more selective for HCT-116 cells.

The literature reports many diterpenes as remarkable antitumor compounds, among them lathyrane and jatrophane typically occur in *Jatropha* species [16,17]. In the current study, only the jatrophane-type derivatives exhibited cytotoxic activity. Compared to jatrophone, the results imply that differences in oxidation patterns, such as the presence of epoxide between C-2 and C-3 and hydroxyl groups in C-4 and C-9, constitute a key factor in modulating the bioactivity. Furthermore, structural changes such as lactone cleavage, variations in the size of the macrocyclic ring, and substitutions in the double bonds may have contributed to the weaker antitumor activity of ribifolones against the selected cell lines.

Despite some previous reports demonstrating that the acetoxy group at C-9, the benzoyl moiety occupying position 3, and the propyl group at C-8 decrease cytotoxicity of some jatrophane diterpenes, many aspects about this topic remain unclear, especially due to the diversity of tumor lineages presented in each work [18,19]. Based on that, complementary studies designed with a larger number of analogs and well-defined cell lines are required to obtain suitable data to develop QSAR models.

Regarding their mechanisms of action, despite being recognized as promising scaffolds, macrocyclic diterpenes from *Jatropha* remain largely unexplored [20]. In this context, the use of modern approaches such as network pharmacology and molecular modeling, including docking and dynamics, has become increasingly common in the study of natural products. The combination of these methods also assists in identifying pharmacophoric groups and other key properties relevant to the development of pharmacological studies, whether in vitro or in vivo models.

### 2.3. Pharmacological Network and Molecular Modeling

To explore putative molecular targets underlying the effects of the active compounds, a combination of in silico approaches was employed. Based on the activity of ribifolone C and jatrophone against both cell lines, a network pharmacology workflow was applied to colorectal and melanoma cancers. In this context, the analyses performed using the PharmMapper and AiA platforms identified 271 proteins as potential targets for the compounds, whereas GeneCards retrieved 3485 and 2290 genes associated with colorectal and melanoma cancers, respectively. The intersection analysis identified proteins encoded by disease-associated genes that overlapped with the predicted protein targets, yielding 118 candidates for colorectal cancer and 80 for melanoma.

**Figure 4:**
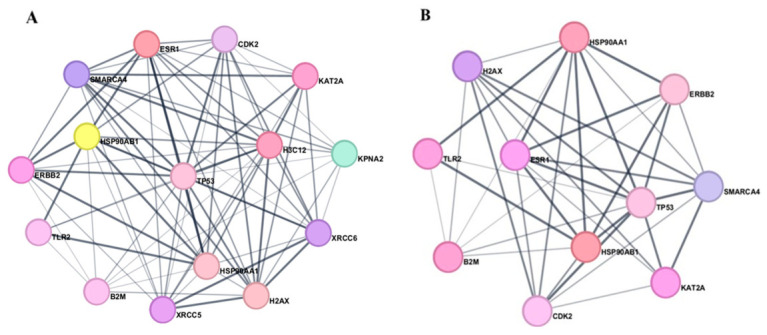
Network pharmacology networks of ribifolone C and jatrophone in colorectal cancer (**A**) and melanoma (**B**), emphasizing highly connected hub proteins. Node colors were applied exclusively for visual differentiation.

For both cancer types, the resulting networks exhibited high interconnectivity, with average node degrees of 8.73 for colorectal cancer and 8.25 for melanoma, indicating a complex pattern of molecular interactions. Several hub genes present degrees equal to or greater than 15, suggesting that these nodes may represent key points of functional convergence within cancer-related pathways. In network pharmacology, highly connected nodes are often considered topologically influential, as their modulation may affect multiple interconnected biological processes rather than a single signaling route. Accordingly, the application of a degree threshold of 17 allowed the prioritization of the most interconnected targets, highlighting a subset of proteins that may contribute to the biological effects observed for ribifolone C and jatrophone. This network supports the hypothesis that its activity may be mediated through a multi-target mechanism, as illustrated in Figure 4.

**Figure 5:**
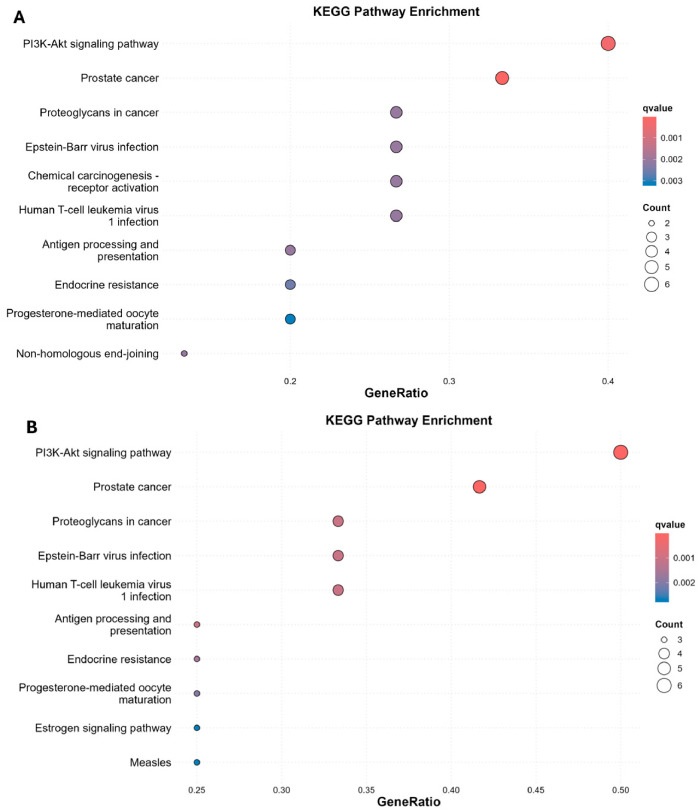
KEGG pathway enrichment analysis of colorectal (**A**) and melanoma (**B**) cancer-associated genes overlapping with the predicted targets. The x-axis shows the GeneRatio, circle size represents Count, and color indicates the q-value, with red corresponding to higher enrichment significance.

The Kyoto Encyclopedia of Genes and Genomes (KEGG) is a curated resource that links genes and proteins to canonical biological pathways, enabling functional interpretation of disease-associated gene sets. In this context, the observed enrichment suggests that a considerable fraction of genes associated with these cancers, whose encoded proteins overlap with the predicted targets of ribifolone C and jatrophone, converge on the PI3K–Akt–mTOR axis. As shown in Figure 5, KEGG enrichment analysis indicated that the PI3K–Akt–mTOR signaling pathway presented the highest GeneRate value in both colorectal and melanoma networks. Notably, this pathway is frequently deregulated in cancer and is known to regulate essential cellular processes, including proliferation, survival, growth, and metabolism [21,22].

Given the prominence of the PI3K–Akt–mTOR signaling pathway as a potential component associated with the selected compounds, molecular docking simulations were performed focusing on key proteins within this pathway. The results are summarized in Table 4. Docking scores were used to estimate the binding energies of the complexes formed between the ligands and the selected targets, with more negative values indicating greater complex stability [23]. The ligand efficiency (LE) was calculated as the ratio of docking score to the number of heavy atoms, as described in the Methods Section.

Regarding the interaction profiles between the diterpenes and the target receptor, docking analysis indicated that the crystallized compound WAY-697 achieved the most favorable binding score (−113.65) against ERβ when compared with ribifolone C (−103.41) and jatrophone (−106.90). Nevertheless, ligand efficiency analysis revealed that WAY-697 exhibited the lowest LE value (3.67), whereas jatrophone (4.65) and ribifolone C (4.31) displayed higher efficiencies. WAY-697 was employed as the reference ligand, as it is a potent and selective ERβ agonist with an IC_50_ value of 1.9 nM.

**Figure 6:**
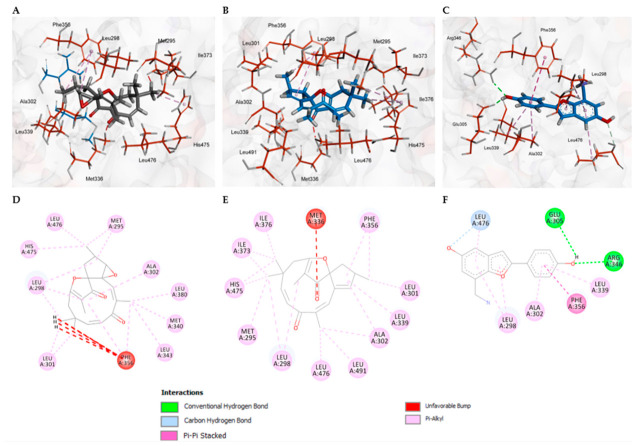
Amino acid interactions formed between ribifolone C (**A**,**D**), jatrophone (**B**,**E**), and the reference ligand WAY-697 (**C**,**F**) against ERβ.

Both diterpenes displayed comparable binding patterns, dominated by hydrophobic interactions with residues Met295, Leu298, Leu301, Ala302, Phe356, His475, and Leu476 (Figure 6). However, ribifolone C formed a greater number of unfavorable contacts (Phe356) compared to jatrophone (Met336), which may account for the differences observed in docking scores and LE values. WAY-697 interacted with ERβ through hydrophobic contacts involving Leu298, Ala302, and Phe356, and established three hydrogen bonds: one conventional with Leu476, and two additional ones with Glu305 and Arg346. The agonist shared four key interactions with ribifolone C and jatrophone (Ala302, Leu339, and Leu476), which may represent critical residues associated with agonistic activity, as shown in Figure 6. Detailed information on the interaction profiles of ribifolone C, jatrophone, and the other selected targets is provided in the Supplementary Materials.

Estrogen receptors (ERs) are nuclear receptors frequently associated with the pathophysiology of cancer [24], and exist in two main isoforms, ERα and ERβ. ERα is commonly linked to tumor cell proliferation and survival, whereas ERβ generally acts as a tumor suppressor, downregulating processes such as proliferation, differentiation, angiogenesis, and immune invasion. Accumulating evidence suggests that progression of different types of cancer may be influenced by estrogen receptor expression [25,26,27].

In colorectal cancer, ER expression exhibits distinct regulatory patterns. ERβ is frequently downregulated, a feature that may contribute to increased cancer cell proliferation. Conversely, ERα, which is typically expressed at low levels in healthy colon tissue, tends to be upregulated, particularly through activation of the PI3K/Akt signaling pathway. In melanoma, the role of ERα remains less clearly defined, whereas ERβ predominates and exerts tumor-suppressive effects by inhibiting PI3K/Akt signaling, thereby restraining cell proliferation and promoting apoptosis [28].

Given that molecular docking analyses indicated more favorable binding scores for ribifolone C and jatrophone toward ERβ when compared with the reference agonist WAY-697, molecular dynamics (MD) simulations were subsequently performed to assess the stability and conformational behavior of the resulting complexes over time. For this purpose, root mean square deviation (RMSD), root mean square fluctuation (RMSF), and interaction energy values were calculated for ERβ complexes with ribifolone C, jatrophone, and WAY-697. The results of the MD simulations are summarized in Table 5.

The results of the MD simulations demonstrate that WAY-697 exhibits the most favorable total interaction energy (−210.13 kJ·mol^−1^), primarily driven by electrostatic contributions calculated through Coulombic forces. In contrast, for ribifolone C, the total interaction energy (−146.76 kJ·mol^−1^) and hydrophobic interactions represent the major stabilizing contribution, followed by electrostatic forces. A similar interaction pattern was observed for jatrophone, although with a less favorable total energy (−103.82 kJ·mol^−1^). This energetic profile is consistent with the lipophilic nature of the diterpenes and the predominantly hydrophobic binding environment of ERβ. Rather than indicating unexpected interaction features, these results support the docking, confirming that the predicted binding modes are energetically sustained over the simulation time.

**Figure 7:**
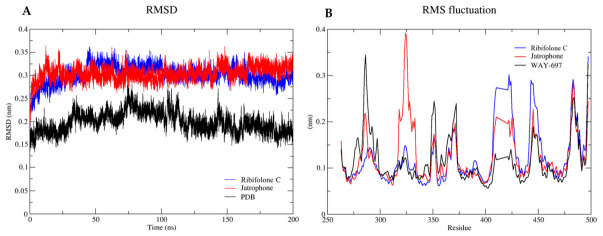
Molecular dynamics simulation analysis of ERβ complexes with ribifolone C, jatrophone, and the reference ligand WAY-697. Interaction energy plots: (**A**) root mean square deviation (RMSD) and (**B**) root mean square fluctuation (RMSF) profiles of the protein–ligand complexes over the simulation time.

In this context, the complementary outcomes of molecular dynamics and docking normalization analyses indicate that WAY-697 exhibits greater dynamic stability, whereas ribifolone C and jatrophone display higher ligand efficiency. As illustrated in Figure 7, the RMSD and RMSF profiles reflect these distinct dynamic behaviors of the ERβ complexes, supporting an interpretation in which the diterpenes present efficient binding characteristics, while the co-crystallized ligand maintains enhanced structural stability within the ERβ binding site.

The results obtained from network pharmacology and molecular modeling analyses suggest the involvement of the PI3K–AKT–mTOR signaling pathway in the activity of ribifolone C and jatrophone, with estrogen receptors, particularly ERβ, emerging as potential targets for these compounds. Such receptors play a key role in the pharmacotherapy of different types of cancer, including breast and prostate cancer. Previous studies have demonstrated the cytotoxic activity of jatrophone against MCF-7 cells (IC_50_ = 1.8 µM), a resistant breast cancer cell line [4]. Regarding its mechanism of action in breast cancer, jatrophone has been shown to act by targeting the PI3K/AKT/NF-κB and Wnt/β-catenin signaling pathways [21,22]. Despite promising results, experimental validation is still required, as the complexity inherent to biological systems makes their precise in silico simulation challenging; therefore, validation using experimental data is essential to determine both the predictive ability and applicability of a computational model [29,30].

## 3. Materials and Methods

### 3.1. General Experimental Procedures

Isolation and purification were performed by different chromatographic methods, including column chromatography (CC) and thin-layer chromatography (TLC). Silica gel (particle size of 0.040–0.063 mm, Silicycle, Zeochem, Quebec, QC, Canada) was used for CC. Commercial silica gel (Whatman, Cytiva, Marlborough, MA, USA) plates were used in TLC in layers with a thickness of 0.25 mm on an aluminum support (20 × 20 cm). The spots in TLC were analyzed by using ultraviolet radiation at wavelengths of 254 and 366 nm (Spectroline^®^, Supelco, Sigma-Aldrich, St. Louis, MO, USA). Additionally, a high-performance liquid chromatography (HPLC) apparatus coupled with a photodiode array detector (PDA) was also used (Shimadzu Proeminence, Shimadzu, Kyoto, Japan). The separations were carried out in an analytical C18 (YMC; 250 mm × 4.6 mm × 5 μm) and preparative C18 (YMC; 250 mm × 21.2 mm × 5 μm) columns (YMC Europe GmbH, Dinslaken, Germany). The mobile phases were composed of ultrapure water (A) (Milli-Q^®^, MA, USA) and Acetonitrile HPLC grade (B) (Supelco, Sigma-Aldrich, MO, USA).

The IR spectra were recorded on a Fourier transform infrared spectrophotometer (Shimadzu IRSpirit-T, Shimadzu, Kyoto, Japan) using the QATR-S accessory. The 1D and 2D NMR experiments were conducted using two NMR spectrometers, the Bruker Ascend 400 and 100 MHz for ^1^H and ^13^C, and Bruker AvanceNeo 500 and 125 MHz (Bruker, Billerica, MA, USA). The ^1^H and ^13^C NMR chemical shifts were referenced to the solvent peaks for residual internal CDCl_3_. A mass spectrometer (MicroTOF II, Bruker Daltonics, Billerica, MA, USA) with an electrospray ion source (ESI) was used to perform ESI-TOF-MS analysis.

### 3.2. Plant Material

The botanical material of the species was collected in the city of Serra Branca-PB in March 2016 and identified by Prof. Maria de Fátima Agra. The voucher specimen was deposited at the Lauro Pires Xavier Herbarium (UFPB) under voucher number 123491. The access registration in the National System for the Management of Genetic Heritage and Associated Traditional Knowledge (SISGEN) was obtained under the number A22E9B0.

### 3.3. Extraction and Isolation

The underground parts were dried at room temperature for 72 h, yielding 245.0 g. The botanical material was then ground and macerated with hexane (3 L) for 7 days, with this process repeated three times. The extract solutions were filtered and concentrated under reduced pressure at 40 °C, resulting in 2.53 g of hexane extract from the roots (JRRHx). After that, the process of extraction was repeated with methanol, resulting in the roots’ methanolic extract.

Initially, 2.45 g of JRRHx was subjected to column chromatography (CC) using silica gel 60 (0.063–0.2 mm) as the stationary phase. The extract was eluted with hexane, ethyl acetate, or binary mixtures in a gradient of increasing polarity; 150 mL were collected for each fraction, and the column was completed with pure methanol, yielding 33 fractions. The fractions were then analyzed by TLC, using plates eluted with a hexane and ethyl acetate gradient and visualized under UV light at 296 and 364 nm. Subsequently, the fractions were analyzed by analytical-scale HPLC with exploratory gradients ranging from 50% to 100% acetonitrile (ACN) and water over 60 min. The fractions were also subjected to 1H NMR analysis in CDCl3. Fraction FR09 (700 mg) showed a single band on TLC and was directly analyzed by NMR. Meanwhile, the remaining fractions were grouped and analyzed by HPLC and 1H NMR to identify their similarity, resulting in the following sets: FR3-5 (135.0 mg), FR11-13 (352 mg), FR-15-16 (50 mg) and FR20-22 (95 mg).

All mentioned samples were subsequently subjected to exploratory analyses on analytical HPLC and further fractionated on semipreparative-scale HPLC after method optimization. The chromatographic conditions used for isolation in each sample are provided as follows: For FR3-5, the chromatography run begins with 0.0–40.0 min (70–100% of B), 40.0–60.0 min (100–100% of B), and 60.0–65.0 min (100–70% of B), with a volume injection of 100 µL and flow of 3 mL/min. For a fraction encoded as FR11-13, it was a gradient ranging from 0.0 to 75.0 min (35–100% of B), 75.0 to 85.0 min (100–100% of B), and 85.0 to 90.0 min (100–35% of B). In FR-15-16, the system was settled as 0.0–40.0 min (10–80% of B), 40.0–45.0 min (80–100% of B), 45.0–60.0 min (100–100% of B), and 60.0–65.0 min (100–100% of B). For fraction FR20-22, the elution was carried out from 0.0 to 40.0 min (35% of B), 40.0 to 45.0 min (35–45% of B), 45.0 to 75.0 min (45% of B), 75.0 to 80.0 min (45–100% of B), 80.0 to 90.0 (100–100% of B), and 90.0 to 95 (100–35% of B). For the last three samples, the volume injection of 100 µL and the flow rate of 8 mL/min were used.

### 3.4. Cytotoxicity Assay

The cytotoxicity of the ribifolones A-H was evaluated using the MTT (3-(4,5-dimethylthiazol-2-yl)-2,5-diphenyltetrazolium bromide) reduction assay [31]. SK-MEL-28 (human melanoma), HCT-116 (human colorectal carcinoma), and HEK-293 (non-tumor human embryonic kidney) cells were seeded in 96-well plates at densities of 1 × 10^5^, 3 × 10^5^ and 2 × 10^5^ cells/mL, respectively. After a 24 h pre-incubation period to allow cell attachment, the cells were treated with ribifolones A-H at concentrations ranging from 1.56 to 100 µM and incubated for 72 h. Subsequently, MTT solution (5 mg/mL) was added to each well, and the plates were incubated for an additional 4 h at 37 °C and 5% of CO_2_. The resulting formazan crystals were solubilized overnight with one hundred µL of 10% sodium dodecyl sulfate (SDS) solution [32]. Absorbance was measured at 570 nm using a microplate spectrophotometer. All experiments were performed in triplicate, and the half-maximal inhibitory concentration (IC_50_) values were determined by nonlinear regression analysis using GraphPad Prism 8.0.1 software.

### 3.5. Network Pharmacology

Initially, potential pharmacological targets for ribifolone C were collected using the AmIActive (AiA) and PharmMapper platforms [33,34]. Subsequently, the main targets involved in the onset and progression of colorectal and melanoma cancer were obtained through the GeneCards platform [35]. Next, the common targets between rib-3 and selected types of cancer were identified. These overlapping targets were used to construct two molecular interaction networks, one for colorectal cancer and the other for melanoma. For this purpose, the Search Tool for the Retrieval of Interacting Genes/Proteins (STRING) database was employed [36], considering a confidence score cutoff of 0.4 for protein–protein interactions.

The Cytoscape software (v. 3.10.3) was used for visualization and analysis of the constructed drug–disease networks [37]. Subsequently, functional enrichment analysis was performed to investigate the main signaling pathways associated with the common targets. For this, the protocol provided by the National Cancer Institute was followed, employing the clusterProfiler package in the R environment, focusing on the KEGG (Kyoto Encyclopedia of Genes and Genomes) category [38,39]. A significance criterion of *p* < 0.05 was adopted. Finally, the targets present in the main enriched pathways, as well as those described as relevant to the pathophysiology of melanoma and colorectal cancers, were selected for molecular docking and molecular dynamics studies.

### 3.6. Molecular Docking

Based on in vitro assays, ribifolone C was selected for docking analysis; its structure was modeled using MarvinSketch v.25.3.0 software [23]. The targets were selected according to the highest number of interactions in the network analysis. Then, the crystallographic structures of four targets were obtained from the Protein Data Bank (PDB) (Table 6). Two compounds were used as standards for comparison: the cocrystallized inhibitor and the known active molecule jatrophone.

Molegro Virtual Docker v. 2013.6.0.1 was used to perform molecular docking [45]. All water molecules and cofactors were removed, and prior to molecular docking, a redocking step was carried out to assess the accuracy and reliability of the results using the root mean square deviation (RMSD), and values ≤ 2 Å were considered satisfactory. The PI3K-Akt-mTOR pathway was analyzed using the PI3Kγ isoform, available in its cocrystallized form.

The simulation was conducted using default settings. The MolDock Score function was used to evaluate ligand poses, considering internal energy, hydrogen bonds, and torsional energy contributions. Fifty runs were performed using the MolDock SE algorithm, and the five best poses were retained. A grid with a radius of 15 Å and a resolution of 0.30 Å was generated, centered on the positions of the crystallographic ligands in the selected proteins. The docked poses were subsequently analyzed using Discovery Studio Visualizer v. 21.1.0.20298 [46].

Ligand efficiency (LE) was calculated for all ligands [47]. Larger molecules tend to establish more contact points with the target, and, therefore, the docking score may reflect these multiple interactions rather than the intrinsic chemical efficiency of the molecule. LE is a metric in which the docking score is normalized by the number of heavy atoms, reflecting the actual effectiveness of the compound. LE was calculated according to Equation (1), where nHA denotes the number of heavy atoms.

(1) $$LE=\frac{Docking\;score}{nHA}$$

### 3.7. Molecular Dynamics

Molecular dynamics (MD) simulations were performed using GROMACS 5.0 [48] to evaluate the stability of the protein–ligand complexes. Ligand topologies were generated with the Automated Topology Builder (https://atb.uq.edu.au/index.py, accessed on 31 January 2025), applying the same force field as used for the protein, GROMOS54a7 [49].

Each system was solvated in a dodecahedron box of water molecules using the SPC extended point charge model, and sodium ions were added to neutralize the overall charge. Energy minimization was carried out to remove steric clashes, followed by equilibration in two stages. First, 100 ps of NVT equilibration was performed at 300 K using the velocity rescaling thermostat, followed by 100 ps of NPT equilibration at 1 atm using the Parrinello–Rahman barostat to stabilize the system. MD simulations were then performed for 200 ns with a timestep of 2 fs under periodic boundary conditions. The structural stability of the complexes was assessed by calculating the root mean square deviation (RMSD), while the flexibility of individual residues was evaluated using root mean square fluctuation (RMSF) analysis. The energy interaction of the protein–ligand complex was also calculated using the short-range Coulombic interaction energy and the Lennard–Jones short-range energy for 200 ns.

**Ribifolone A (1)**

Yellowish oil; UV (ACN) λmax 206, 213, 223, and 256 nm; IR (ATR) νmax 3529, 3406, 2961, 2929, 2877, 1754, and 1651 cm^−1^; ^1^H and ^13^C NMR data, see Table 1 and Table 2; and positive-ion HR-ESI-MS *m*/*z* 345.1690 [M − H_2_O + H]^+^ (calcd for C_20_H_25_O_5_^+^, 345.1697, Δ*_m_*_/*z*_ theoretical = 2.0 ppm).

**Ribifolone B (2)**

Yellowish oil; UV (ACN) λmax 193, 208, 243, and 279 nm; IR (ATR) νmax 3458, 3363, 2938, 2923, 2868, 2854, 1706, 1683, 1372, and 1054 cm^−1^; ^1^H and ^13^C NMR data, see Table 1 and Table 2; and positive-ion HR-ESI-MS *m*/*z* 347.1857 [M − H_2_O + H]^+^ (calcd for C_20_H_26_O_5_^+^, 347.1853, Δ*_m_*_/*z*_ theoretical = −1.3 ppm).

**Ribifolone C (3)**

White amorphous powder; UV (ACN) λmax 201, 219, 247, and 279 nm; IR (ATR) νmax 2964, 2941, 2895, 1746, 1691, 1223, and 1036 cm^−1^; ^1^H and ^13^C NMR data, see Table 1 and Table 2; positive-ion HR-ESI-MS *m*/*z* 351.1572 [M + Na]^+^ (calcd for C_20_H_24_NaO_4_, 351.1567, Δ*_m_*_/*z*_ theoretical = −1.6 ppm).

**Ribifolone D (4)**

Yellowish oil; UV (ACN) λmax 218 and 277 nm; IR (ATR) νmax 3420, 2961, 2929, 2872, 1697, 1622, and 1370 cm^−1^; ^1^H and ^13^C NMR data, see Table 1 and Table 2; and positive-ion HR-ESI-MS *m*/*z* 311.1632 [M − H_2_O + H]^+^ (calcd. for C_20_H_23_O_3_^+^, 311.1642, Δ*_m_*_/*z*_ theoretical = 3.3 ppm).

**Ribifolone E (5)**

Yellow oil; UV (ACN) λmax 216 and 278 nm; IR (ATR) νmax 2958, 2926, 2872, 1703, and 1619 cm^−1^; ^1^H and ^13^C NMR data, see Table 1 and Table 2; and positive-ion HR-ESI-MS *m*/*z* 311.1637 [M + H]^+^ (calcd for C_20_H_23_O_3_^+^; 311.1642, Δ*_m_*_/*z*_ theoretical = 1.5 ppm).

**Ribifolone F (6)**

White amorphous powder; UV (ACN) λmax 193,237 and 245 nm; IR (ATR) νmax 3015, 2964, 2877, 1749, 1691, 1223, and 1042 cm^−1^; ^1^H and ^13^C NMR data, see Table 1 and Table 2; and positive-ion HR-ESI-MS *m*/*z* 331.1903 [M + H]^+^ (calcd for C_20_H_27_O_4_^+^, Δ*_m_*_/*z*_ theoretical = 0.1 ppm).

**Ribifolone G (7)**

Yellowish oil; UV (ACN) λmax 193, 225 and 282 nm; IR (ATR) νmax 3437, 2958, 2926, 2872, 1746, 1235, and 1077 cm^−1^; ^1^H and ^13^C NMR data, see Table 1 and Table 2; and positive-ion HR-ESI-MS *m*/*z* 313.1798 [M − H_2_O + H]^+^ (calcd for C_20_H_25_O_3_^+^, 313.1798, Δ*_m_*_/*z*_ theoretical = 1.4 ppm).

**Ribifolone H (8)**

Yellowish oil; UV (ACN) λmax 200, 225, 273, and 394 nm; IR (ATR) νmax 3630, 3413, 3235, 1638, 1617, 1562, 1384, and 621 cm^−1^; ^1^H and ^13^C NMR data, see Table 1 and Table 2; and positive-ion HR-ESI-MS *m*/*z* 369.1672 [M + Na]^+^ (calcd for C_20_H_26_NaO_5_^+^, 369.1672, Δ*m*/*z* theoretical = 0.1 ppm).

## 4. Conclusions

The chemical investigation of *J. ribifolia* led to the isolation and structural characterization of sixteen compounds, including eight new macrocyclic diterpenes, herein named ribifolones A–H. Regarding their biological activity, ribifolones A, C and D exhibited cytotoxic effects against melanoma (SK-MEL-28) and colorectal cancer (HCT-116) cell lines, although with distinct potency profiles. Among the tested compounds, ribifolone C and jatrophone displayed comparatively stronger activity. The complementary application of network pharmacology, molecular docking, and molecular dynamics simulations indicated that the effects of both compounds could be related to the modulation of the PI3K–AKT-mTOR signaling pathway, with estrogen receptor beta (ERβ) emerging as a potential target. Nevertheless, these findings require further experimental validation to confirm the proposed molecular interactions and downstream biological effects.

In summary, these findings highlight the chemical diversity of the *Jatropha* genus and reinforce the pharmacological relevance of *J. ribifolia*. The integration of in vitro and in silico strategies proved valuable for generating biologically grounded hypotheses, supporting the continued exploration of *Euphorbiaceae* species as sources of structurally distinctive bioactive metabolites.

## Figures and Tables

**Table 1:** ^1^H NMR data at 400 MHz, in CDCl_3_, for ribifolones A–H (1–8), δ_H_ in ppm, J in Hz.

	1	2	3	4	5	6	7	8
Position	δ_H_	δ_H_	δ_H_	δ_H_	δ_H_	δ_H_	δ_H_	δ_H_
1*α*	2.22 dd (14.4; 8.4)	2.43 m	1.67 m	2.6 d (12)	2.99 dt (1.6)	1.24 d (13.3)	2.11 m	1.9 m
1*β*	1.89 d (14.4)	2.11 d (4.4)	2.04 dd (14.3; 8.3)	2.2 d (12)	2.83 m	1.64–1.54 m	2.71 m	2.1 m
2	2.38 m	2.99 m	2.48 dd	-	-	2.12–2.06 m	2.12–2.08 m	2.32 m
3	3.34 s	6.15 d (2.0)	3.20 m	6.10 s	6.6 s	3.16 sl	4.27 d (4.0)	4.05 d (3,6)
4	-	-	-	-	-	-	-	
5	5.63 ql (1.3)	6.51 s	5.74 dd (3.22. 1.6)	6.11 bs	6.3 bs	5.24 ql (1.6)	5.99 s	5.7 sl
6	-	-	-	-	-	-	-	
7	-	-	-	-	-	-	-	
8	3.82 d (11.3)	3.31 d (1.6)	6.23 d (16.5)	6.0 d (13.2)	6.01 d (12.0)	2.56–2.50 m	2.78 d 2.81 d (1.6)	2.83 dd 2.75 dd
9	4.05 d (11.3)	4.81 t	6.41 d (16.5)	6.5 d (13.2)	6.54 d (12.0)	2.84 dd (10.0; 1.7)	2.59 d (2.0)	1.73 m
10	-	-	-	-	-	-	-	
11*α*	2.13 d (15.3)	2.30 dd (1.6; 1.6)	2.48 dd (0.8; 0.8)	2.7 d (12.0)	2.80 m	2.43 d (16.0)	2.49 d (10.8)	2.0 d (12)
11*β*	1.86 d (15.3)	2.60 d (13.6)	2.86 d (14.8)	2.4 d (12.0)	2.50 m	2.24 d (16.0)	2.23 d (12.8)	
12	-	-	-	-	-	-	-	-
13	-	-	-	-	-	-	-	-
14	-	-	-	-	-	-	-	-
15	-	-	-	-	-	3.41 dd (10.0; 7.5)	-	-
16	1.12 d (6.6)	1.06 d (5.6)	1.20 d (7.6)	1.65 s	5.10 bt (1.6) 5.25 bt (1.6)	1.05 d (7.5)	1.14 d (5.2)	1.1 sl
17	1.83 d (1.3)	1.98 d (1.5)	1.83 d (1.5)	1.95 d (1.3)	1.98 d (0.8)	1.90 d (1.6)	1.95 d (1.5)	1.30 s
18	1.11 s	0.94 s	1.22 s	1.21 s	1.23 s	1.21 s	1.28 s	1.41 s
19	1.13 s	1.22 s	1.41 s	1.33 s	1.36 s	0.84 s	0.88 s	1.39 s
20	1.16 s	1.52 s	1.70 d (0.7)	1.73 s	1.77 s	1.34 s	1.32 s	1.78 s

**Table 2:** ^13^C NMR data at 100 MHz, in CDCl_3_, for ribifolones A–H (1–8), δ_C_ in ppm.

	1	2	3	4	5	6	7	8
Position	δ_C_	δ_C_	δ_C_	δ_C_	δ_C_	δ_C_	δ_C_	δ_C_
1	44.4	46.0	37.6	41.5	37.1	33.6	41.6	44.1
2	31.0	37.0	33.2	139.0	148.9	34.9	43.0	36.4
3	75.6	148.9	69.2	136.2	135.9	65.8	86.9	83.1
4	64.0	140.8	68.4	93.9	142.1	66.8	143.0	138.2
5	131.4	127.3	122.9	136.3	137.2	129.1	125.2	132.2
6	143.5	137.7	142.3	143.9	142.2	139.1	143.6	77.0
7	202.7	199.8	201.3	202.3	201.9	211.2	209.1	208.5
8	66.5	66.9	129.7	128.7	128.3	38.8	38.9	33.6
9	79.9	80.8	158.3	158.6	157.7	49.2	51.1	31.4
10	37.2	37.8	36.7	40.9	40.8	37.6	37.4	29.4
11	43.2	48.9	41.7	43.3	43.2	55.2	55.4	27.6
12	77.6	91.1	185.3	185.6	185.3	214.7	214.7	189.0
13	89.4	88.0	112.2	113.4	113.1	64.0	64.2	109.5
14	212.4	206.8	204.4	204.4	203.2	211.5	211.3	203.4
15	87.0	91.0	94.8	97.7	98.5	45.6	139.7	91.1
16	16.5	20.4	17.2	24.2	109.6	14.9	18.2	14.3
17	22.1	20.7	21.4	13.3	13.1	21.2	19.2	29.4
18	27.6	25.3	30.8	30.4	30.3	27.6	27.3	28.8
19	22.3	28.0	27.2	26.5	26.3	23.6	23.6	15.4
20	14.0	17.3	6.1	7.3	7.2	14.2	14.5	5.8

**Table 3:** Cytotoxicity of ribifolones A–H in micromolar (µM), using the MTT reduction assay after 72 h of treatment. The data is presented as the half-maximal (50%) inhibitory concentration (IC_50_) values as the mean ± SEM (three independent experiments in triplicate), calculated by nonlinear regression analysis using concentration–response curves.

Compounds	IC_50_ (µM)		
	Cell Lines		
	SK-MEL-28	HCT-116	HEK-293
**ribifolone A**	32.25 ± 2.41	>100.0	>100.0
**ribifolone B**	>100.0	>100.0	-
**ribifolone C**	50.71 ± 3.80	33.39 ± 1.98	>100.0
**ribifolone** **D**	95.64 ± 1.51	75.35 ± 4.82	>100.0
**ribifolone E**	>100.0	>100.0	-
**ribifolone F**	>100.0	>100.0	-
**ribifolone G**	>100.0	>100.0	-
**ribifolone H**	>100.0	>100.0	-
**jatrophone**	6.19 ± 0.96	10.09 ± 0.61	20.28 ± 0.98

**Table 4:** Docking results for target proteins comparing ribifolone C, jatrophone, and reference ligands displaying Moldock score, ligand efficiency (LE), and RMSD values.

Protein	Ligand	Moldock Score	LE	RMSD (Å)
ERα	ribifolone C	−99.6025	4.15	
	jatrophone	−99.9278	4.34	
	compound **15**	−176.836	5.20	0.152
ERβ	ribifolone C	−103.407	4.31	
	jatrophone	−106.904	4.65	
	WAY-697	−113.646	3.67	0.137
HER2	ribifolone C	−109.498	4.56	
	jatrophone	−109.166	4.75	
	SYR127063	−211.971	6.23	0.206
HSP90α	ribifolone C	−93.2953	3.89	
	jatrophone	−92.7489	4.03	
	PU3	−133.74	4.46	0.226
PI3Kγ	ribifolone C	−131.08	2.49	
	jatrophone	−133.74	2.36	
	PF-04979064	−133.74	3.86	0.337

**Table 5:** Interaction energy, RMSD, and total drift values obtained from molecular dynamics simulations for ERβ complexes with ribifolone C, jatrophone, and WAY-697.

Molecule	Energy	Average	RMSD (Å)	Total−Drift
Ribifolone C	Coulombic	−68.839	30.630	−50.900
	Lennard–Jones	−77.920	16.805	−19.054
Jatrophone	Coulombic	−25.577	19.671	18.438
	Lennard–Jones	−78.050	18.327	9.609
WAY-697	Coulombic	−158.979	39.815	−58.408
	Lennard–Jones	−51.151	20.575	−43.673

**Table 6:** Crystallographic structures of the selected molecular targets retrieved from the Protein Data Bank (PDB), including their respective PDB identification codes.

Targets	PDB (ID)	Resolution	Ligands	Reference
Erα *	1XP1	1.80 Å	Compound **15**	[40]
Erβ **	1X76	2.20 Å	WAY-697	[41]
HER2 ***	3PP0	2.25 Å	SYR127063	[42]
HSP90α ****	1UYF	2.00 Å	PU3	[43]
PI3Kγ *****	4HVB	2.35 Å	PF-04979064	[44]

* Human estrogen receptor alpha (ERα); ** human estrogen receptor beta (ERβ); *** human epidermal growth factor receptor 2; **** heat shock protein-90alpha; ***** phosphoinositide 3-kinase-γ.

## Data Availability

All the information required was made available as a Supplementary Materials.

## Supplementary Materials

The following supporting information can be downloaded at: https://www.mdpi.com/article/10.3390/molecules31101663/s1.
