# Supplementary material for: Ribifolones A–H, New Macrocyclic Diterpenes from Jatropha ribifolia, Their Cytotoxic Activity and Insights Supported by Network Pharmacology and Molecular Modeling
Source: Molecules. 2026 May 14;31(10):1663. doi: 10.3390/molecules31101663 (PMC13209920; doi:10.3390/molecules31101663)
Supplement: Supplementary file 1 [file molecules-31-01663-s001.zip › molecules-4204495-supplementary.pdf]

Supplementary Information

Ribifolones A–H, New Macrocyclic Diterpenes from *Jatropha ribifolia*, their Cytotoxic Activity and Insights Supported by Network Pharmacology and Molecular Modeling

Thalisson Amorim de Souza[1], Alan Ferreira Alves[1], Ramon Ramos Marques de Souza[1], Ana Carolina Ferreira de Albuquerque[2], Thiago Araújo de Medeiros Brito[1], Marianna Vieira Sobral[1], Fernando Martins dos Santos Júnior[2], Maria de Fátima Agra[3], Luciana Sotti[1], Marcus Tullius Scotti[1], Lucas Silva Abreu[2], Josean Fechine Tavares[1*], Marcelo Sobral da Silva[1].

[1]Federal University of Paraiba, Graduate Program on Natural and Synthetic Bioactive Products (PgPNSB), Health Sciences Center, João Pessoa-PB, Brazil.

[2]Federal University of Paraiba, Center of Biotechnology, João Pessoa-PB, Brazil.

[3]Fluminense Federal University, Department of Organic Chemistry, Niteroi-RJ, Brazil. Federal University of Rio de Janeiro,

* Corresponding authors: Marcus Tullius Scotti; Josean Fechine Tavares

E-mails: mtscotti@gmail.com, josean@ltf.ufpb.br; abreu_lucas@id.uff.br

Table of Contents

Figure S1. HR-ESI-MS spectrum of Ribifolone A. 8

Figure S2. ^{1}H spectrum of Ribifolone A, 400MHz in $CDCl_3$. 8

Figure S3. Expansion of ^{1}H spectrum of Ribifolone A.. 9

Figure S4. Expansion of ^{1}H spectrum of Ribifolone A in $CDCl_3$. 9

Figure S5. Expansion of ^{1}H spectrum of Ribifolone A, in $CDCl_3$. 10

Figure S6. ^{13}C spectrum of Ribifolone A, 100MHz in $CDCl_3$. 10

Figure S7. DEPT135 135 spectrum of Ribifolone A, 100MHz in $CDCl_3$. 11

Figure S8. COSY spectrum of Ribifolone A, 400MHz in $CDCl_3$. 11

Figure S9. HMBC contour map of Ribifolone A, 100MHz X 400MHz in $CDCl_3$......... 12

Figure S10. HSQC contour map of Ribifolone A, 100MHz X 400MHz in $CDCl_3$........ 12

Figure S11. NOESY spectrum of Ribifolone A, 400MHz in $CDCl_3$. 13

Figure S12. NOESY spectrum expansion of Ribifolone A, 400MHz in $CDCl_3$............. 13

Figure S13. Infrared spectrum of Ribifolone A. 14

Figure S14. Ultraviolet (UV) spectrum of Ribifolone A. 14

Figure S16. ^{1}H spectrum of Ribifolone B, 400MHz in $CDCl_3$. 15

Figure S17. Expansion ^{1}H spectrum of Ribifolone B in $CDCl_3$. 16

Figure S18. Expansion ^{1}H spectrum of Ribifolone B in $CDCl_3$. 16

Figure S19. Expansion ^{1}H spectrum of Ribifolone B in $CDCl_3$ 17

Figure S20. ^{13}C spectrum of Ribifolone B, 100MHz in $CDCl_3$...................................... 17

Figure S21. DEPT135135 spectrum of Ribifolone B, 100MHz in $CDCl_3$. 18

Figure S22. COSY spectrum of Ribifolone B, 400MHz in $CDCl_3$................................. 18

Figure S23. HMBC contour map of Ribifolone B, 100MHz X 400MHz in $CDCl_3$....... 19

Figure S24. Expansion of HMBC contour map of Ribifolone B in $CDCl_3$.................... 19

Figure S25. HSQC contour map of Ribifolone B, 100MHz X 400MHz in $CDCl_3$......... 20

Figure S26. NOESY spectrum of Ribifolone B, 400MHz in $CDCl_3$. 20

Figure S27. Infrared spectrum of Ribifolone B. 21

Figure S28. Ultraviolet (UV) spectrum of Ribifolone B. 21

Figure S29. HR-ESI-MS spectrum of Ribifolone C 22

Figure S30. ^1H spectrum of Ribifolone C, 400MHz in $CDCl_3$ 23

Figure S31. Expansion of ^1H spectrum of Ribifolone C, in $CDCl_3$ (1.0-4.1 ppm). 23

Figure S32. Expansion of ^1H spectrum of Ribifolone C in $CDCl_3$. 24

Figure S33. ^{13}C spectrum of Ribifolone C, 100MHz in $CDCl_3$ 24

Figure S34. DEPT135 spectrum of Ribifolone C, 100 MHz in $CDCl_3$ 25

Figure S35. COSY spectrum of Ribifolone C, 400MHz in $CDCl_3$ 25

Figure S36. HMBC contour map of Ribifolone C, 100MHz X 400MHz in $CDCl_3$. 26

Figure S37. Expansion of HMBC contour map of Ribifolone C. 26

Figure S38. Expansion of HMBC contour map of Ribifolone C. 27

Figure S39. HSQC contour map of Ribifolone C, 100MHz X 400MHz in $CDCl_3$. 27

Figure S40. NOESY contour map of Ribifolone C 400MHz in $CDCl_3$ 28

Figure S41. Infrared spectrum of Ribifolone C. 28

Figure S42. Ultraviolet (UV) spectrum of Ribifolone C. 29

Figure S44. ^1H spectrum of Ribifolone D, 400MHz in $CDCl_3$. 30

Figure S45. Expansion ^1H spectrum of Ribifolone D in $CDCl_3$. 30

Figure S46. ^{13}C spectrum of Ribifolone D, 100MHz in $CDCl_3$. 31

Figure S47. DEPT135 spectrum of Ribifolone D, 100MHz in $CDCl_3$. 31

Figure S48. COSY spectrum of Ribifolone D, 400MHz in $CDCl_3$. 32

Figure S49. HMBC contour map of Ribifolone D, 100MHz X 400MHz in $CDCl_3$ 32

Figure S50. Expansion of HMBC contour map of Ribifolone D. 33

Figure S51. HSQC contour map of Ribifolone D, 100MHz X 400MHz in $CDCl_3$ 33

Figure S52. NOESY spectrum of Ribifolone D, 400MHz in $CDCl_3$. 34

Figure S53. Infrared spectrum of Ribifolone D. 34

Figure S54. Ultraviolet (UV) spectrum of Ribifolone D. 35

Figure S55. HR-ESI-MS spectrum of Ribifolone E. 35

Figure S56. ^{1}H spectrum of Ribifolone E, 400MHz in $CDCl_3$. 36

Figure S57. Expansion of ^{1}H spectrum of Ribifolone E in $CDCl_3$. 36

Figure S58. Expansion of ^{1}H spectrum of Ribifolone E in $CDCl_3$. 36

Figure S59. Expansion of ^{1}H spectrum of Ribifolone E in $CDCl_3$ 37

Figure S60. ^{13}C spectrum of Ribifolone E, 100MHz in $CDCl_3$ 38

Figure S61. DEPT135 spectrum of Ribifolone E, 100MHz in $CDCl_3$. 38

Figure S62. COSY spectrum of Ribifolone E, 400MHz in $CDCl_3$ 39

Figure S63. HMBC spectrum of Ribifolone E, 100MHz X 400MHz in $CDCl_3$. 39

Figure S64. HSQC spectrum of Ribifolone E 100MHz X 400MHz in $CDCl_3$. 40

Figure S65. NOESY spectrum of Ribifolone E, 400MHz in $CDCl_3$. 40

Figure S66. Infrared spectrum of Ribifolone E. 41

Figure S67. Ultraviolet (UV) spectrum of Ribifolone E. 41

Figure S68. HR-ESI-MS spectrum of Ribifolone F. 42

Figure S69. ^{1}H spectrum of Ribifolone F, 400MHz in $CDCl_3$. 42

Figure S70. Expansion of ^{1}H spectrum of Ribifolone F in $CDCl_3$ 43

Figure S71. ^{13}C spectrum of Ribifolone F, 100MHz in $CDCl_3$ 43

Figure S72. DEPT135 spectrum of Ribifolone F, 100MHz in $CDCl_3$ 44

Figure S73. COSY spectrum of Ribifolone F, 400MHz in $CDCl_3$ 44

Figure S74. HMBC contour map of Ribifolone F, 400MHz in $CDCl_3$ 45

Figure S75. HSQC contour map of Ribifolone F, 400MHz in $CDCl_3$ 45

Figure S76. NOESY spectrum of Ribifolone F, 400MHz in $CDCl_3$ 46

Figure S77. Infrared spectrum of Ribifolone F. 46

Figure S78. Ultraviolet (UV) spectrum of Ribifolone F. 47

Figure S79. HR-ESI-MS spectrum of Ribifolone G. 47

Figure S80. ^{1}H spectrum of Ribifolone G, 400MHz in $CDCl_3$ 48

Figure S81. Expansion of ^{1}H spectrum of Ribifolone G in $CDCl_3$ 48

Figure S82. Expansion of ^{1}H spectrum of Ribifolone G in $CDCl_3$ 49

Figure S83. Expansion of ^{1}H spectrum of Ribifolone G in $CDCl_3$ 49

Figure S84. ^{13}C spectrum of Ribifolone G, 100MHz in $CDCl_3$. 50

Figure S85. DEPT135 spectrum of Ribifolone G, 100MHz in $CDCl_3$ 50

Figure S86. COSY spectrum of Ribifolone G, 400MHz in $CDCl_3$. 51

Figure S87. HMBC contour map of Ribifolone G, 100MHz X 400MHz in $CDCl_3$........ 51

Figure S88 HMBC contour map expansion of Ribifolone G, 100 MHz x 400MHz in $CDCl_3$. 52

Figure S89. HSQC contour map of Ribifolone G, 100MHz X 400MHz in $CDCl_3$. 52

Figure S90. NOESY spectrum of Ribifolone G, 400MHz in $CDCl_3$. 53

Figure S91. Infrared spectrum of Ribifolone G. 53

Figure S92. Ultraviolet (UV) spectrum of Ribifolone G. 54

Figure S93. HR-ESI-MS spectrum of Ribifolone H. 54

Figure S94. ^{1}H spectrum of Ribifolone H, 400MHz in $CDCl_3$. 55

Figure S95. ^{1}H spectrum of Ribifolone H in $CDCl_3$. 55

Figure S96. ^{1}H spectrum of Ribifolone H in $CDCl_3$. 56

Figure S97. ^{1}H spectrum of Ribifolone H in $CDCl_3$. 56

Figure S98. ^{13}C spectrum of Ribifolone H, 100 MHz in $CDCl_3$. 57

Figure S99. DEPT135135 spectrum of Ribifolone H, 100 MHz in $CDCl_3$. 57

Figure S100. COSY spectrum of Ribifolone H, 400MHz in $CDCl_3$ 58

Figure S101. HMBC contour map of Ribifolone H, 100MHz x 400MHz in $CDCl_3$. 58

Figure S102. HMBC contour map expansion of Ribifolone H, in $CDCl_3$. 59

Figure S103. HMBC contour map expansion of Ribifolone H, in $CDCl_3$. 59

Figure S104. HSQC contour map of Ribifolone H in $CDCl_3$. 60

Figure S105. NOESY spectrum of Ribifolone H, 500MHz in $CDCl_3$............................ 60

Figure S106. Infrared spectrum of Ribifolone H. 61

Figure S107. Ultraviolet (UV) spectrum of Ribifolone H. 61

Figure S108. HR-ESI-MS spectrum of 9β,13α-dehydroxyisabellione.. 62

Figure S109. ^{1}H spectrum of 9β,13α-dehydroxyisabellione, 400MHz in $CDCl_3$.. 62

Figure S110. ^{13}C spectrum of 9β,13α-dehydroxyisabellione, 100MHz in $CDCl_3$.. 63

Figure S111. DEPT135 spectrum of 9β,13α-dehydroxyisabellione, 100 MHz in $CDCl_3$. 63

Figure S112. HR-ESI-MS spectrum of Jatrophone.. 64

Figure S113. ^{1}H spectrum of Jatrophone, 400MHz in $CDCl_3$. 64

Figure S114. ^{13}C spectrum of Jatrophone, 100MHz in $CDCl_3$. 65

Figure S115. HR-ESI-MS spectrum of 2α-jatrophone, 400MHz in $CDCl_3$.. 65

Figure S116. ^{1}H spectrum of 2α-jatrophone, 400MHz in $CDCl_3$. 66

Figure S117. ^{13}C spectrum of 2α-jatrophone, 100MHz in $CDCl_3$. 66

Figure S118. HR-ESI-MS spectrum of 2β-jatrophone, 400MHz in $CDCl_3$.. 67

Figure S119. ^{1}H spectrum of 2β-jatrophone, 400MHz in $CDCl_3$. 67

Figure S120. HR-ESI-MS spectrum of Citlalitrione, 400MHz in $CDCl_3$........................ 68

Figure S121. ^{1}H spectrum of Citlalitrione, 400MHz in $CDCl_3$. 68

Figure S122. ^{13}C spectrum of Citlalitrione, 100MHz in $CDCl_3$. 69

Figure S123. ^{1}H spectrum of 6-Hydroxycyperene, 400MHz in $CDCl_3$.......................... 69

Figure S124. ^{13}C spectrum of 6-Hydroxycyperene, 100MHz in $CDCl_3$......................... 70

Figure S125. DEPT135 spectrum of 6-Hydroxycyperene, 100MHz in $CDCl_3$. 70

Figure S126. ^{1}H spectrum of sugeonol, 400MHz in $CDCl_3$. 71

Figure S127. ^{13}C spectrum of sugeonol, 100MHz in $CDCl_3$. 71

Figure S128. DEPT135 spectrum of sugeonol, 100MHz in $CDCl_3$. 72

Figure S129. ^{1}H spectrum of Patchoulenone, 400MHz in $CDCl_3$. 72

Figure S130. ^{13}C spectrum of Patchoulenone, 400MHz in $CDCl_3$. 73

Figure S131. HMBC contour map of patchoulenone, 100MHz x 400MHz in $CDCl_3$. ... 73

Molecular Docking 74

Figure S132. Three-dimensional representation of ribifolone C and jatrophone complexed with the selected molecular targets involved in the PI3K-AKT-mTor pathway, superimposed with their respective co-crystalized ligands. 74

Figure S133. Amino acids interactions formed between HER2, HSP90α, and PI3Kγ. and ribifolone C, jatrophone and the reference ligands. Amino acid interactions between ribifolone C (A), jatrophone (B), and SYR127063 against HER2. D/E/F. Amino acid interactions between ribifolone C (D), jatrophone (E), and PU3 (F) against HSPα. G/H/ I. Amino acid interactions between ribifolone C (G), jatrophone (H), and PF-04979064 (I) against PI3Kγ 76

Figure S1. HR-ESI-MS spectrum of Ribifolone A.

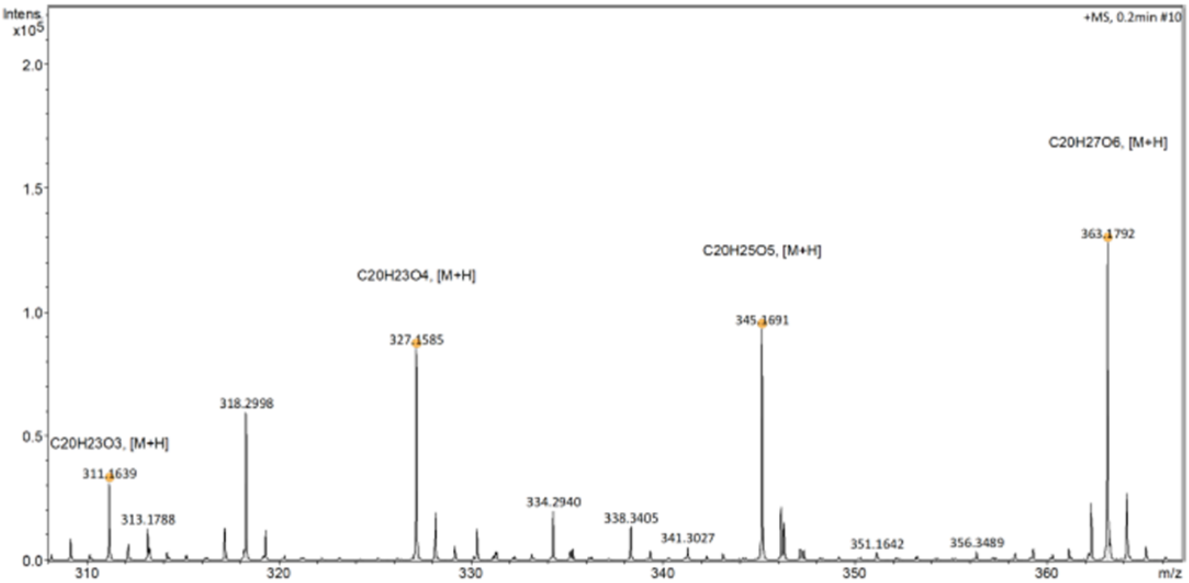

Meas. m/z	#	Ion Formula	m/z	err [ppm]	mSigma	# mSigma	Score	rdb	e¯ Conf	N-Rule
327.1585	1	C20H23O4	327.1591	1.7	20.5	1	100.00	9.5	even	ok
345.1690	1	C20H25O5	345.1697	2.0	8.0	1	100.00	8.5	even	ok
363.1792	1	C20H27O6	363.1802	2.9	8.8	1	100.00	7.5	even	ok

Figure S2. ^{1}H spectrum of Ribifolone A, 400MHz in $CDCl_3$.

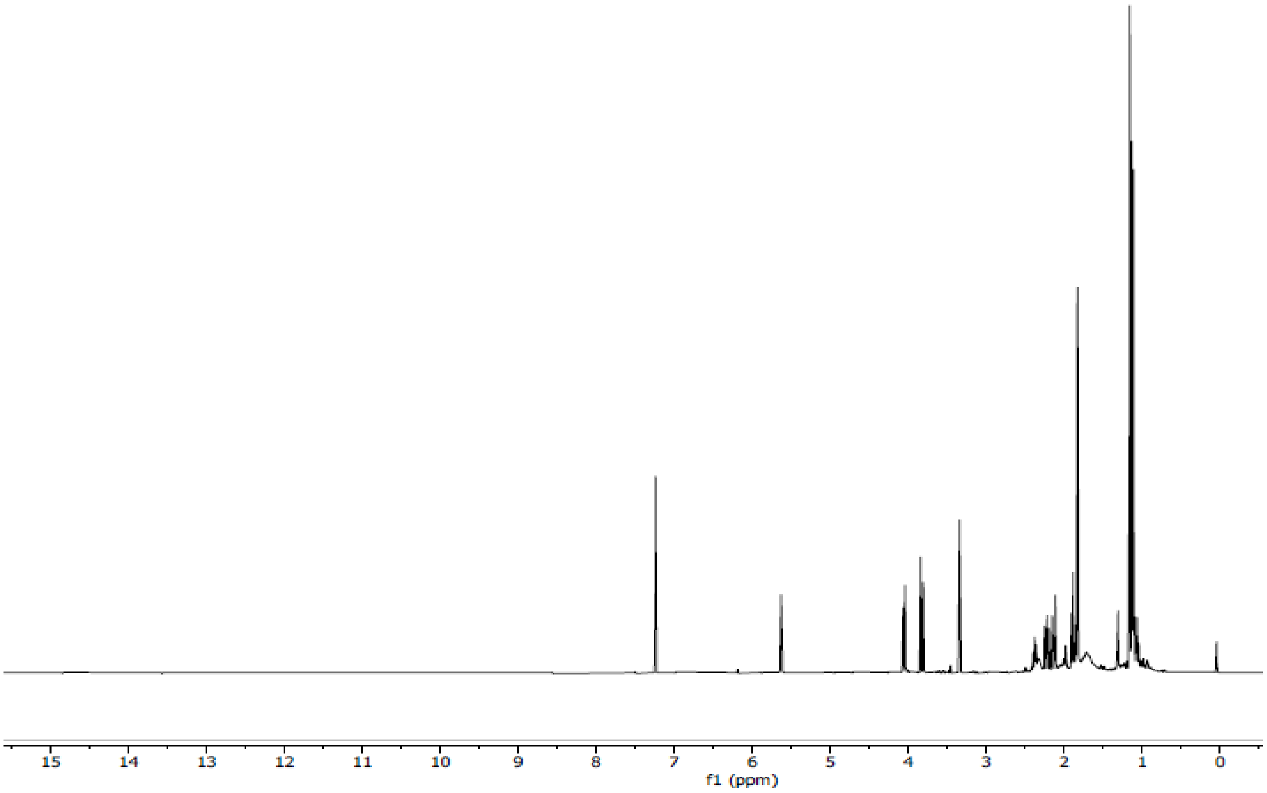

Figure S3. Expansion of ^{1}H spectrum of Ribifolone A.

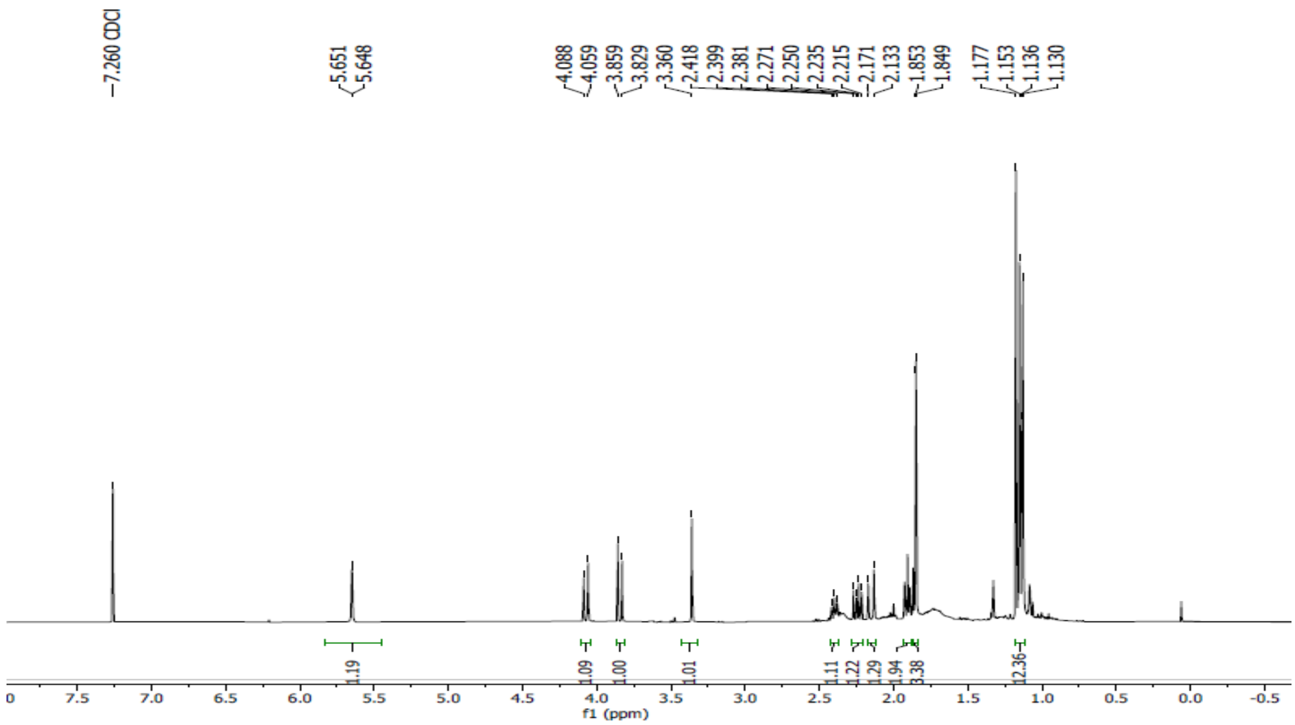

Figure S4. Expansion of ^{1}H spectrum of Ribifolone A in $CDCl_3$.

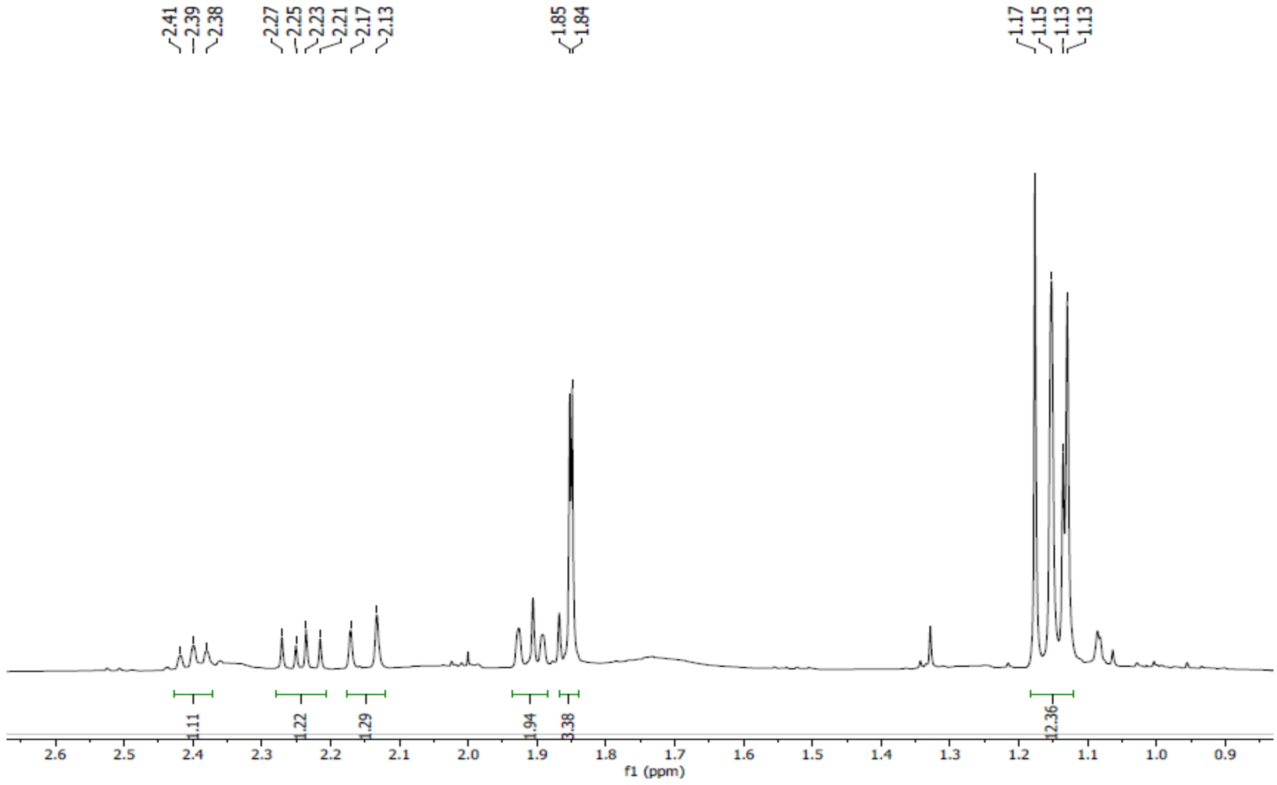

Figure S5. Expansion of ^{1}H spectrum of Ribifolone A, in $CDCl_3$.

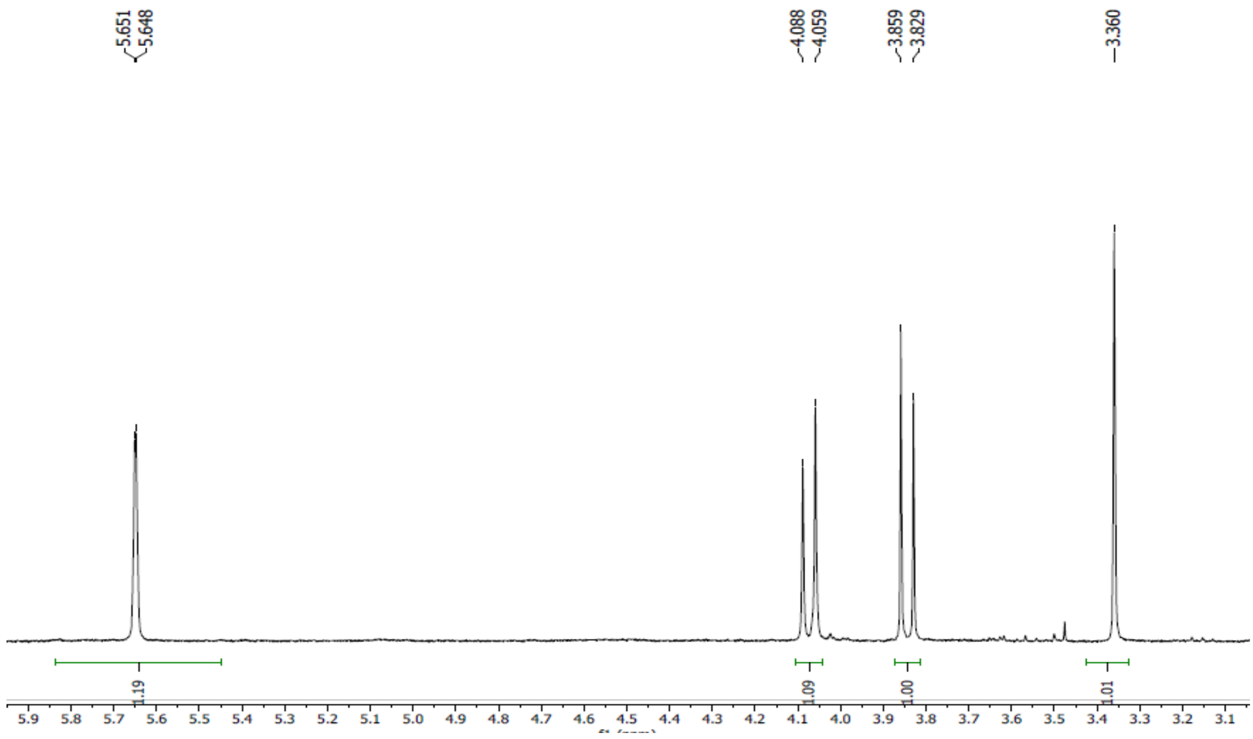

Figure S6. ^{13}C spectrum of Ribifolone A, 100MHz in $CDCl_3$.

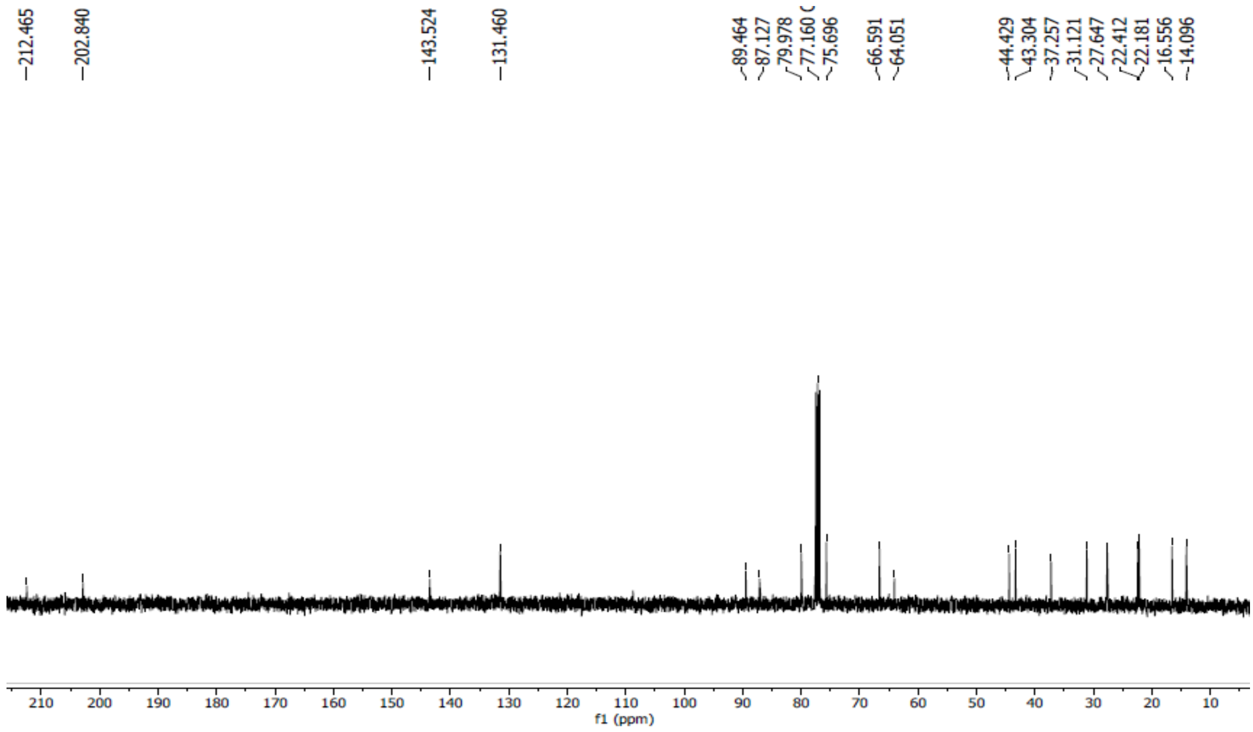

Figure S7. DEPT135 135 spectrum of Ribifolone A, 100MHz in CDCl3.

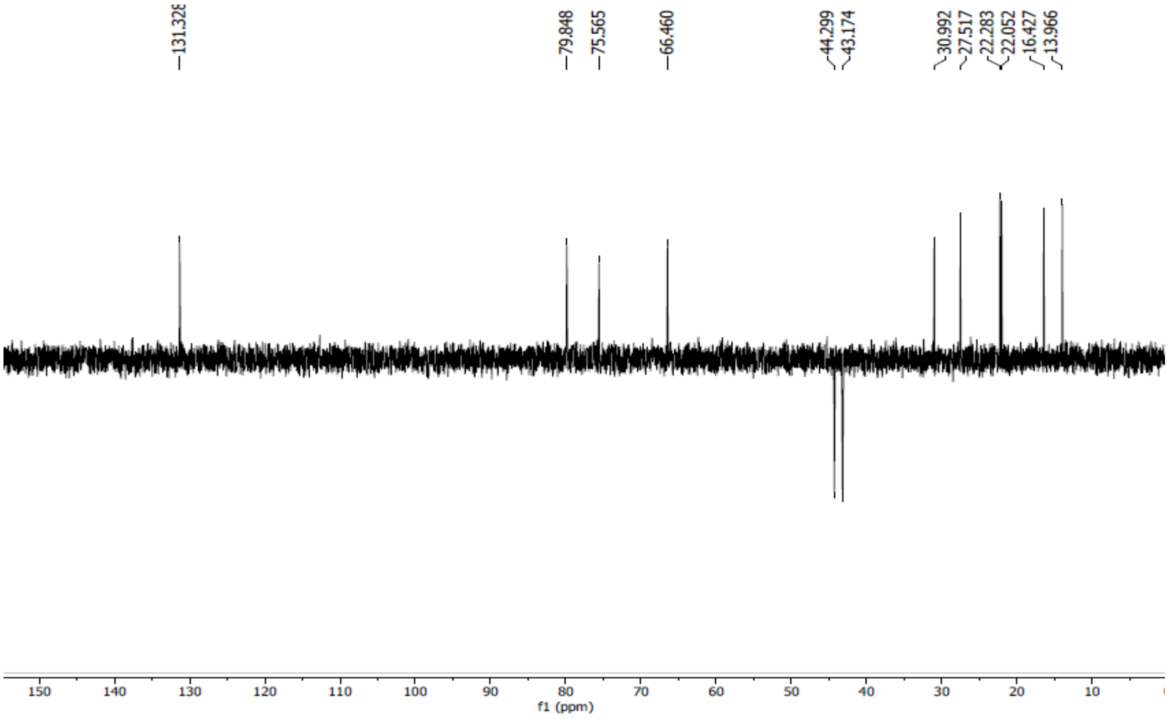

Figure S8. COSY spectrum of Ribifolone A, 400MHz in CDCl3.

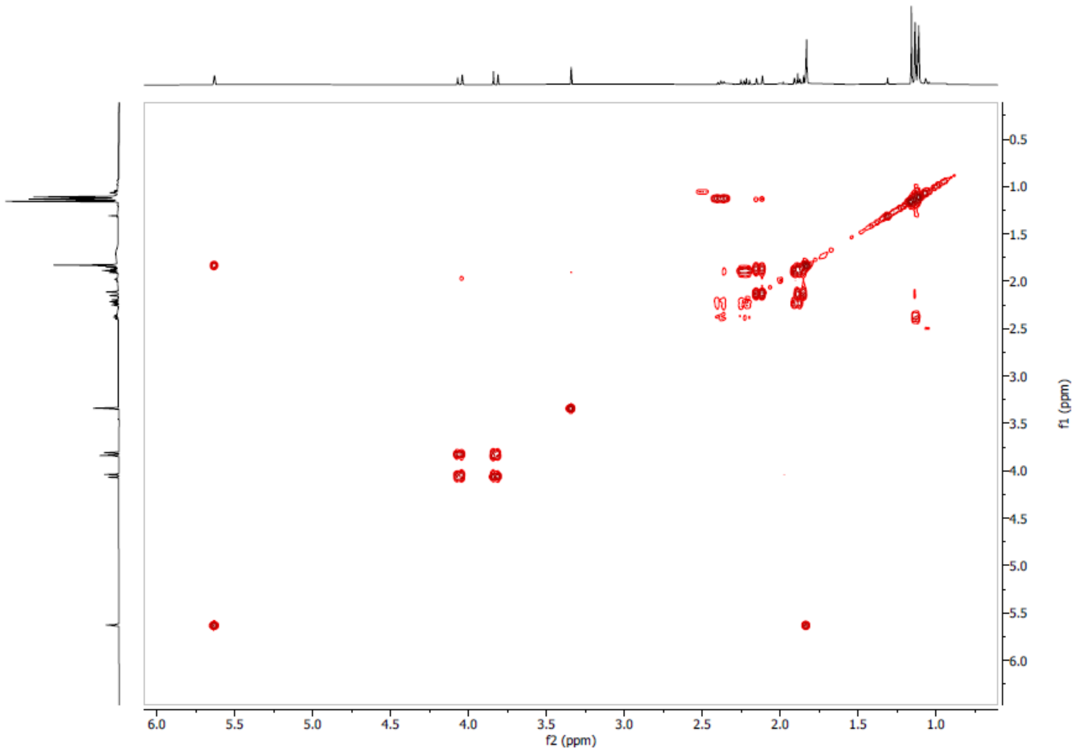

Figure S9. HMBC contour map of Ribifolone A, 100MHz X 400MHz in $CDCl_3$.

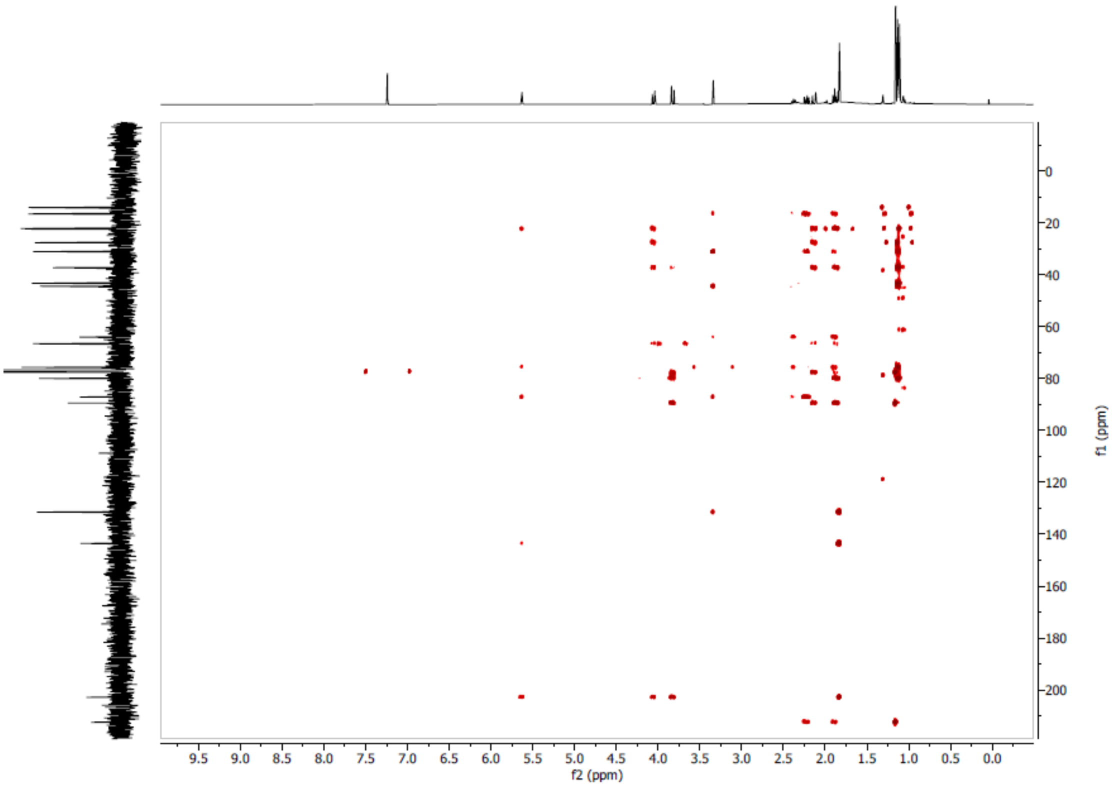

Figure S10. HSQC contour map of Ribifolone A, 100MHz X 400MHz in $CDCl_3$.

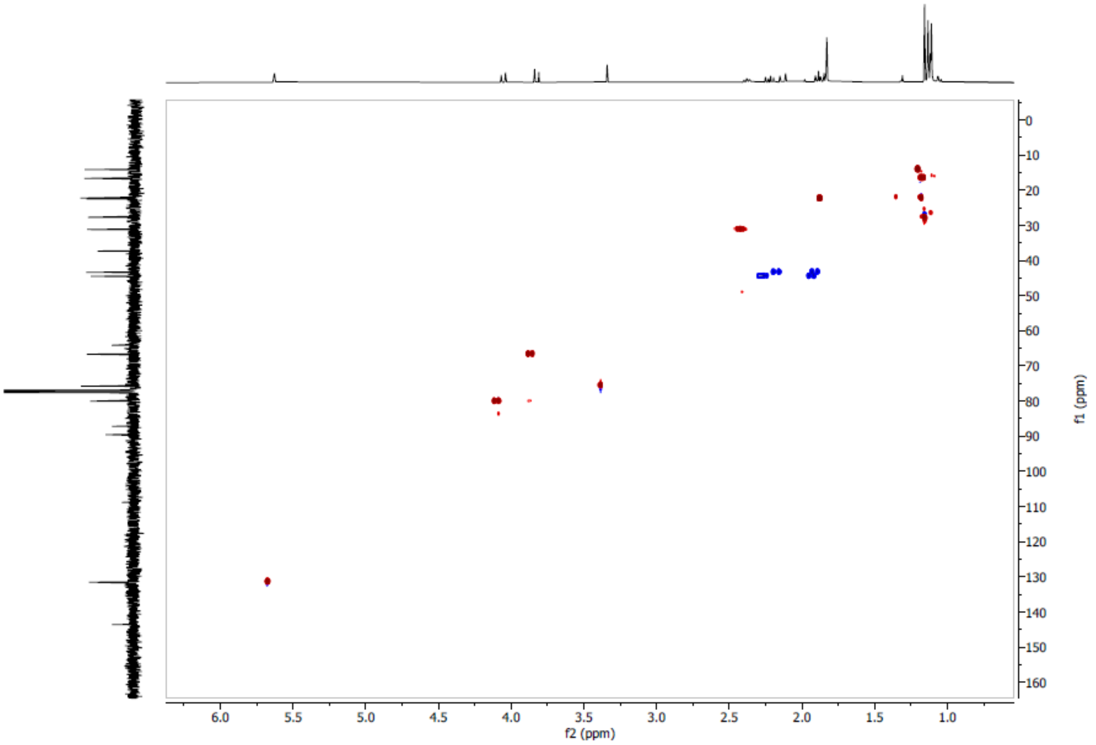

Figure S11. NOESY spectrum of Ribifolone A, 400MHz in $CDCl_3$.

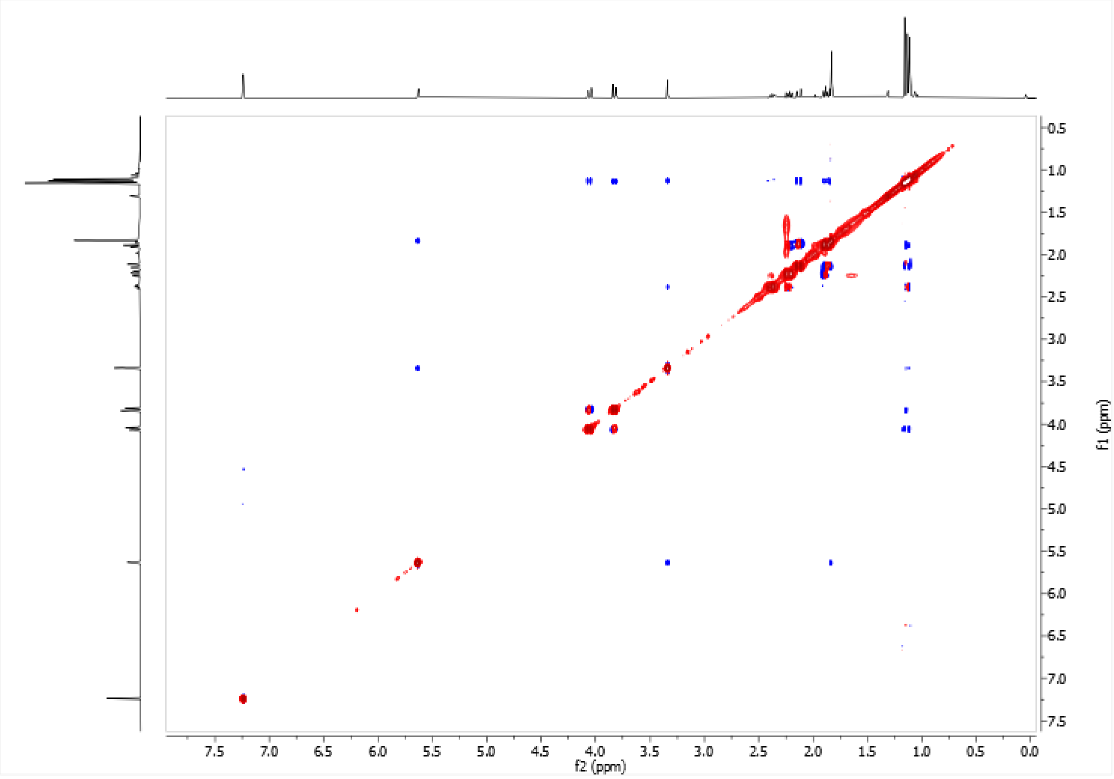

Figure S12. NOESY spectrum expansion of Ribifolone A, 400MHz in $CDCl_3$.

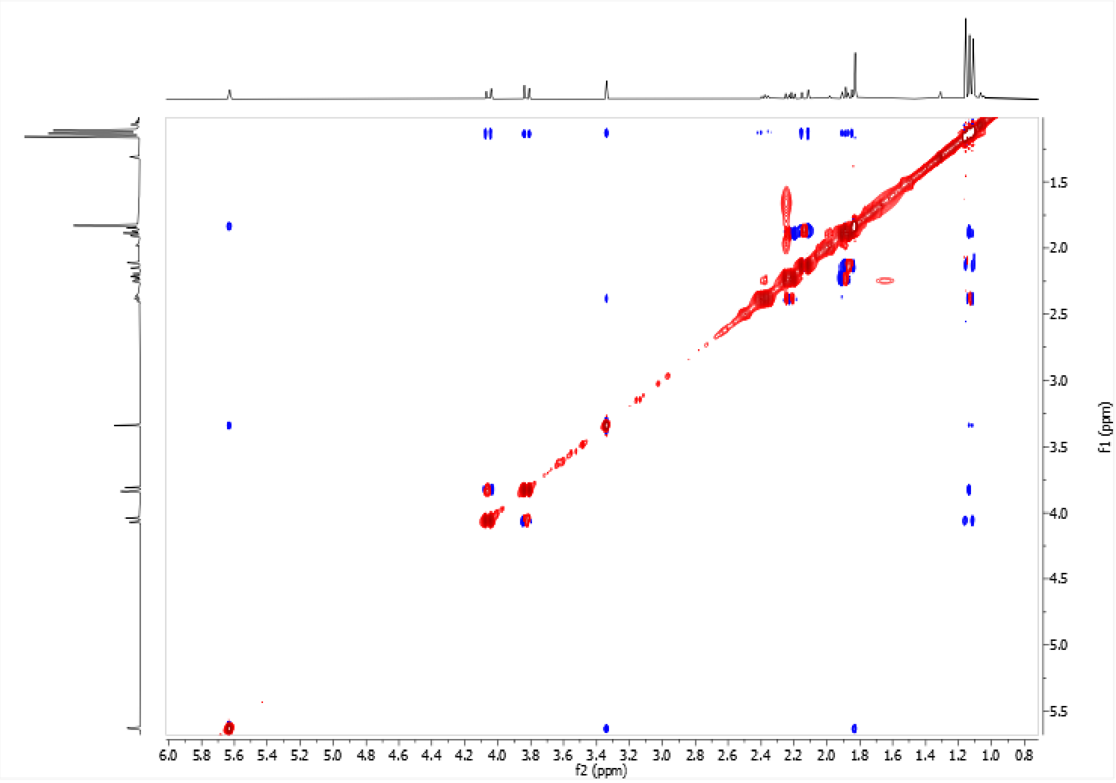

Figure S13. Infrared spectrum of Ribifolone A.

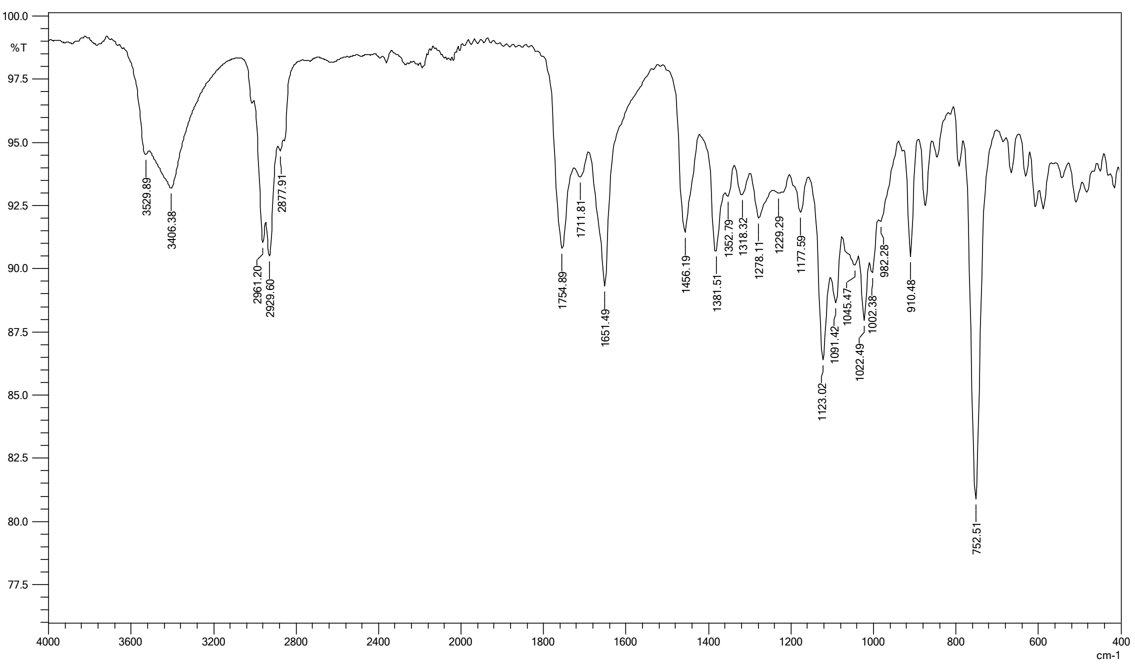

Figure S14. Ultraviolet (UV) spectrum of Ribifolone A.

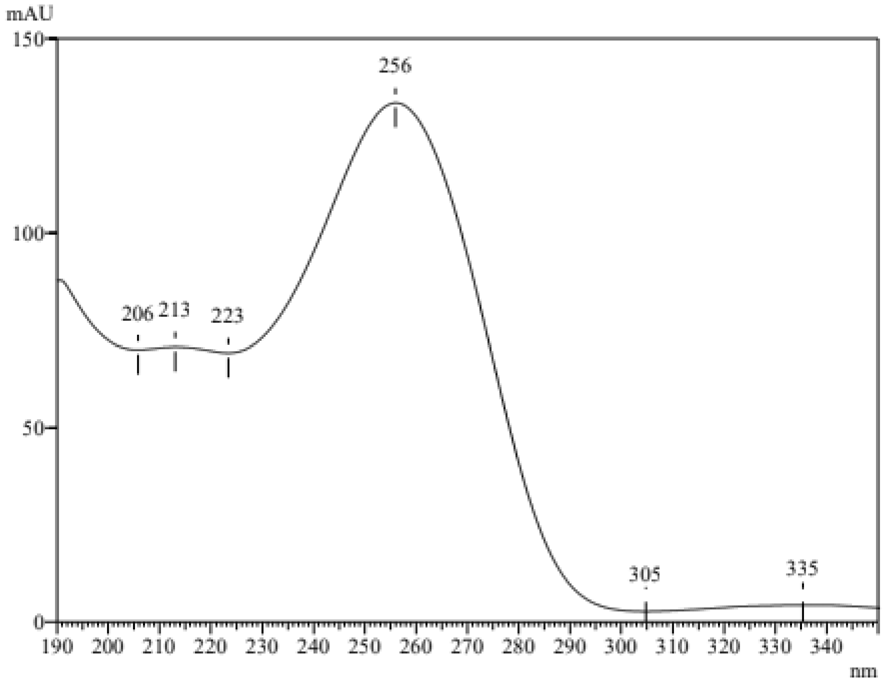

Figure S15. HR-ESI-MS spectrum of Ribifolone B.

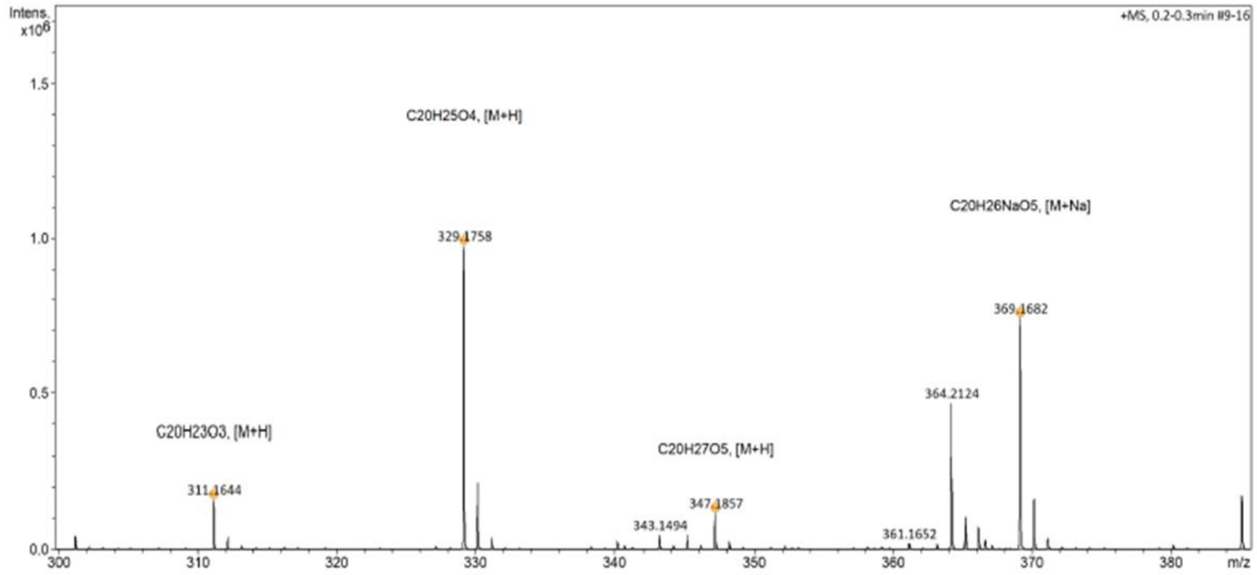

Meas. m/z	#	Ion Formula	m/z	err [ppm]	mSigma	# mSigma	Score	rdb	e⁻ Conf	N-Rule
311.1644	1	C20H23O3	311.1642	-0.7	27.1	1	100.00	9.5	even	ok
329.1758	1	C20H25O4	329.1747	-3.2	4.1	1	82.61	8.5	even	ok
347.1857	1	C20H27O5	347.1853	-1.3	16.9	1	100.00	7.5	even	ok
369.1682	1	C20H26NaO5	369.1672	-2.5	19.4	1	83.77	7.5	even	ok

Figure S16. ^{1}H spectrum of Ribifolone B, 400MHz in $CDCl_3$.

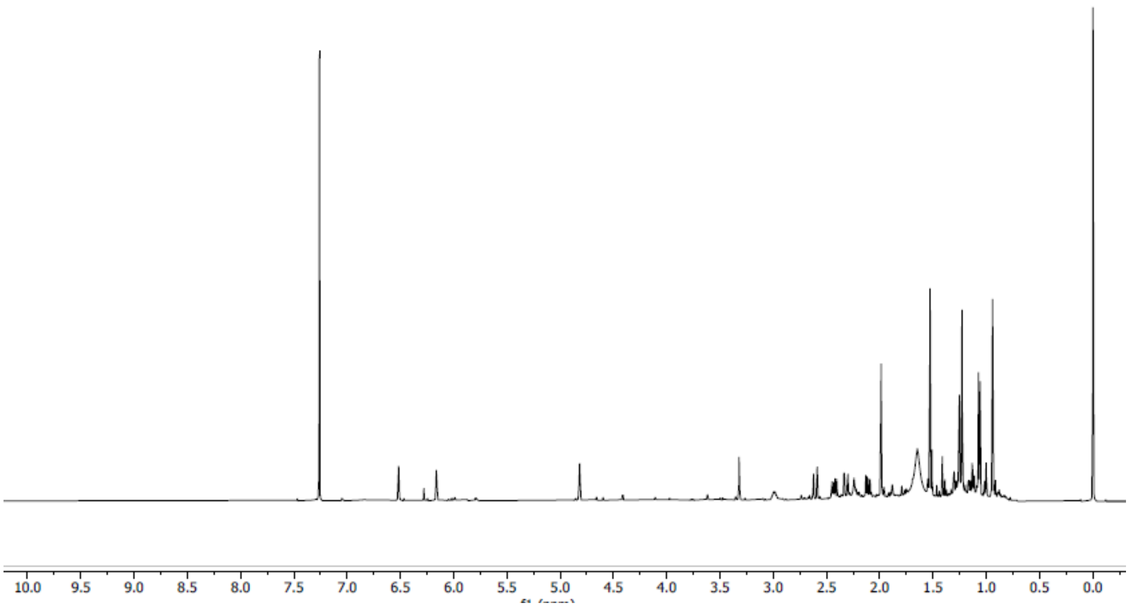

Figure S17. Expansion ^{1}H spectrum of Ribifolone B in $CDCl_3$.

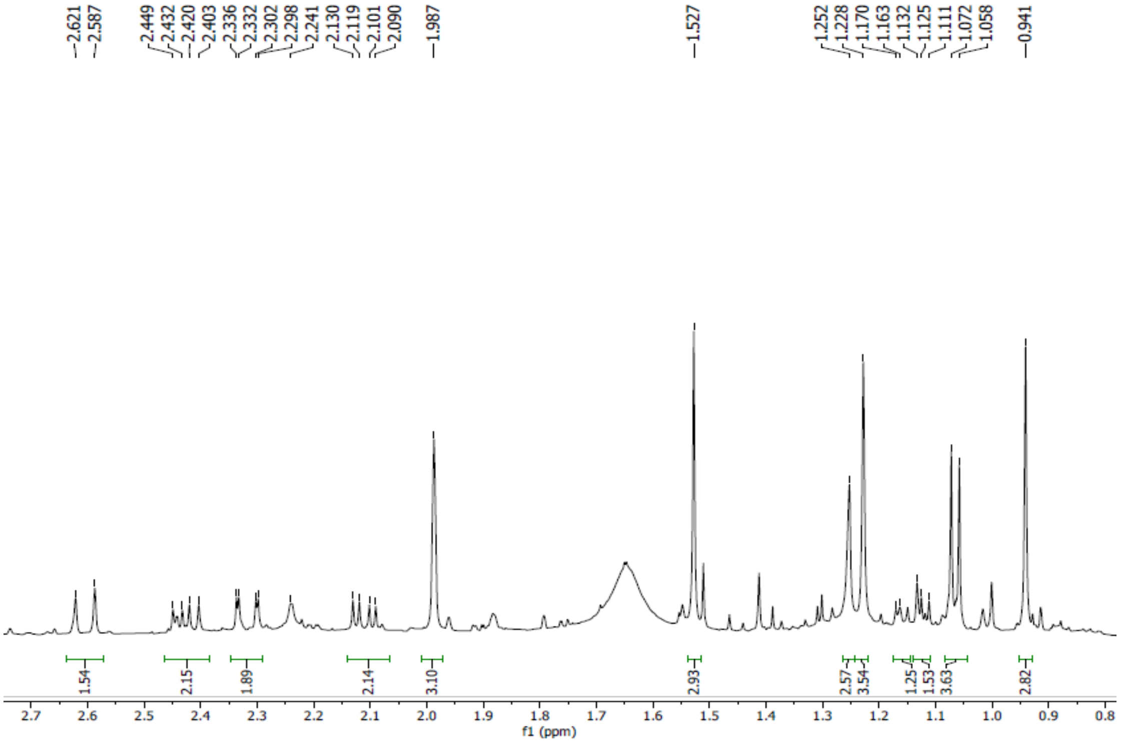

Figure S18. Expansion ^{1}H spectrum of Ribifolone B in $CDCl_3$.

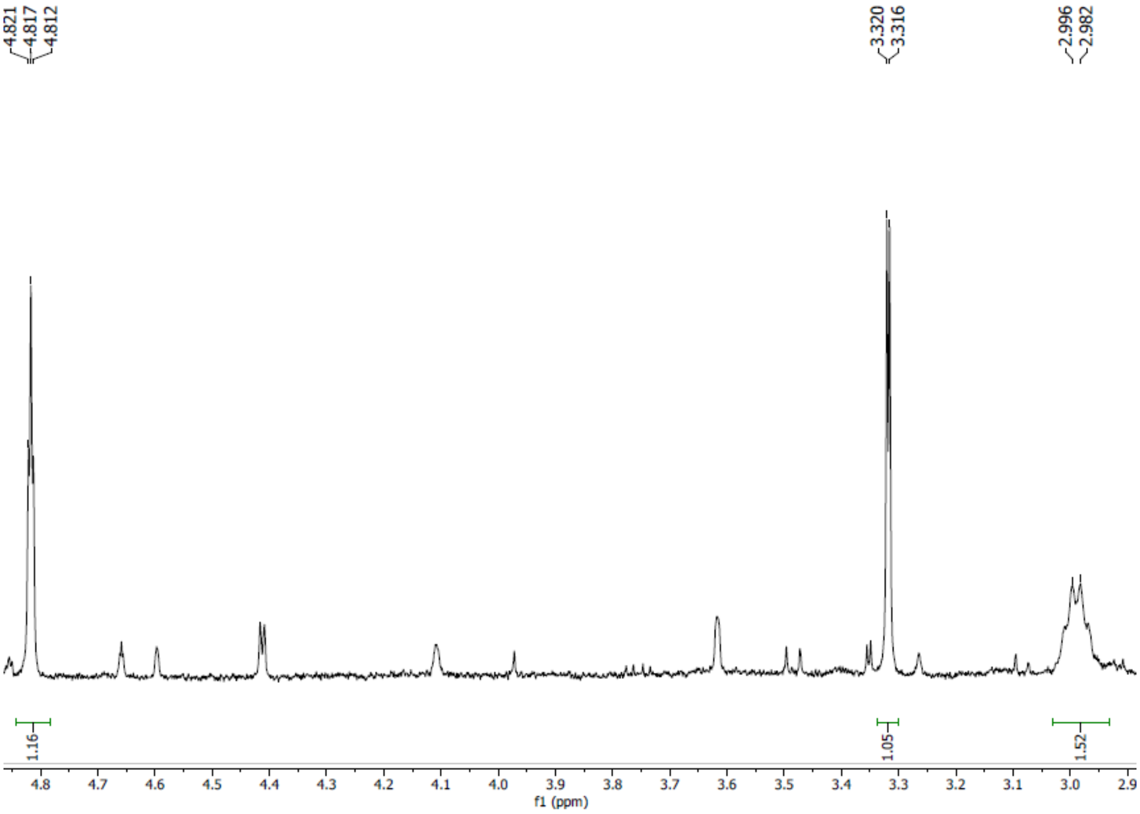

Figure S19. Expansion ^{1}H spectrum of Ribifolone B in $CDCl_3$.

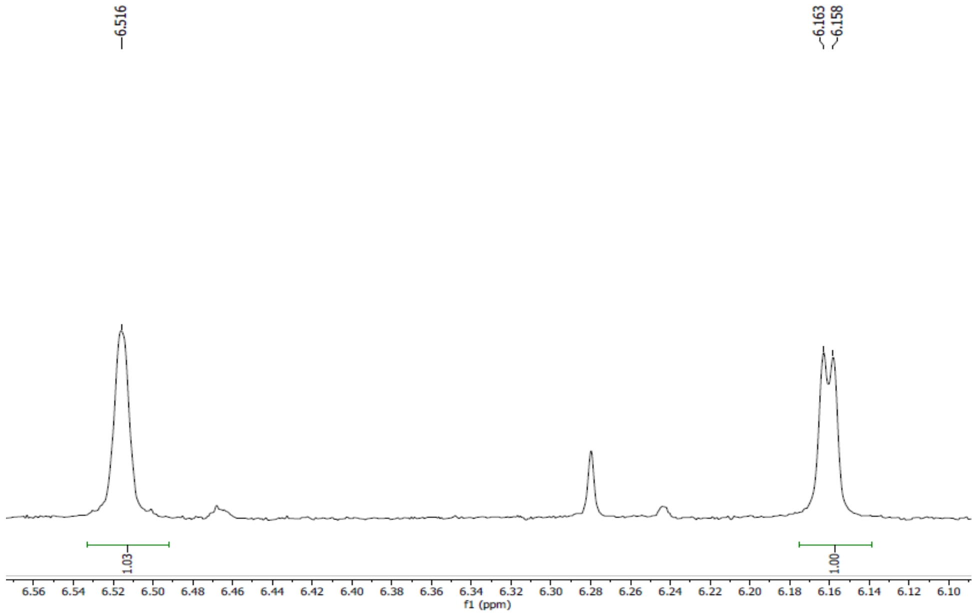

Figure S20. ^{13}C spectrum of Ribifolone B, 100MHz in $CDCl_3$.

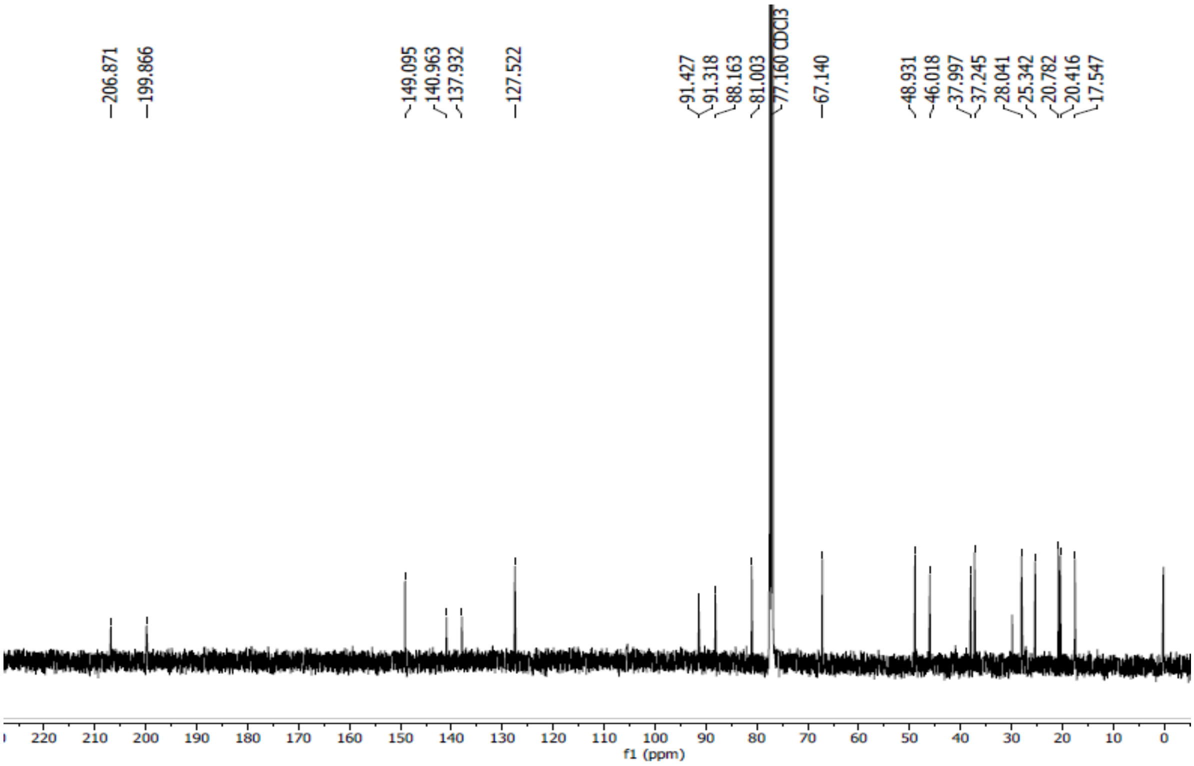

Figure S21. DEPT135135 spectrum of Ribifolone B, 100MHz in $CDCl_3$.

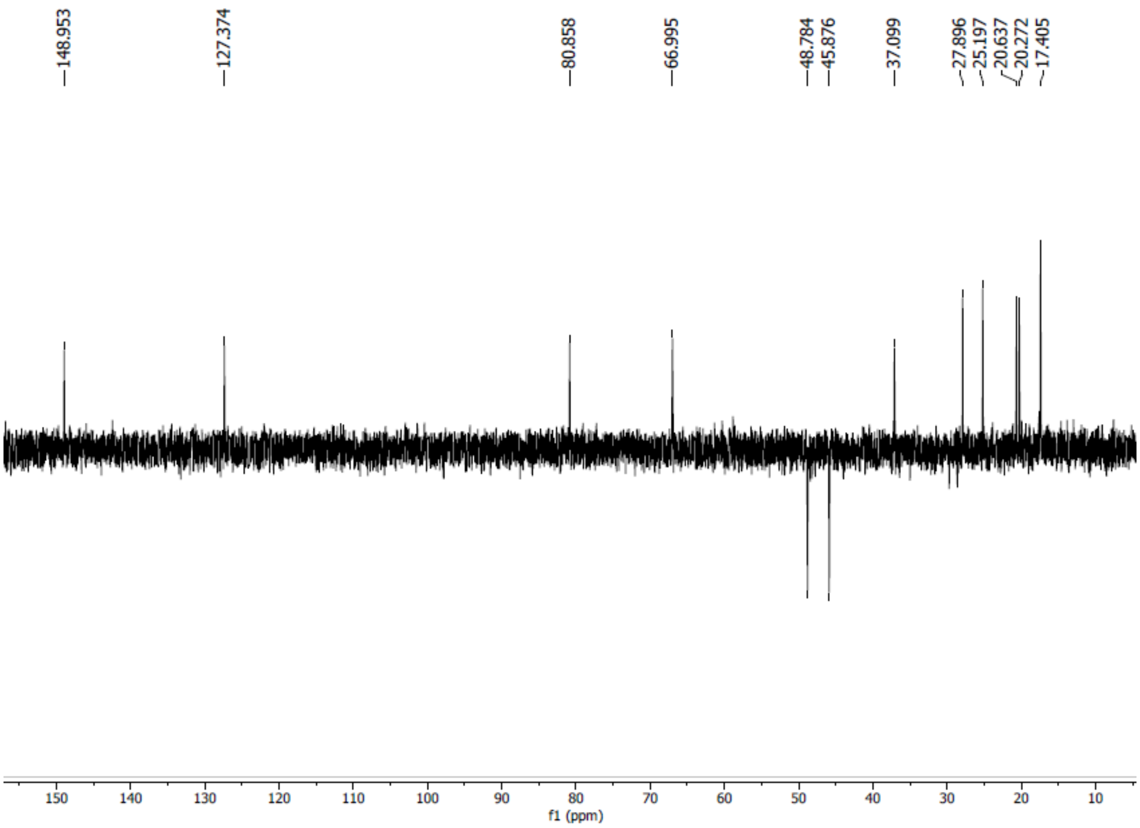

Figure S22. COSY spectrum of Ribifolone B, 400MHz in $CDCl_3$.

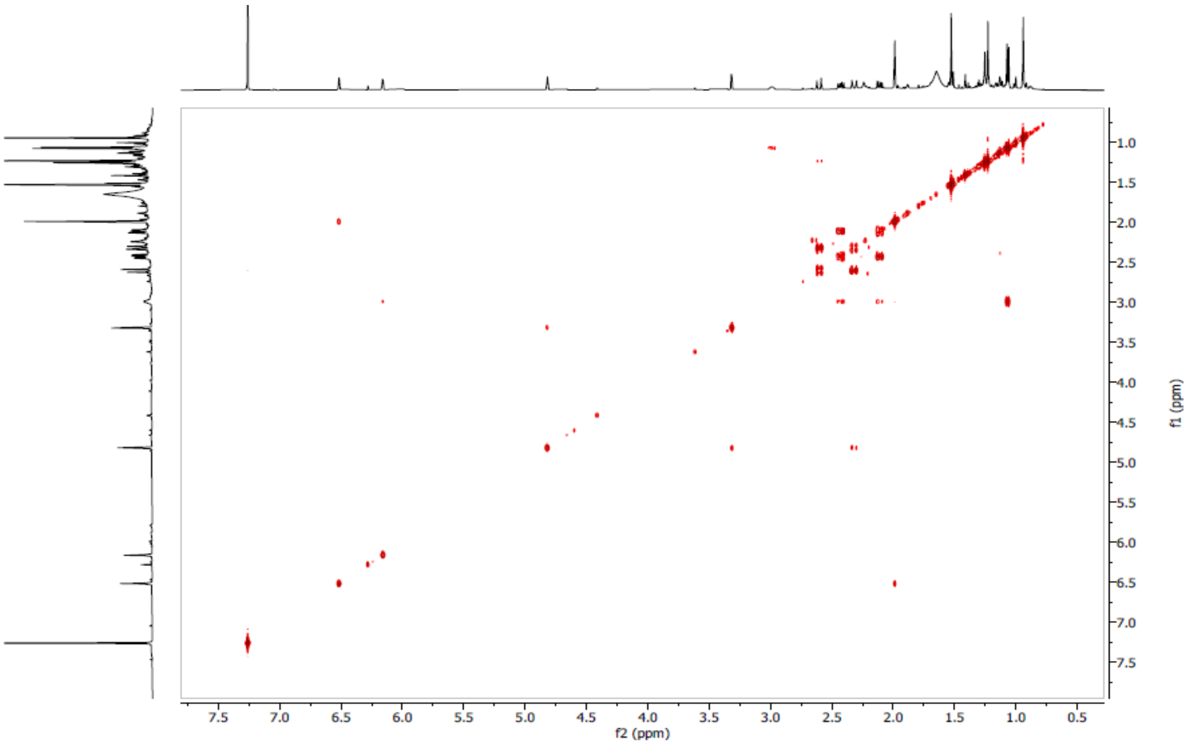

Figure S23. HMBC contour map of Ribifolone B, 100MHz X 400MHz in $CDCl_3$.

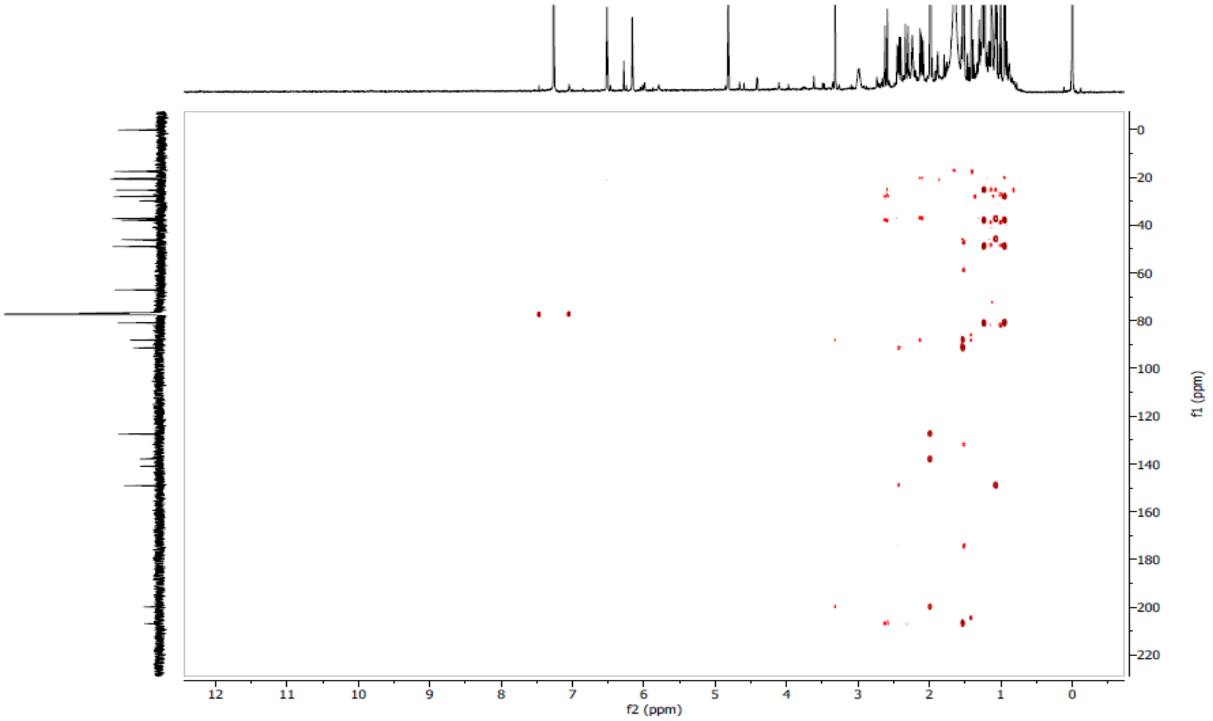

Figure S24. Expansion of HMBC contour map of Ribifolone B in $CDCl_3$.

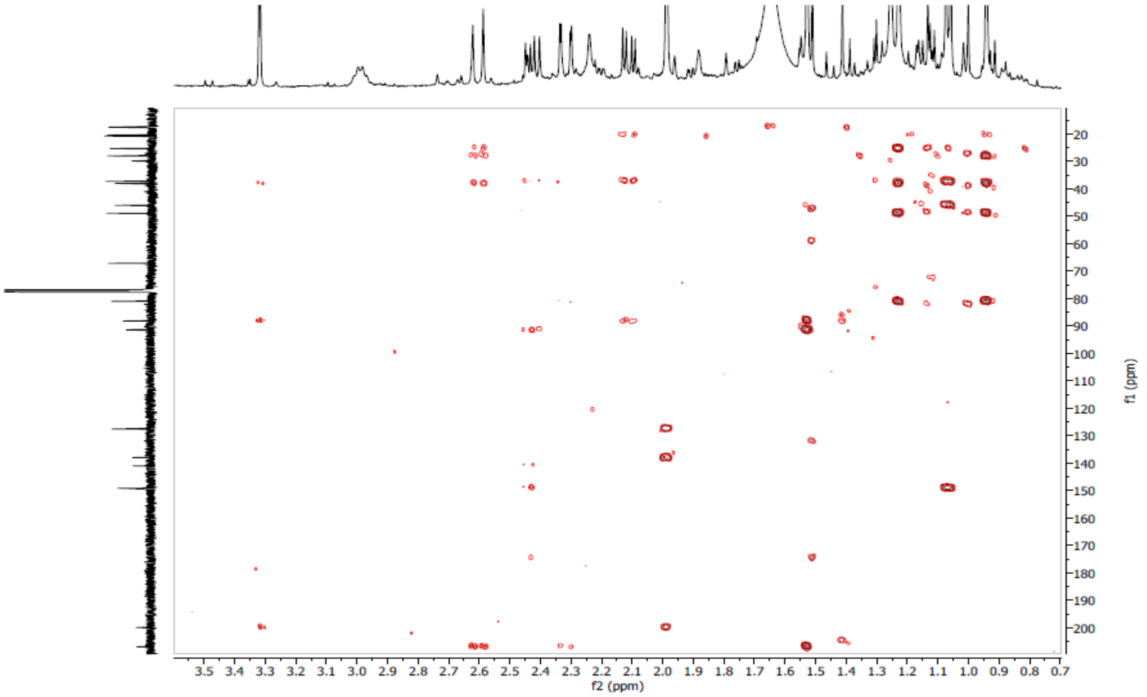

Figure S25. HSQC contour map of Ribifolone B, 100MHz X 400MHz in $CDCl_3$

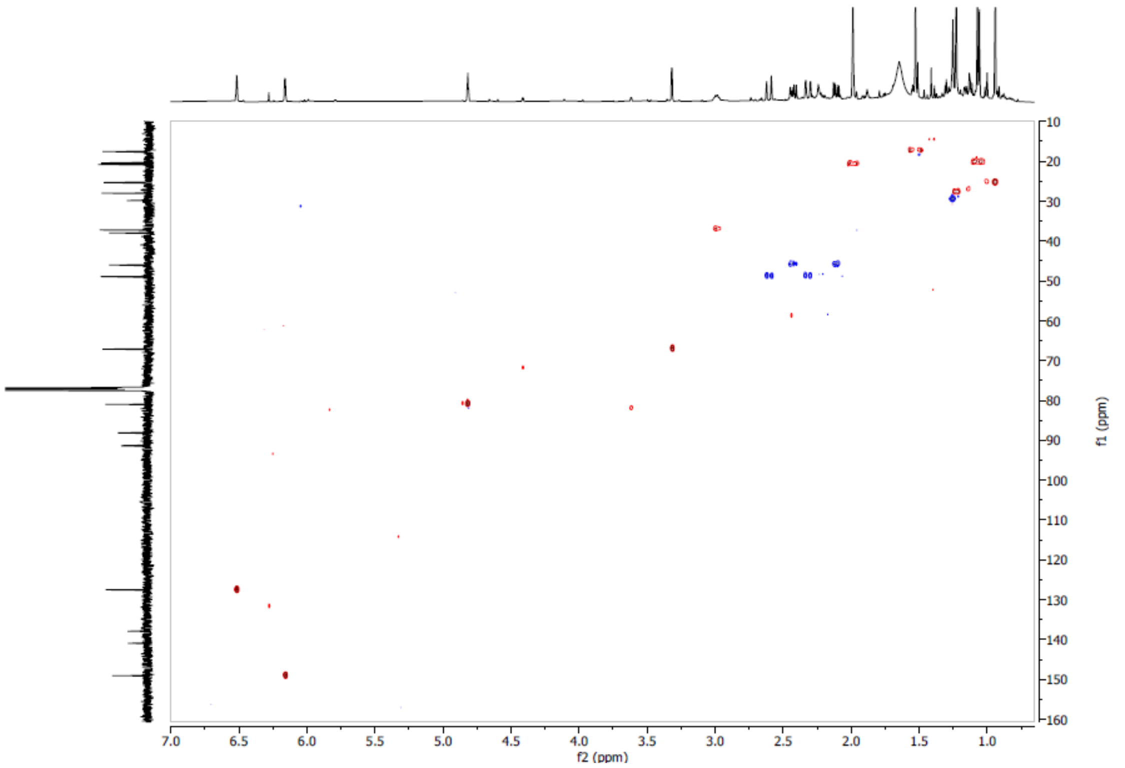

Figure S26. NOESY spectrum of Ribifolone B, 400MHz in $CDCl_3$.

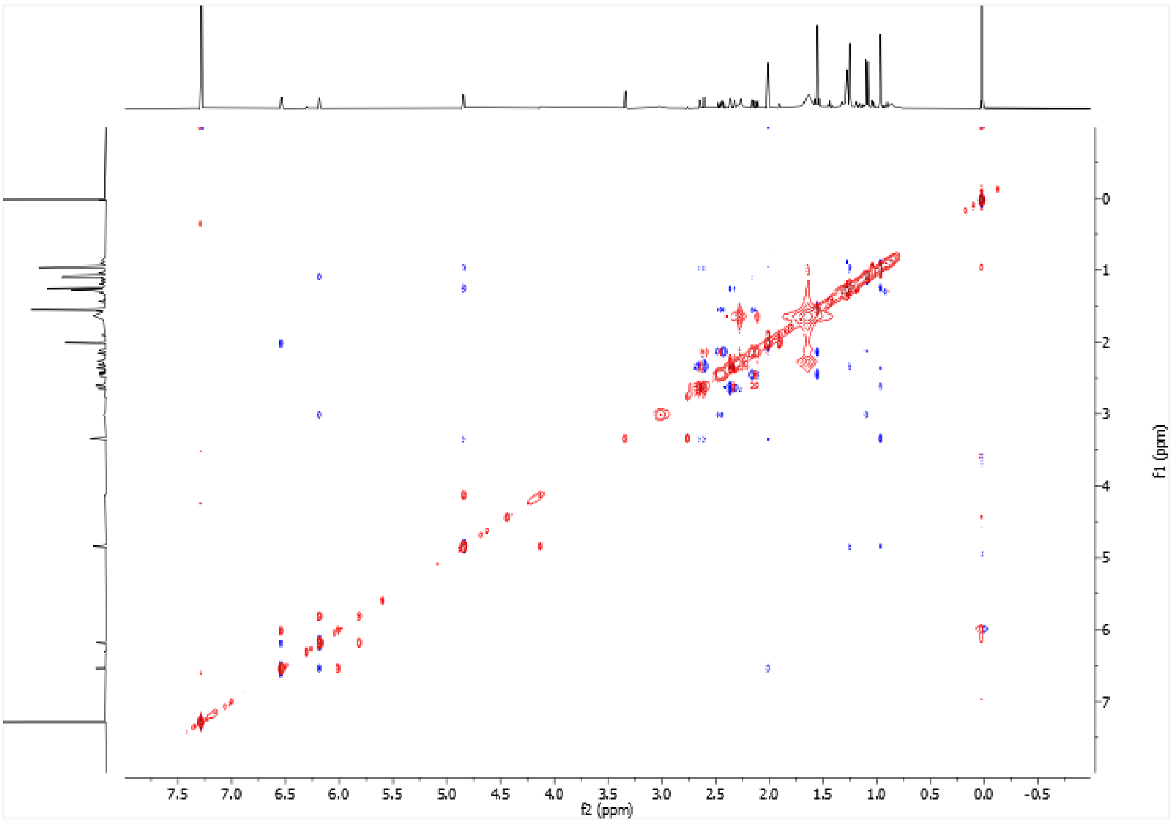

Figure S27. Infrared spectrum of Ribifolone B.

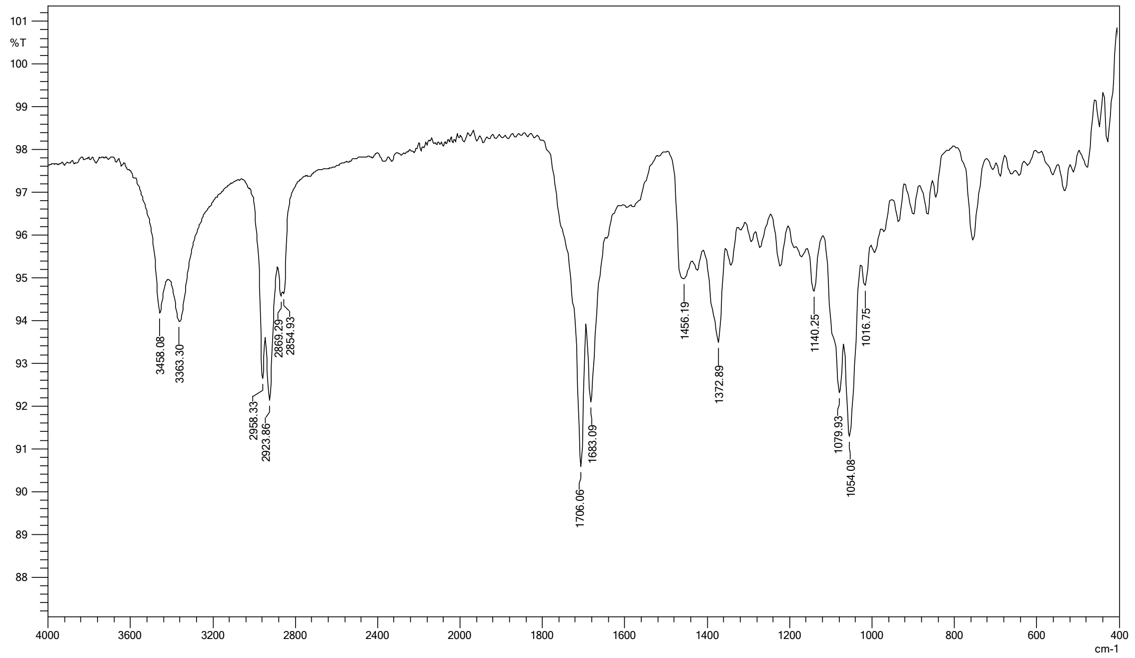

Figure S28. Ultraviolet (UV) spectrum of Ribifolone B.

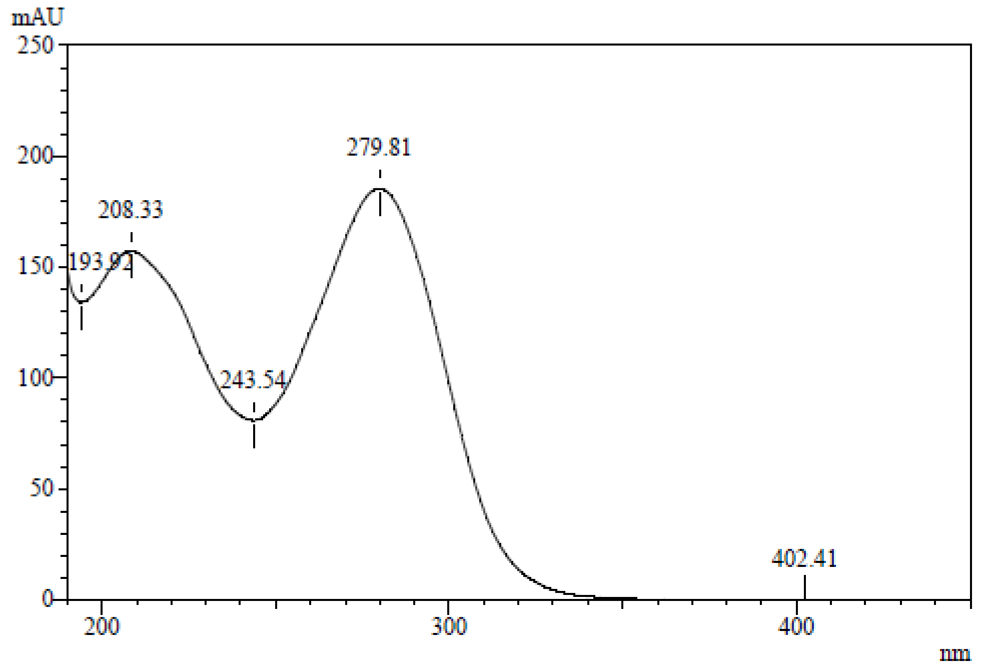

Figure S29. HR-ESI-MS spectrum of Ribifolone C

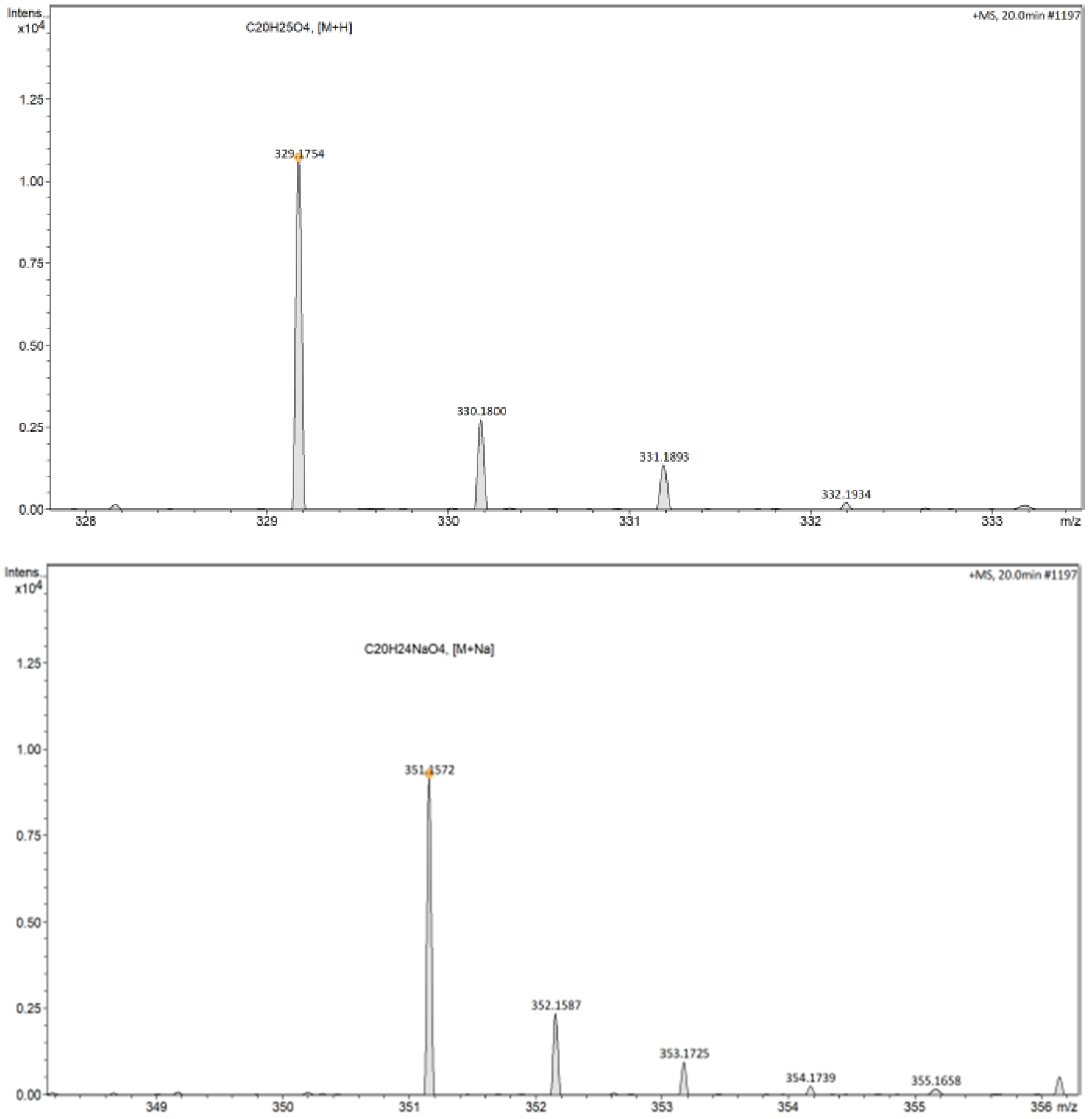

Meas. m/z	#	Ion Formula	m/z	err [ppm]	mSigma	# mSigma	Score	rdb	e⁻ Conf	N-Rule
329.1754	1	C20H25O4	329.1747	-2.1	60.5	1	100.00	8.5	even	ok
	1	C20H25O4	329.1747	-2.1	60.5	1	100.00	8.5	even	ok
351.1572	1	C20H24NaO4	351.1567	-1.6	46.6	1	100.00	8.5	even	ok

Figure S30. ^{1}H spectrum of Ribifolone C, 400MHz in CDCl$_3$

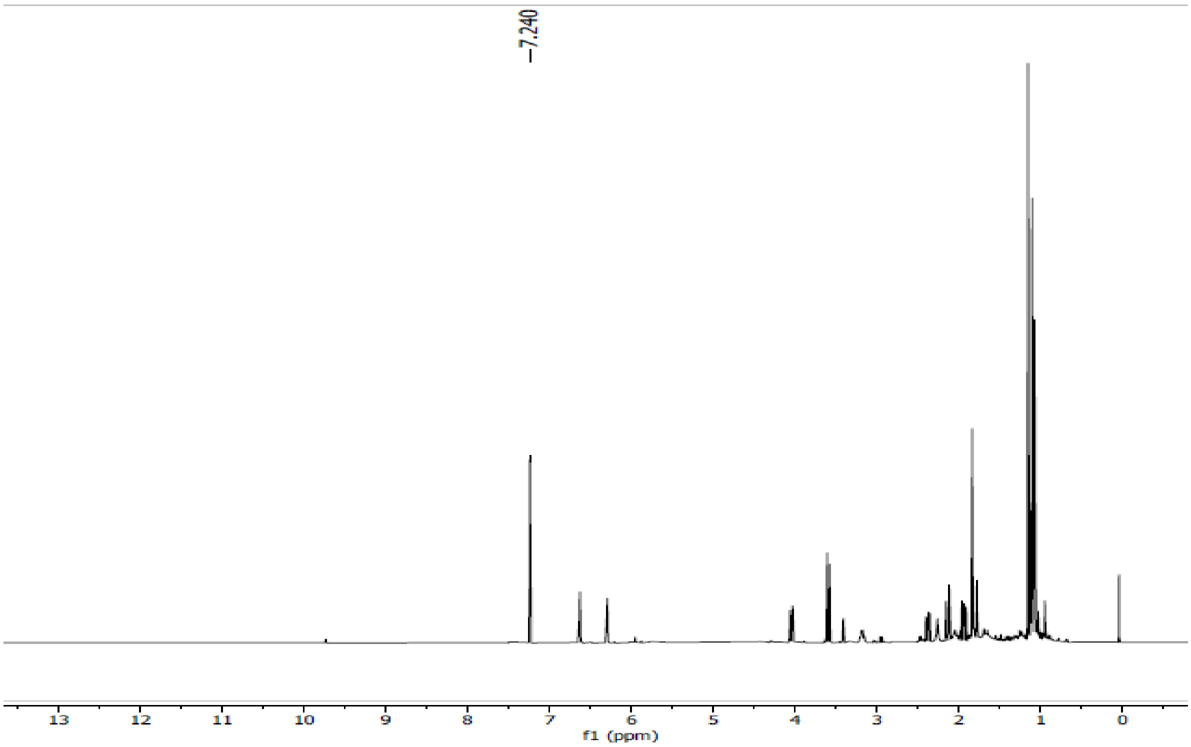

Figure S31. Expansion of ^{1}H spectrum of Ribifolone C, in CDCl$_3$ (1.0-4.1 ppm).

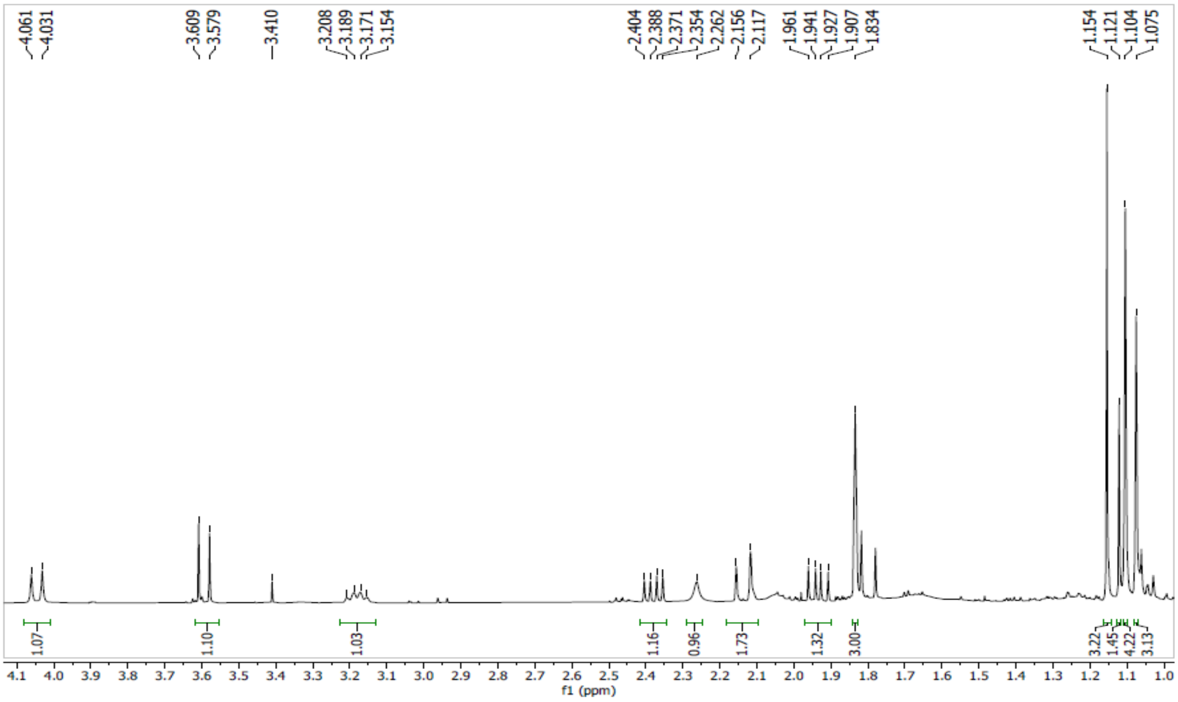

Figure S32. Expansion of ^1H spectrum of Ribifolone C in $CDCl_3$.

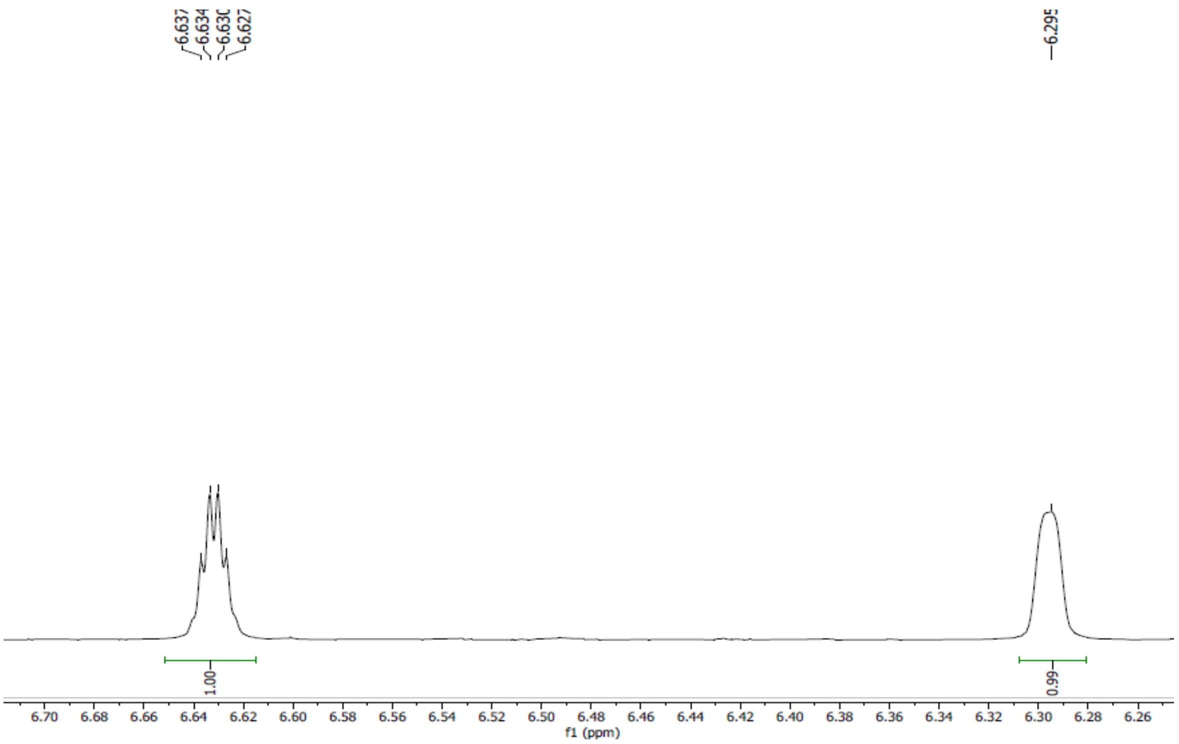

Figure S33. ^{13}C spectrum of Ribifolone C, 100MHz in $CDCl_3$

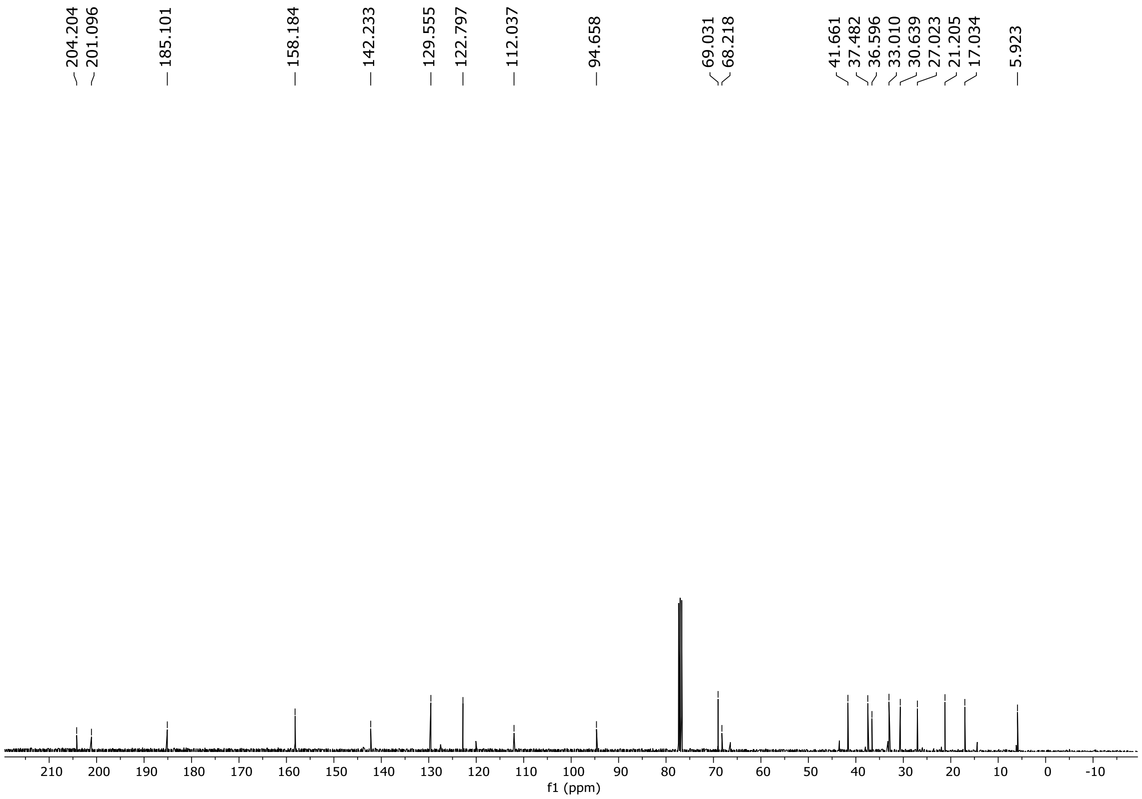

Figure S34. DEPT135 spectrum of Ribifolone C, 100 MHz in $CDCl_3$

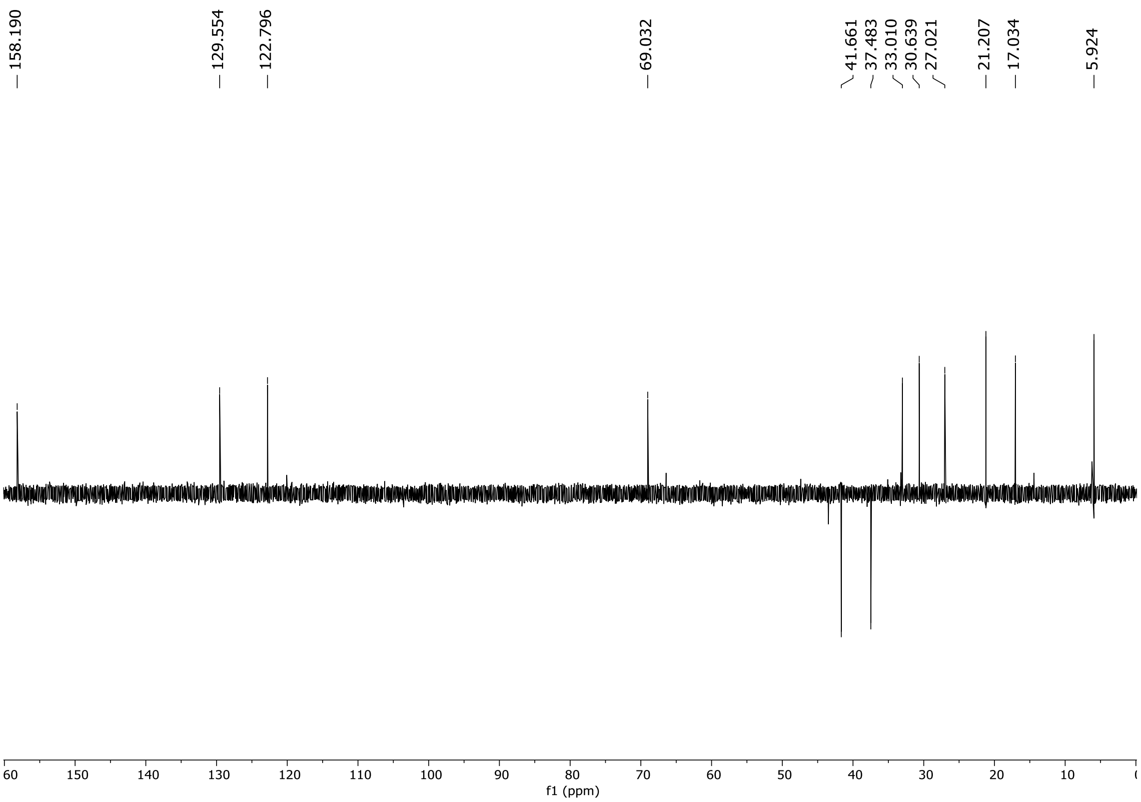

Figure S35. COSY spectrum of Ribifolone C, 400MHz in $CDCl_3$

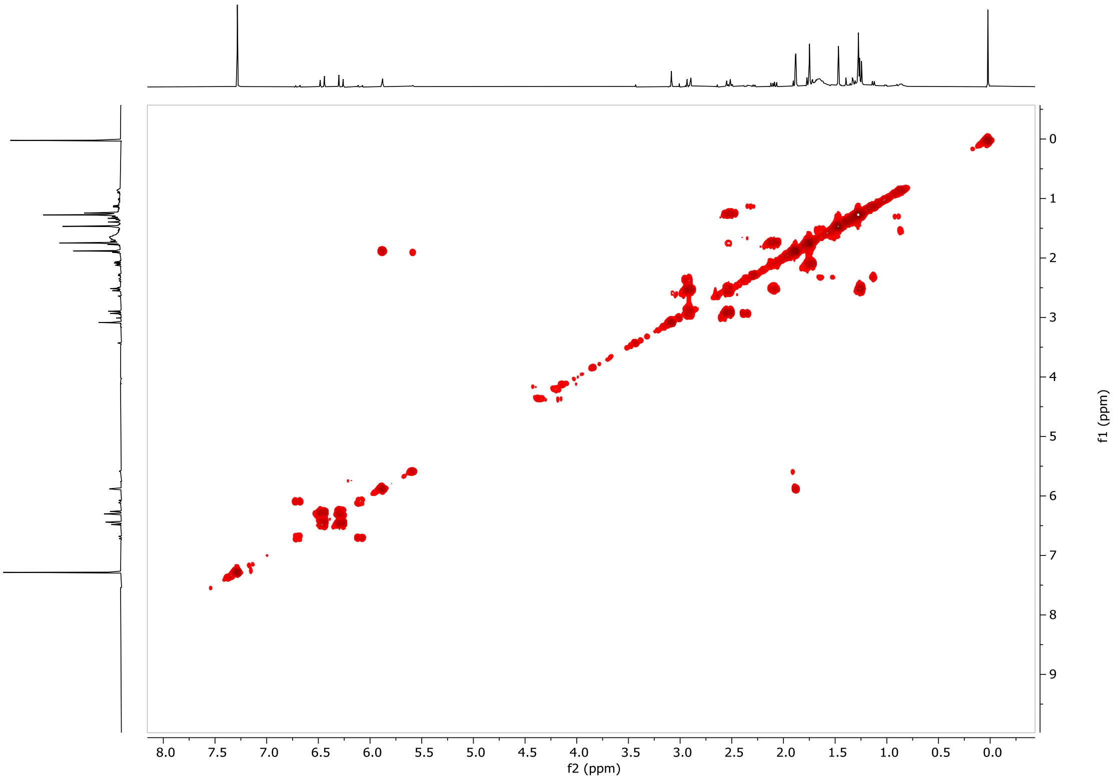

Figure S36. HMBC contour map of Ribifolone C, 100MHz X 400MHz in $CDCl_3$.

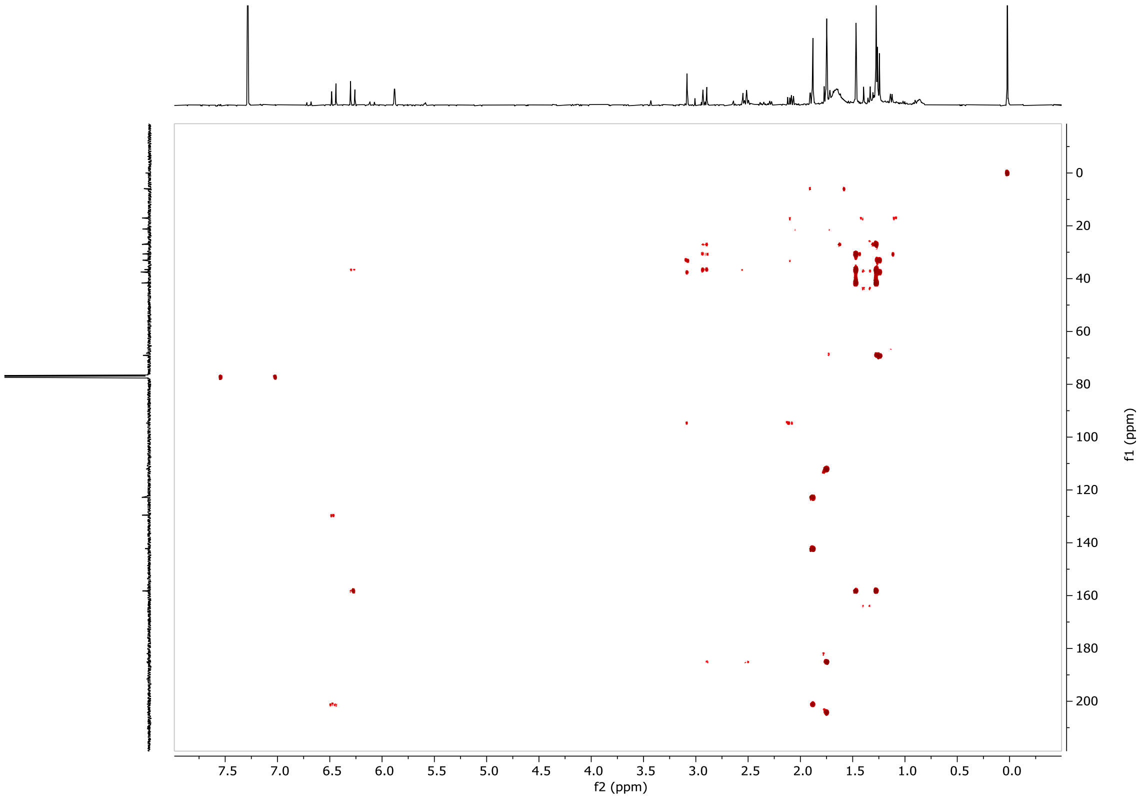

Figure S37. Expansion of HMBC contour map of Ribifolone C.

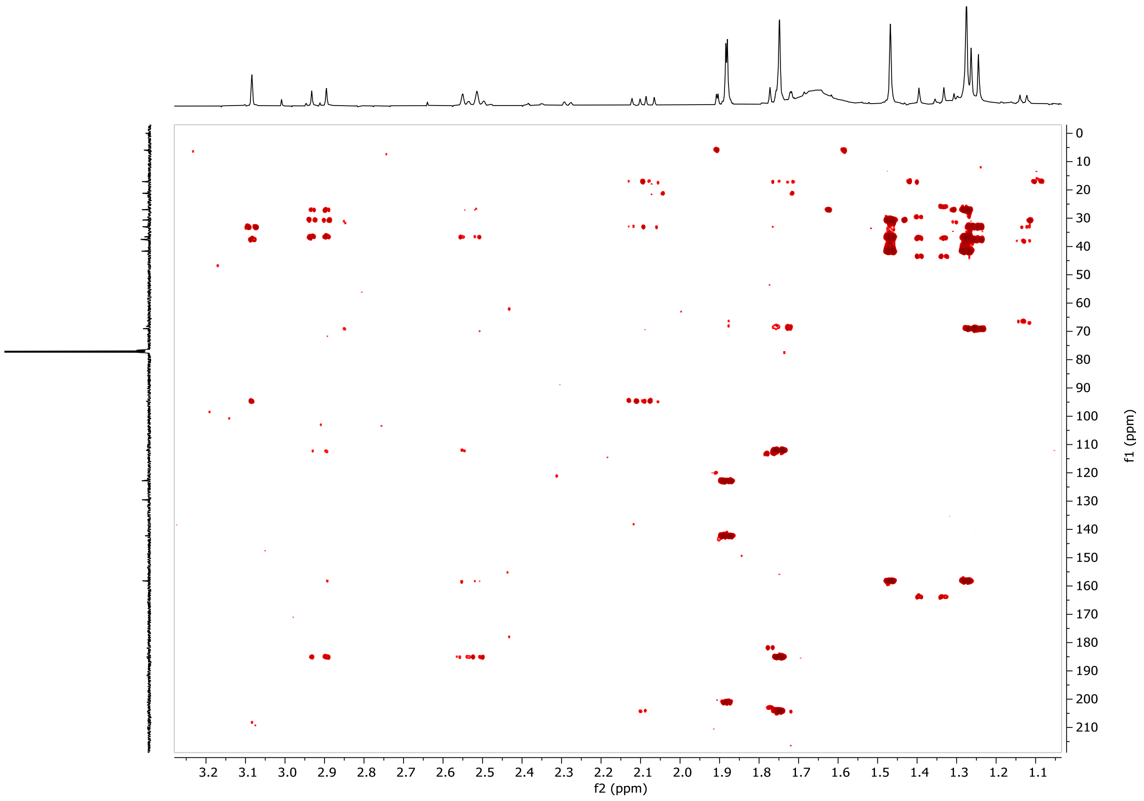

Figure S38. Expansion of HMBC contour map of Ribifolone C.

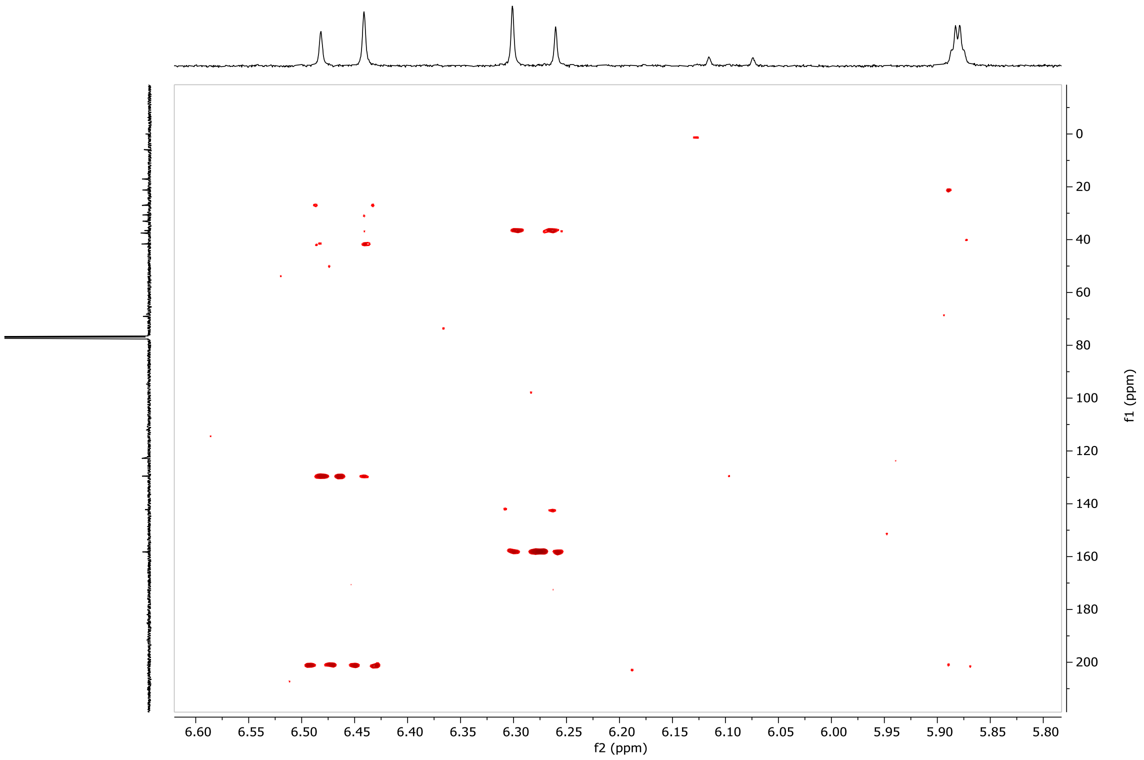

Figure S39. HSQC contour map of Ribifolone C, 100MHz X 400MHz in $CDCl_3$.

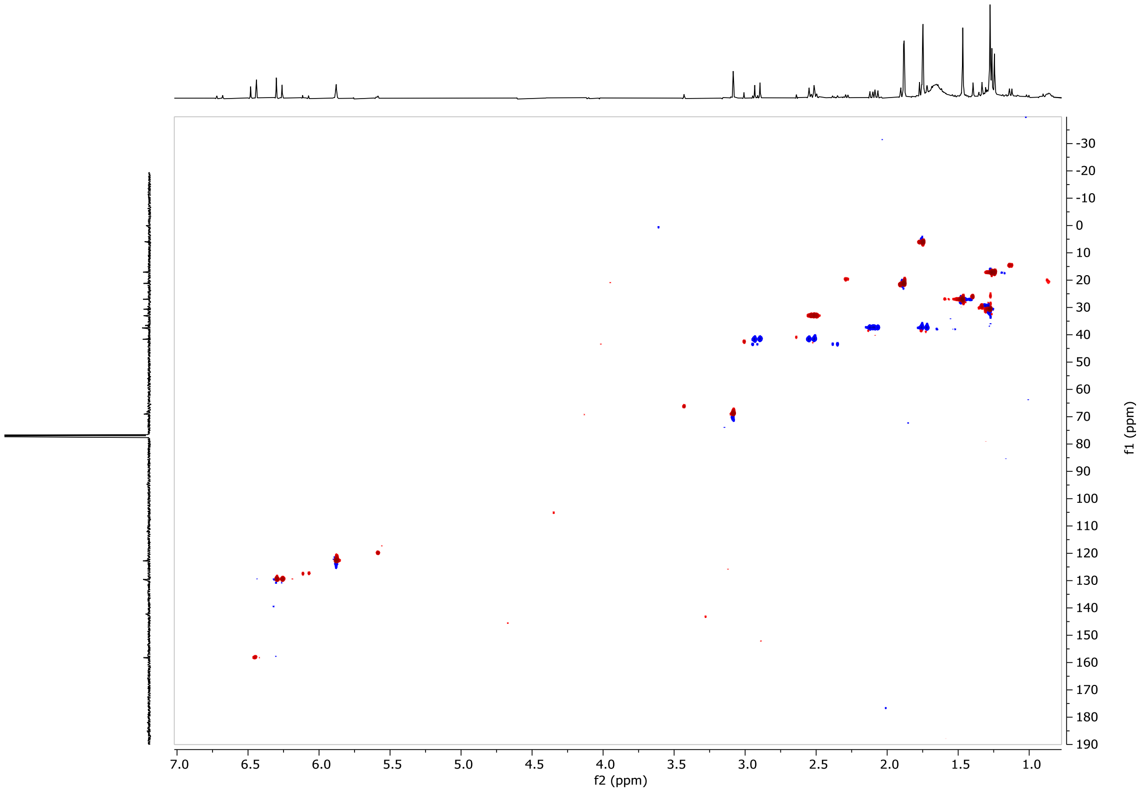

Figure S40. NOESY contour map of Ribifolone C 400MHz in $CDCl_3$

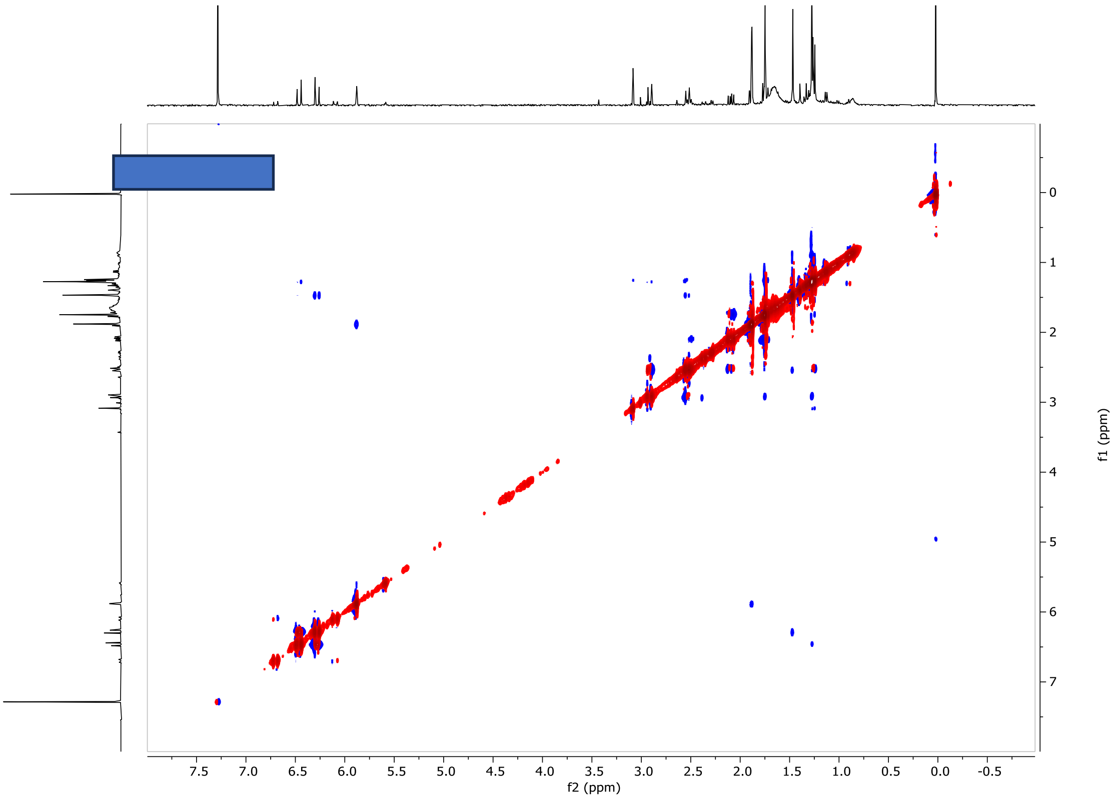

Figure S41. Infrared spectrum of Ribifolone C.

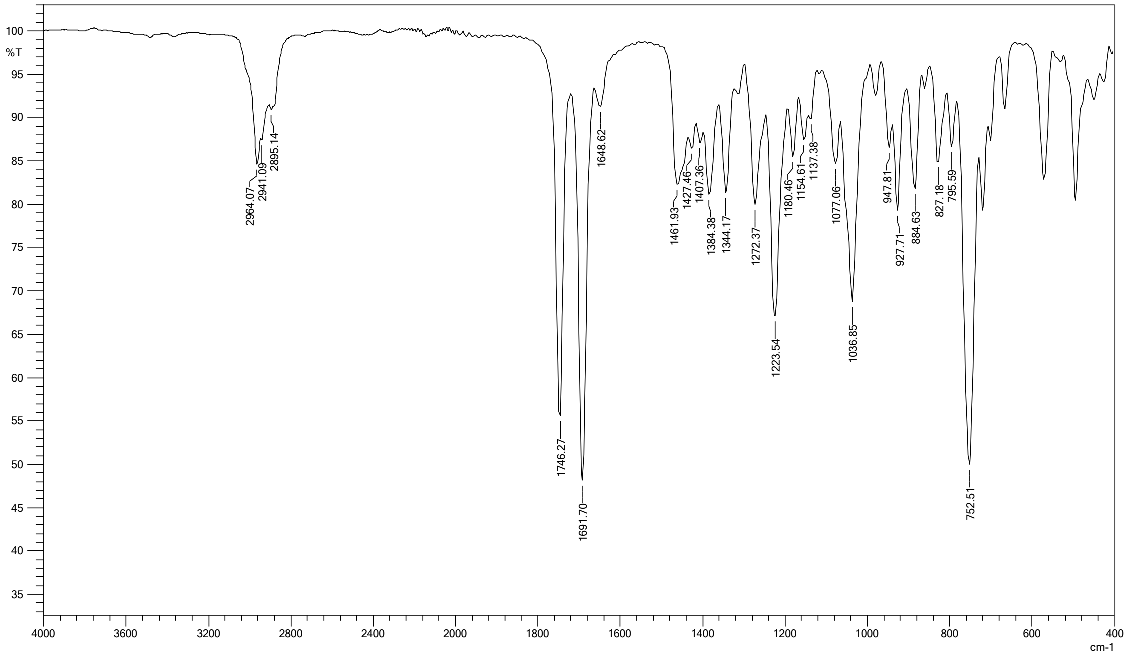

Figure S42. Ultraviolet (UV) spectrum of Ribifolone C.

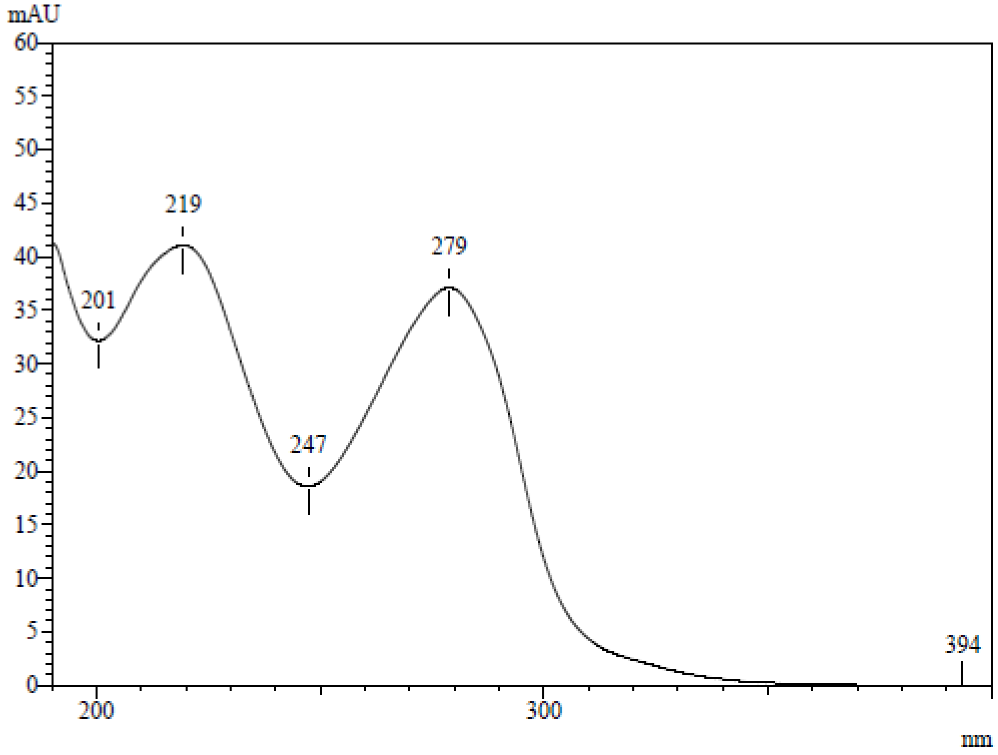

Figure S43. HR-ESI-MS spectrum of Ribifolone D

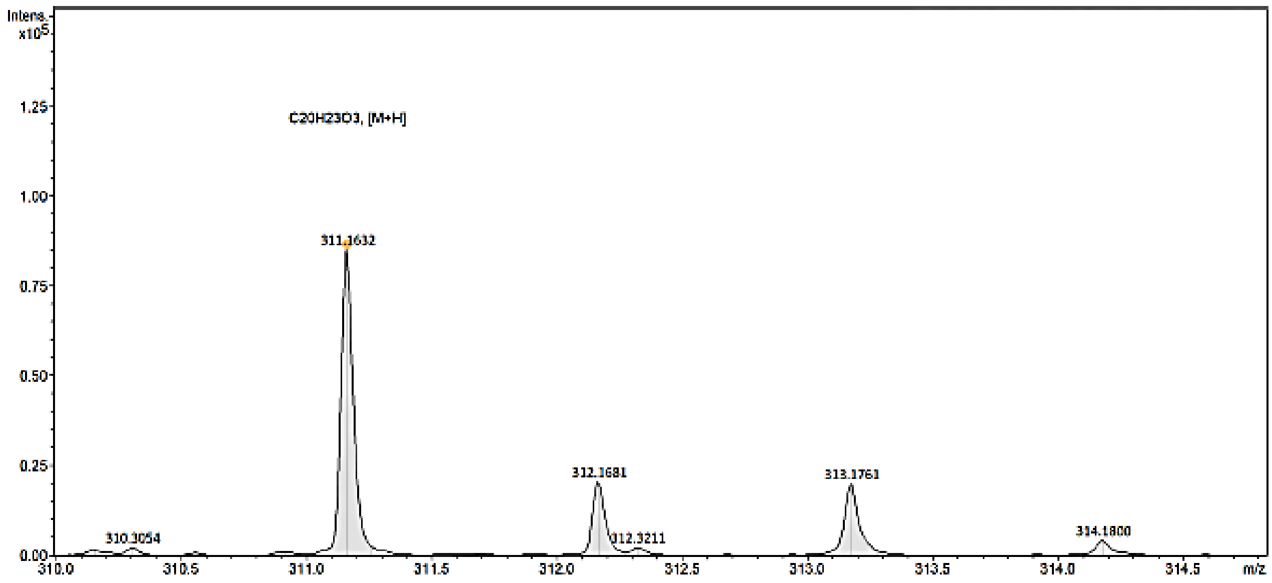

Meas. m/z	#	Ion Formula	m/z	err [ppm]	mSigma	#mSigma	Score	rdb	e¯ Conf	N-Rule
311.1632	1	C20H23O3	311.1642	3.3	118.6	1	100.00	9.5	even	ok

Figure S44. ^{1}H spectrum of Ribifolone D, 400MHz in $CDCl_3$.

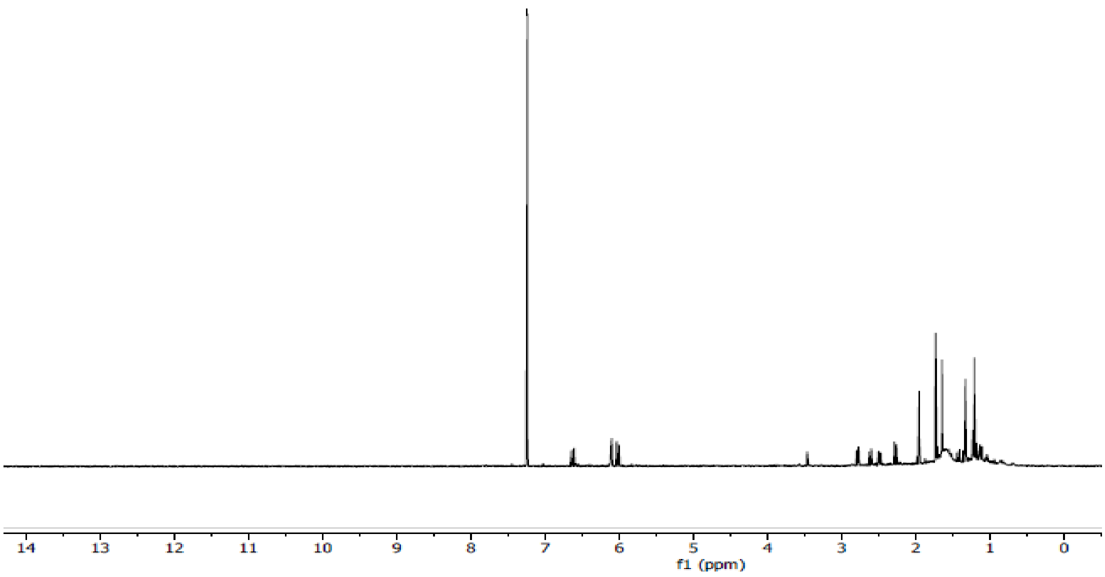

Figure S45. Expansion ^{1}H spectrum of Ribifolone D in $CDCl_3$.

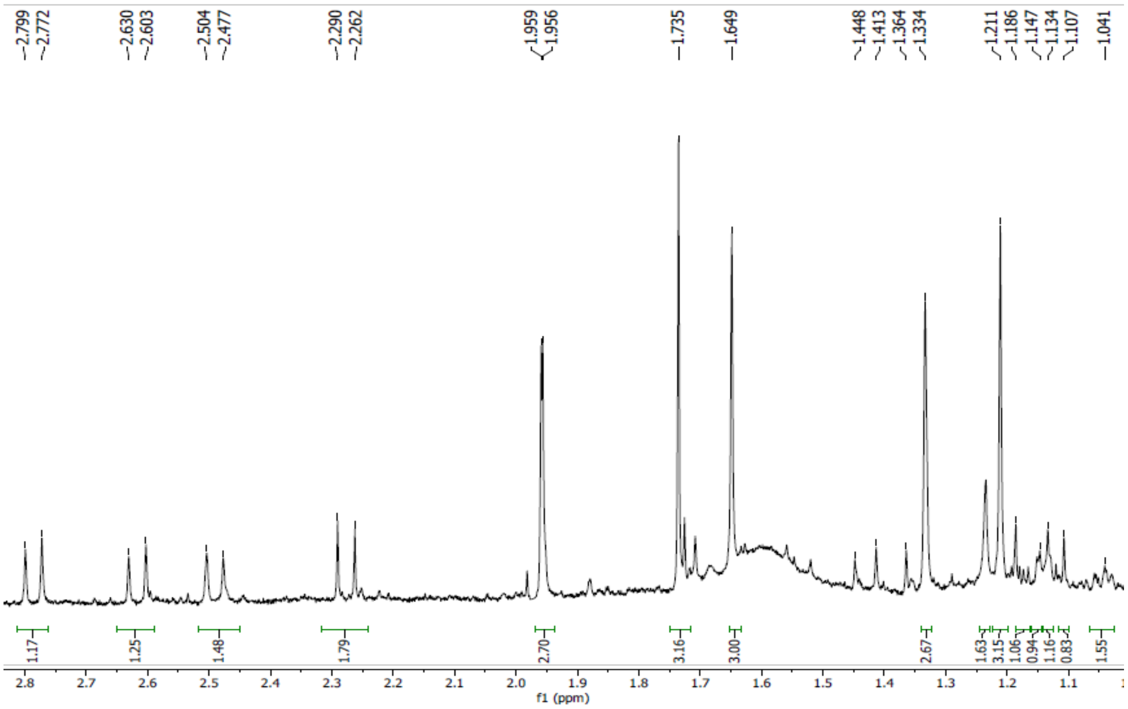

Figure S46. ^{13}C spectrum of Ribifolone D, 100MHz in $CDCl_3$.

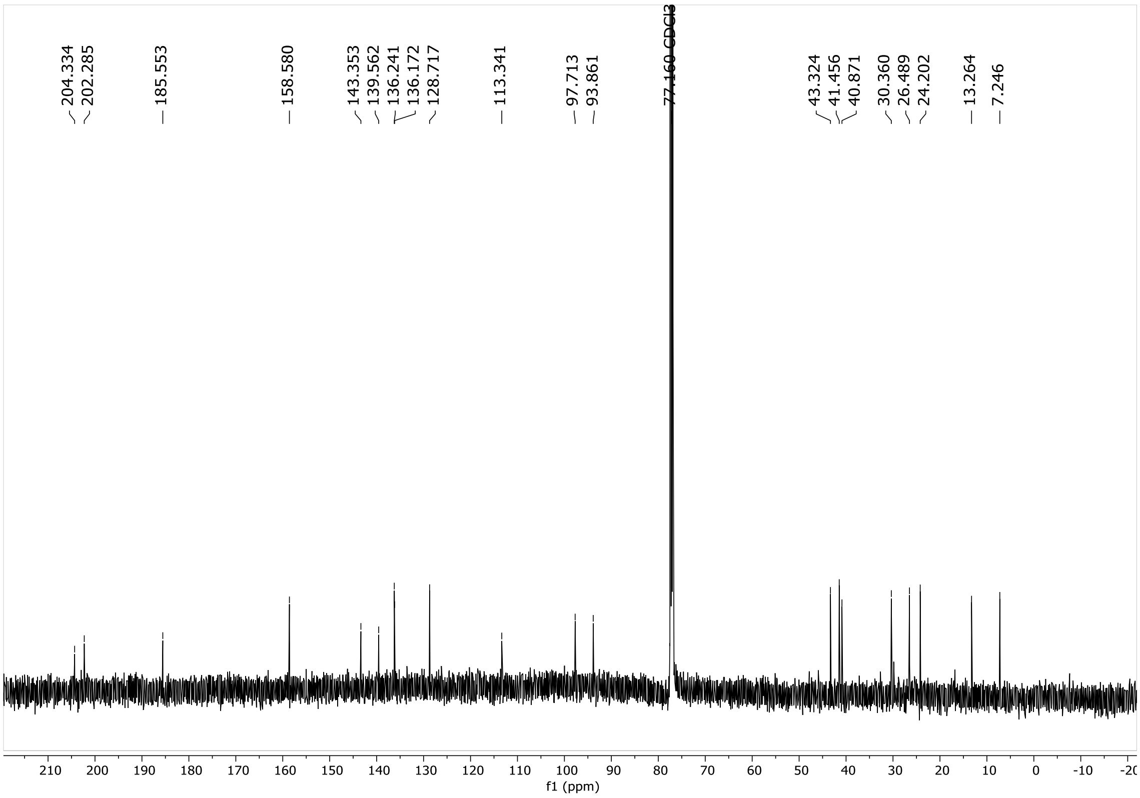

Figure S47. DEPT135 spectrum of Ribifolone D, 100MHz in $CDCl_3$.

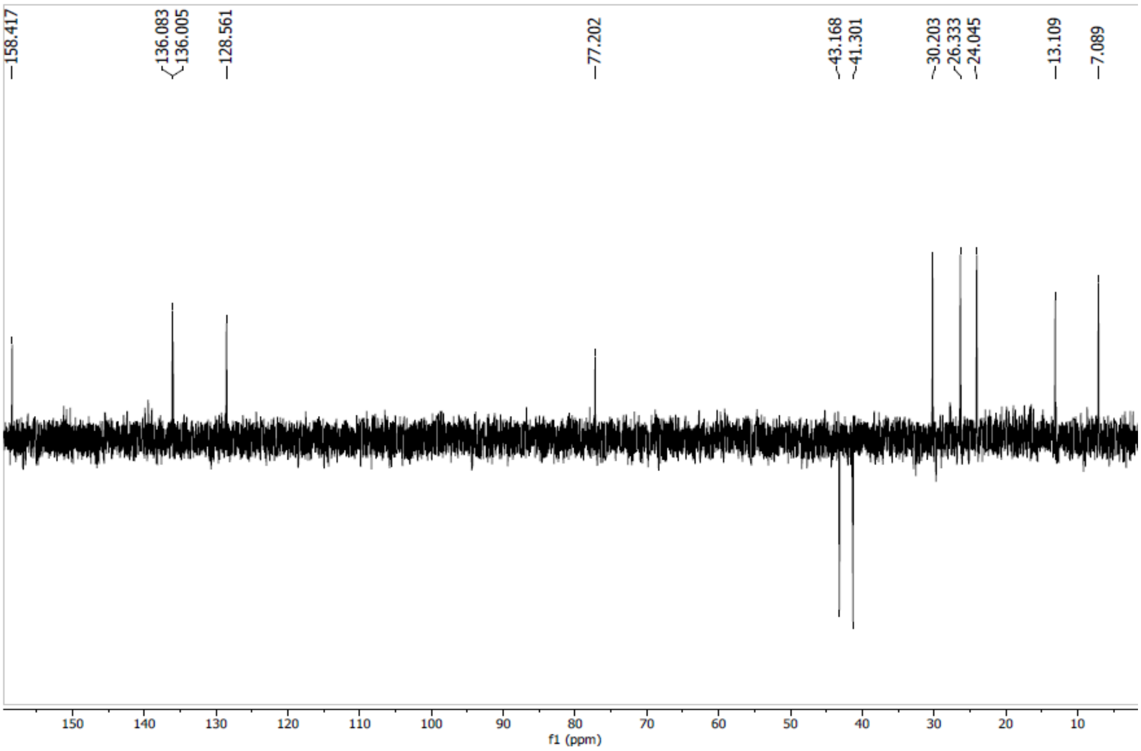

Figure S48. COSY spectrum of Ribifolone D, 400MHz in $CDCl_3$.

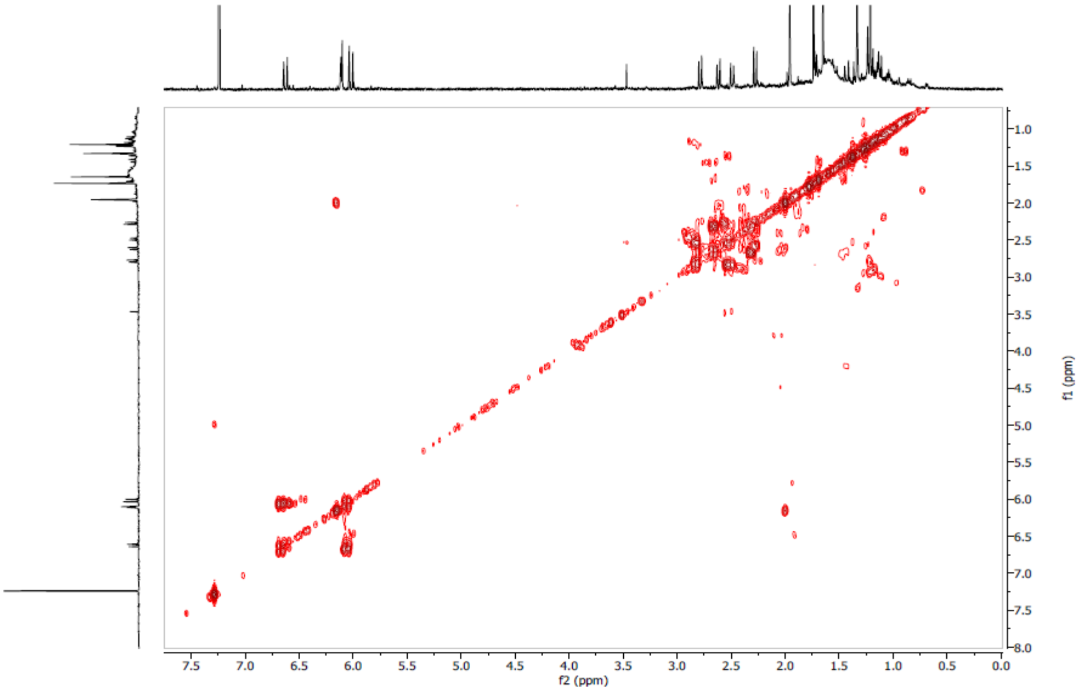

Figure S49. HMBC contour map of Ribifolone D, 100MHz X 400MHz in $CDCl_3$.

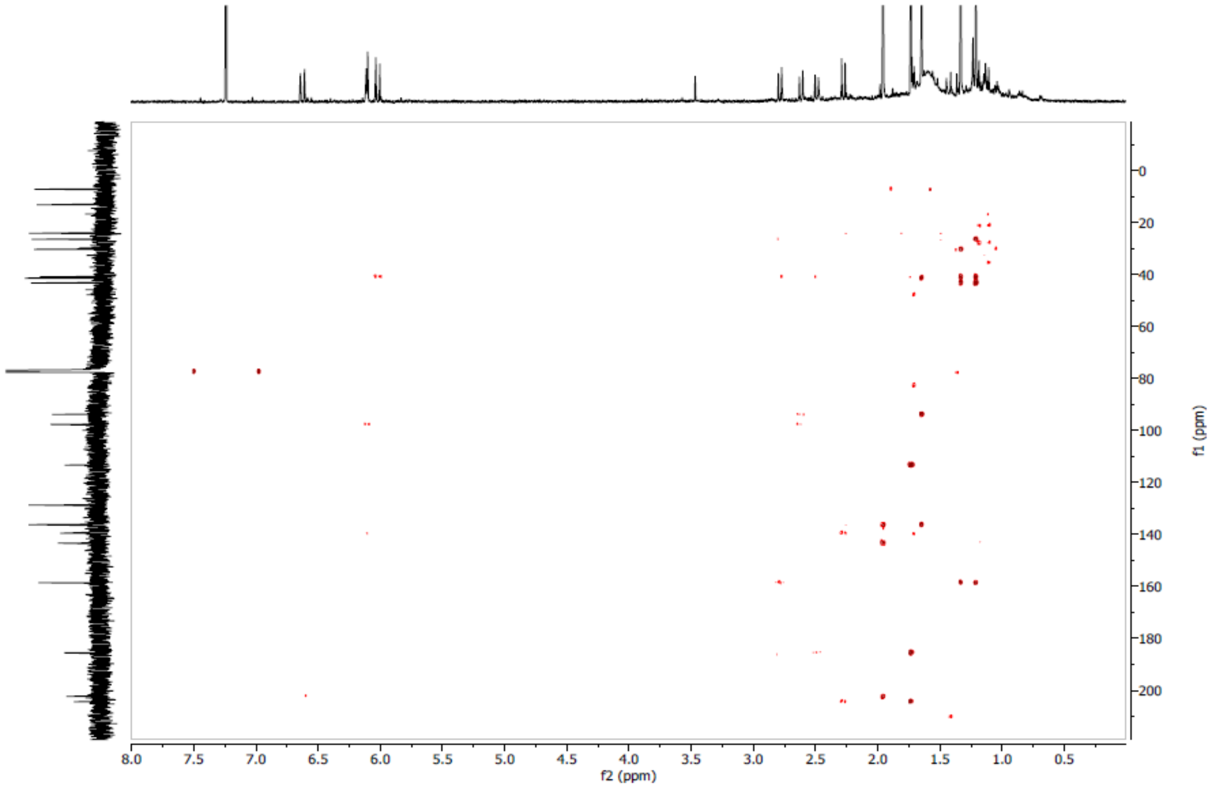

Figure S50. Expansion of HMBC contour map of Ribifolone D.

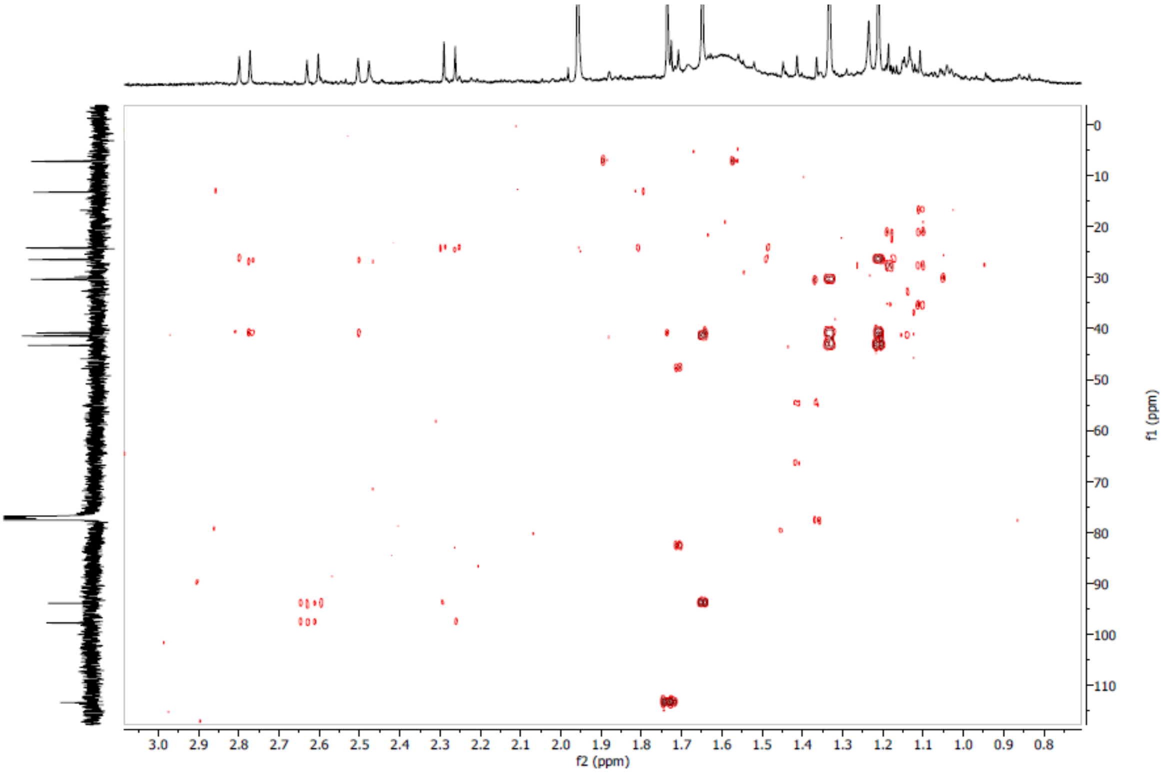

Figure S51. HSQC contour map of Ribifolone D, 100MHz X 400MHz in $CDCl_3$.

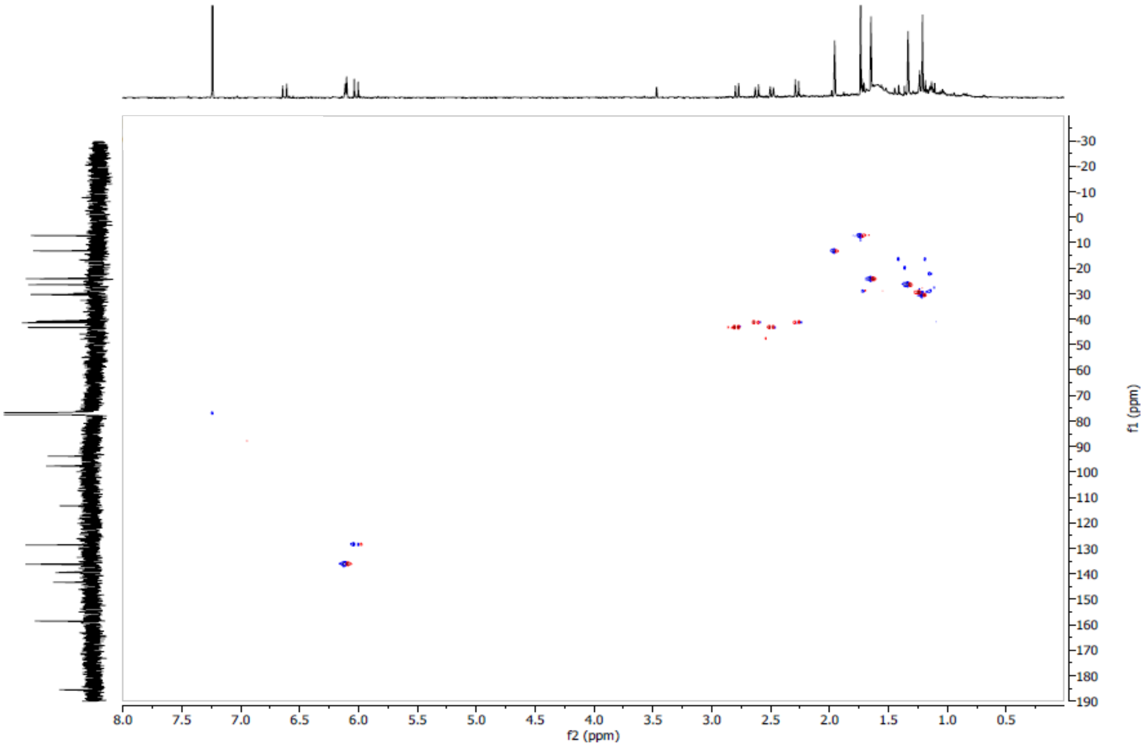

Figure S52. NOESY spectrum of Ribifolone D, 400MHz in $CDCl_3$.

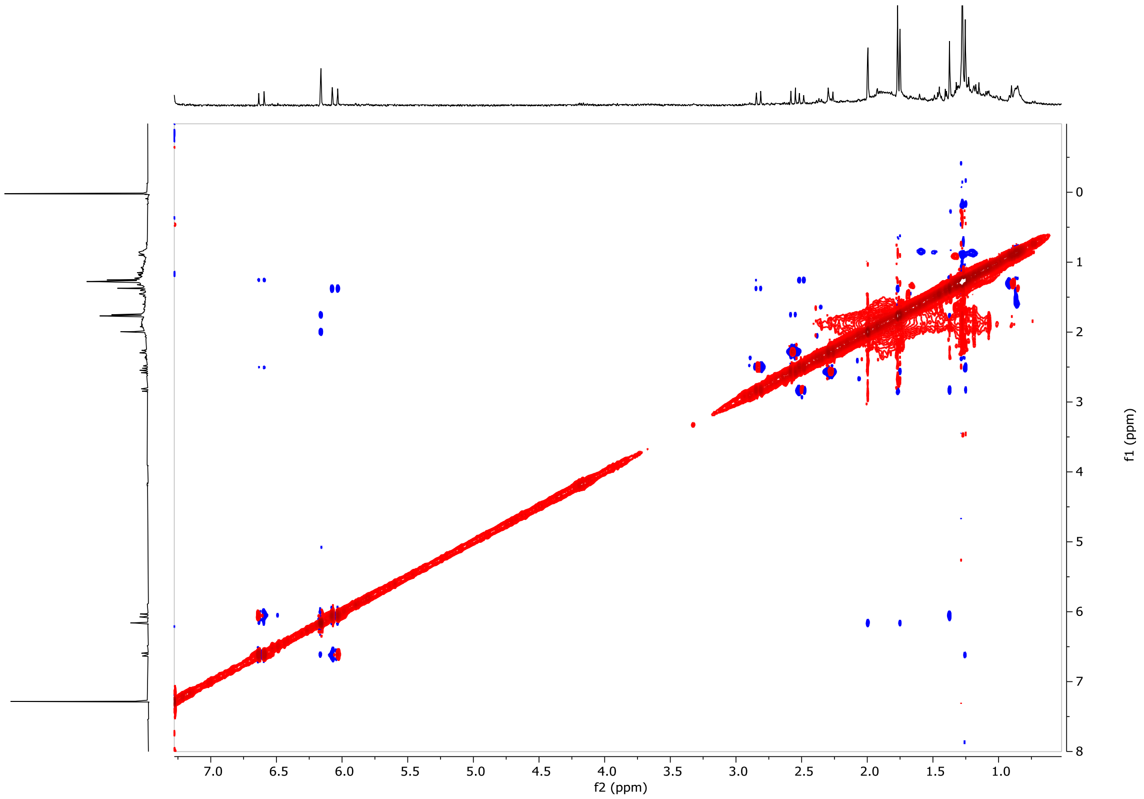

Figure S53. Infrared spectrum of Ribifolone D.

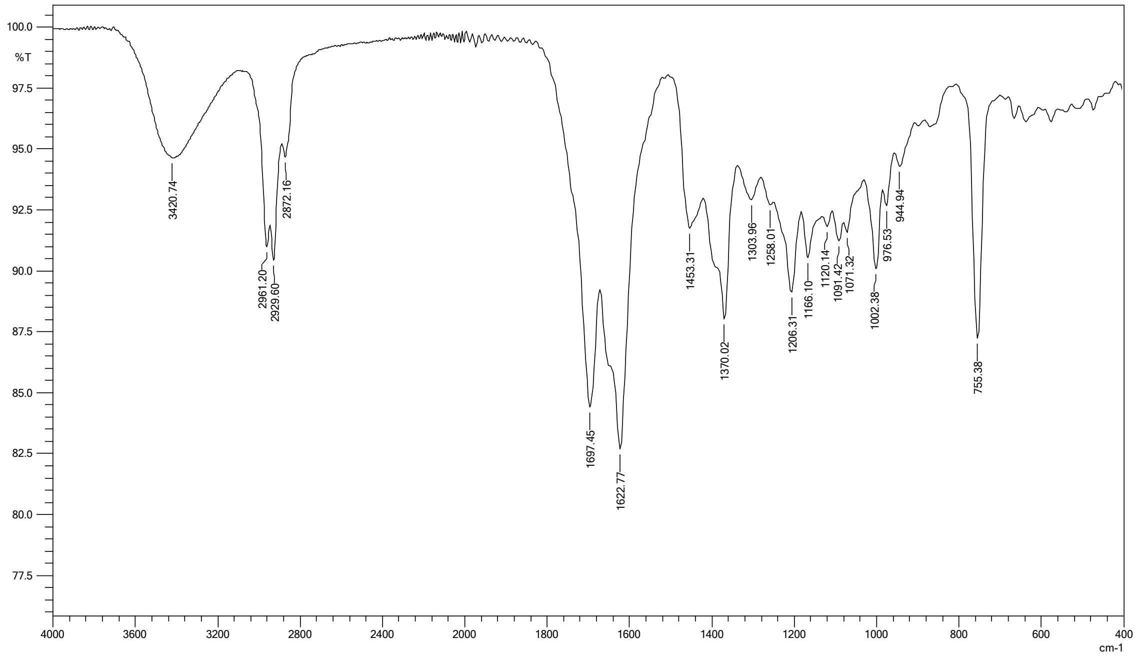

Figure S54. Ultraviolet (UV) spectrum of Ribifolone D.

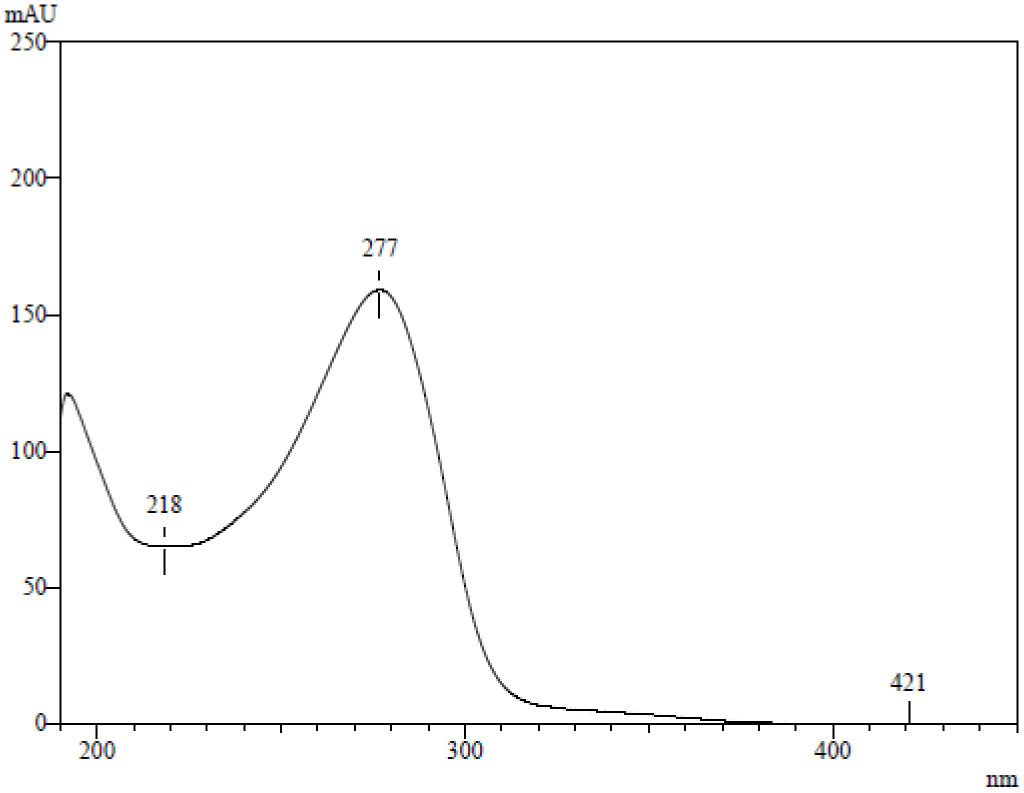

Figure S55. HR-ESI-MS spectrum of Ribifolone E.

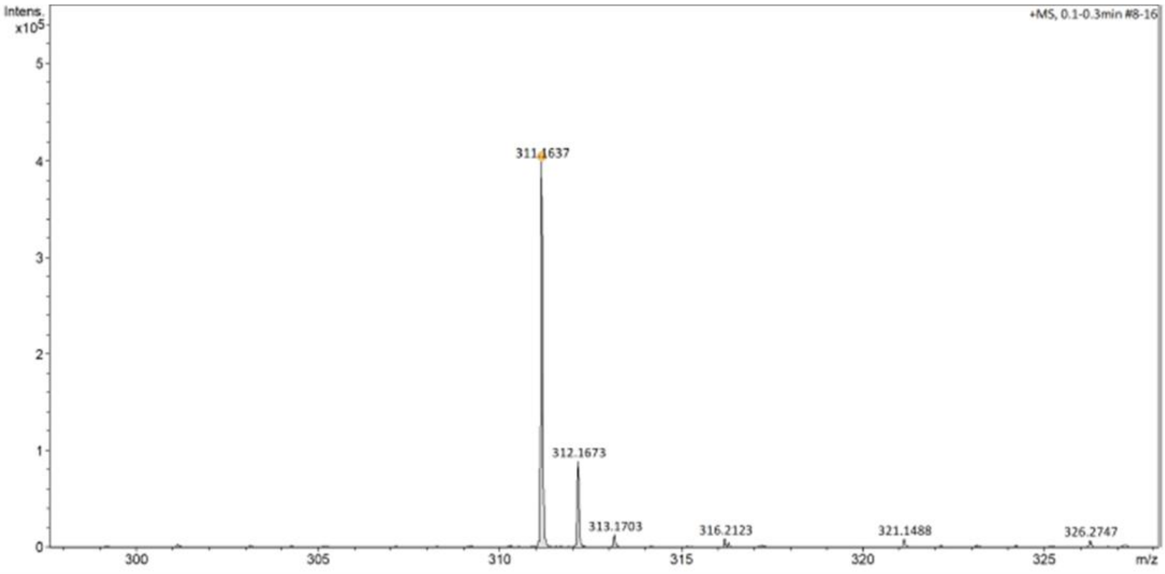

Meas. m/z	#	Ion Formula	m/z	err [ppm]	mSigma	# mSigma	Score	rdb	e⁻ Conf	N-Rule
311.1637	1	C20H23O3	311.1642	1.5	1.8	1	100.00	9.5	even	ok

Figure S56. ^{1}H spectrum of Ribifolone E, 400MHz in $CDCl_3$.

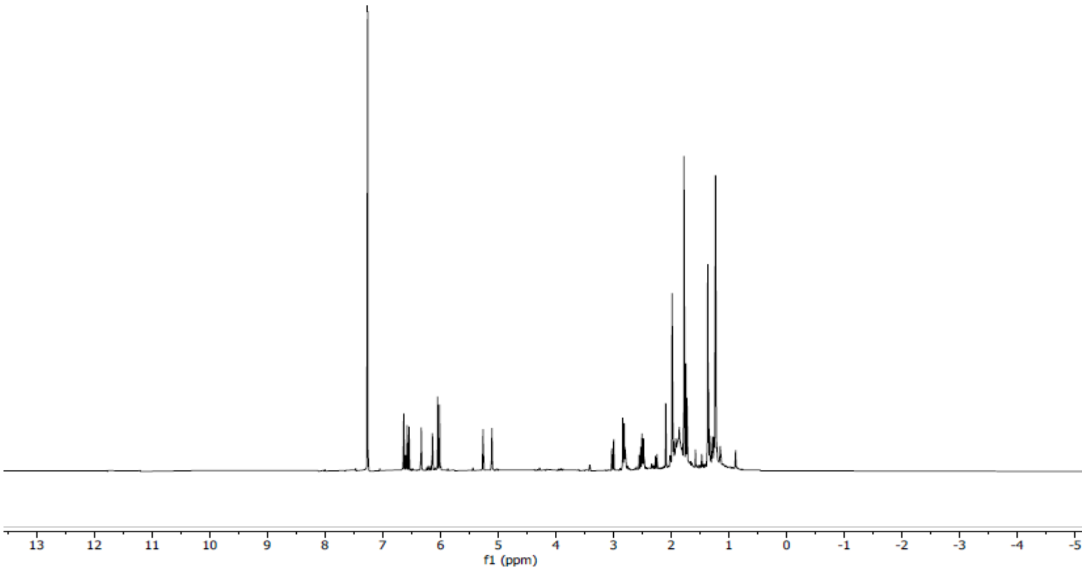

Figure S57. Expansion of ^{1}H spectrum of Ribifolone E in $CDCl_3$.

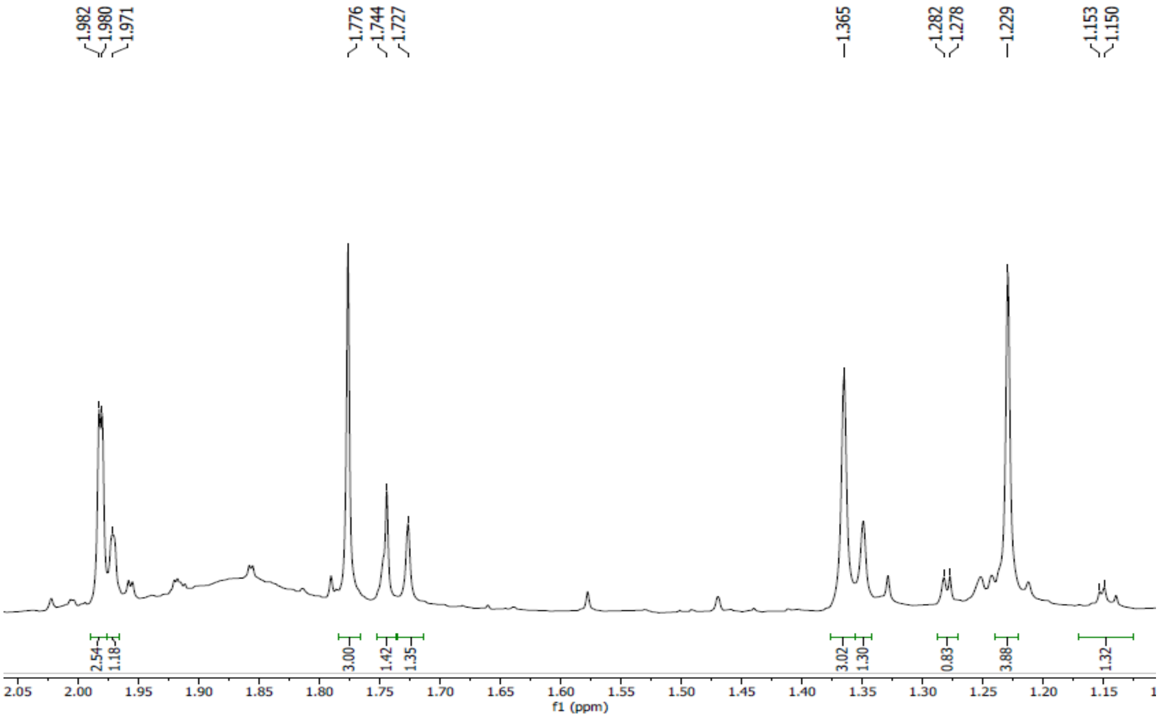

Figure S58. Expansion of ^{1}H spectrum of Ribifolone E in $CDCl_3$.

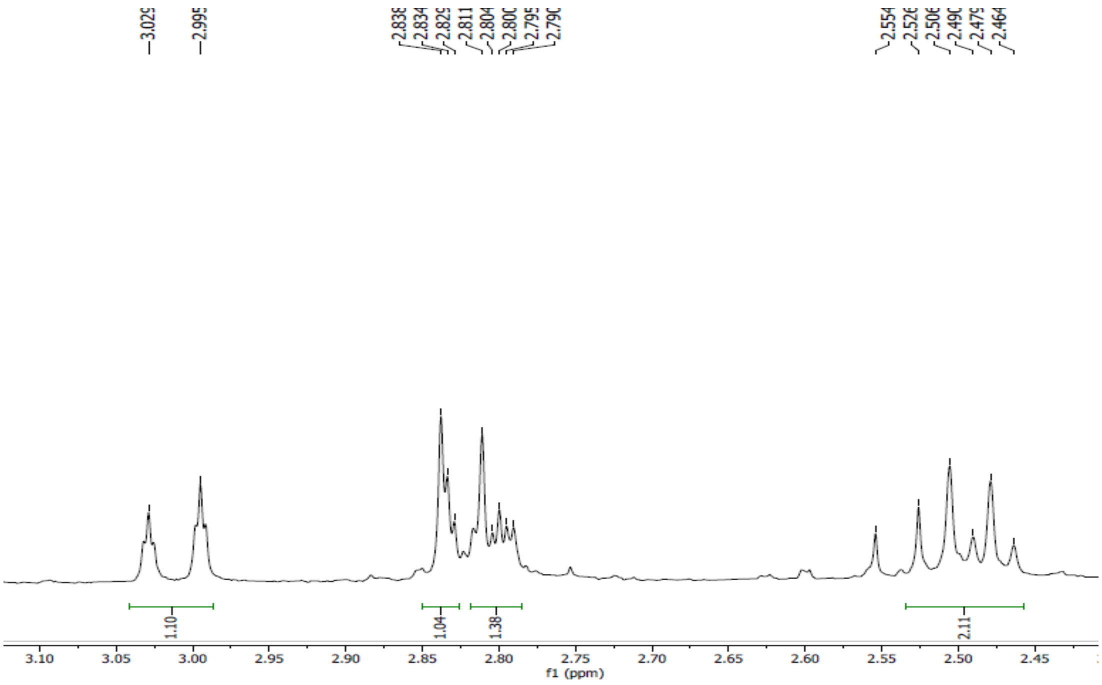

Figure S59. Expansion of ^{1}H spectrum of Ribifolone E in $CDCl_3$

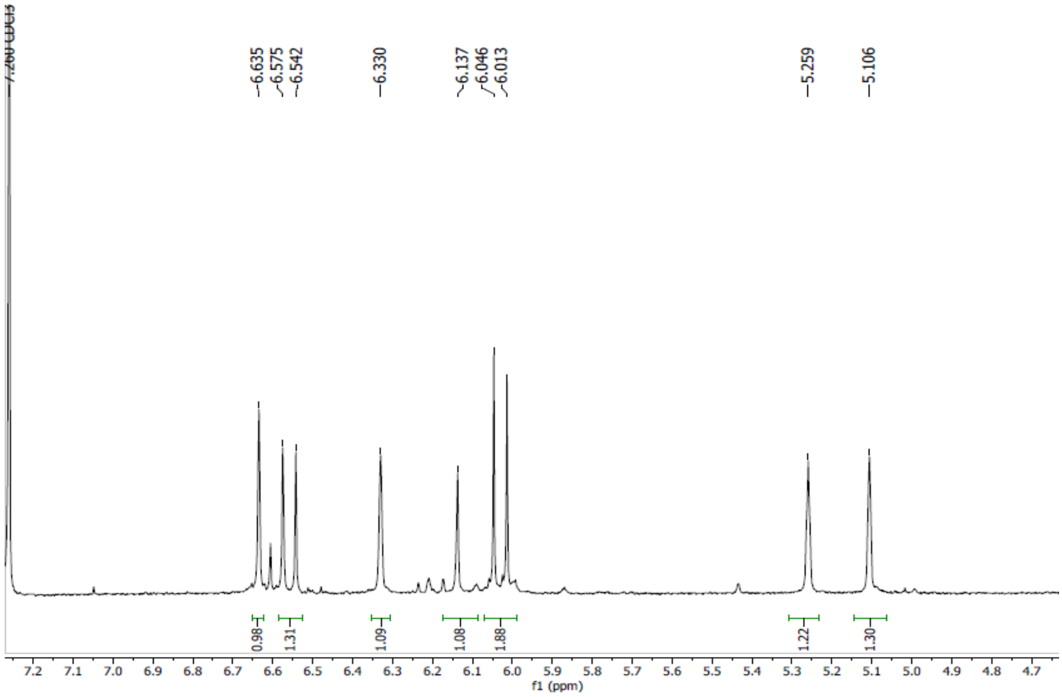

Figure S60. ^{13}C spectrum of Ribifolone E, 100MHz in $CDCl_3$

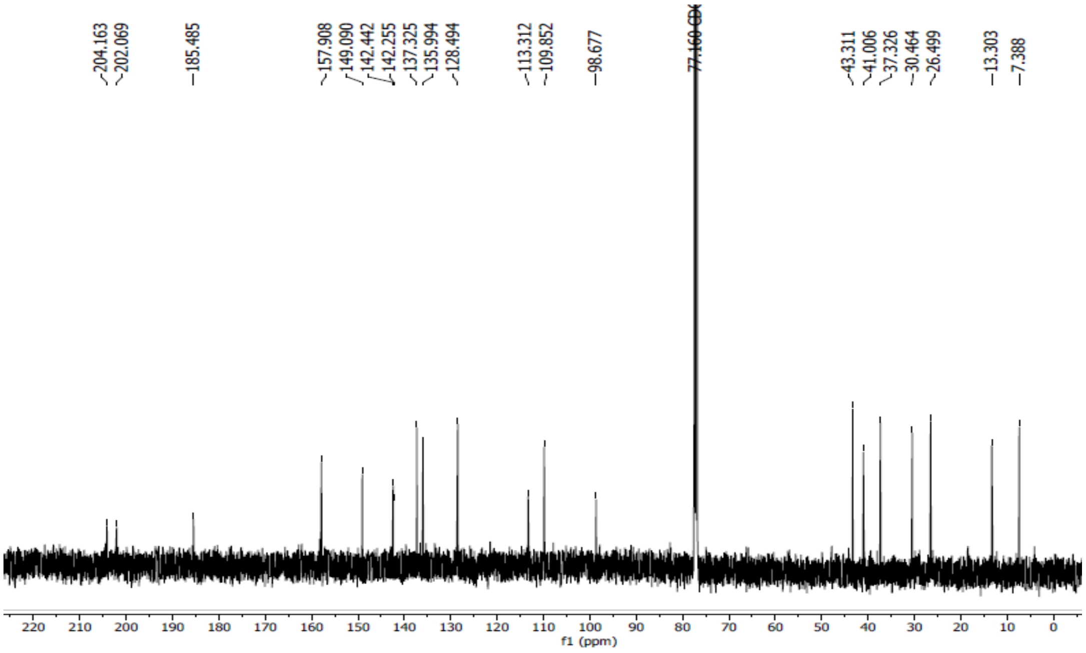

Figure S61. DEPT135 spectrum of Ribifolone E, 100MHz in $CDCl_3$.

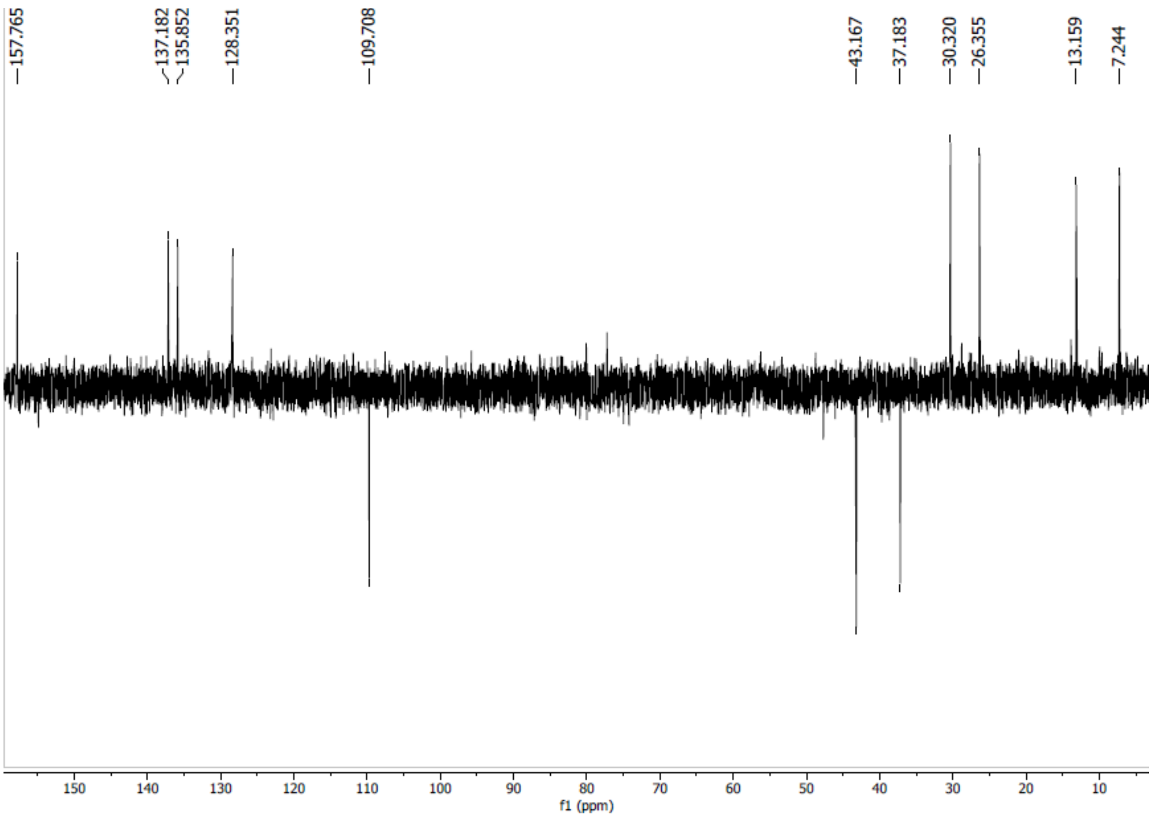

Figure S62. COSY spectrum of Ribifolone E, 400MHz in $CDCl_3$

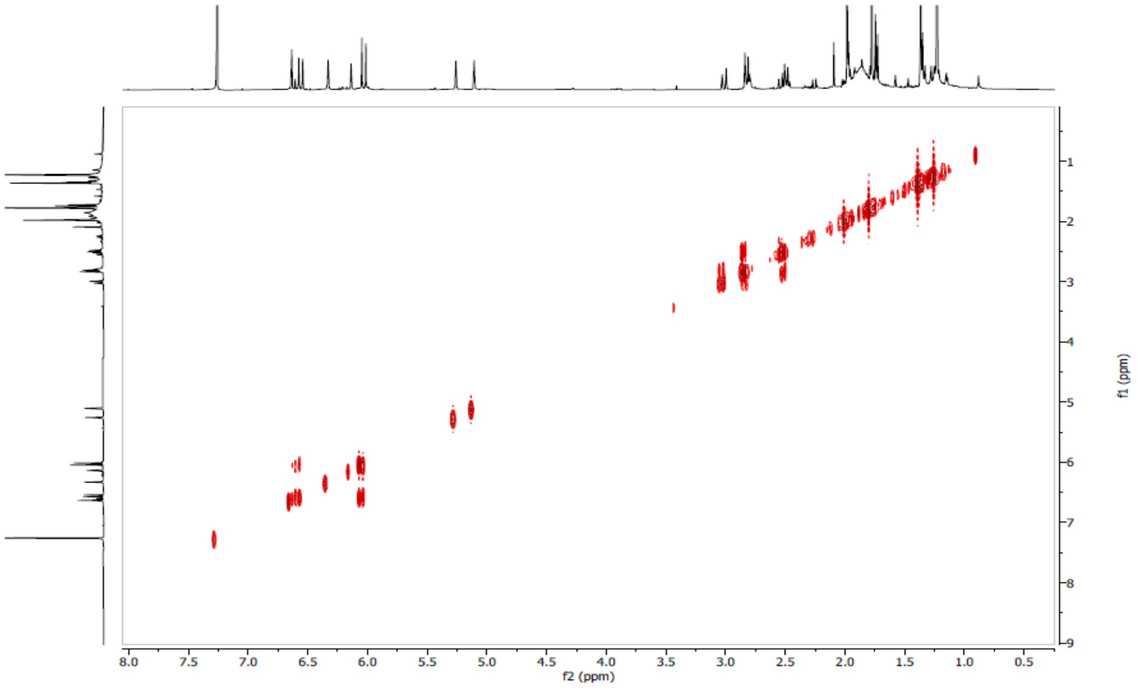

Figure S63. HMBC spectrum of Ribifolone E, 100MHz X 400MHz in $CDCl_3$.

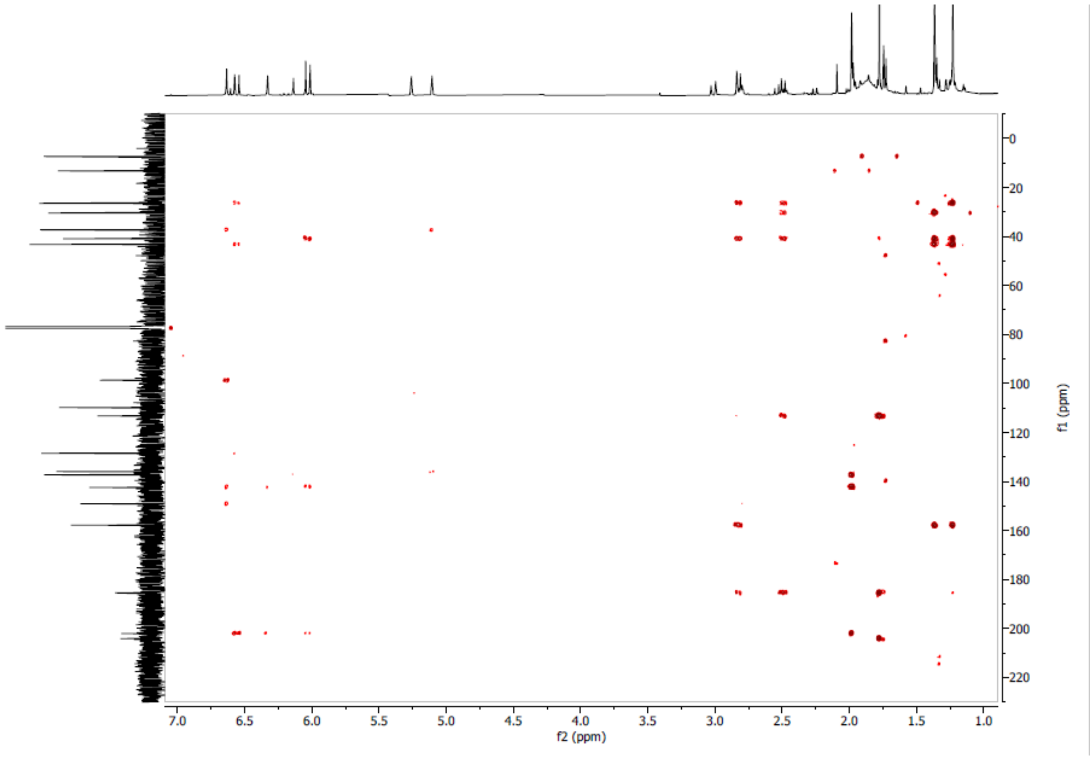

Figure S64. HSQC spectrum of Ribifolone E 100MHz X 400MHz in $CDCl_3$.

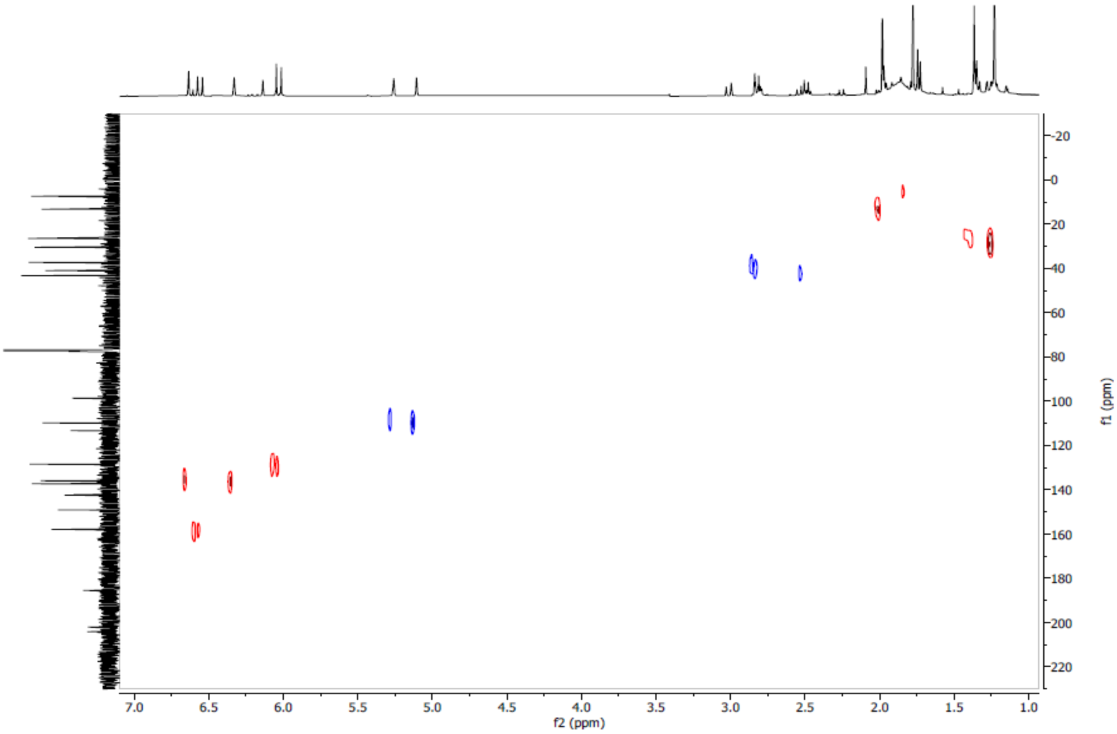

Figure S65. NOESY spectrum of Ribifolone E, 400MHz in $CDCl_3$.

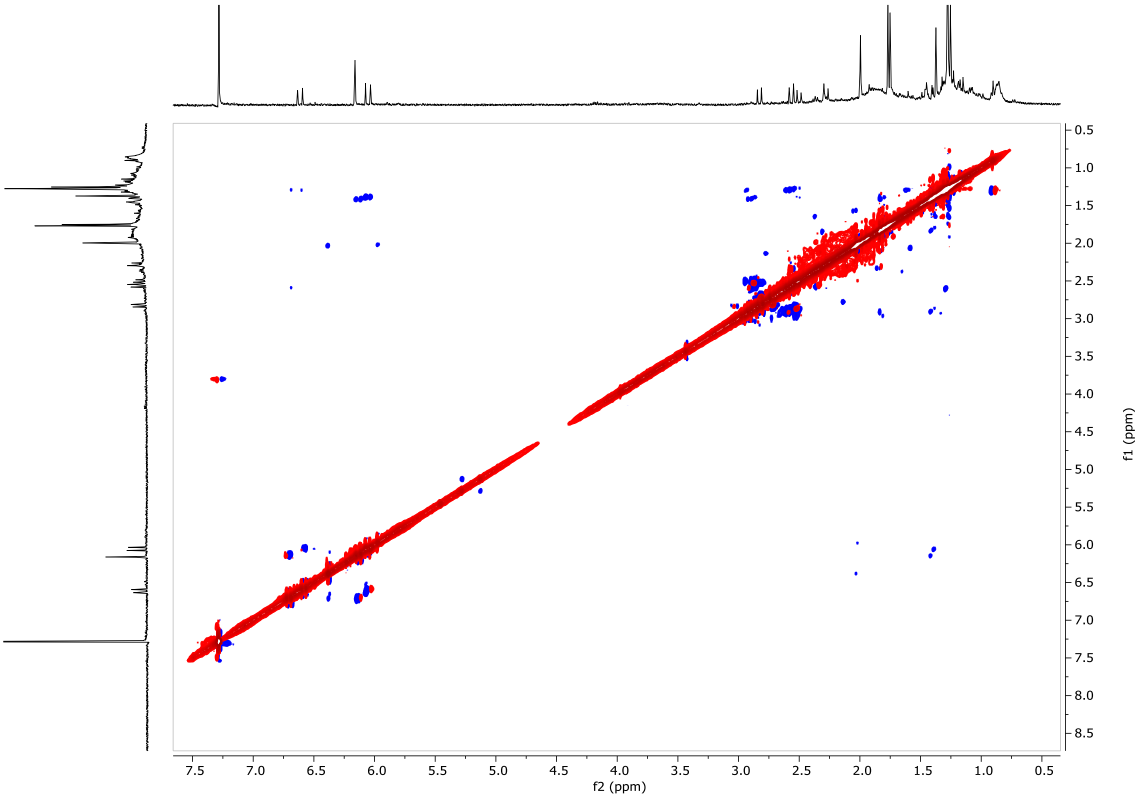

Figure S66. Infrared spectrum of Ribifolone E.

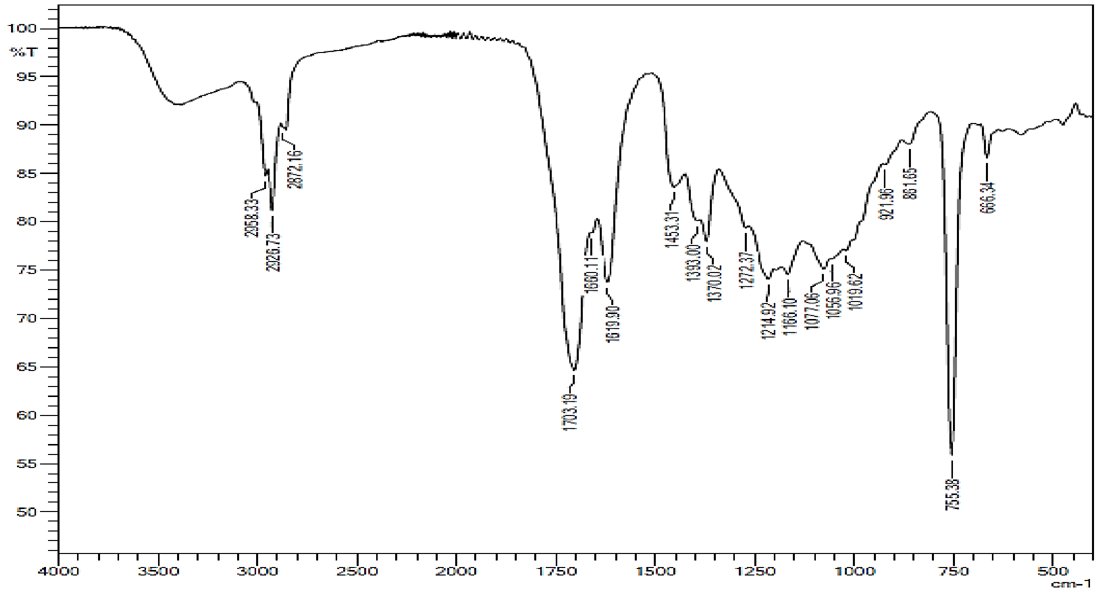

Figure S67. Ultraviolet (UV) spectrum of Ribifolone E.

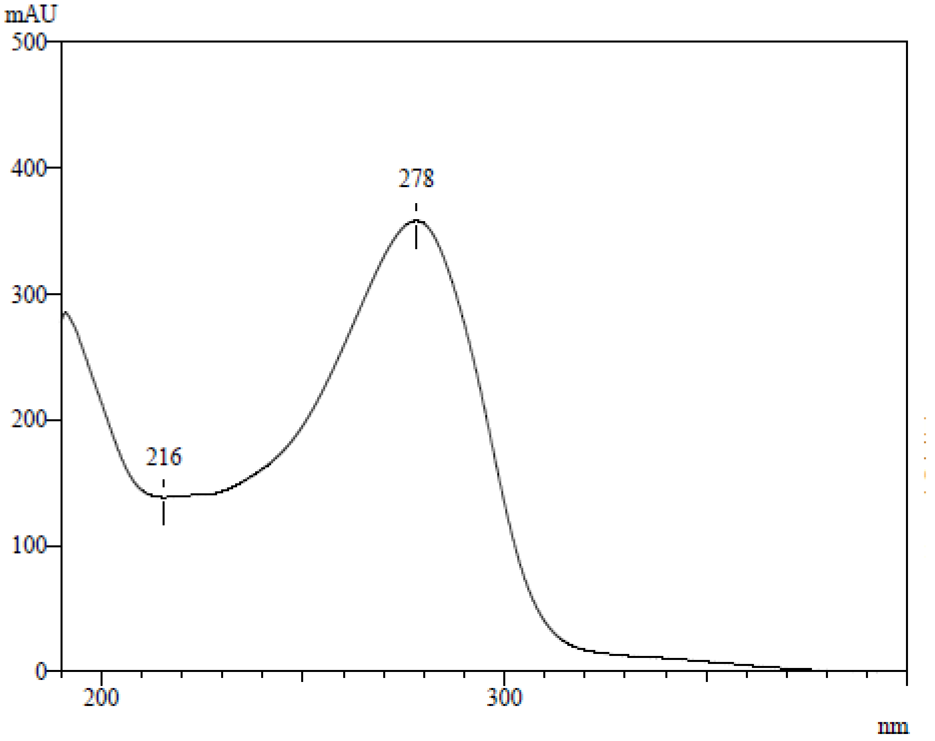

Figure S68. HR-ESI-MS spectrum of Ribifolone F.

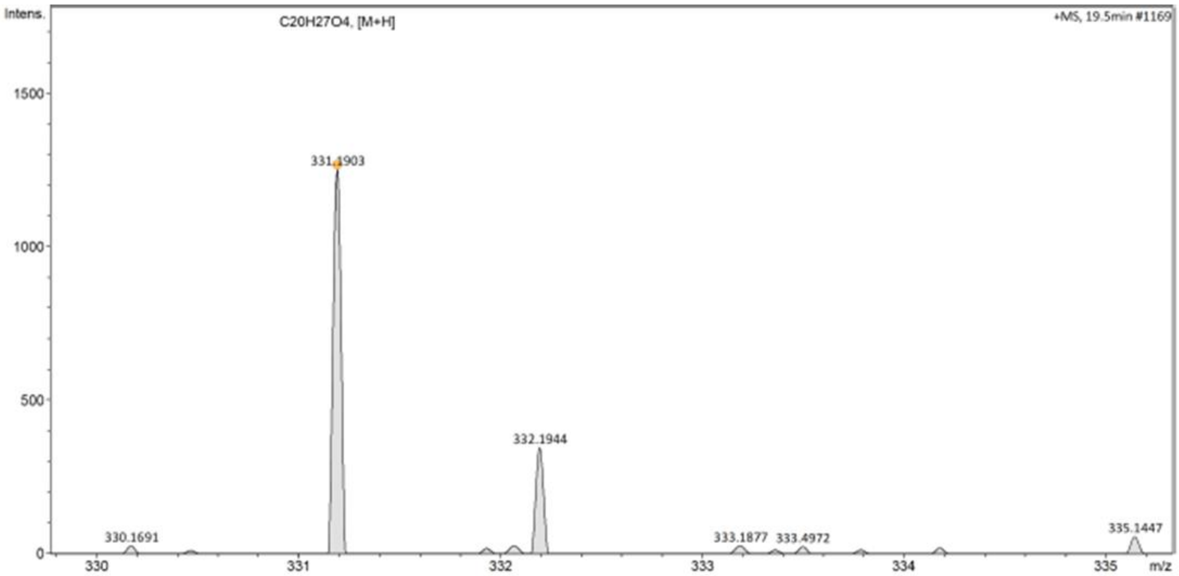

Meas. m/z	#	Ion Formula	m/z	err [ppm]	mSigma	# mSigma	Score	rdb	e⁻ Conf	N-Rule
331.1903	1	C20H27O4	331.1904	0.1	32.6	1	100.00	7.5	even	ok
	1	C20H27O4	331.1904	0.1	32.6	1	100.00	7.5	even	ok

Figure S69. ^{1}H spectrum of Ribifolone F, 400MHz in $CDCl_3$.

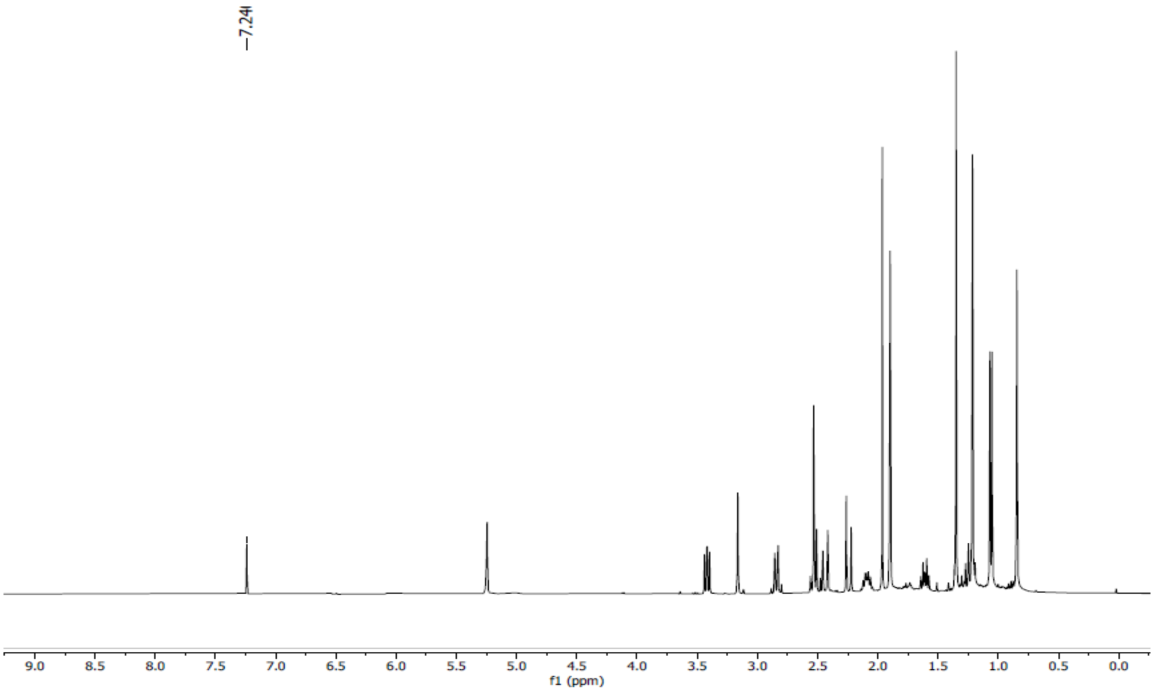

Figure S70. Expansion of ^{1}H spectrum of Ribifolone F in $CDCl_3$

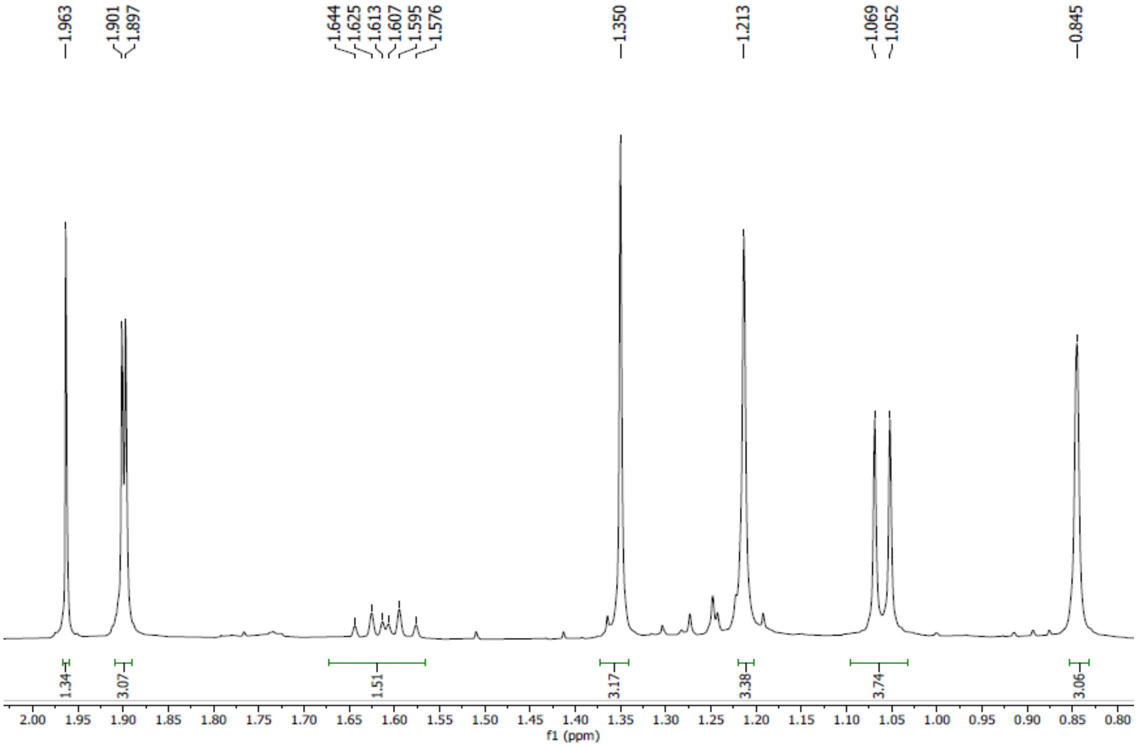

Figure S71. ^{13}C spectrum of Ribifolone F, 100MHz in $CDCl_3$

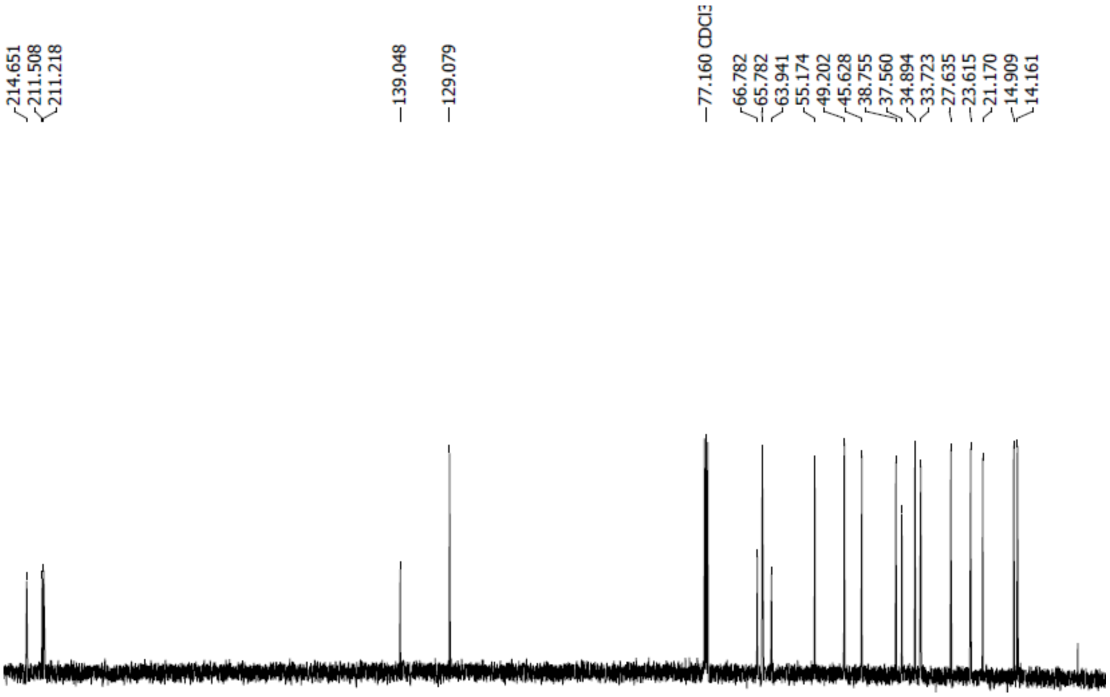

Figure S72. DEPT135 spectrum of Ribifolone F, 100MHz in $CDCl_3$

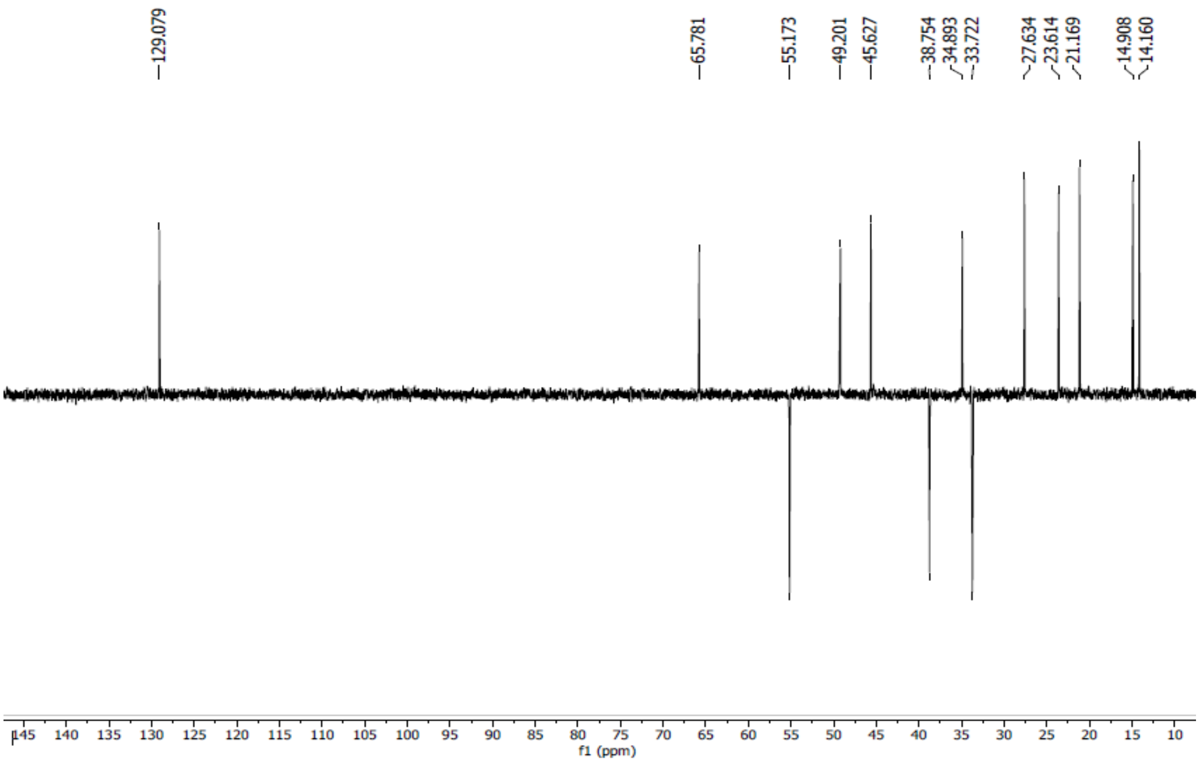

Figure S73. COSY spectrum of Ribifolone F, 400MHz in $CDCl_3$

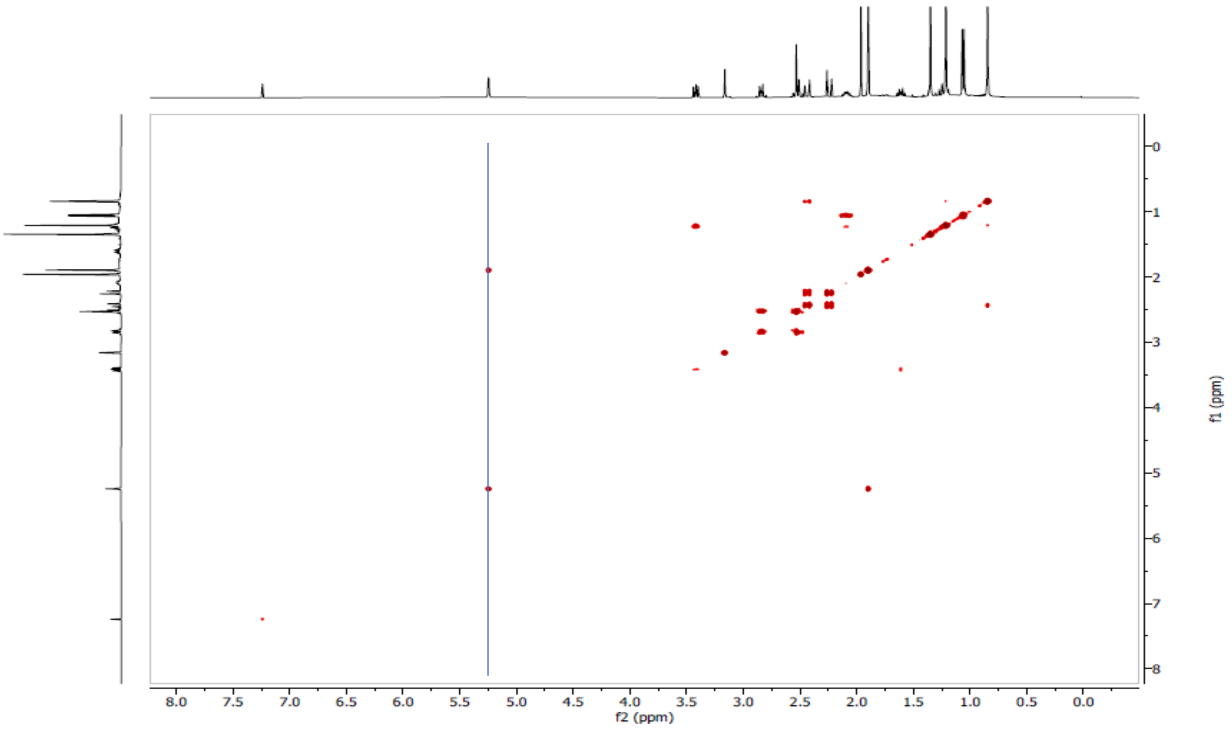

Figure S74. HMBC contour map of Ribifolone F, 400MHz in $CDCl_3$

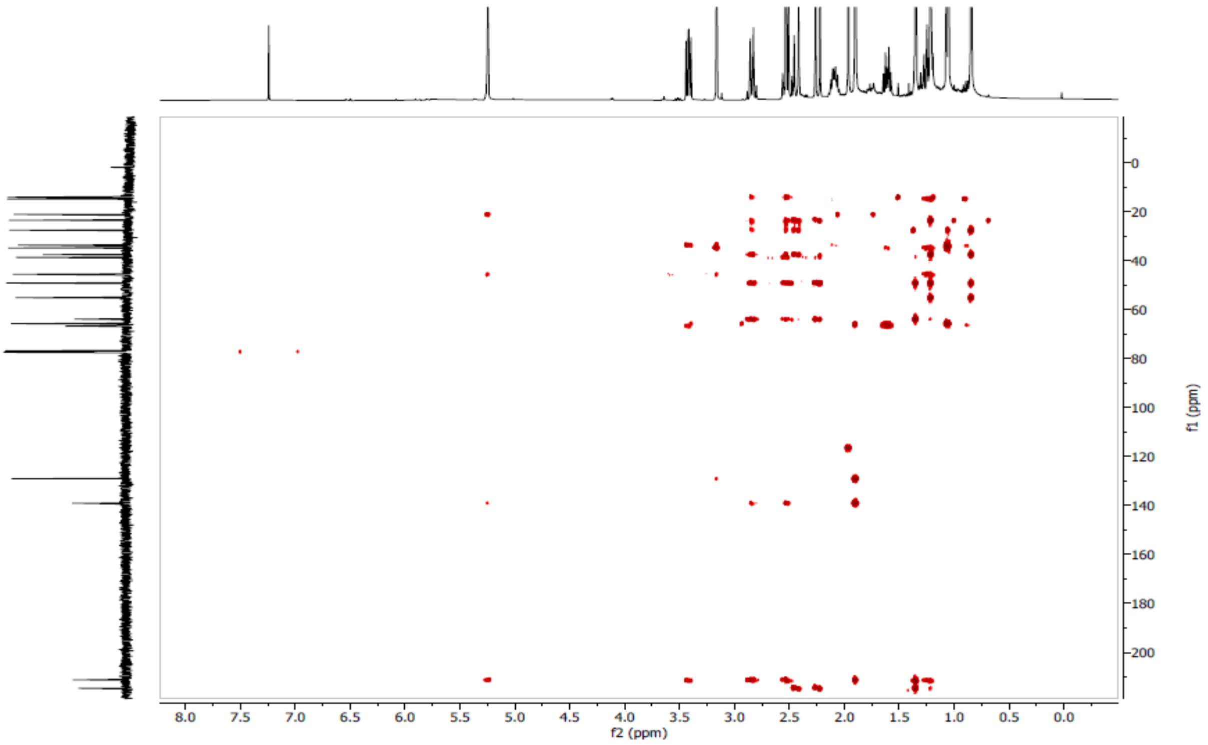

Figure S75. HSQC contour map of Ribifolone F, 400MHz in $CDCl_3$

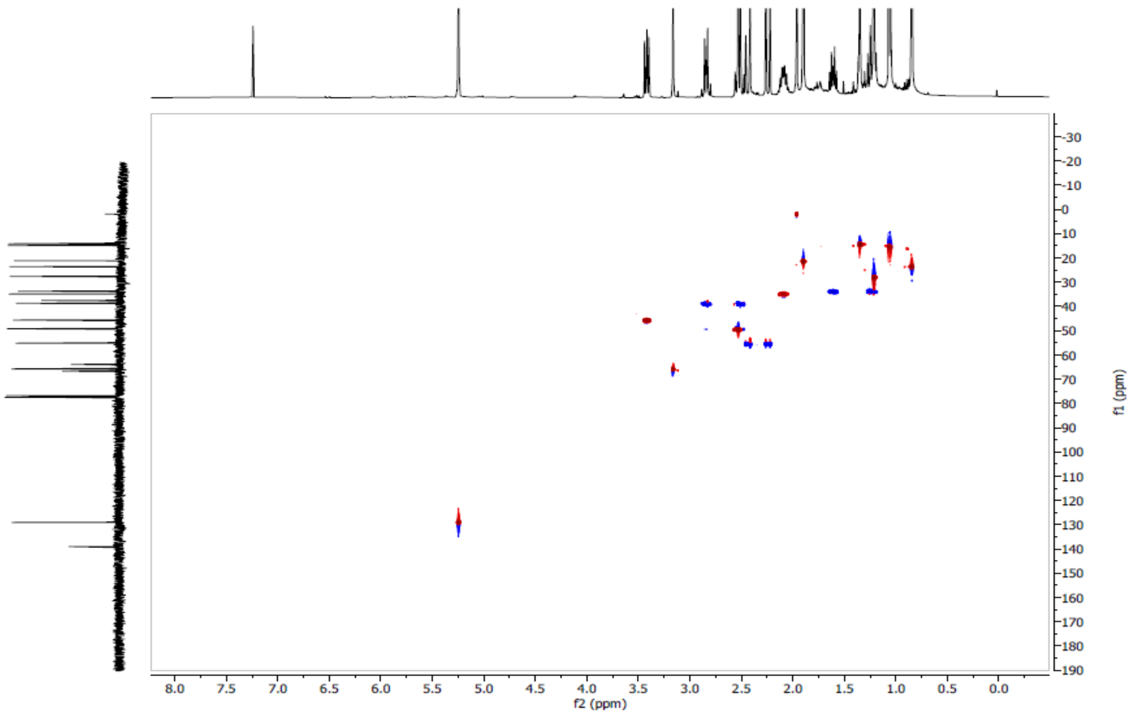

Figure S76. NOESY spectrum of Ribifolone F, 400MHz in $CDCl_3$

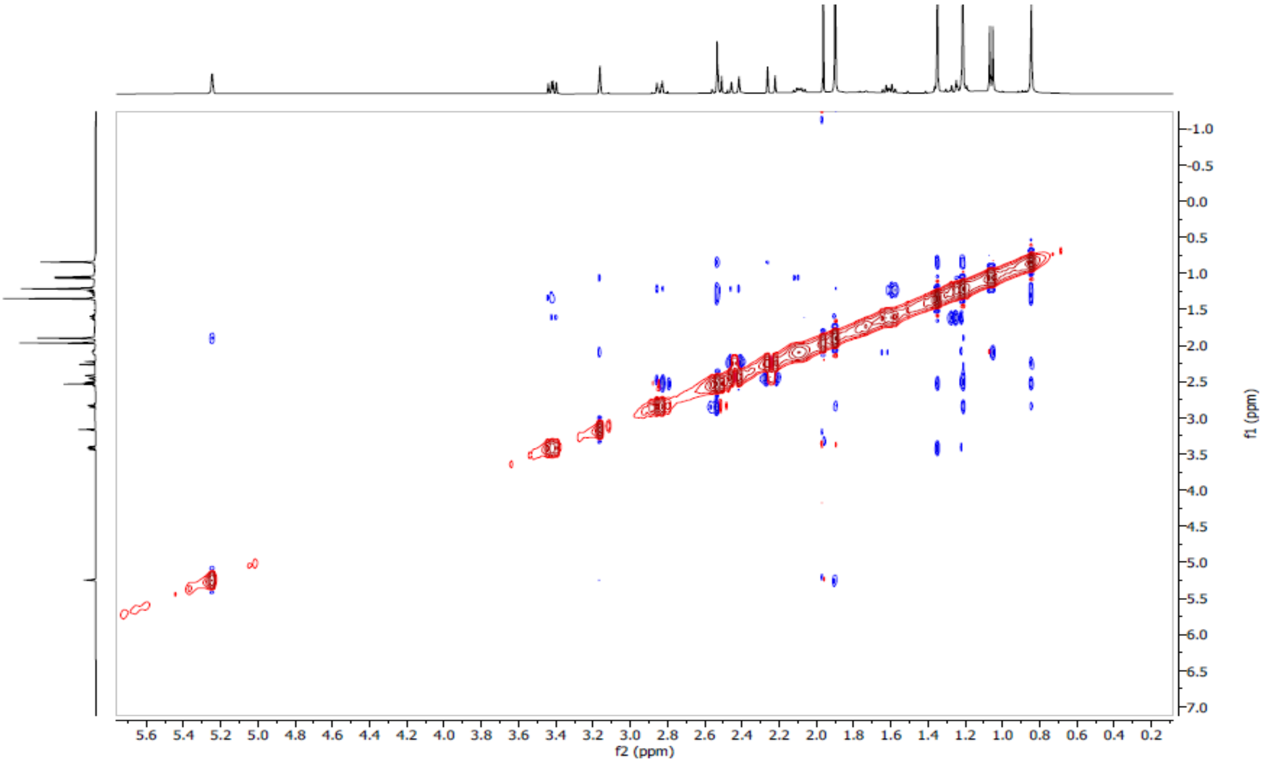

Figure S77. Infrared spectrum of Ribifolone F.

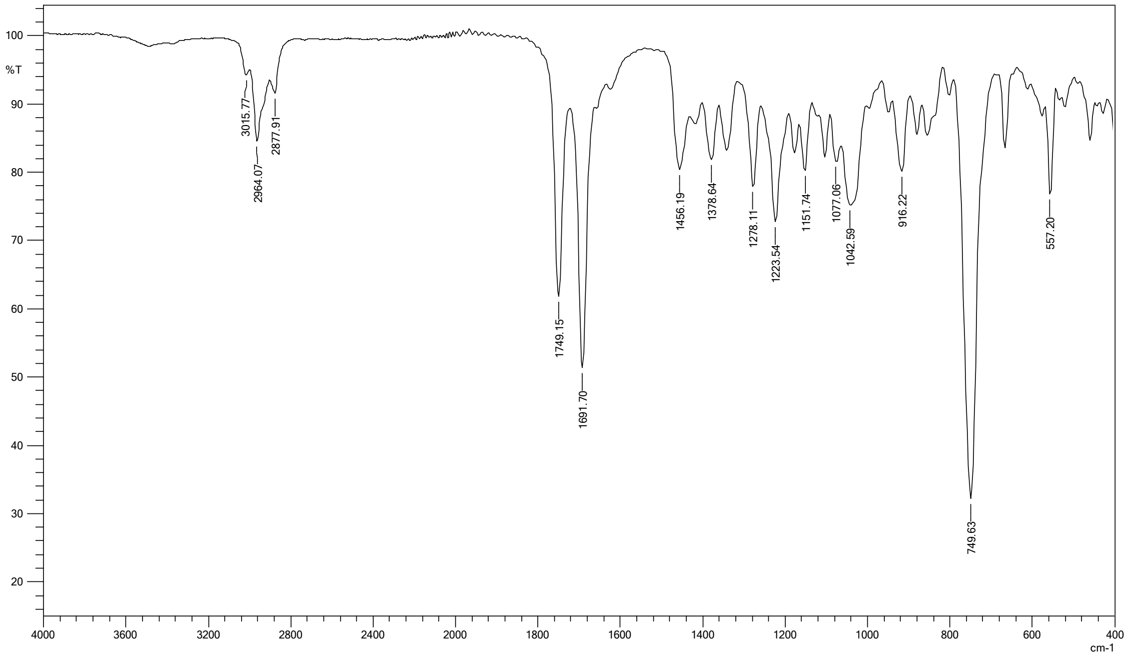

Figure S78. Ultraviolet (UV) spectrum of Ribifolone F.

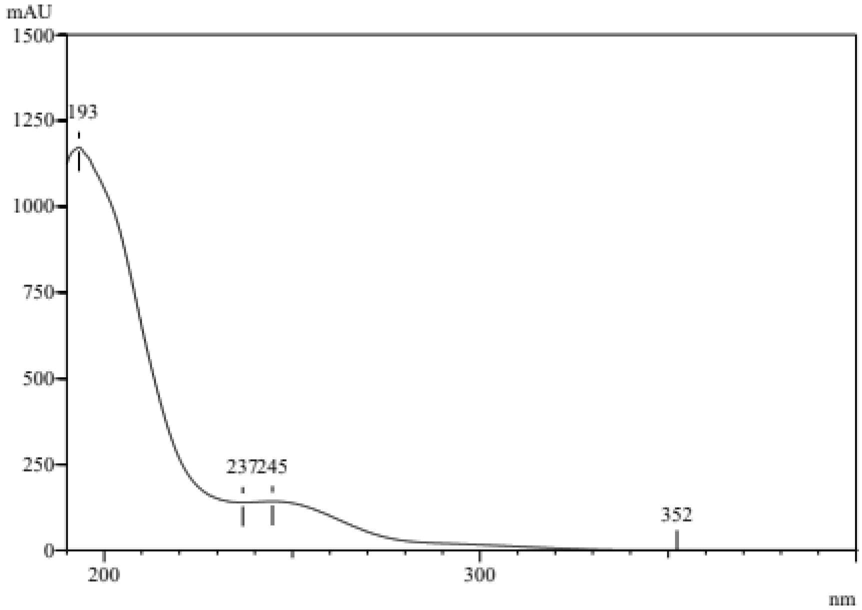

Figure S79. HR-ESI-MS spectrum of Ribifolone G.

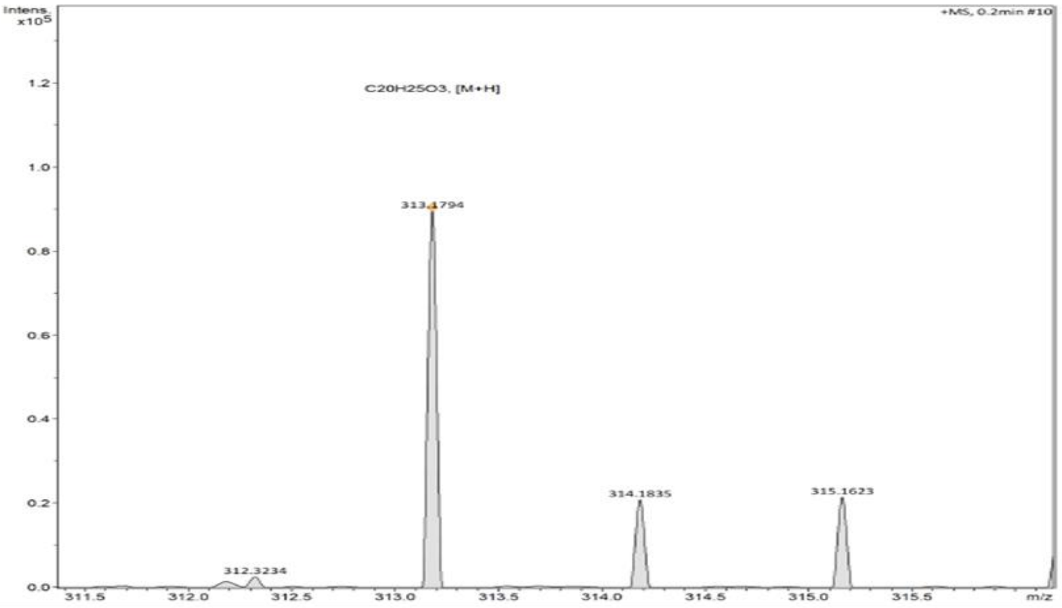

Meas. m/z	#	Ion Formula	m/z	err [ppm]	mSigma	# mSigma	Score	rdb	e⁻ Conf	N-Rule
313.1794	1	$C_{20}H_{25}O_3$	313.1798	1.4	18.2	1	100.00	8.5	even	ok

Figure S80. ^{1}H spectrum of Ribifolone G, 400MHz in $CDCl_3$

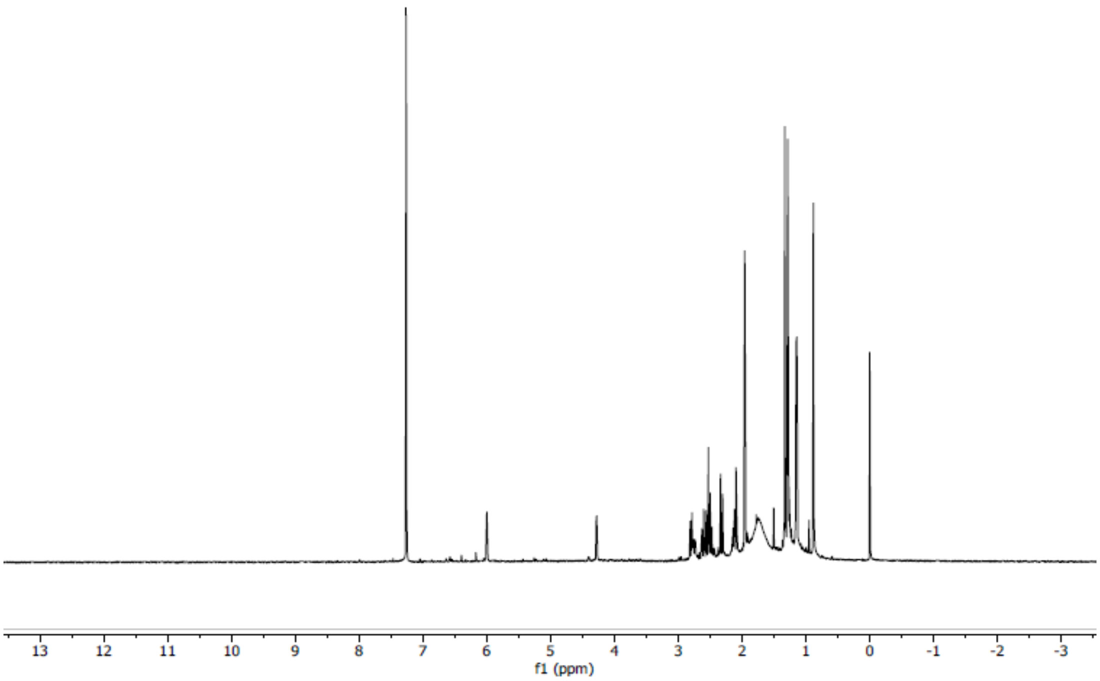

Figure S81. Expansion of ^{1}H spectrum of Ribifolone G in $CDCl_3$

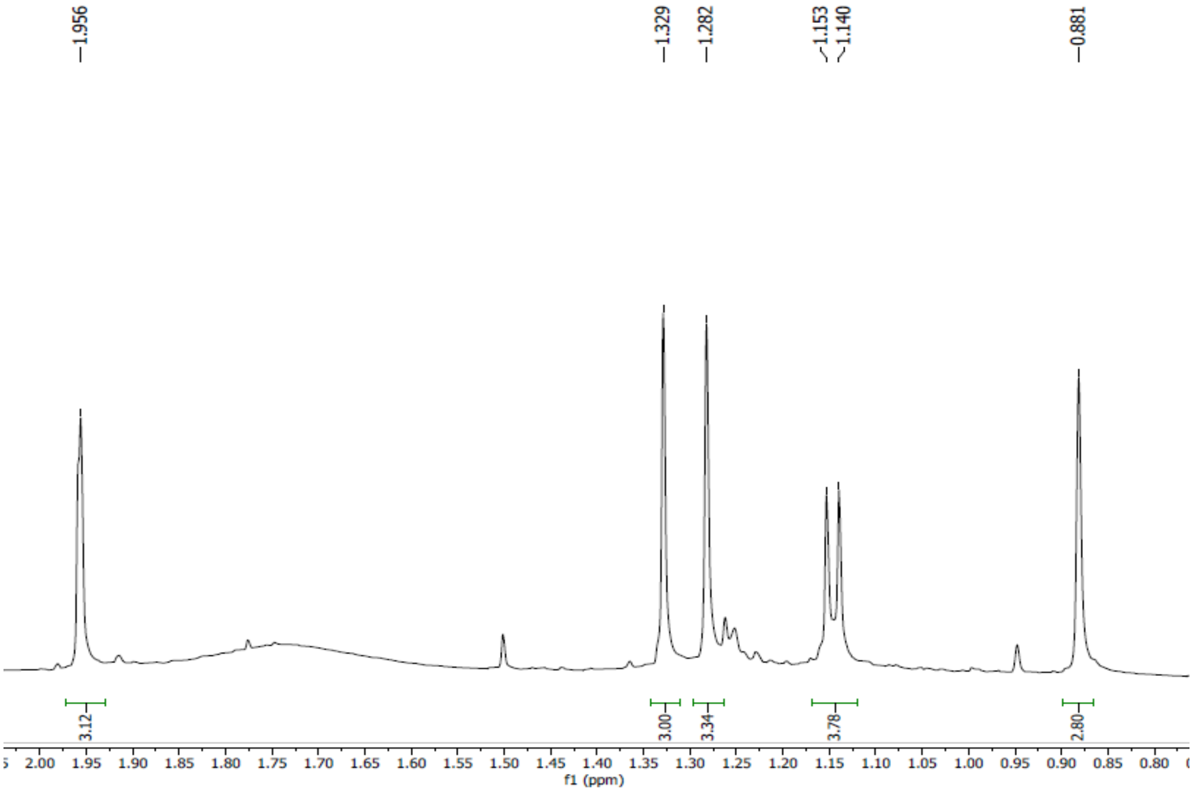

Figure S82. Expansion of ^{1}H spectrum of Ribifolone G in $CDCl_3$

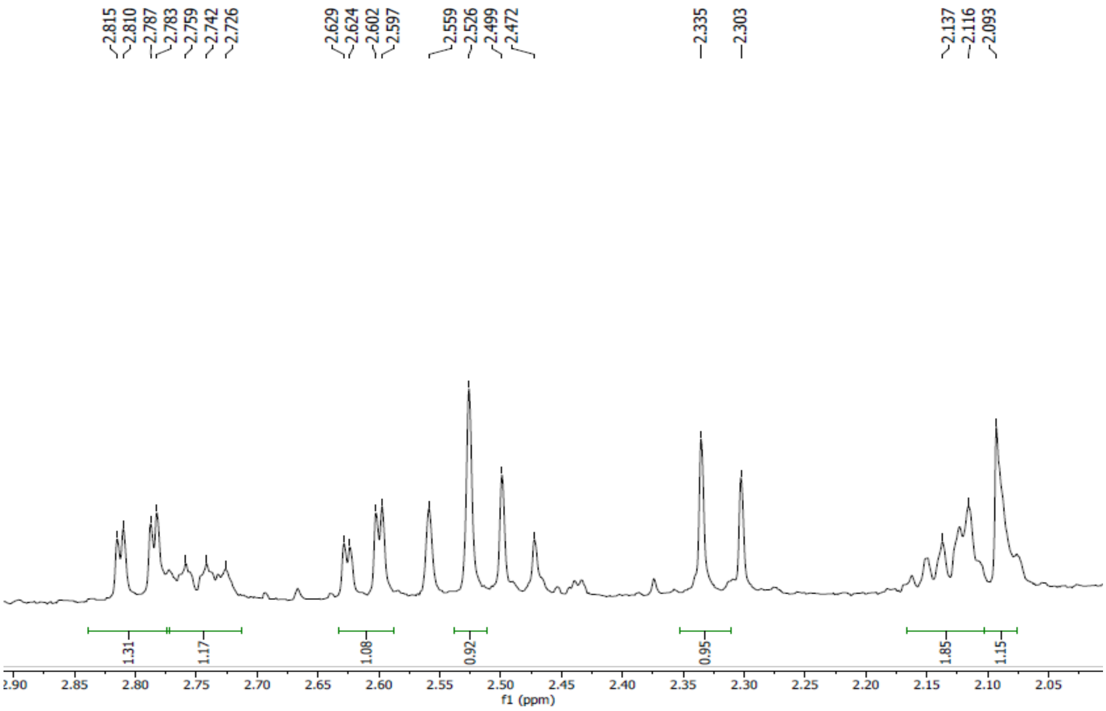

Figure S83. Expansion of ^{1}H spectrum of Ribifolone G in $CDCl_3$

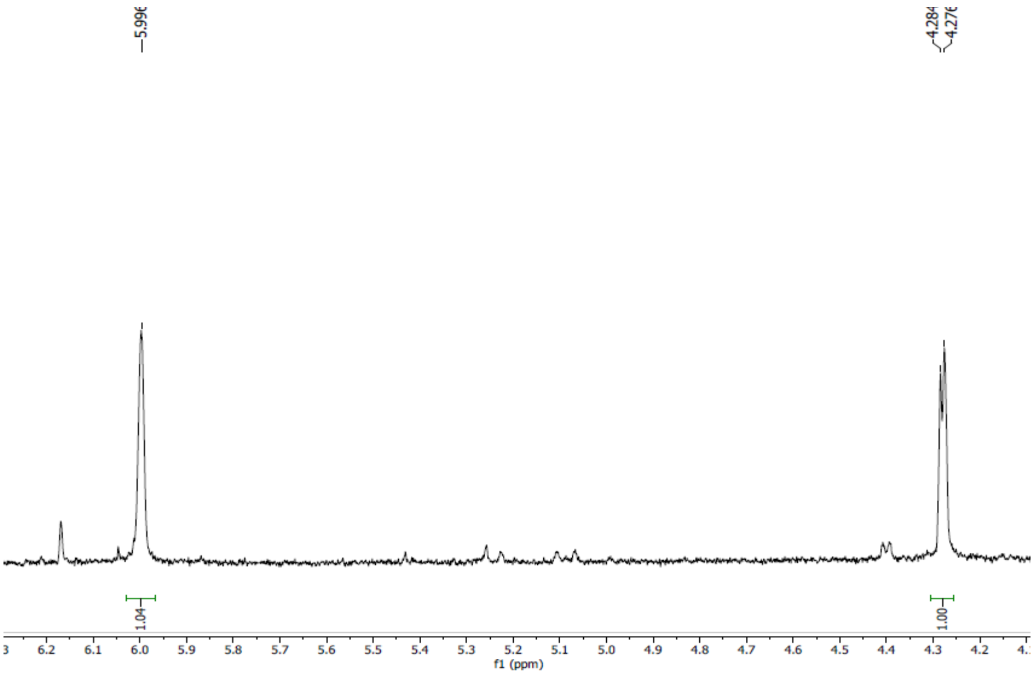

Figure S84. ^{13}C spectrum of Ribifolone G, 100MHz in $CDCl_3$.

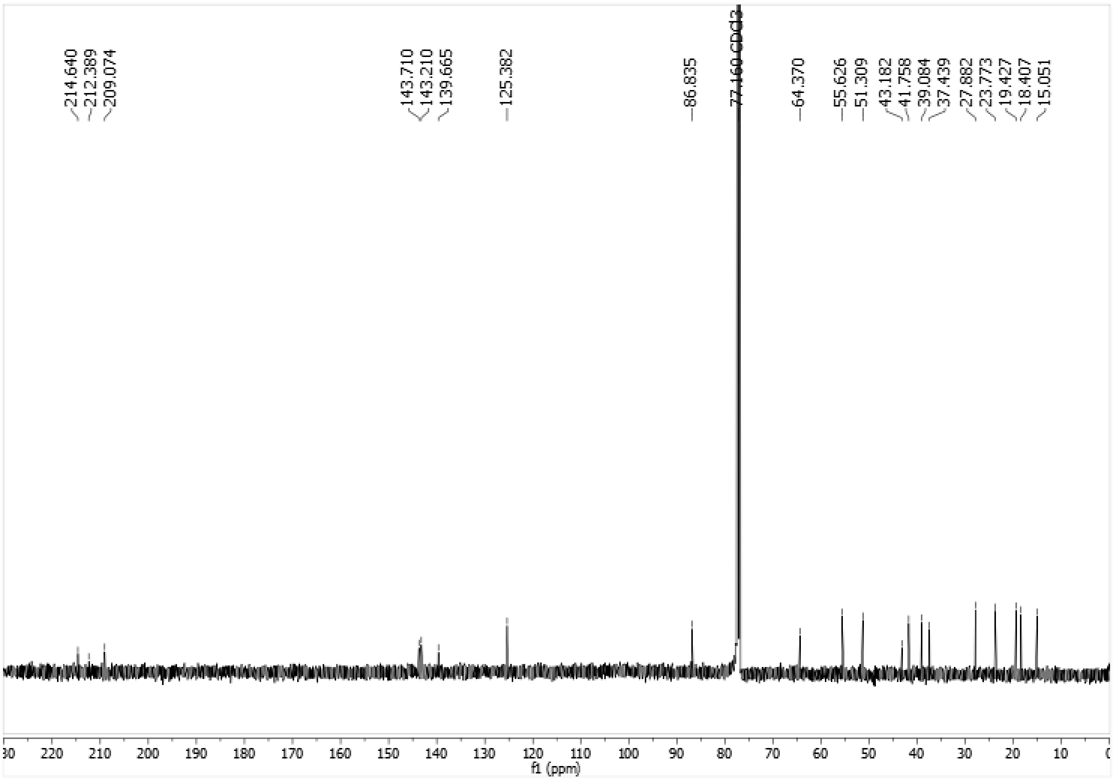

Figure S85. DEPT135 spectrum of Ribifolone G, 100MHz in $CDCl_3$

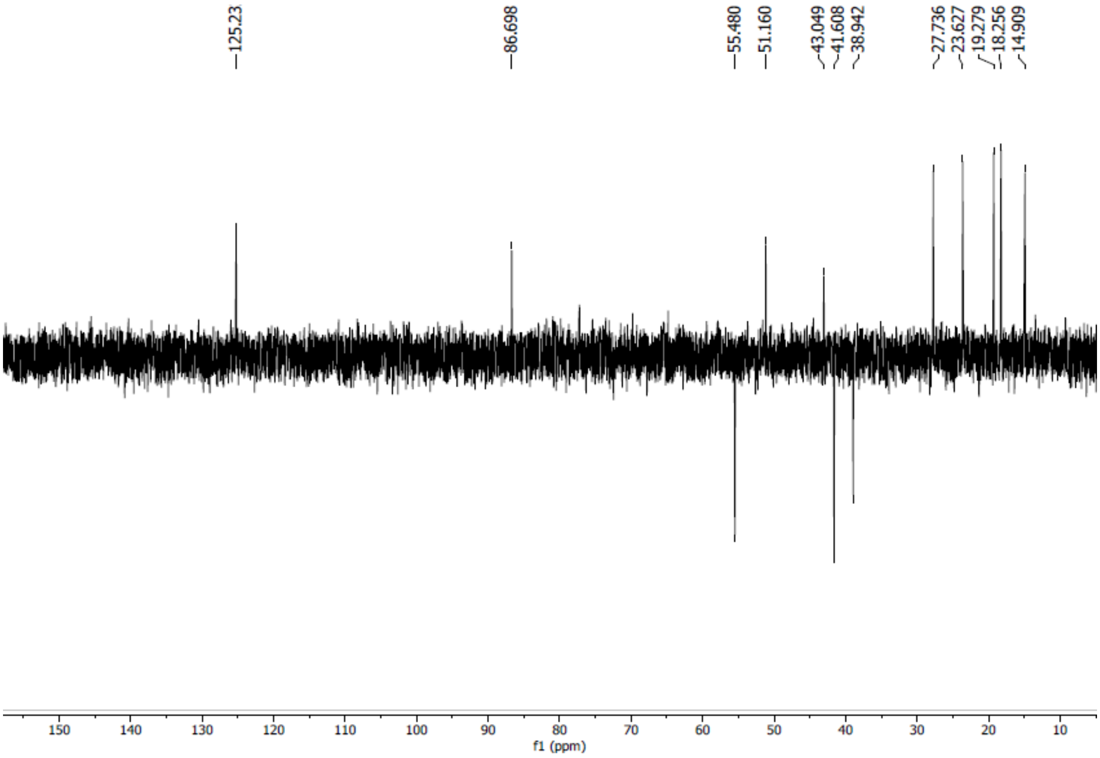

Figure S86. COSY spectrum of Ribifolone G, 400MHz in $CDCl_3$.

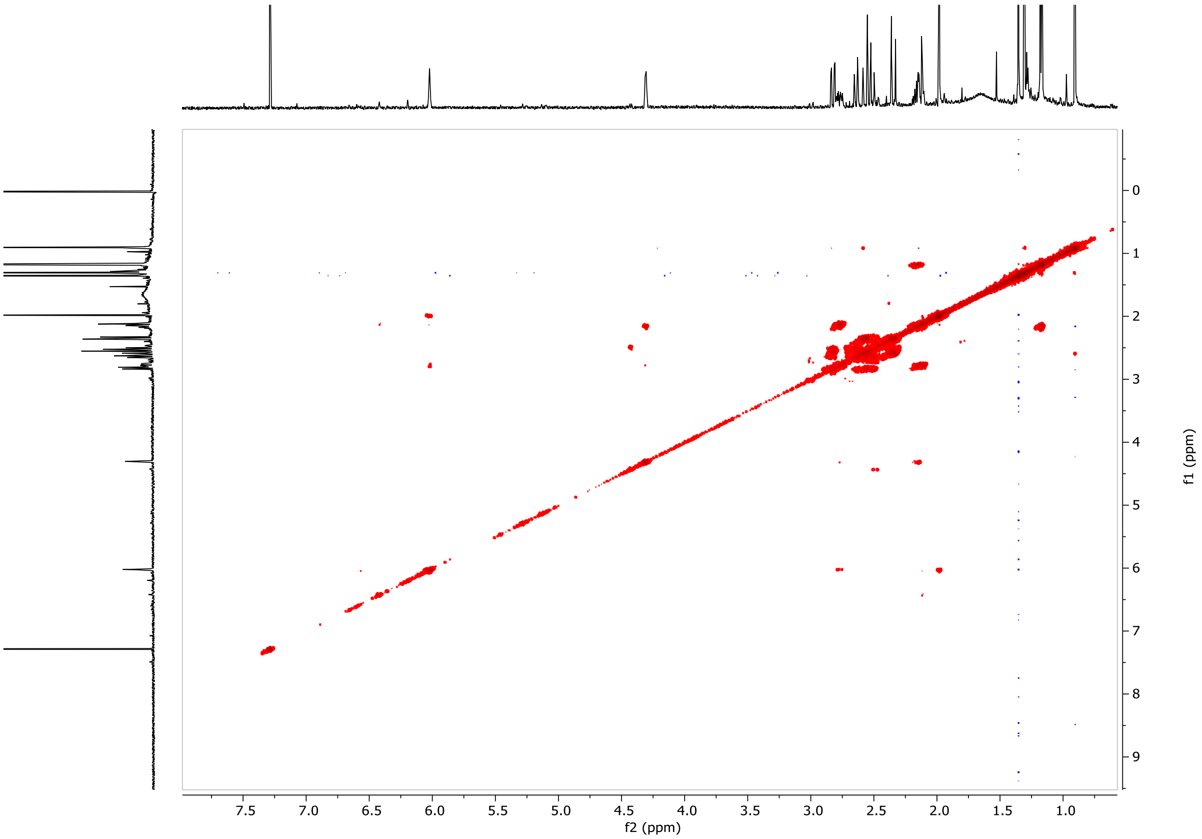

Figure S87. HMBC contour map of Ribifolone G, 100MHz X 400MHz in $CDCl_3$.

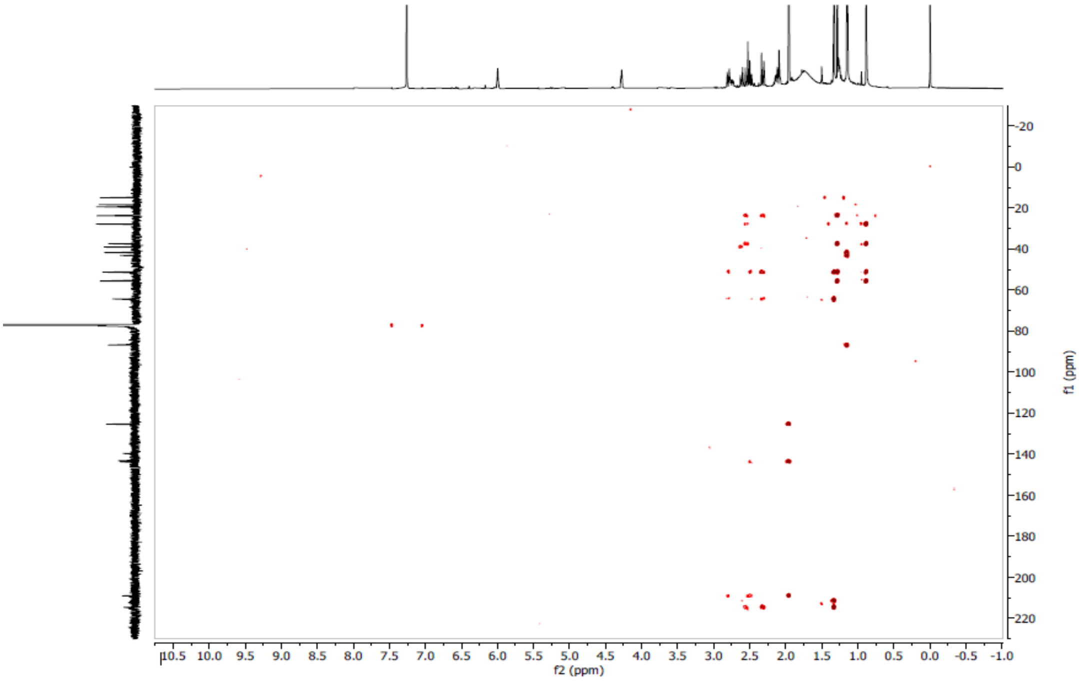

Figure S88. HMBC contour map expansion of Ribifolone G, 100 MHz x 400MHz in $CDCl_3$.

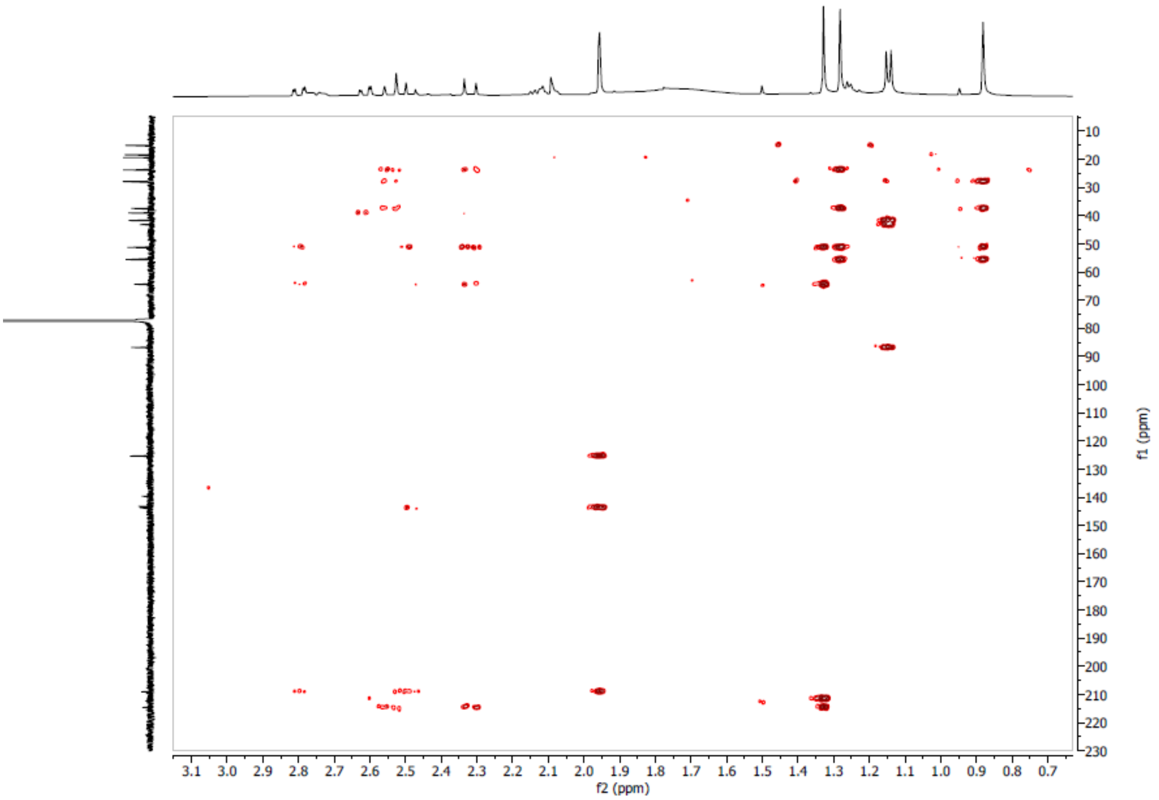

Figure S89. HSQC contour map of Ribifolone G, 100MHz X 400MHz in $CDCl_3$.

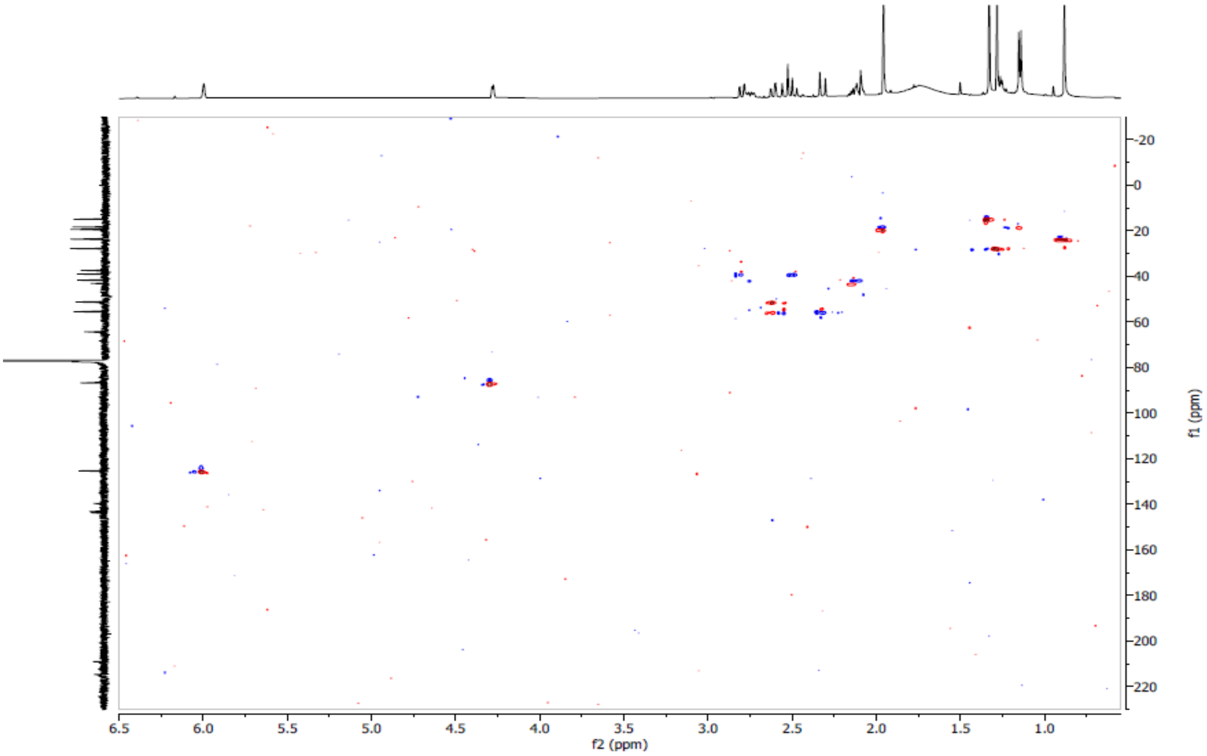

Figure S90. NOESY spectrum of Ribifolone G, 400MHz in $CDCl_3$.

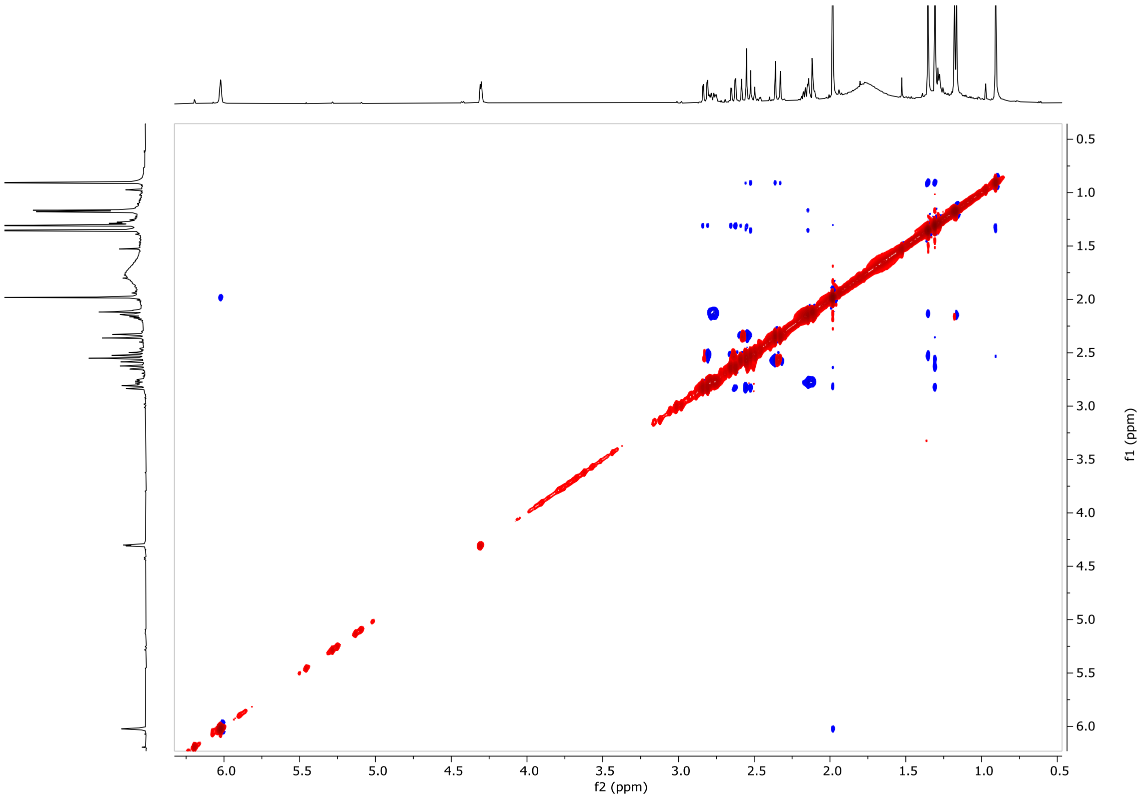

Figure S91. Infrared spectrum of Ribifolone G.

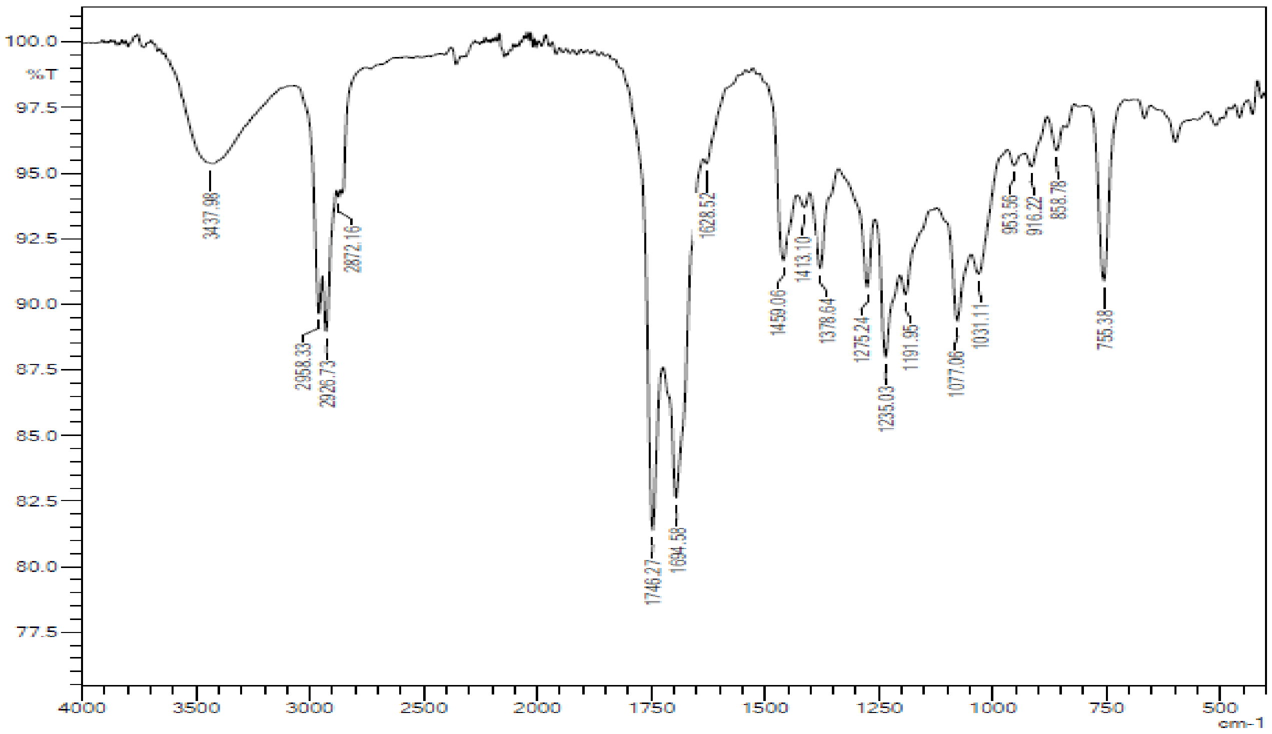

Figure S92. Ultraviolet (UV) spectrum of Ribifolone G.

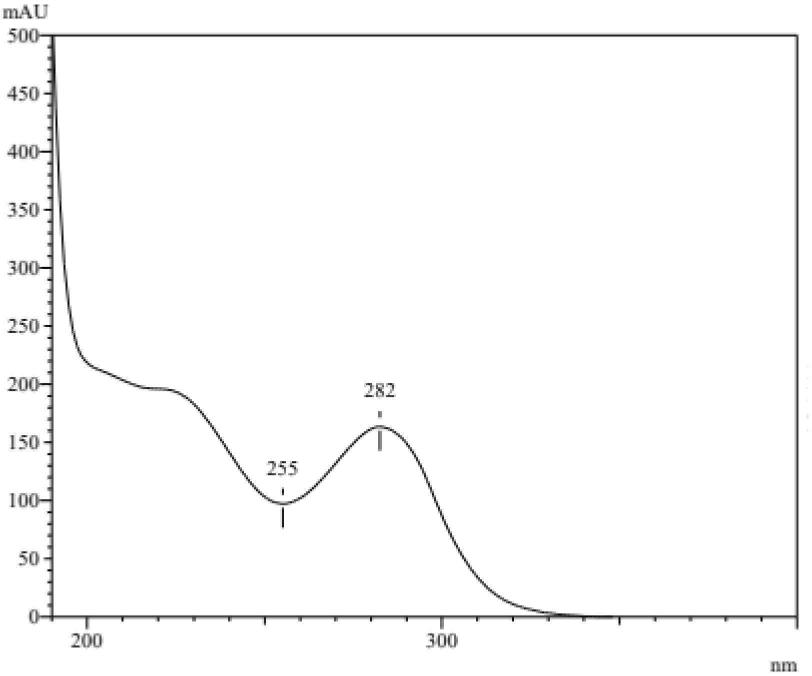

Figure S93. HR-ESI-MS spectrum of Ribifolone H.

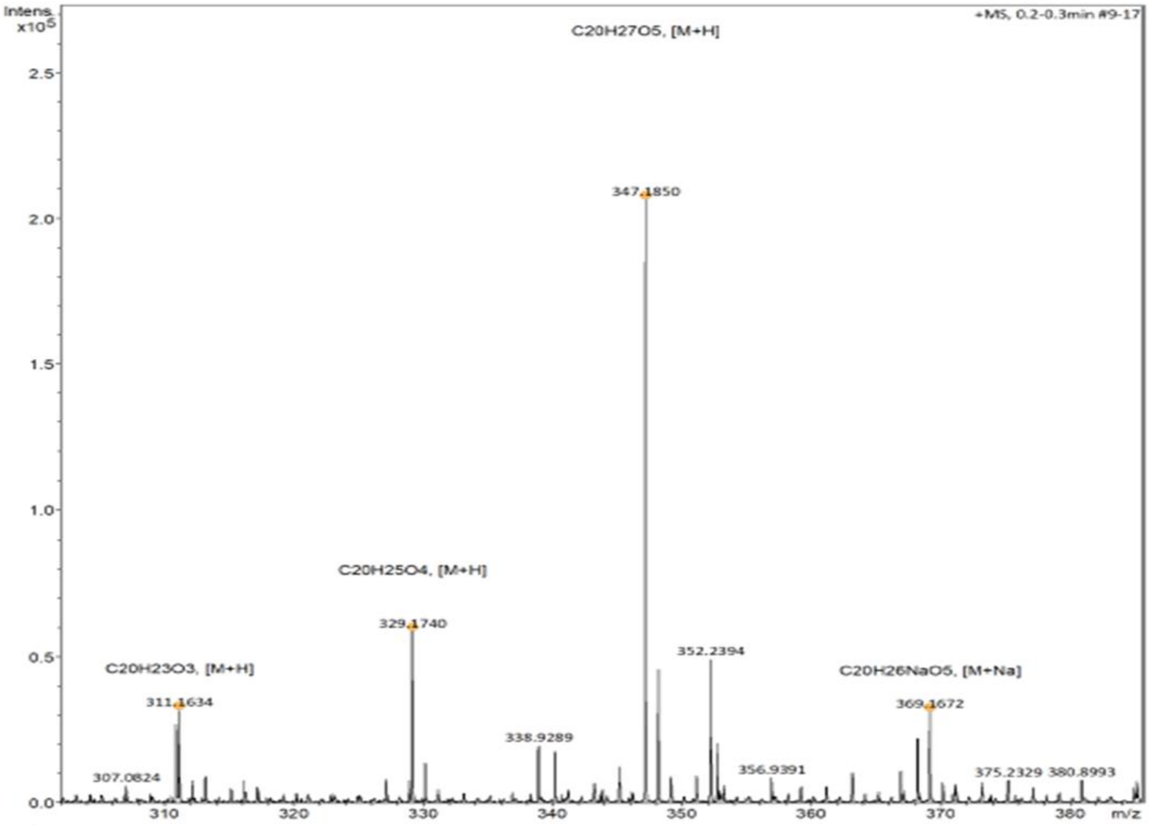

Meas. m/z	#	Ion Formula	m/z	err [ppm]	mSigma	# mSigma	Score	rdb	e⁻ Conf	N-Rule
311.1634	1	C20H23O3	311.1642	2.6	146.6	1	100.00	9.5	even	ok
329.1740	1	C20H25O4	329.1747	2.2	28.9	1	100.00	8.5	even	ok
347.1850	1	C20H27O5	347.1853	0.9	6.1	1	100.00	7.5	even	ok
369.1672	1	C20H26NaO5	369.1672	0.1	19.4	1	100.00	7.5	even	ok

Figure S94. ^{1}H spectrum of Ribifolone H, 400MHz in $CDCl_3$.

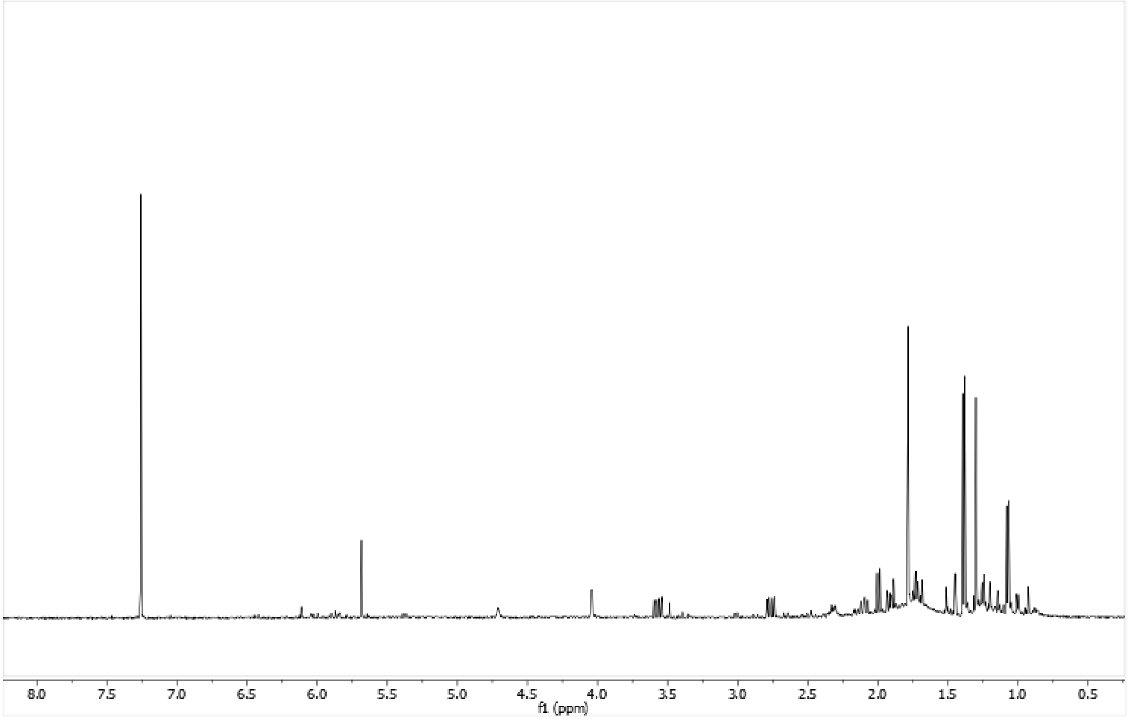

Figure S95. ^{1}H spectrum of Ribifolone H in $CDCl_3$.

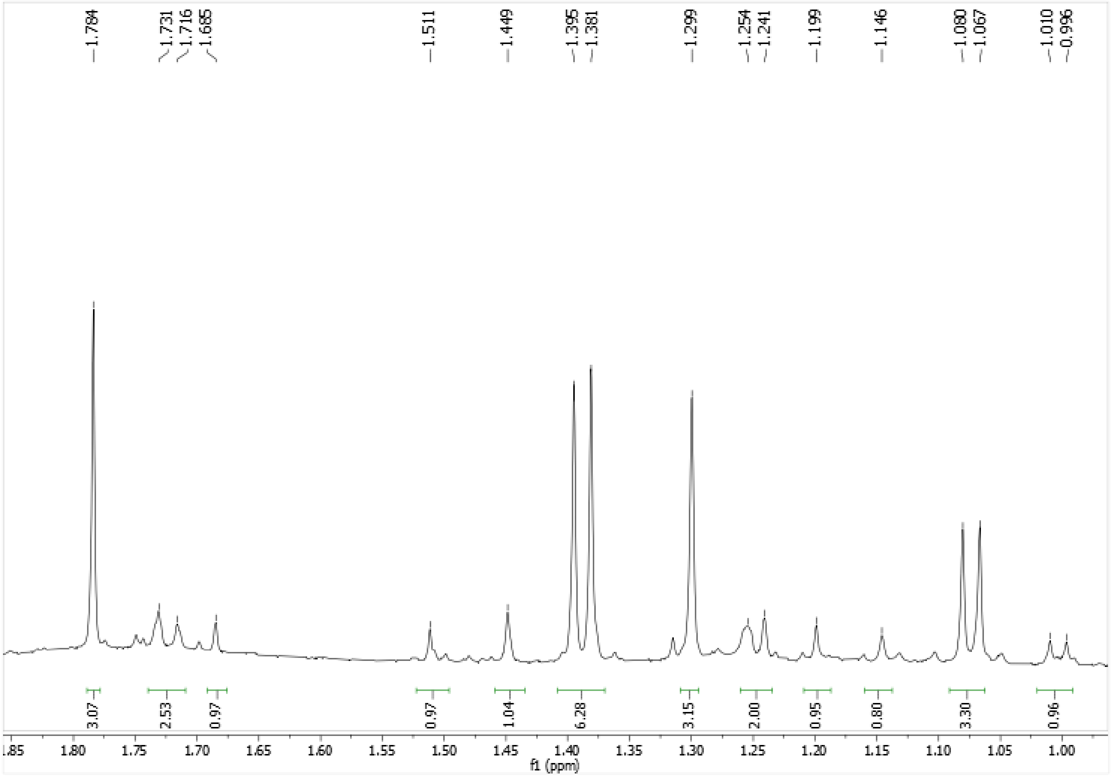

Figure S96. ^{1}H spectrum of Ribifolone H in $CDCl_3$.

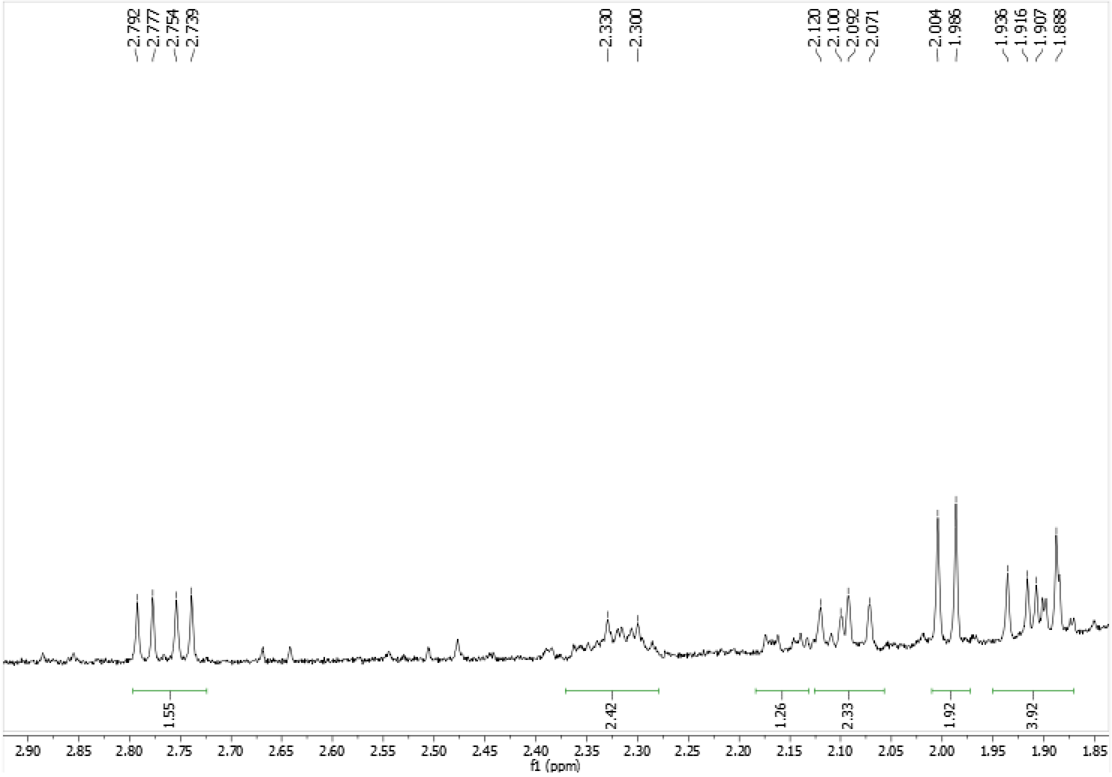

Figure S97. ^{1}H spectrum of Ribifolone H in $CDCl_3$.

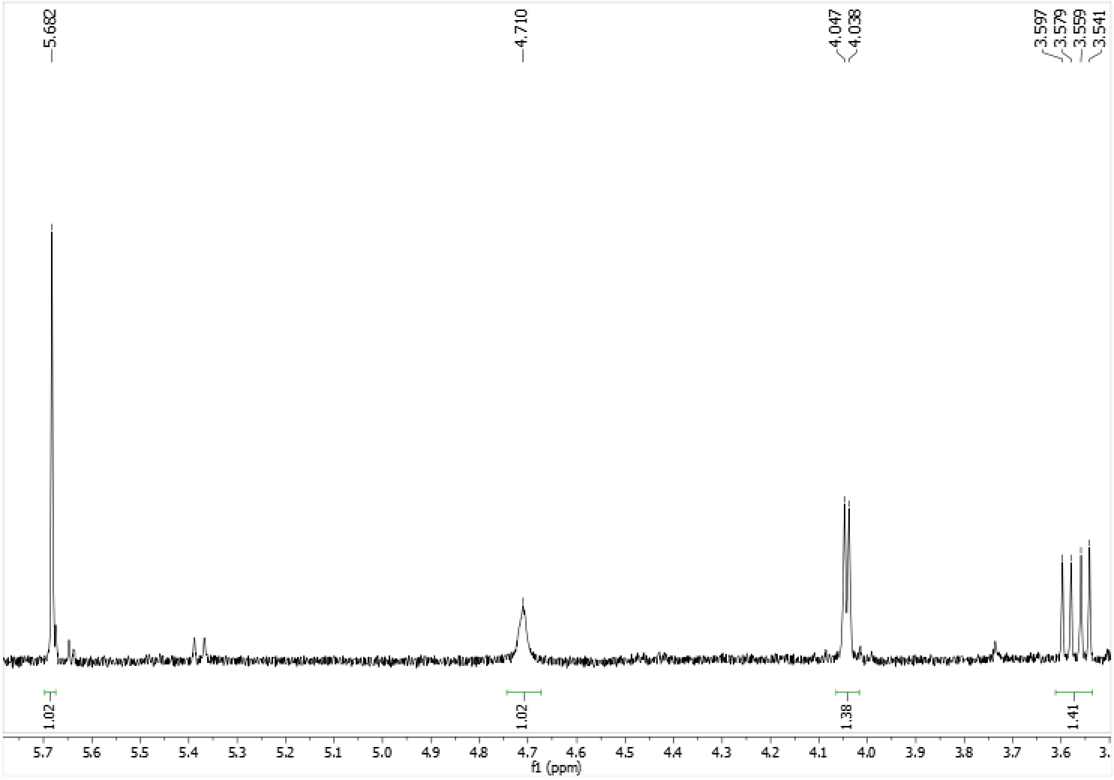

Figure S98. ^{13}C spectrum of Ribifolone H, 100 MHz in $CDCl_3$.

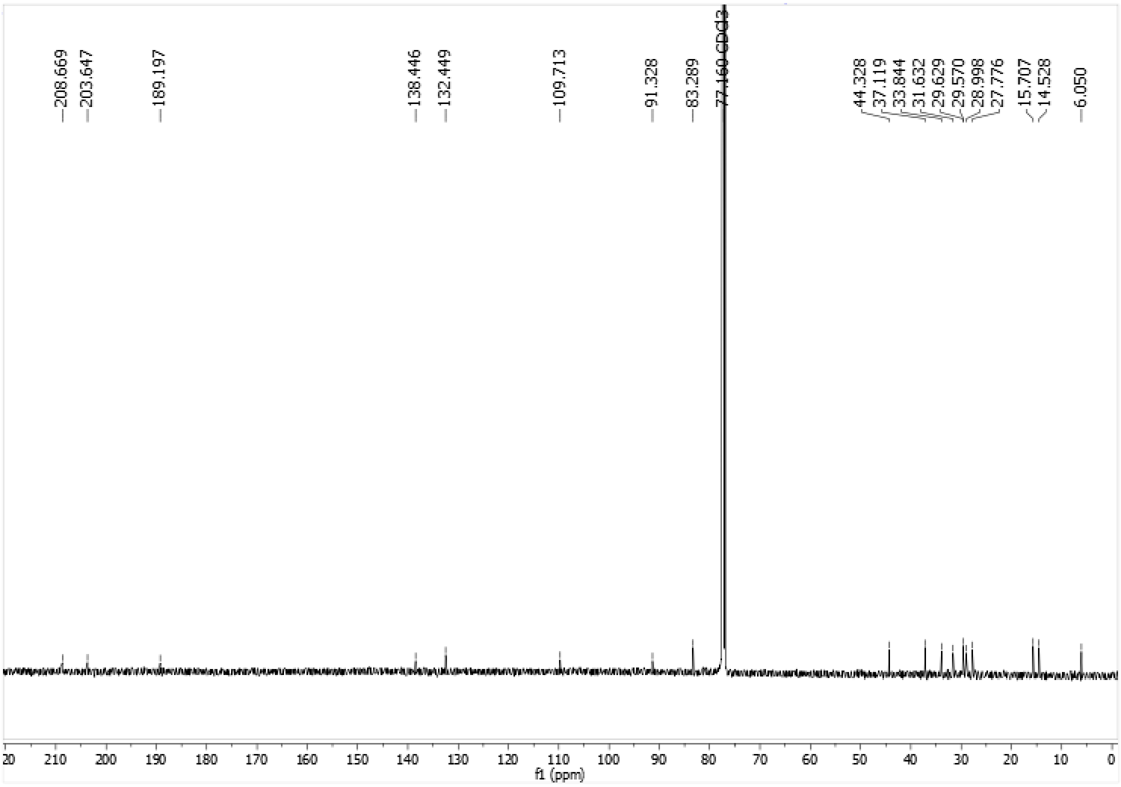

Figure S99. DEPT135135 spectrum of Ribifolone H, 100 MHz in $CDCl_3$.

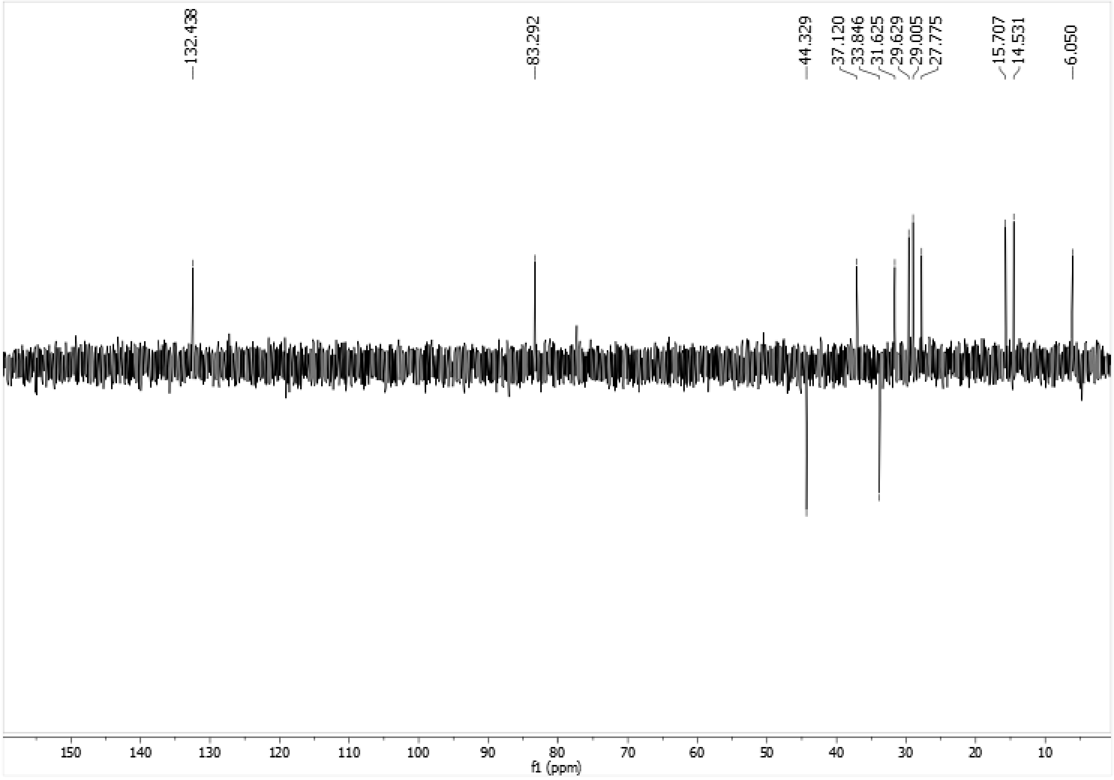

Figure S100. COSY spectrum of Ribifolone H, 400MHz in $CDCl_3$

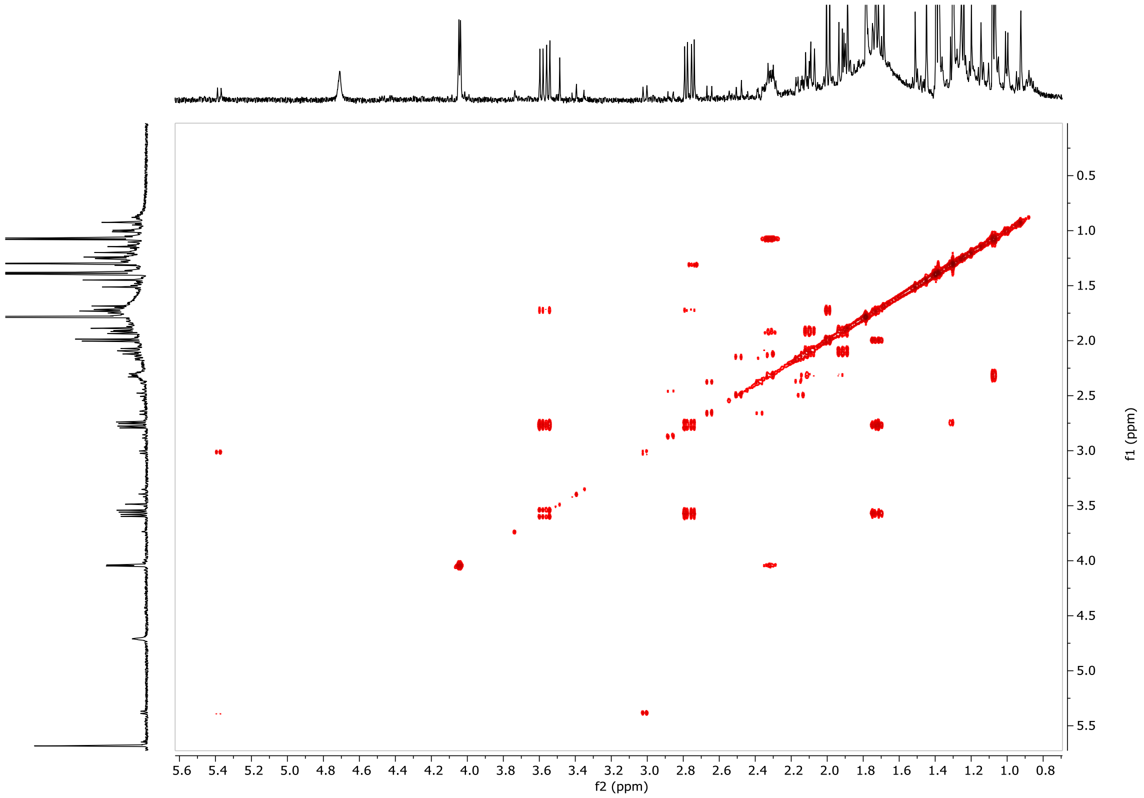

Figure S101. HMBC contour map of Ribifolone H, 100MHz x 400MHz in $CDCl_3$.

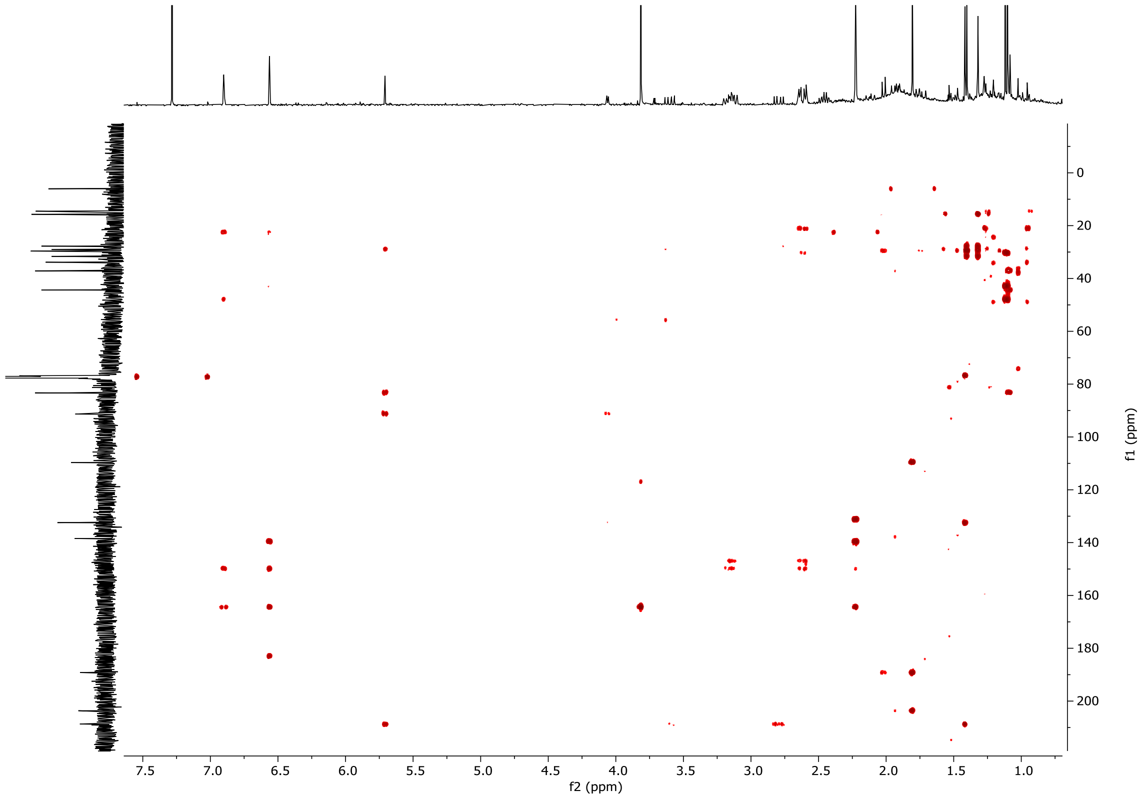

Figure S102. HMBC contour map expansion of Ribifolone H, in $CDCl_3$.

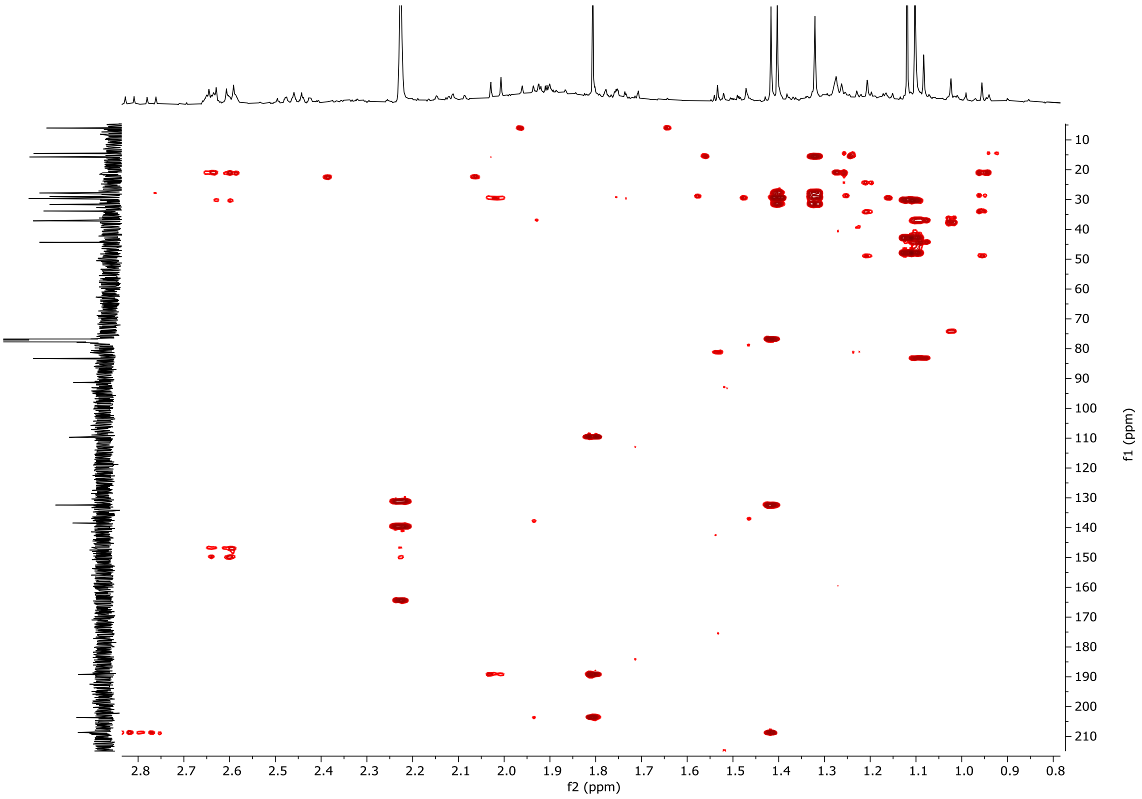

Figure S103. HMBC contour map expansion of Ribifolone H, in $CDCl_3$.

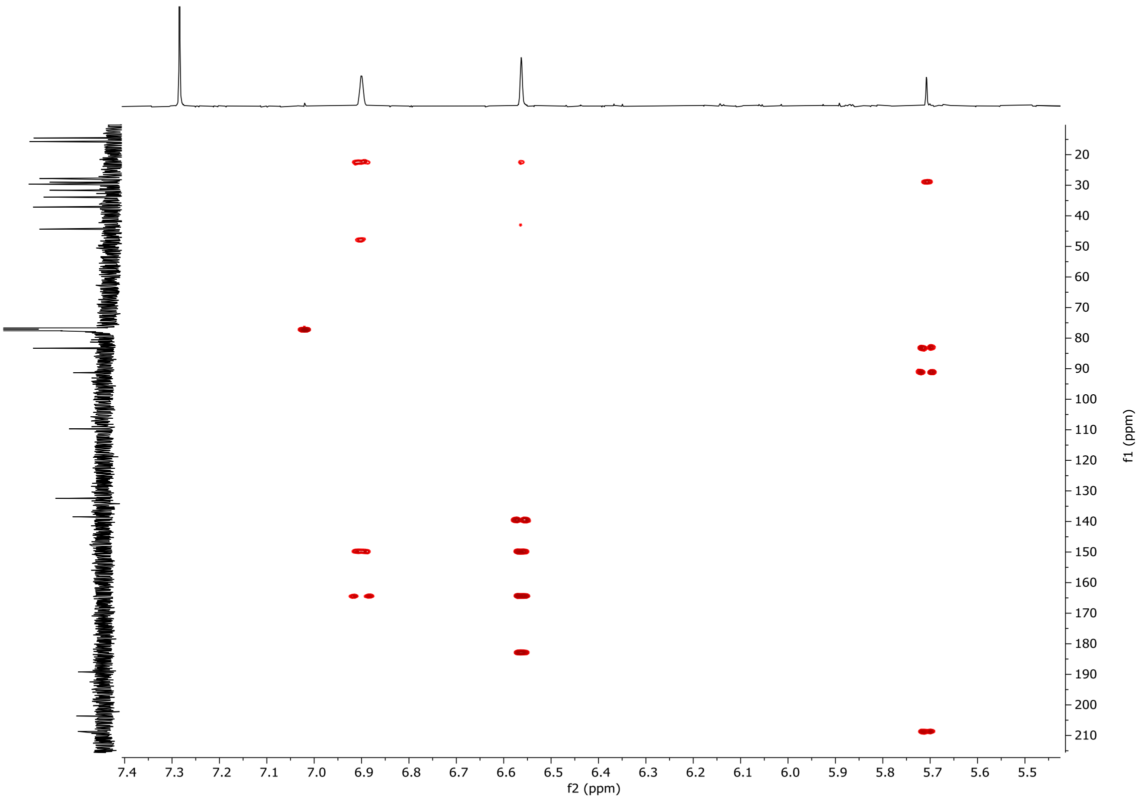

Figure S104. HSQC contour map of Ribifolone H in $CDCl_3$.

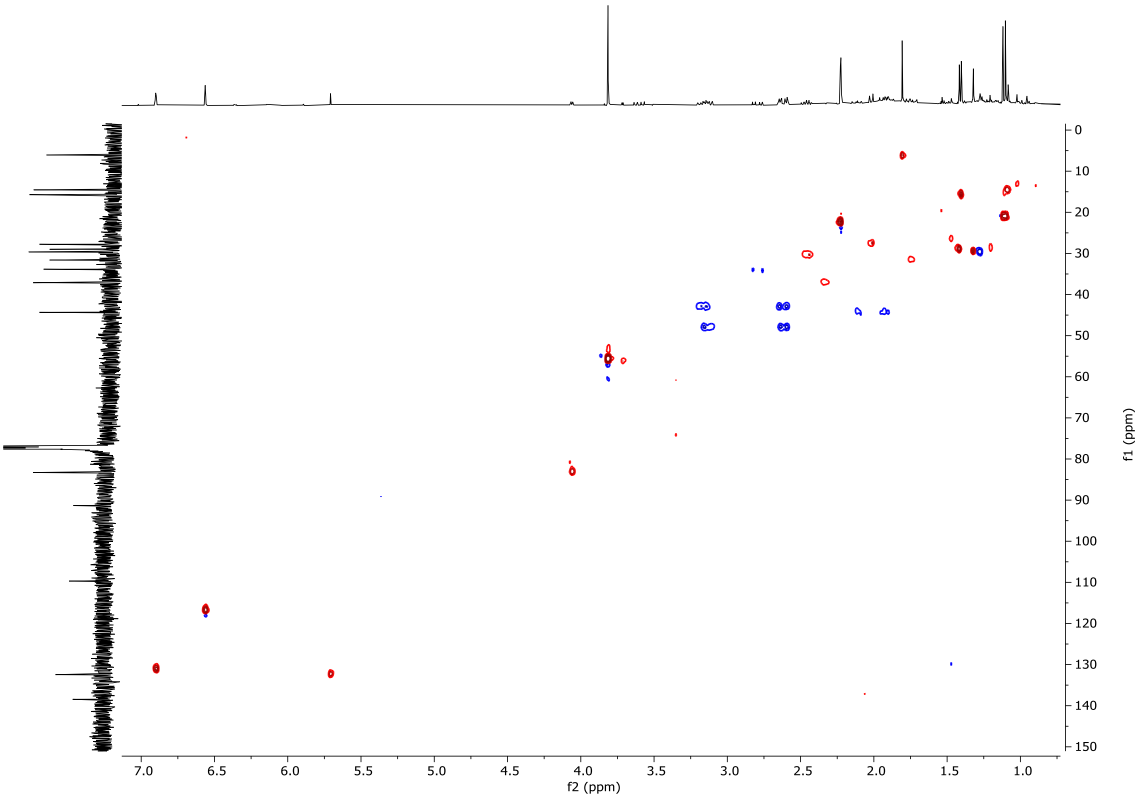

Figure S105. NOESY spectrum of Ribifolone H, 500MHz in $CDCl_3$.

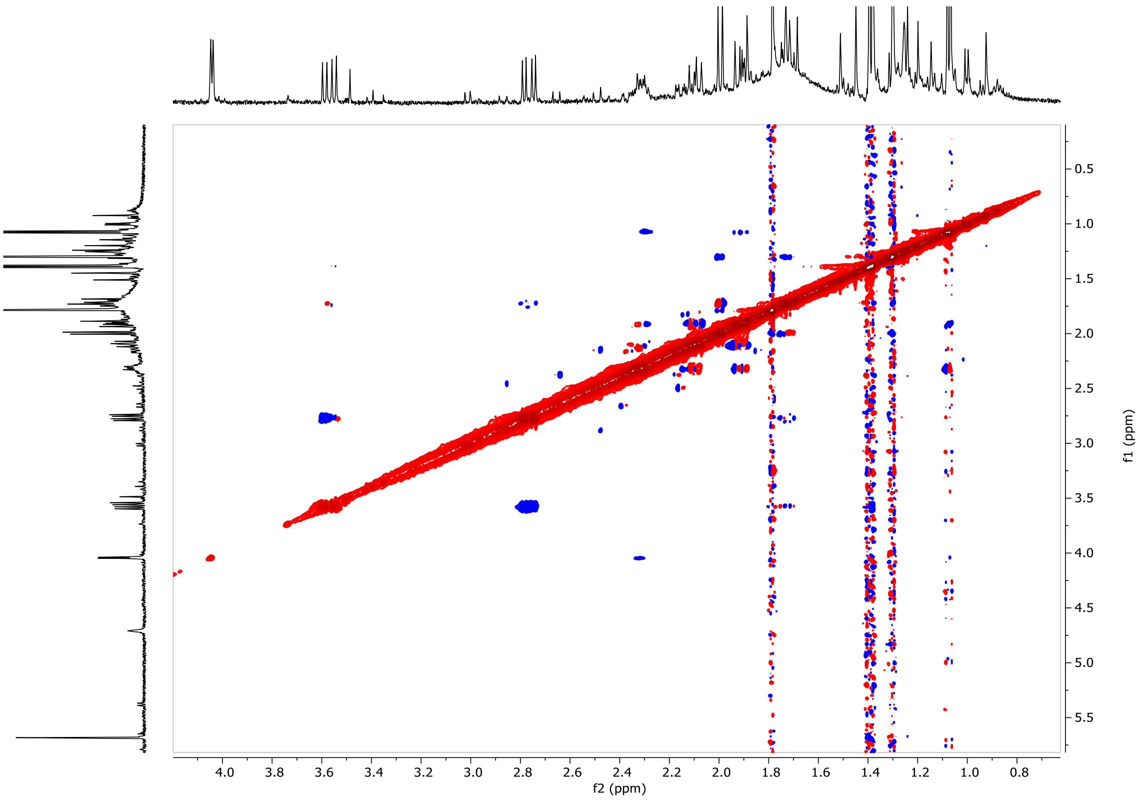

Figure S106. Infrared spectrum of Ribifolone H.

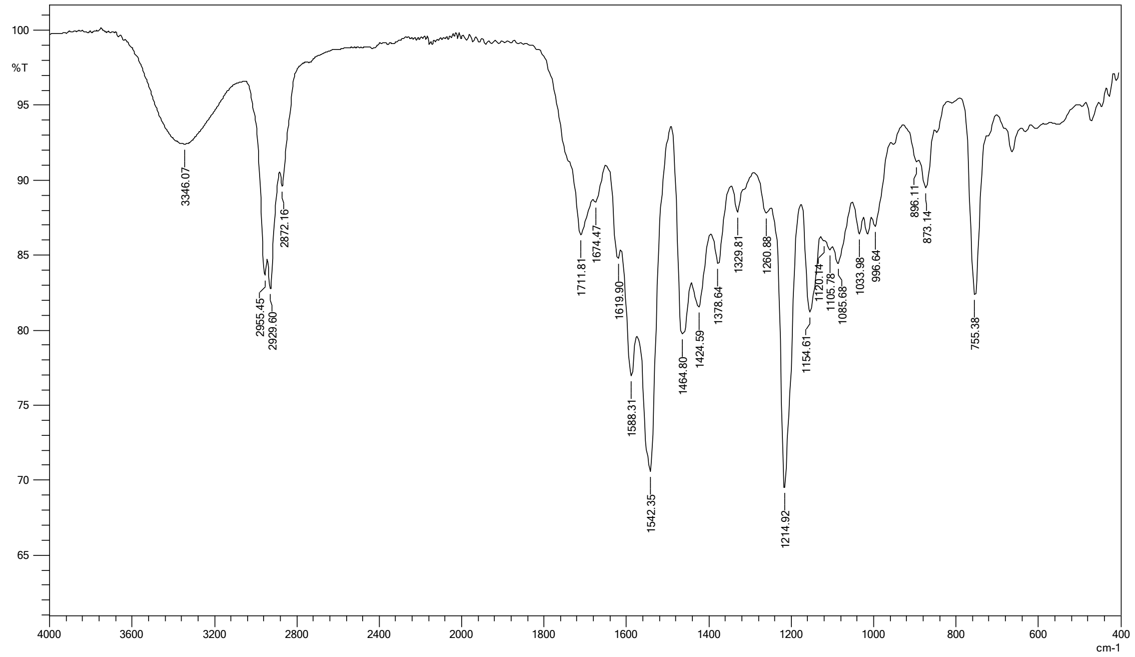

Figure S107. Ultraviolet (UV) spectrum of Ribifolone H.

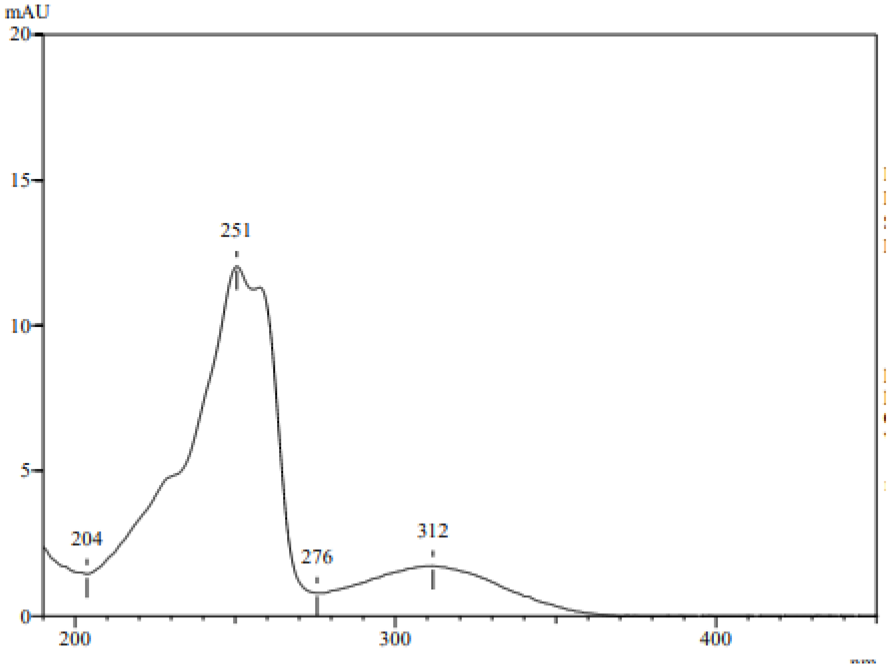

Figure S108. HR-ESI-MS spectrum of 9β,13α-dehydroxyisabellione.

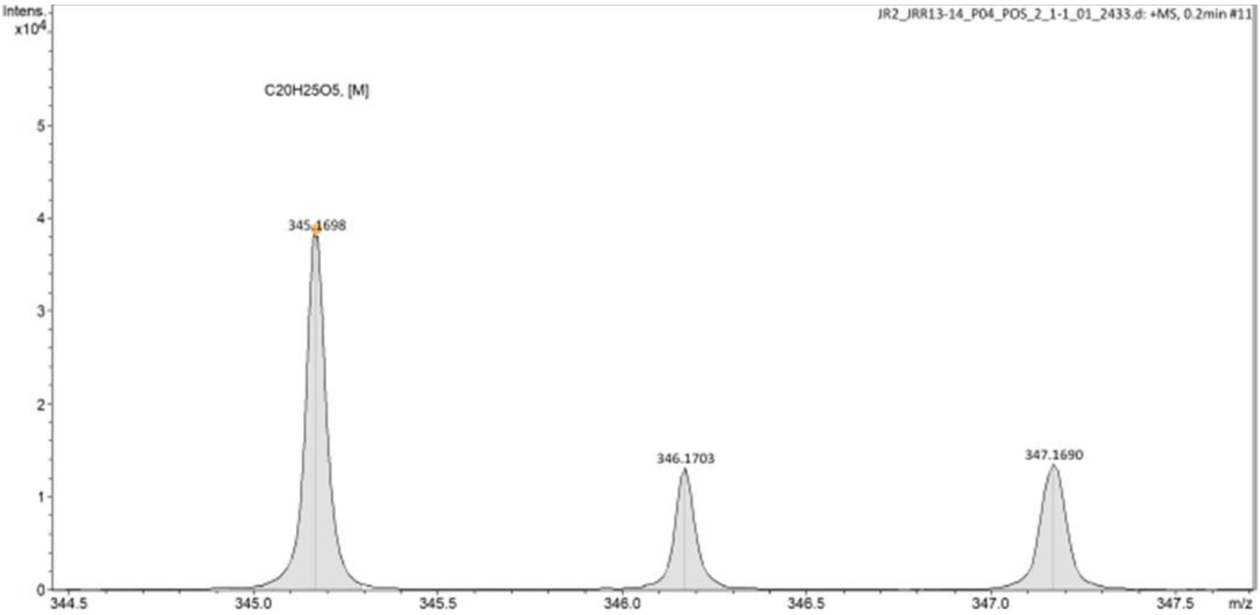

Meas. m/z	#	Ion Formula	m/z	err [ppm]	mSigma	# mSigma	Score	rdb	e⁻ Conf	N-Rule
345.1698	1	C20H25O5	345.1697	-0.3	198.5	1	100.00	8.5	even	ok

Figure S109. ^{1}H spectrum of 9β,13α-dehydroxyisabellione, 400MHz in $CDCl_3$.

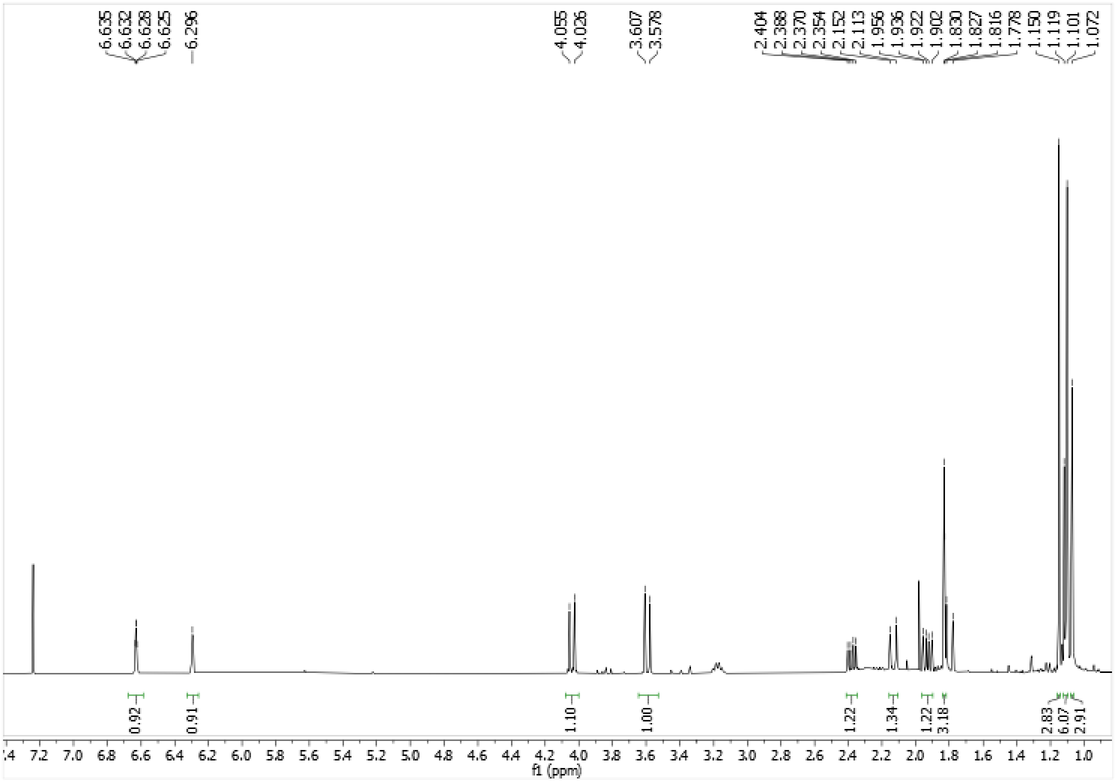

Figure S110. ^{13}C spectrum of 9β,13α-dehydroxyisabellione, 100MHz in CDCl$_3$.

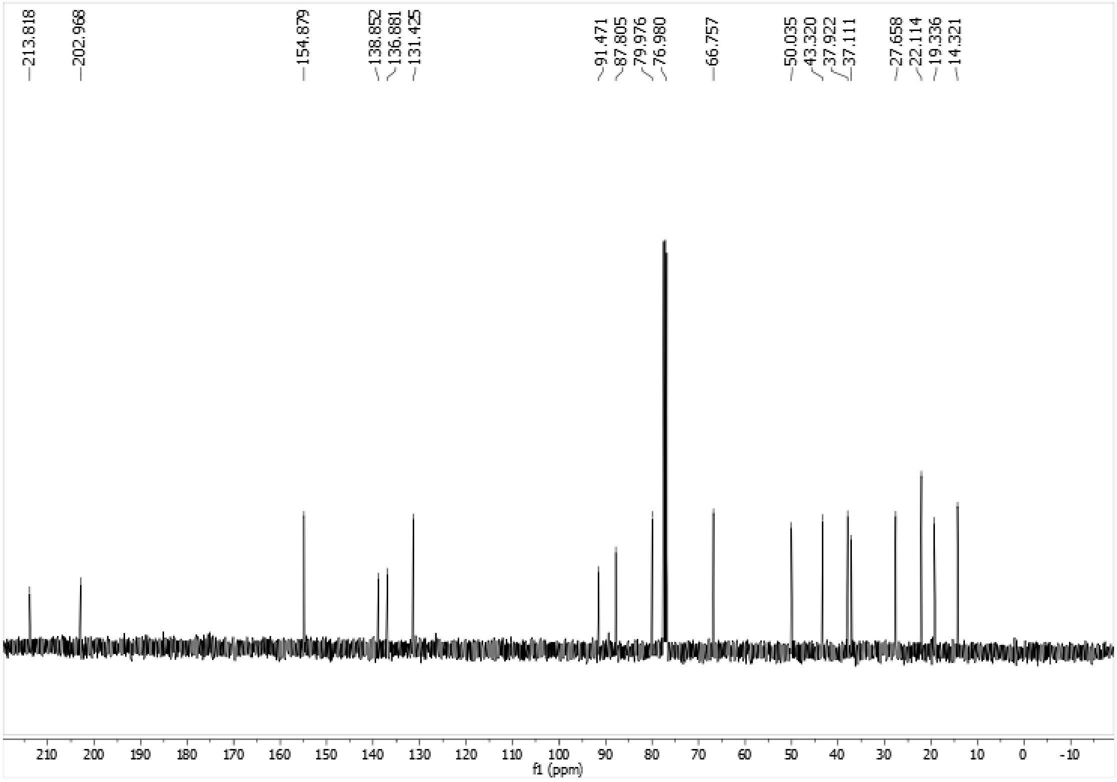

Figure S111. DEPT135 spectrum of 9β,13α-dehydroxyisabellione, 100 MHz in CDCl$_3$.

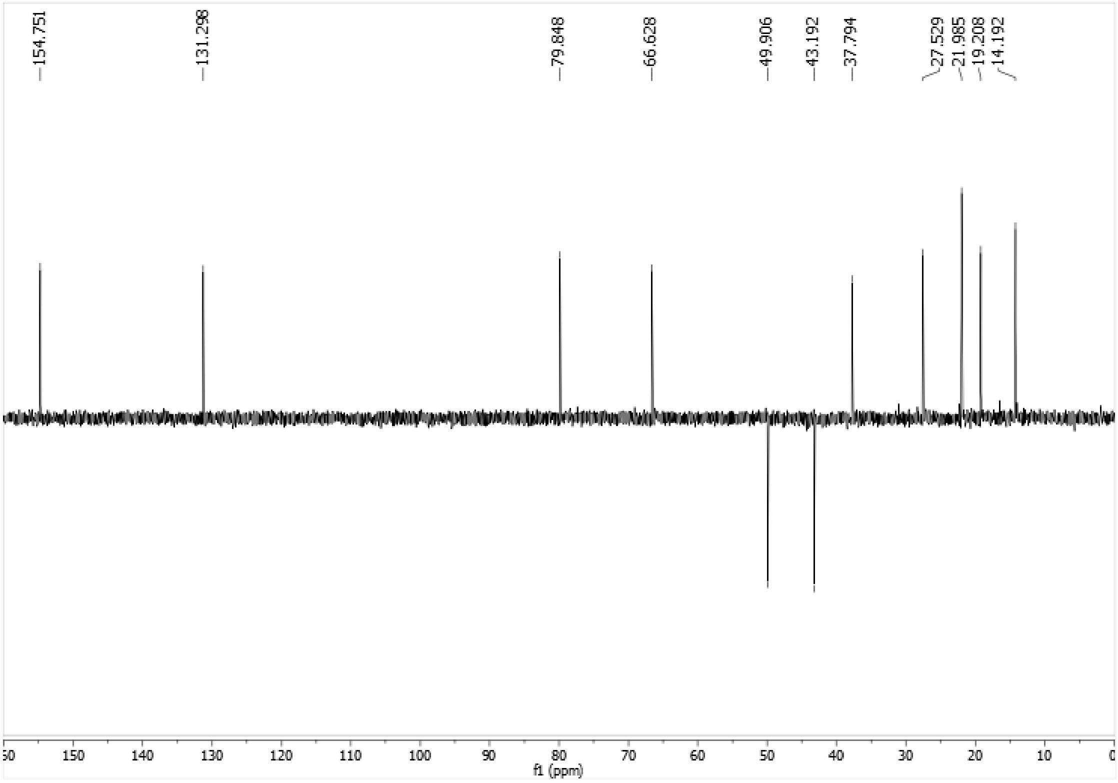

Figure S112. HR-ESI-MS spectrum of Jatrophone.

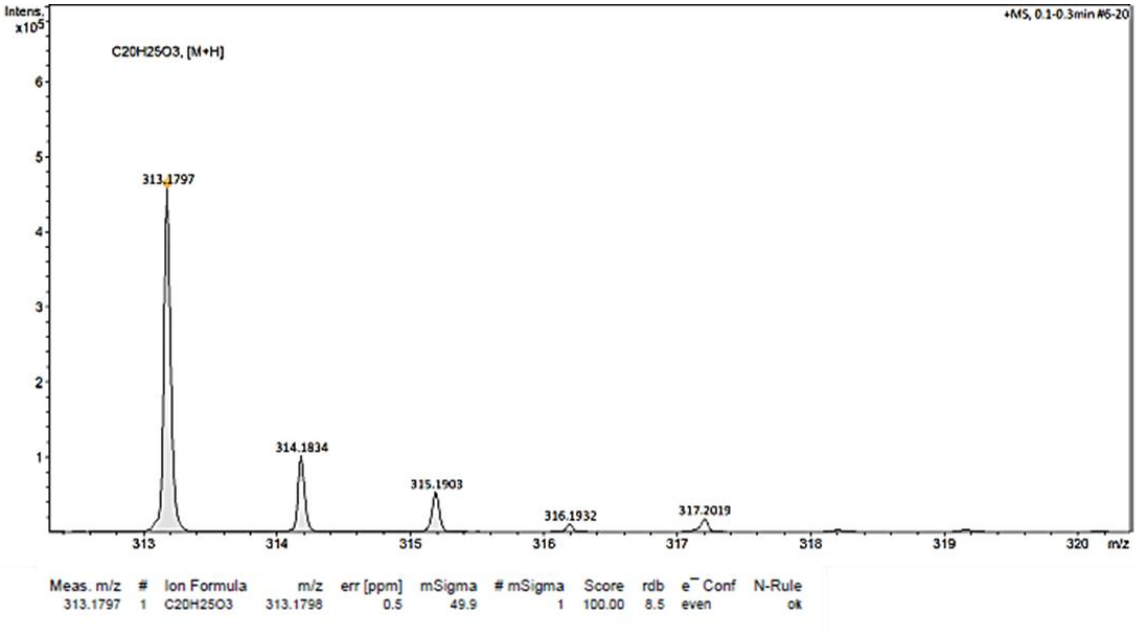

Figure S113. ^{1}H spectrum of Jatrophone, 400MHz in $CDCl_3$.

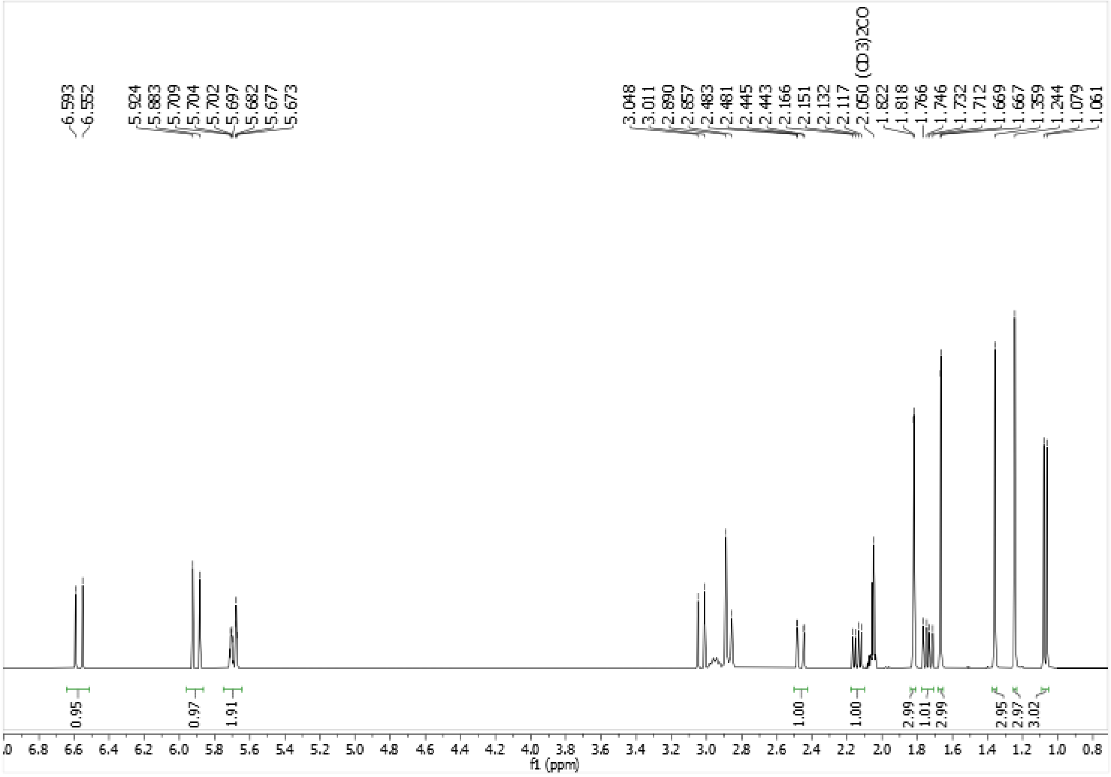

Figure S114. ^{13}C spectrum of Jatrophone, 100MHz in $CDCl_3$.

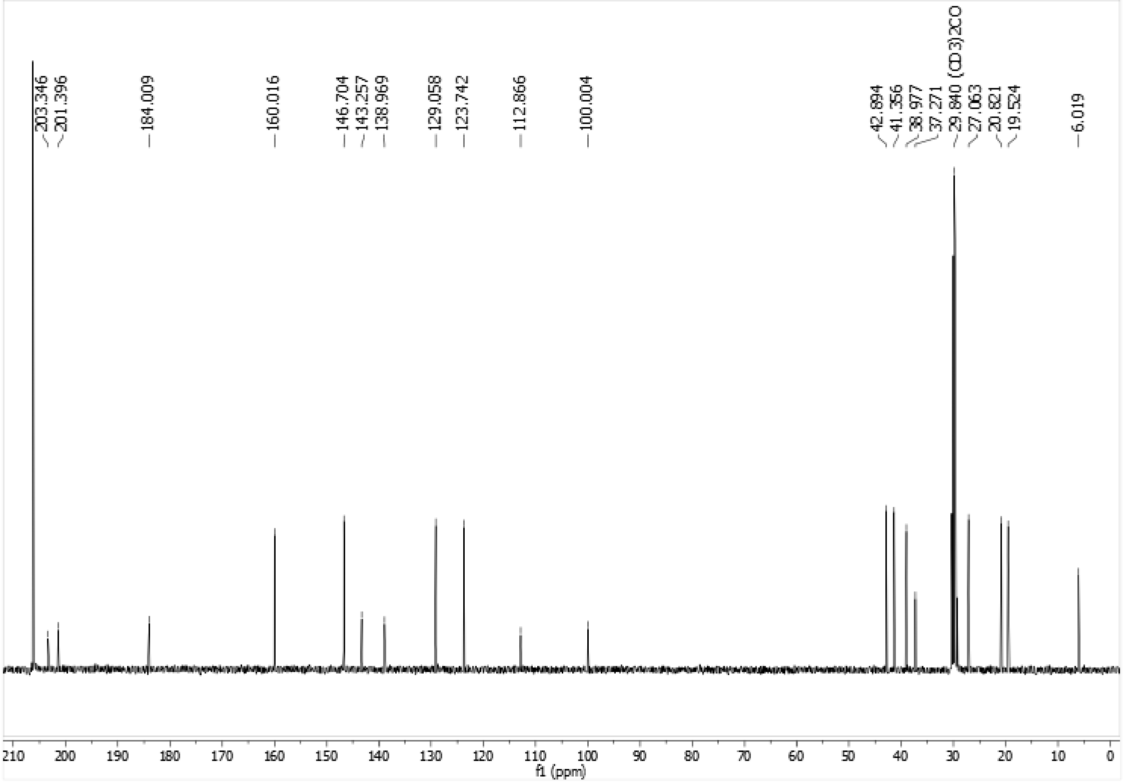

Figure S115. HR-ESI-MS spectrum of 2α-jatrophone, 400MHz in $CDCl_3$.

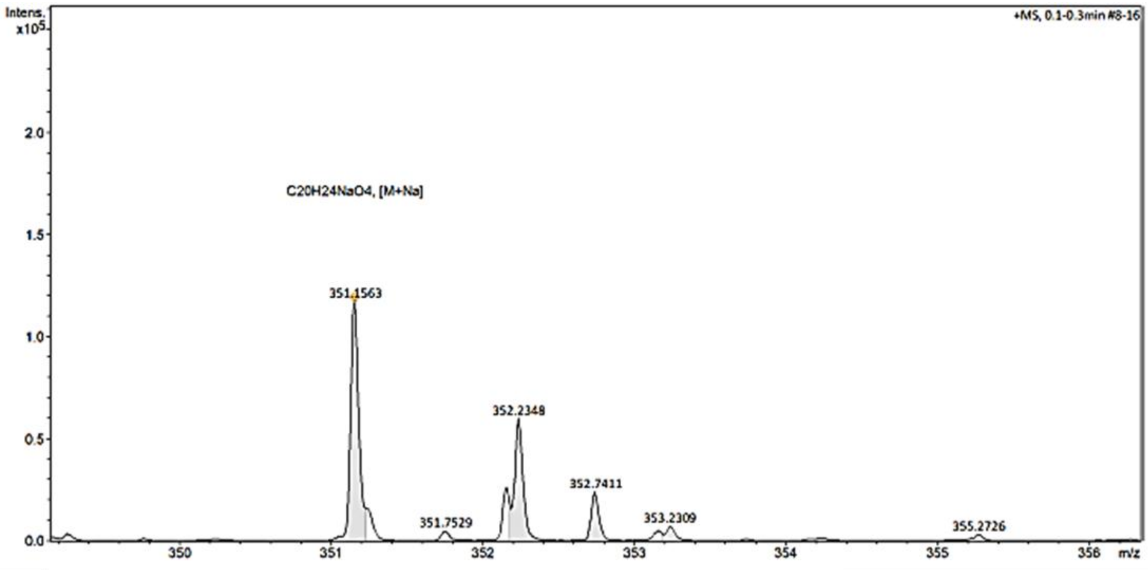

Meas. m/z	#	Ion Formula	m/z	err [ppm]	mSigma	# mSigma	Score	rdb	e⁻ Conf	N-Rule
351.1563	1	C20H24NaO4	351.1567	1.0	n.a.	1	100.00	8.5	even	ok

Figure S116. ^{1}H spectrum of 2α-jatrophone, 400MHz in $CDCl_3$.

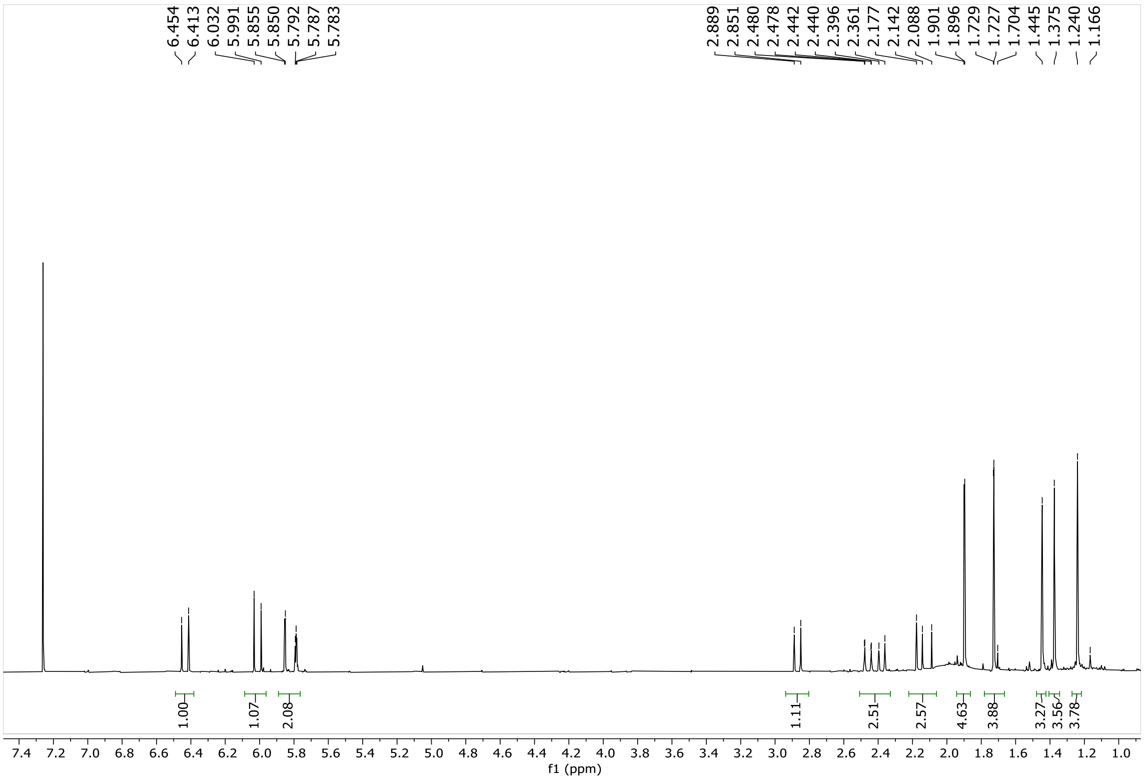

Figure S117. ^{13}C spectrum of 2α-jatrophone, 100MHz in $CDCl_3$.

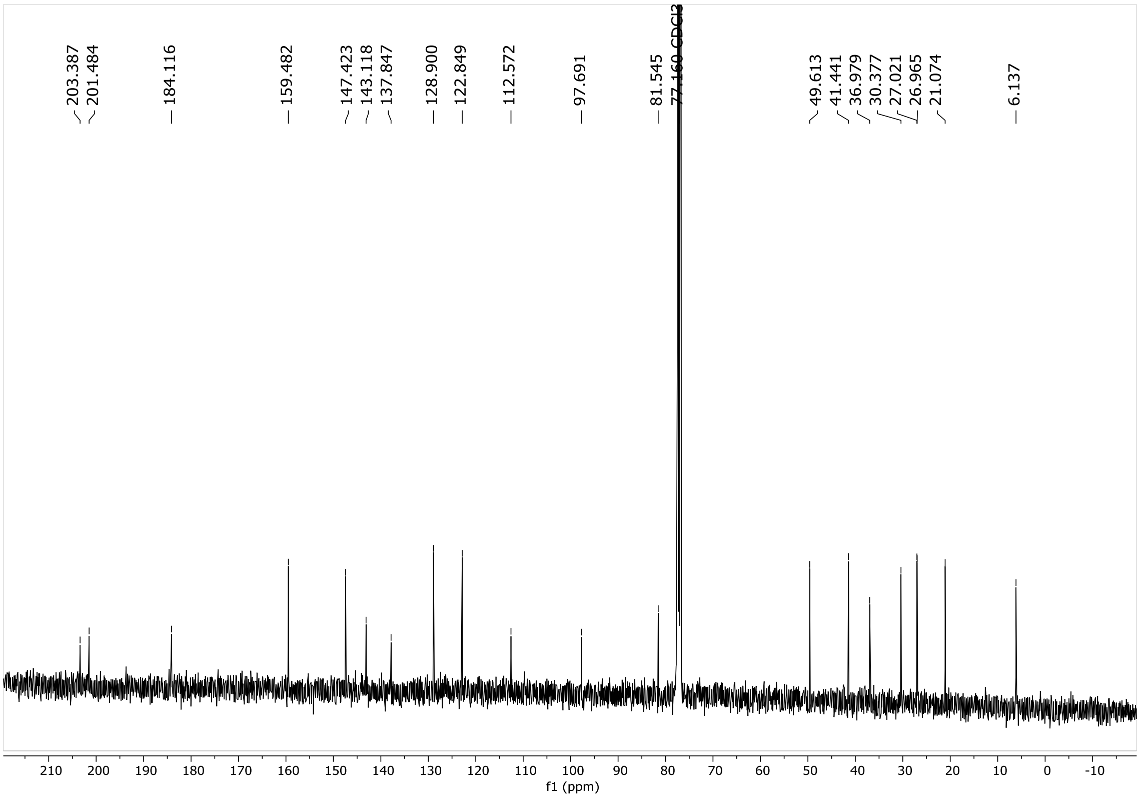

Figure S118. HR-ESI-MS spectrum of 2β-jatrophone, 400MHz in CDCl3.

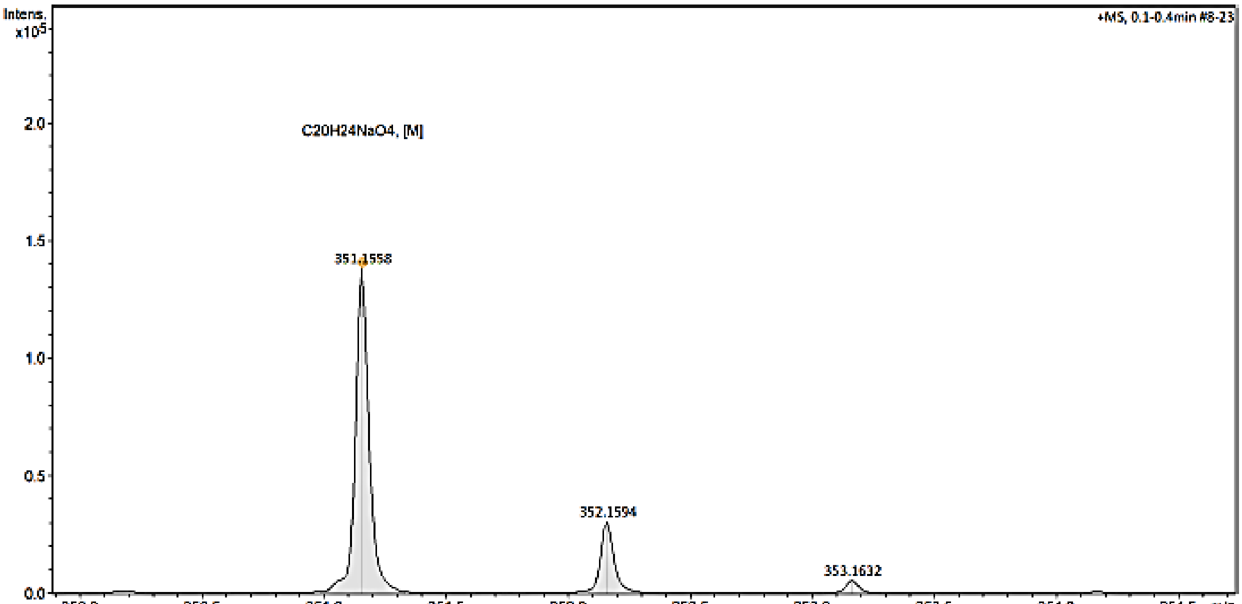

Meas. m/z	#	Ion Formula	m/z	err [ppm]	mSigma	# mSigma	Score	rdb	e⁻ Conf	N-Rule
351.1558	1	C20H24NaO4	351.1567	2.6	5.2	1	100.00	8.5	even	ok

Figure S119. ^{1}H spectrum of 2β-jatrophone, 400MHz in CDCl3.

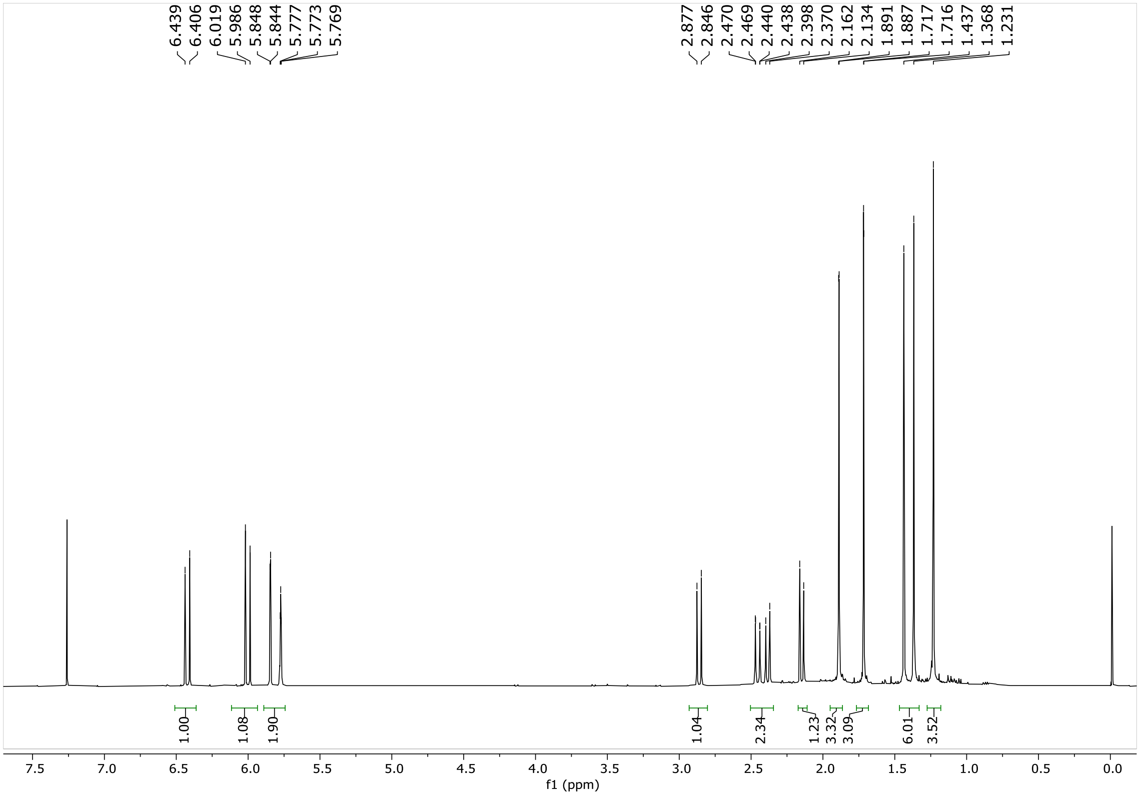

Figure S120. HR-ESI-MS spectrum of Citlalitrione, 400MHz in $CDCl_3$.

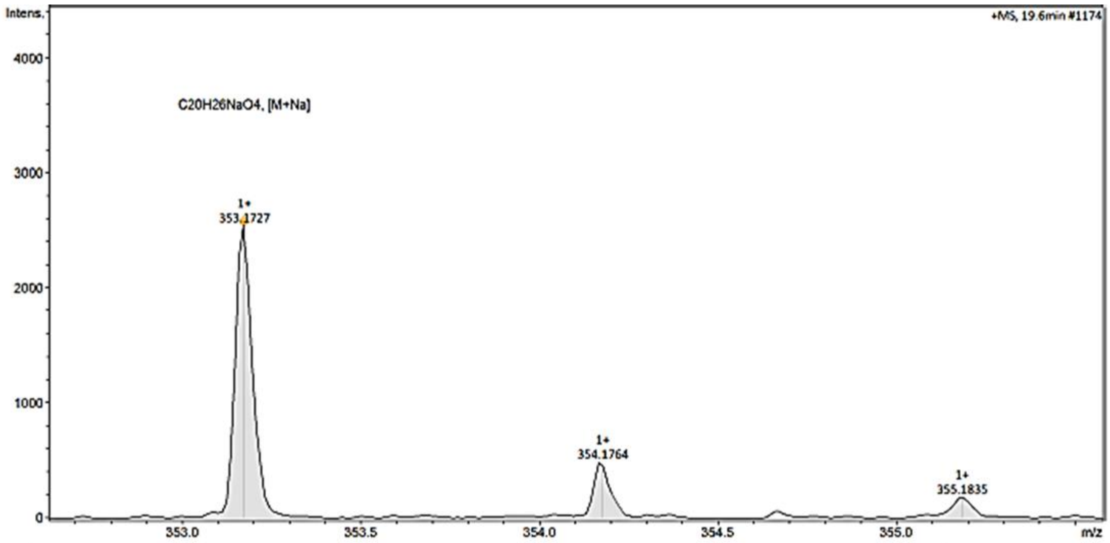

Meas. m/z	#	Ion Formula	Score	m/z	err [mDa]	err [ppm]	mSigma	rdb	e⁻ Conf	N-Rule	Adduct
353.172702	1	C20H26NaO4	100.00	353.172330	-0.4	-1.1	28.3	7.5	even	ok	M+Na

Figure S121. ^{1}H spectrum of Citlalitrione, 400MHz in $CDCl_3$.

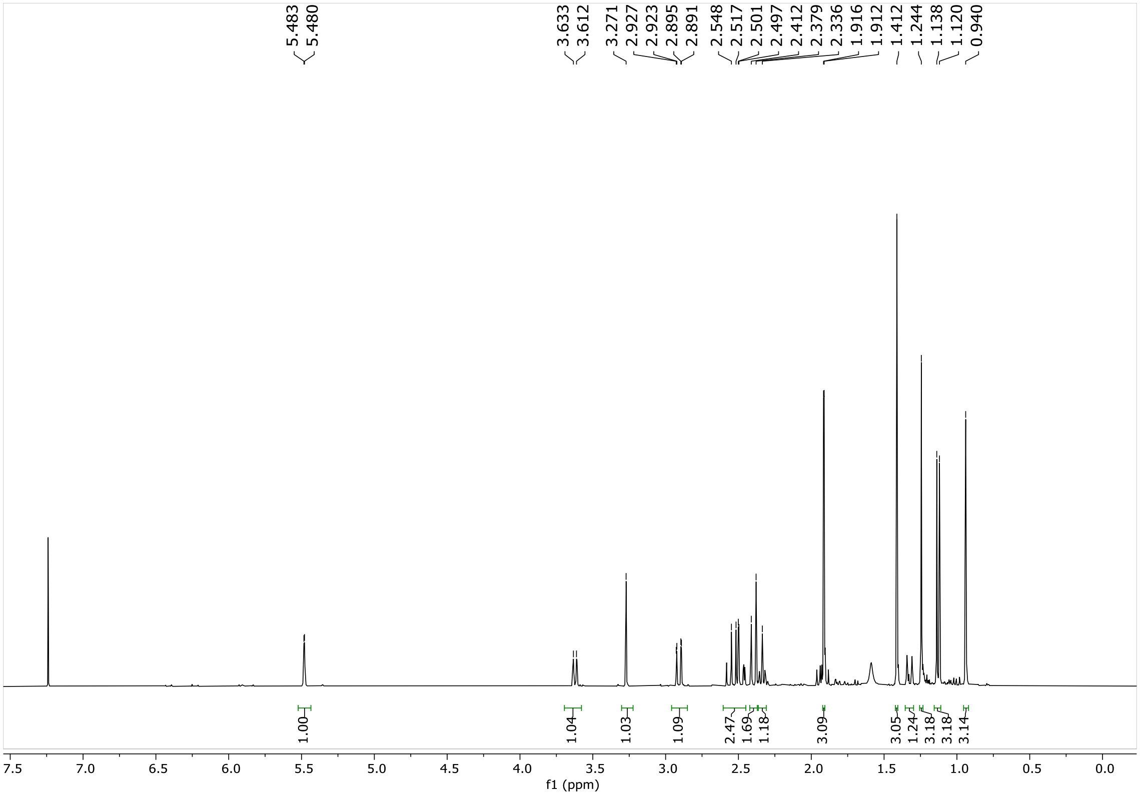

Figure S122. ^{13}C spectrum of Citlalitrione, 100MHz in $CDCl_3$.

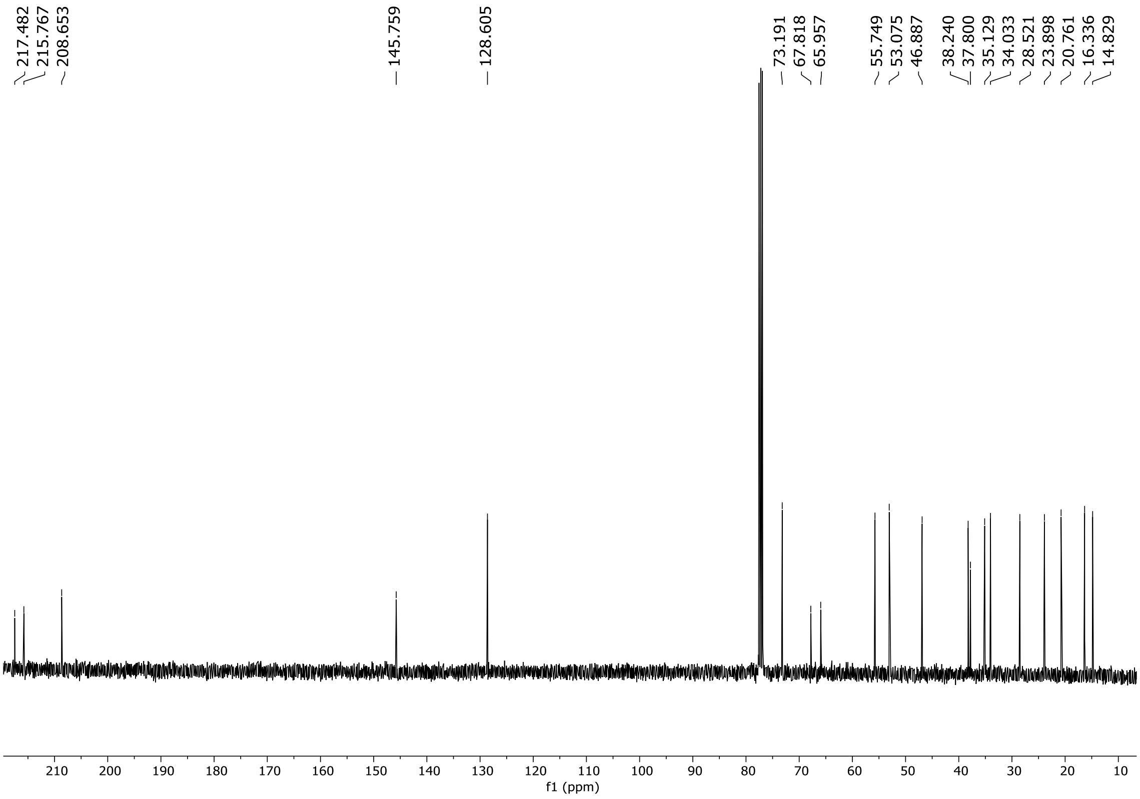

Figure S123. ^{1}H spectrum of 6-Hydroxycyperene, 400MHz in $CDCl_3$.

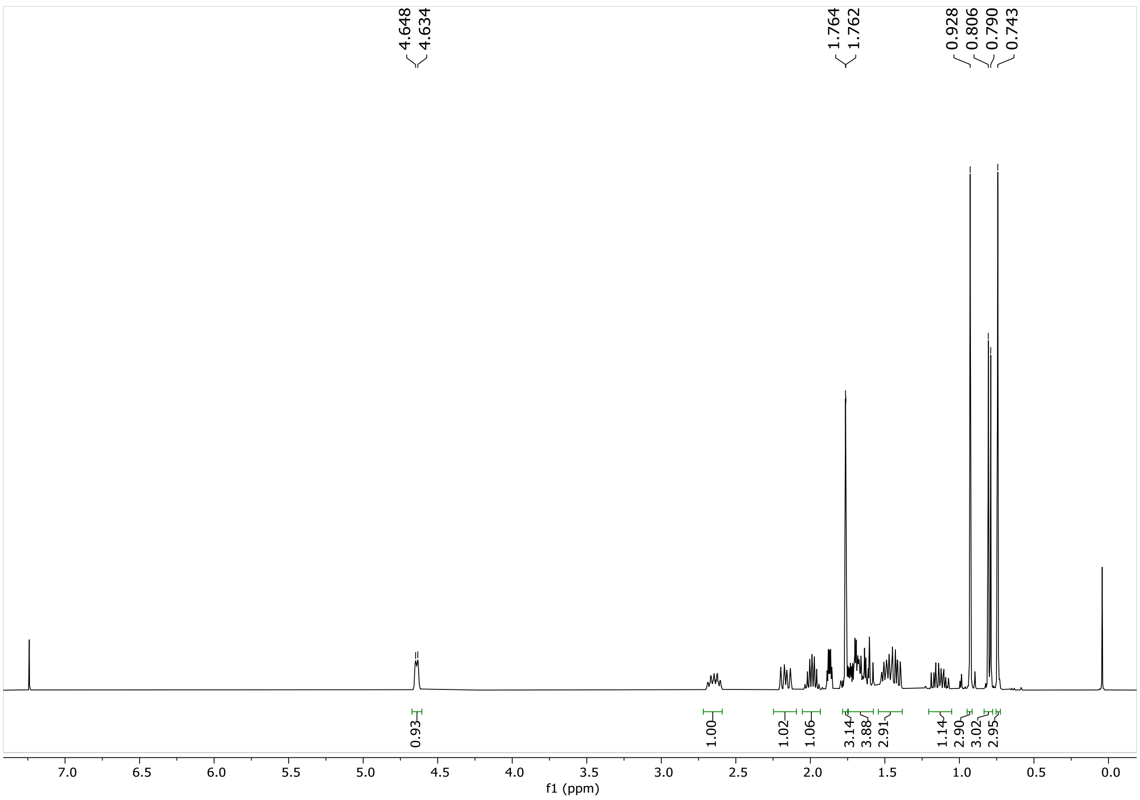

Figure S124. ^{13}C spectrum of 6-Hydroxycyperene, 100MHz in $CDCl_3$.

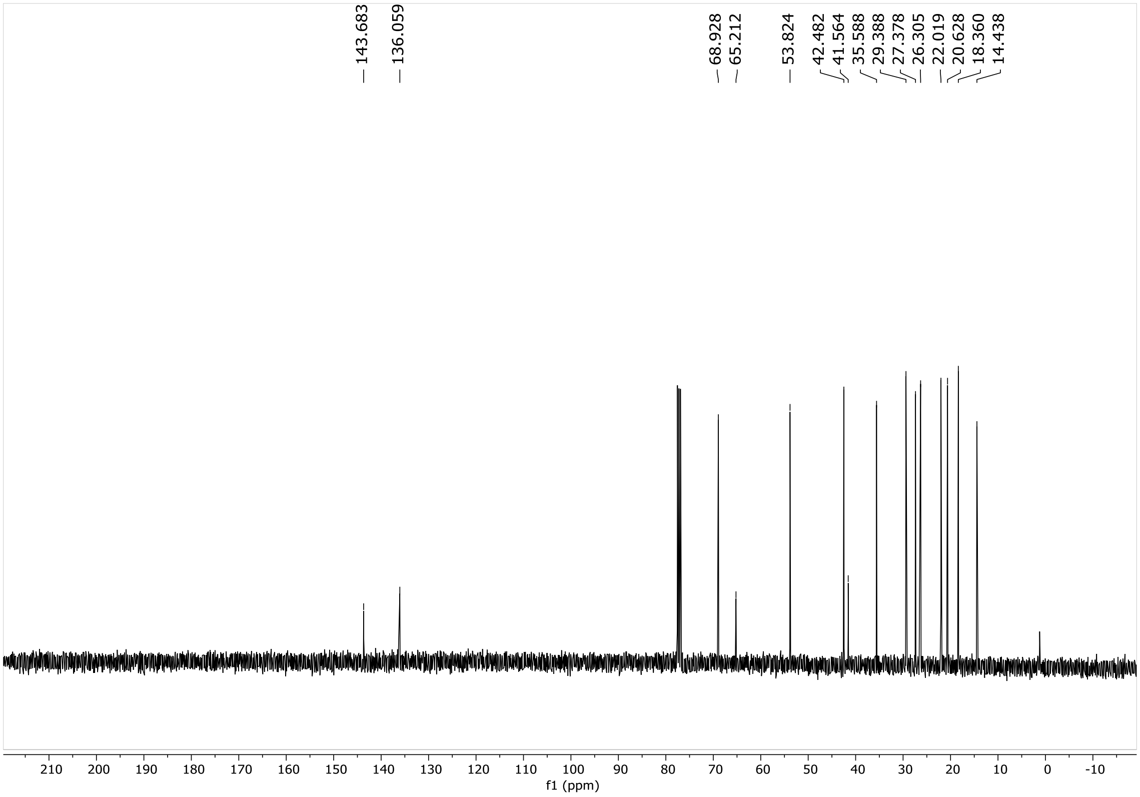

Figure S125. DEPT135 spectrum of 6-Hydroxycyperene, 100MHz in $CDCl_3$.

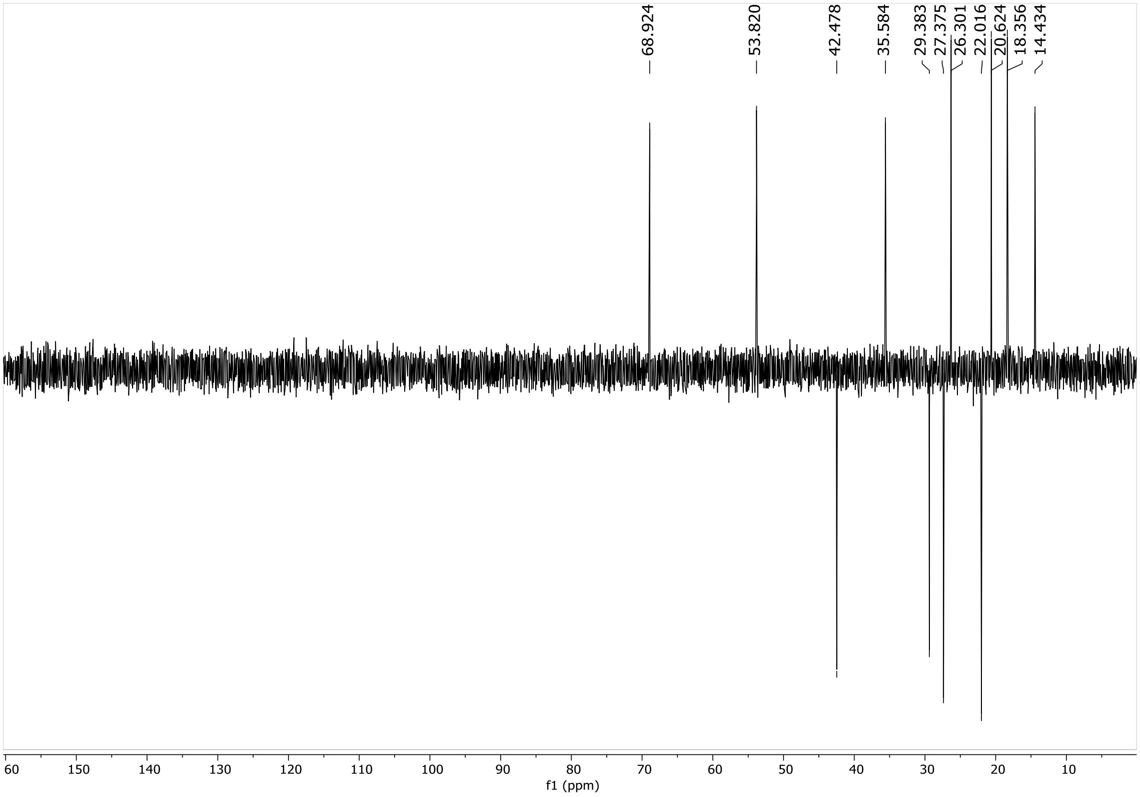

Figure S126. ^{1}H spectrum of sugeonol, 400MHz in $CDCl_3$.

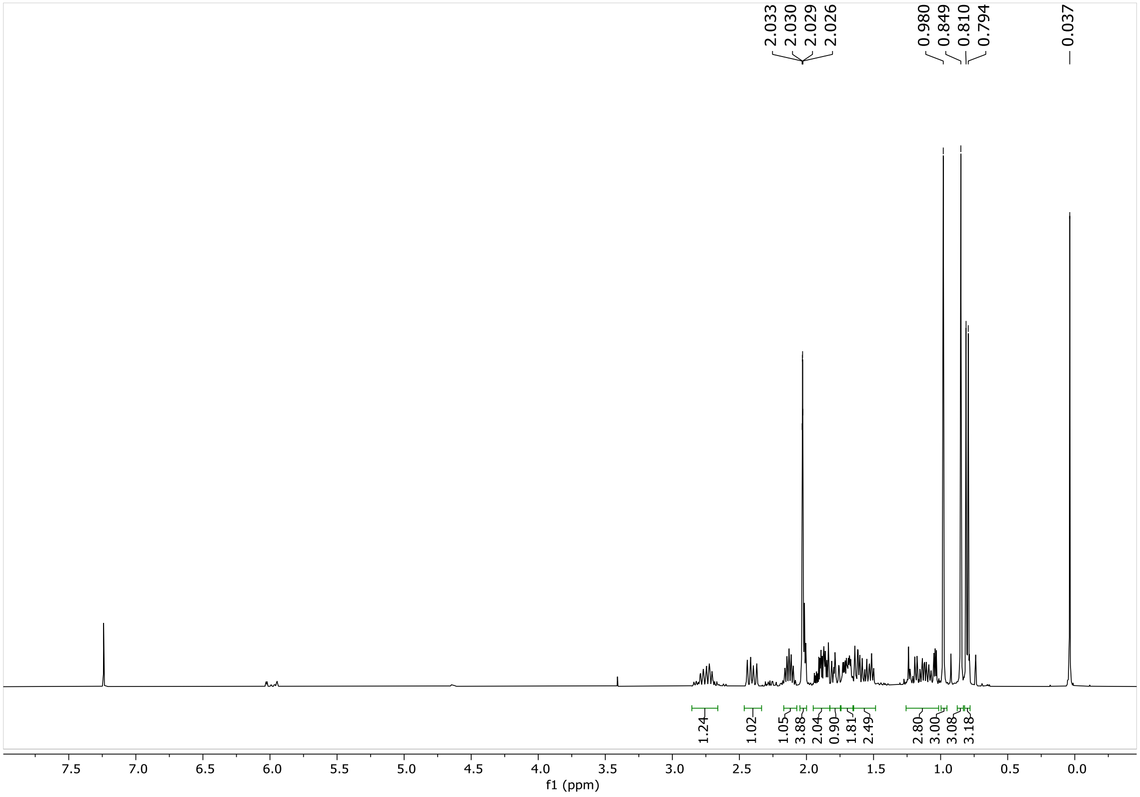

Figure S127. ^{13}C spectrum of sugeonol, 100MHz in $CDCl_3$.

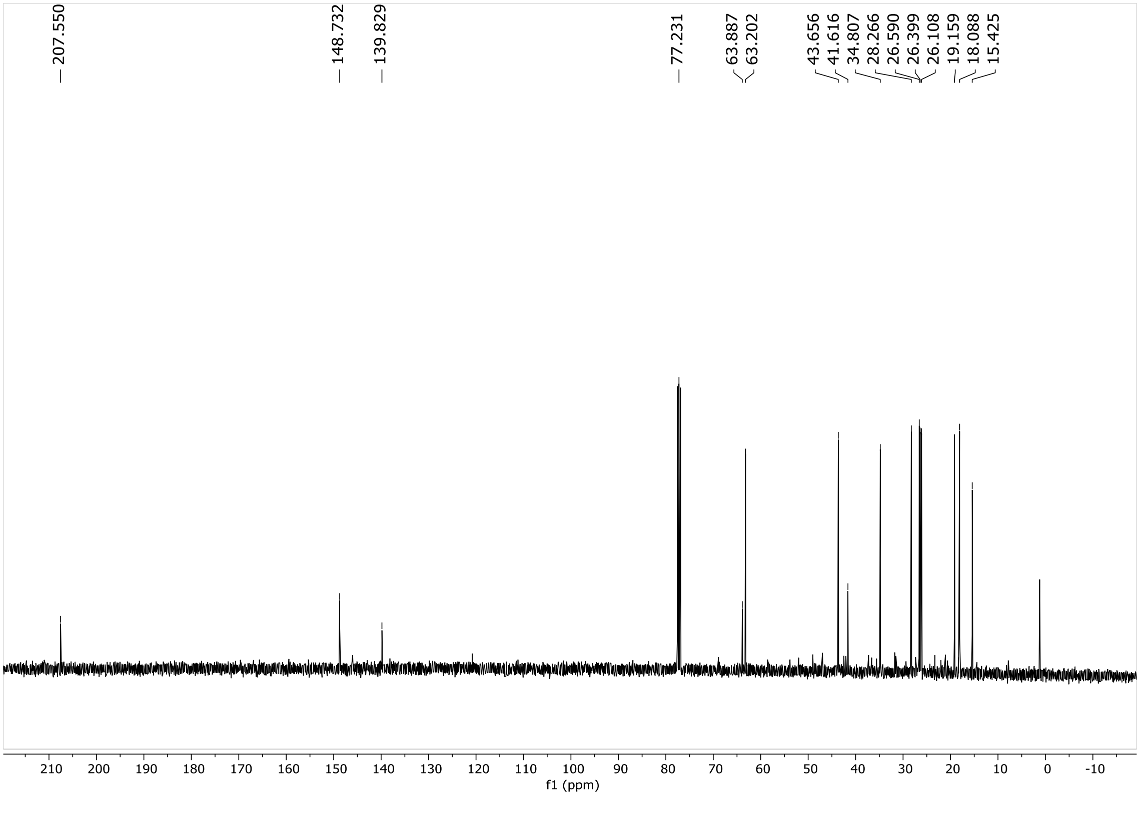

Figure S128. DEPT135 spectrum of sugeonol, 100MHz in $CDCl_3$.

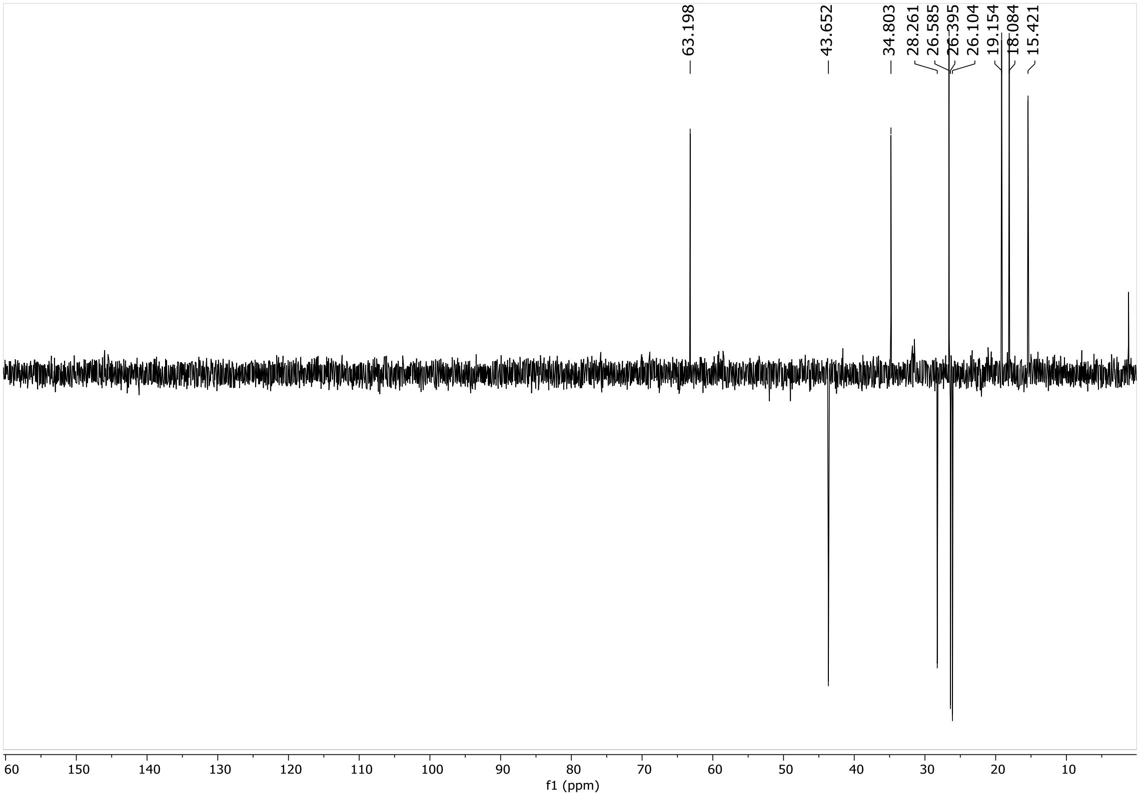

Figure S129. ^{1}H spectrum of Patchoulenone, 400MHz in $CDCl_3$.

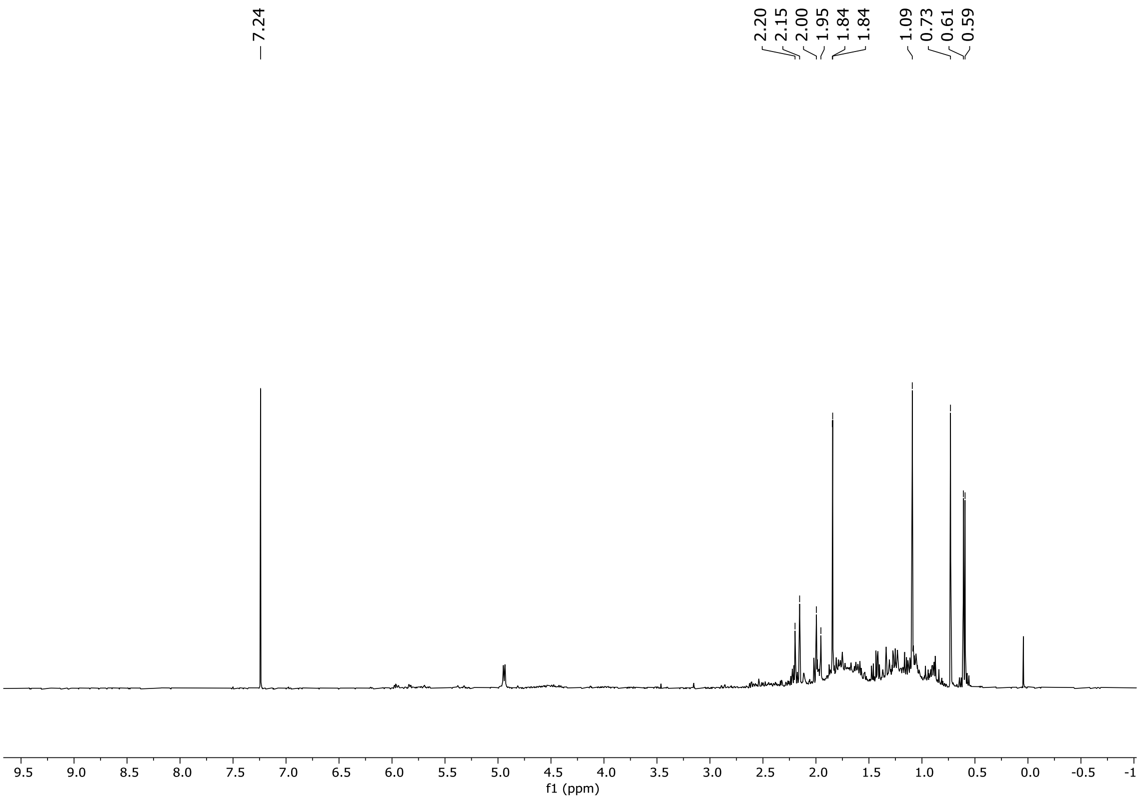

Figure S130. ^{13}C spectrum of Patchoulenone, 400MHz in $CDCl_3$.

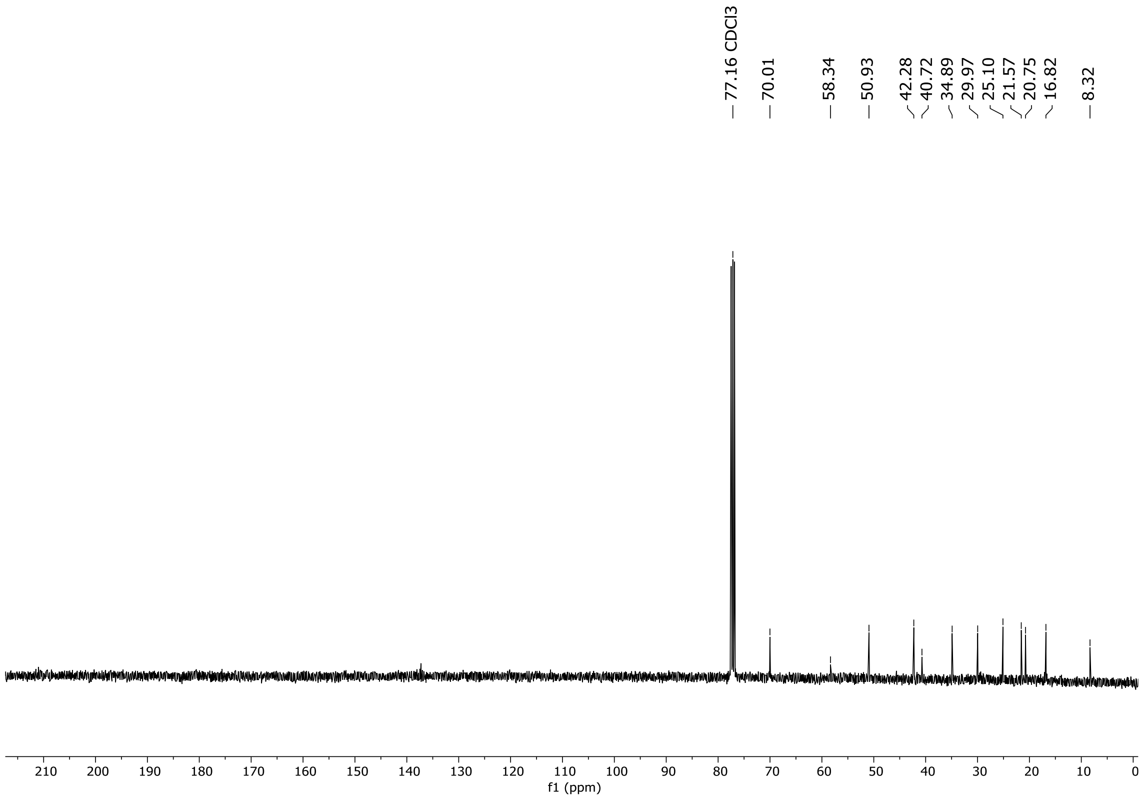

Figure S131. HMBC contour map of patchoulenone, 100MHz x 400MHz in $CDCl_3$.

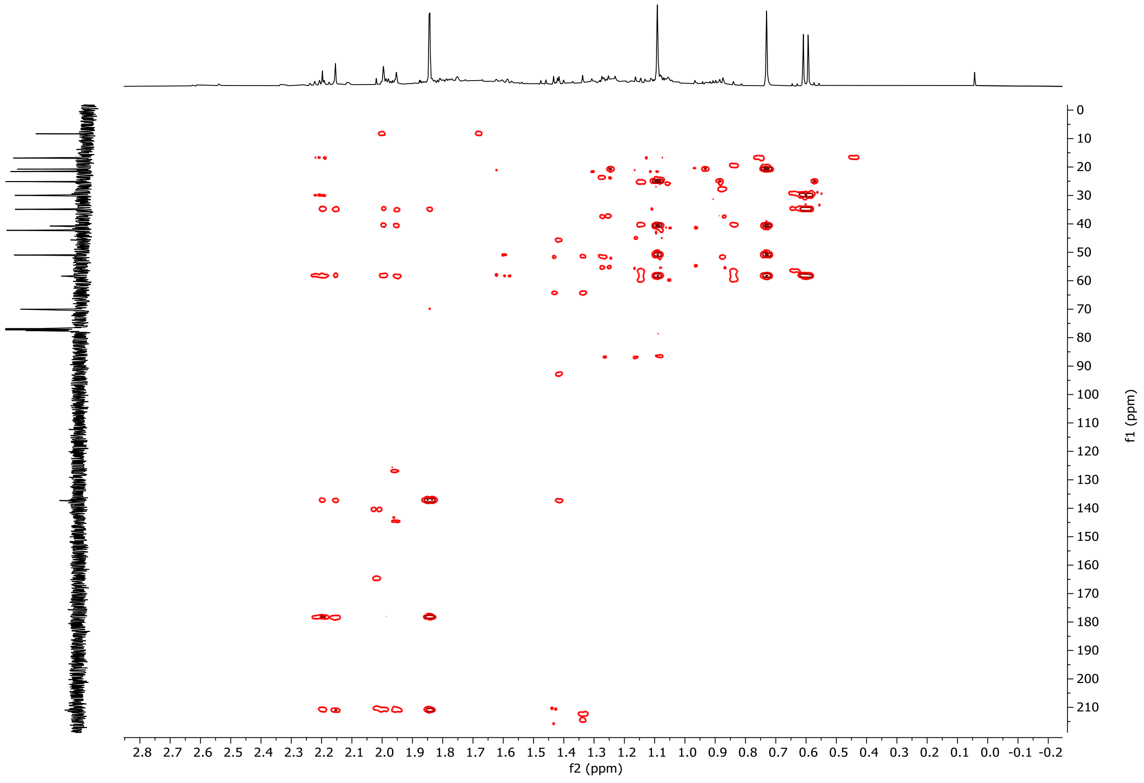

Molecular Docking

Figure S132. Three-dimensional representation of ribifolone C and jatrophone complexed with the selected molecular targets involved in the PI3K-AKT-mTor pathway, superimposed with their respective co-crystalized ligands.

PI3K

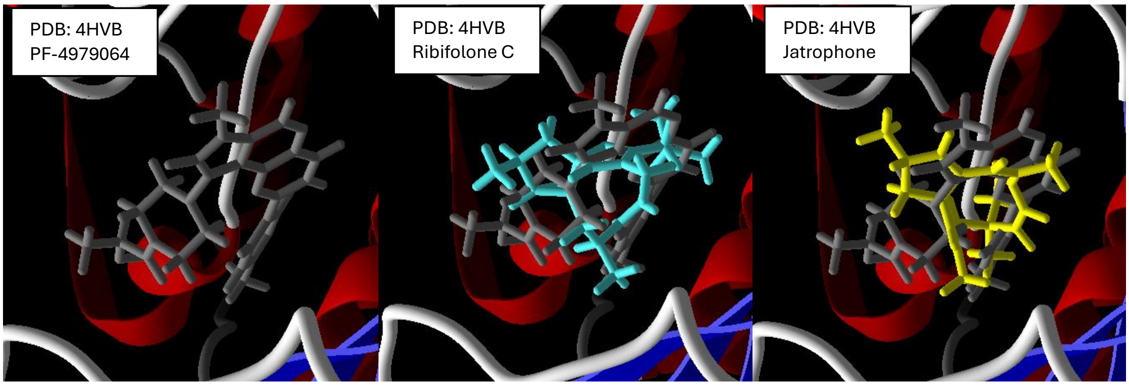

ERβ

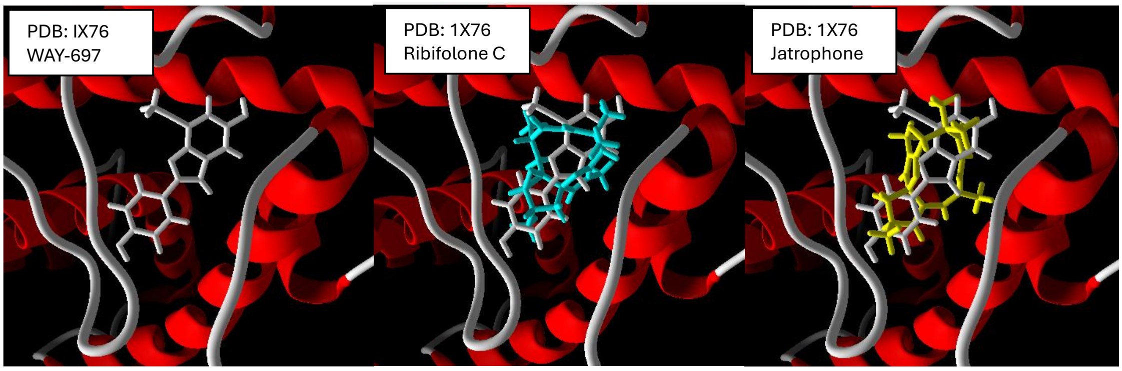

ERα

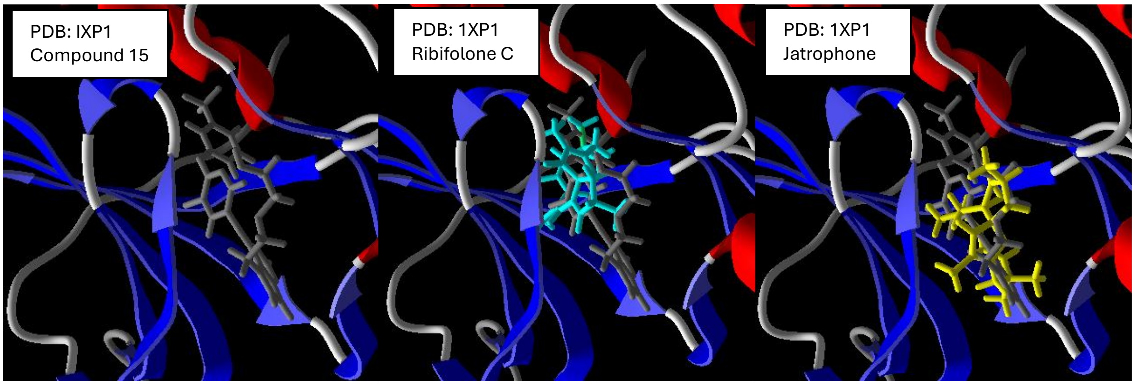

HER2

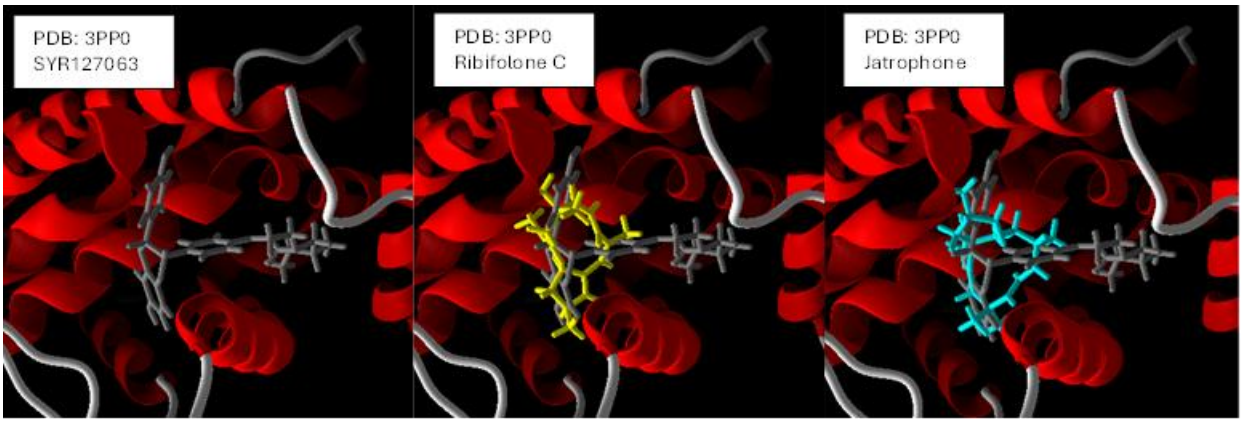

HSP

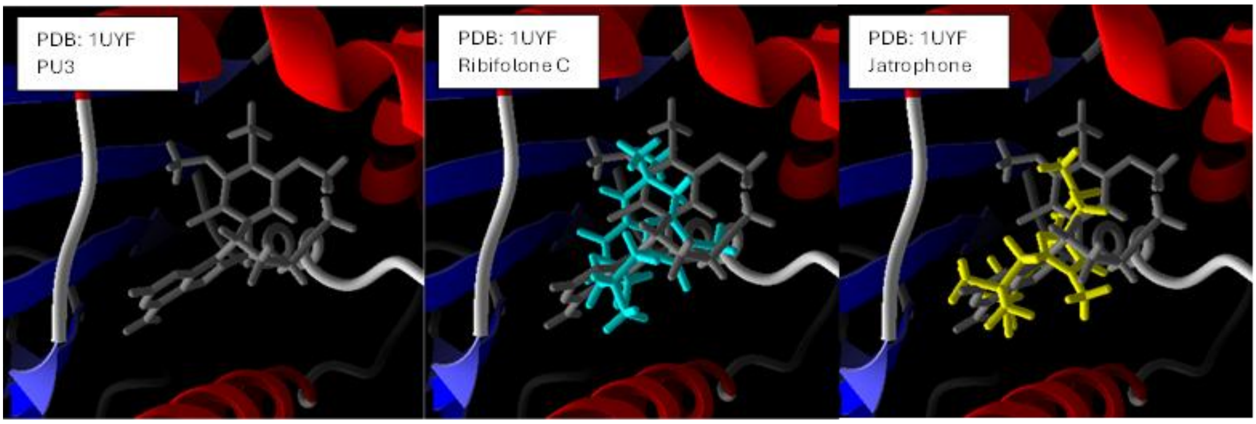

Molecular Docking for HER2, HSP90 and PI3Kγ proteins

HER2 (human epidermal growth factor receptor 2) is a receptor that bind and form heterodimers to activate growth factors and signaling pathways that promote cell proliferation and survival. The overexpression of HER2 can lead to uncontrolled if often seen in many cancer types, including melanoma and colorectal cancer.

The inhibitor SYR127063 is a potent HER2 inhibitor with an IC50 of 11 nM. This molecule prevents the HER2 dimer from getting in the right conformation to be catalytically active. In the docking simulations, the complex formed with this inhibitor had the lowest docking score (−211.971) and an LE of 6.23, compared with Ribifolone C (score −109.498; LE 4.56) and jatrophone (score −109.166; LE 4.75).

Jatrophone and ribifolone C form mainly hydrophobic interactions, but the first one also exhibits more unfavorable contacts (Lys753, Leu796, Asp863) than Ribifolone C (Phe864), which suggests a more instable complex (Figure 133A,B).

The amino acid interactions showed that SYR127063 forms multiple hydrogen bonds (Glu770, Asn850, Asp863) and carbon–hydrogen bonds (Gln799, Arg849), which can explain the scores seen (Figure 133C).

Figure S133. Amino acids interactions formed between HER2, HSP90α, and PI3Kγ. and ribifolone C, jatrophone and the reference ligands. Amino acid interactions between ribifolone C (A), jatrophone (B), and SYR127063 against HER2. D/E/F. Amino acid interactions between ribifolone C (D), jatrophone (E), and PU3 (F) against HSPα. G/H/I. Amino acid interactions between ribifolone C (G), jatrophone (H), and PF-04979064 (I) against PI3Kγ. Amino acid interactions between ribifolone C (J), jatrophone (K), and Compound 15 (L)

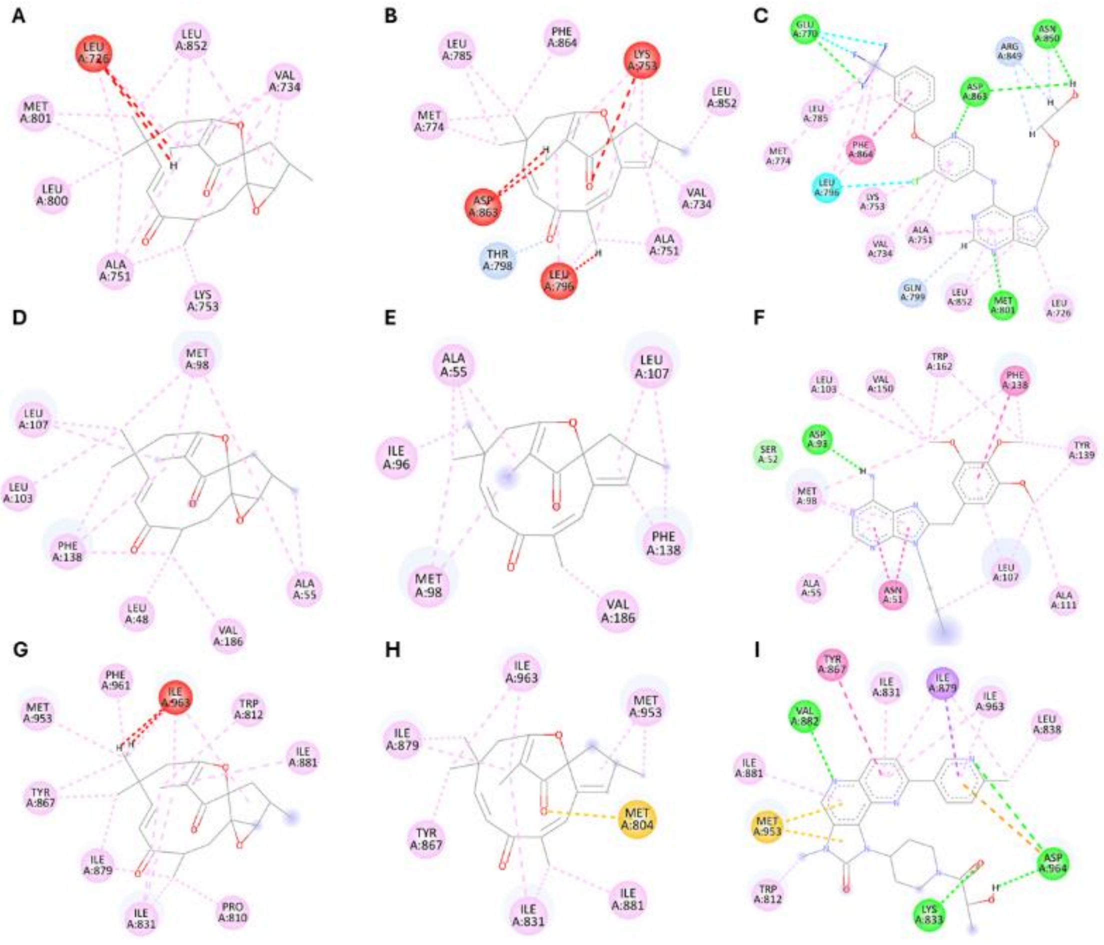

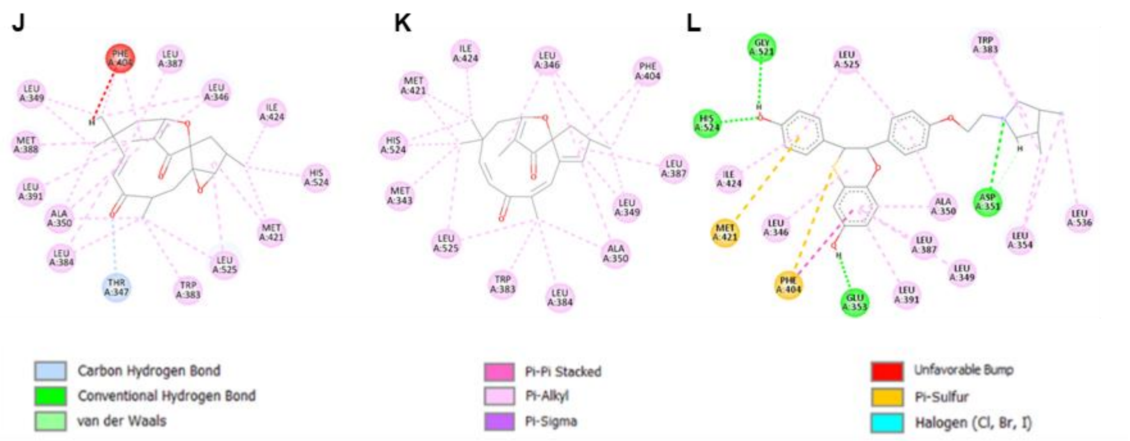

Heat shock proteins (HSPs) are a family of enzymes involved in the correct folding of proteins. These molecules can stabilize the active conformation of oncogenic factors, such as HER2 and AKT, which prevents their degradation, also they can inhibit apoptosis of cancerous cells and help avoid the immune system. The isoform of HSP90α is the isoform most related to cancer, strongly upregulated, which makes this protein a strong target for new drugs. For this simulation, the inhibitor PU3 was selected. This inhibitor exhibits moderate potency, with an IC_{50} of 30 μM.

Regarding the complex formed with HSP90, both Ribifolone C and jatrophone (Figure 133-D,E) establish mainly hydrophobic interactions (Ala55, Met98, Leu107, and Phe138), with Ribifolone C forming an additional interaction with Leu48 that is not observed with jatrophone or PU3. PU3, besides hydrophobic interactions with the same residues, also form hydrogen bonds (Asp51, Ser52, Asp93), which explains the higher chemical efficiency observed (Figure 133-F).

Lastly, regarding the PI3K–Akt–mTOR pathway, the dual inhibitor PF-04979064 was used as a comparative ligand. This inhibitor is a highly potent compound designed via structure-based optimization, with subnanomolar activity against both PI3K and mTOR. Both ribifolone C and jatrophone (Figure 133- G,H) share multiple hydrophobic interactions (Ile831, Ile879, Ile881, Ile963, Trp812, and Tyr867). However, jatrophone also forms a π–sulfur interaction with Met804, while ribifolone C forms an unfavorable interaction with Ile963. In addition to hydrophobic interactions (Trp812, Ile831, Tyr867, Ile879, Ile881, Leu838, Ile963), PDBL also establishes additional polar contacts (Figure 133-I), such as hydrogen bonds (Val882, Lys833, and Asp964) and a π–sulfur interaction (Met953), which enhances the molecule's effectiveness and may further stabilize the complex.

Molecular docking simulations against ERα revealed that the crystallized inhibitor Compound 15 exhibited the most favorable Moldock score (−176.84) when compared with ribifolone C (−99.60) and jatrophone (−99.93). Compound 15 is recognized as a potent ERα inhibitor, with an IC_{50} value of 0.5 nM. The ligand efficiency (LE) values further indicated that Compound 15 possesses superior molecular efficiency relative to both diterpenes. The amino acid interaction analysis demonstrated that ribifolone C and jatrophone share a comparable interaction profile (Figure 133-J,K), predominantly characterized by hydrophobic contacts. Ribifolone C established an additional interaction with Thr347, which was not detected for jatrophone or Compound 15, while also displaying an unfavorable contact with Phe404. In contrast, Compound 15 formed a greater number of polar interactions, including hydrogen bonds with Asp351, Gly521, and His524, as well as hydrophobic contacts with Leu354 and Leu536, and π–sulfur interactions involving Phe404 and Met421. These latter interactions were not observed for the other two ligands (Figure 133-L).
